# Global, regional, national burden of colorectal cancer from 1990 to 2021, with projections of incidence to 2050: a systematic analysis of the global burden of disease study 2021

**DOI:** 10.3389/fonc.2025.1597847

**Published:** 2025-10-06

**Authors:** Shiyu Chang, Yu Chang, Fan Li, Zifeng Xu, Xiaoping Han, Yue Liu, Hongyan Li, Sileng Hu, Tongyu Tang

**Affiliations:** Department of Gastroenterology, The First Hospital of Jilin University, Changchun, Jilin, China

**Keywords:** colorectal cancer, disease burden, Global Burden of Disease Study (GBD) 2021, incidence, mortality, disability-adjusted life-years (DALYs), risk factor

## Abstract

**Background:**

Colorectal cancer (CRC) is a common malignant tumor of the digestive system, characterized by a high incidence and mortality rate. This study aimed to investigate the epidemiological characteristics of CRC between 1990 and 2021.

**Methods:**

Data on CRC were obtained from the Global Burden of Diseases, Injuries, and Risk Factors Study (GBD) 2021. We focused on the effects of age, sex, risk factors, and the socio-demographic index (SDI) on the burden of CRC. Estimated annual percentage changes (EAPCs) were calculated to evaluate changes in the age-standardized rate (ASR) of incidence, mortality and DALYs, as well as their trends in CRC burden. Frontier and health inequality analyses assessed health management potential and disease burden by country, while the Bayesian age-period-cohort (BAPC) model predicted CRC incidence patterns by age group through 2050.

**Findings:**

Global ASMR and ASDR declined, but ASIR rose overall (EAPC = 0.15), with significant gender disparities (male EAPC = 0.50 *vs*. female EAPC=-0.29). Middle SDI regions saw the steepest ASIR increase (EAPC = 1.38). In 2021, Australasia had the highest ASIR, while East Asia had elevated ASIR and ASMR. CRC burden predominantly affected ages 60–79. Dietary risks surpassed metabolic, environmental, and behavioral factors as the leading contributor to CRC burden. Frontier analysis revealed that 15 countries with the longest effective distances (indicating suboptimal health outcomes) were predominantly high-SDI nations (SDI >0.70). Projections suggest persistently high global ASIR through 2050, particularly among ages 70–74.

**Interpretation:**

CRC burden varies by gender, age, and region. While some regions show declining mortality, overall burden remains substantial, especially in middle/low-middle SDI areas. Notably, even high-SDI countries exhibit significant gaps in CRC management. Targeted public health strategies—optimizing prevention, early detection, and resource allocation—are critical to address rising incidence and disparities.

## Graphical Abstract

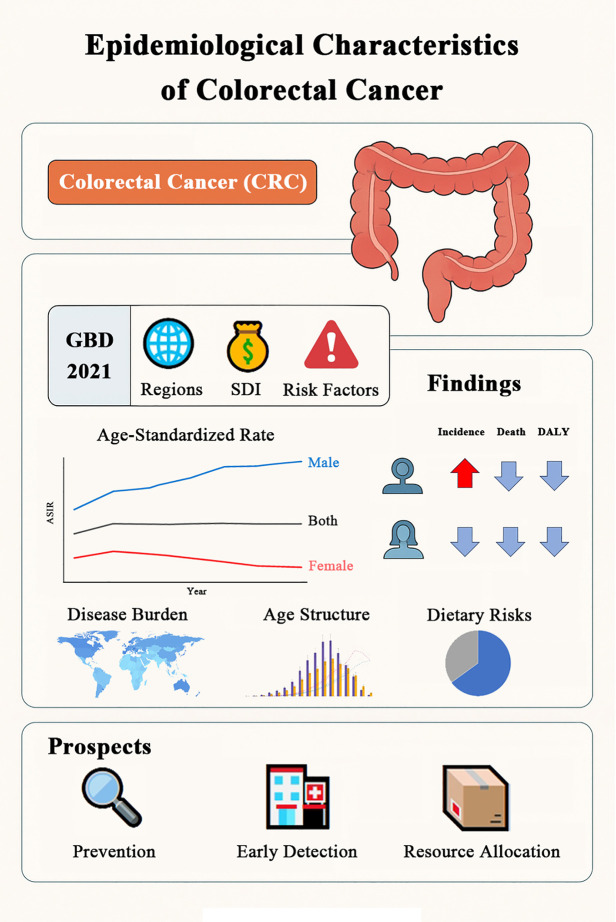

## Introduction

Colorectal cancer (CRC) is one of the most common malignant tumors globally, typically originating from the glandular epithelium of the colon or rectum. Its occurrence is driven by the interaction of various environmental, genetic, and lifestyle factors (1, 2). In recent years, with changes in global demographics, population aging and shifts in lifestyle, the incidence and mortality of CRC have steadily increased (3). According to data from the Global Burden of Disease (GBD) database, CRC has become the third most common cancer worldwide and the second leading cause of cancer-related deaths (4, 5), imposing a significant burden on global public health systems.

CRC exhibits significant geographic variation in its prevalence across the globe. The incidence is generally higher in high-income countries, while it tends to be lower in low-income nations (6). However, with the development of the socio-economy, regions with a medium socio-economic index, particularly in East Asia, Eastern Europe, and South America, are experiencing a gradual increase in CRC incidence (7). The disparities in the incidence and mortality rates of CRC across the globe are closely linked to varying levels of socio-economic development, further highlighting the widening health inequalities and the escalating burden of the disease.

In recent years, the incidence of colorectal cancer among young and middle-aged individuals has shown a consistent upward trend (8, 9). Early-onset colorectal cancer exhibits distinct pathological characteristics. The majority of young patients present with metastatic disease at their initial diagnosis, exhibit a high risk of recurrence, and have a significantly reduced life expectancy (10, 11). This phenomenon has significantly exacerbated the burden of CRC on both patients and society.

Although multiple studies have investigated the burden of CRC across different regions globally, previous research has predominantly focused on specific geographic areas and particular age groups. Utilizing the latest data from GBD 2021, this study evaluates the impact of various risk factors on the CRC burden across different countries and territories worldwide, with a particular emphasis on analyzing health inequalities related to CRC between 1990 and 2021. It explores disparities in incidence, mortality, and disease burden among diverse regions, income levels, and social groups. Additionally, predictive models were constructed to forecast future trends in CRC incidence among different sex and age subgroups. Our research aims to provide a robust scientific foundation for the development of more precise public health policies, to inform optimal resource allocation, and to enhance the accessibility of early screening and treatment.

## Method

### Data source

GBD 2021 utilizes the latest epidemiological data and refined standardized methodologies to conduct a comprehensive assessment of over 370 diseases and injuries, as well as 88 risk factors, by age and gender across 204 countries and territories. It offers detailed insights into incidence, mortality, disability-adjusted life years (DALYs), and age-standardized rates (ASRs) (12, 13). All data were meticulously sourced from reputable public databases, having undergone rigorous screening and quality control procedures to ensure their reliability and accuracy. Through meticulous data cleaning, transformation, and modelling of datasets obtained from research institutions worldwide, the GBD collaborator network has generated comprehensive estimates for a wide range of health indicators. All data are accessible via the GBD Results Tool (https://vizhub.healthdata.org/gbd-results/).

### Disability-adjusted life-years

Disability weight is defined as a quantitative measure of the severity of health loss or non-fatal disability. The calculation of Years Lived with Disability (YLD) involves multiplying the number of affected individuals by the duration of illness prior to remission or death, and then by the corresponding disability weight. Similarly, Years of Life Lost (YLL) are calculated by multiplying the number of deaths by the standard life expectancy derived from a reference life table. The DALY (Disability-Adjusted Life Year) serves as a comprehensive metric quantifying health loss attributable to both fatal and non-fatal outcomes, calculated as the sum of YLDs and YLLs (14). As a critical parameter for assessing disease burden, the loss of one year of full health equates to one DALY. The disparity between current health status and optimal health is represented by the total DALY across all populations (3).

### Socio-demographic index

Socio-Demographic Index (SDI) is a comprehensive development indicator that reflects the impact of socio-economic conditions on health outcomes across different regions. SDI is calculated as the geometric mean of several indicators, ranging from 0 to 100, which include the Total Fertility Rate for Women Under 25 (TFU25), the Average Education Level aged 15 and above (EDU15+), and the per capita Lagged Distributed Income (LDI). As a composite measure, regions with an SDI of 0 represent the theoretical lowest level of development in relation to health, while regions with an SDI of 100 represent the theoretical highest level of development. Based on SDI values, 204 countries and territories are classified into five groups: high SDI, middle-high SDI, middle SDI, low-middle SDI, and low SDI regions (3). In the GBD 2021 report, the SDI values range from 0 to 100, while our study maintained the commonly used range of 0 to 1. This discrepancy may lead to variations in SDI values across different versions of the GBD reports (15).

### Estimation of risk factor

Attributable risk factors are assessed across four levels (16). The risk factors analyzed include alcohol use, smoking, diet high in processed meat, diet high in red meat, diet low in calcium, diet low in fiber, diet low in milk, diet low in whole grains, low physical activity, high body-mass index and high fasting plasma glucose [**Supplementary Table S9**]. The percentages and specific values for CRC related deaths and DALYs can be derived using the GBD outcome tool. In this study, we applied the previously established definitions of these risk factors (17, 18).

### Definition of CRC

According to the list of International Classification of Diseases (ICD) codes mapped to non-fatal causes and injuries in the GBD 2021, colon and rectum cancer is defined as C18−C19.0, C20, C21−C21.8 (3).

### Cross-country health inequality analysis

Cross-country health inequality refers to measurable differences in health across different social, economic, geographical, or demographic characteristics, typically manifested as health disparities between different subgroups of the population (19, 20). In order to evaluate health disparities across regions with varying levels of the SDI, commonly employed indicators include the Slope Index of Inequality (SII) and the Relative Index of Inequality (RII). The SII is used to assess absolute inequality, while the RII measures relative inequality. These indices facilitate the quantification of disease prevalence, incidence, mortality rates, and DALYs across different regions (21). The SII is derived via weighted regression of health indicators across age groups, reflecting relative positions based on SDI. It typically represents group positions using the midpoint of the cumulative distribution range of SDI rankings. The Concentration Index (CI), on the other hand, is evaluated by calculating the area under the Lorenz curve, with values ranging from -1 to 1. A negative CI value indicates a lower concentration of disease burden in countries with lower SDI levels. The Lorenz curve is constructed based on the relative cumulative scores and the cumulative relative population distribution of the SDI, and is used to measure health inequality between different SDI groups (22).

### Frontier analysis

Frontier analysis is applied to assesses the performance and efficiency of decision-making units (DMUs), such as countries or territories, in transforming inputs into health outputs (23). We employ Data Envelopment Analysis (DEA) and Stochastic Frontier Analysis (SFA) to construct the efficiency frontier of best practices. The efficiency level of each DMU is then evaluated by comparing it with this frontier. When the observed ASRs are significantly lower than the efficiency frontier corresponding to the SDI category, this suggests that there may be potential for further improvement in disease burden.

### Bayesian Age-Period-Cohort

The Bayesian Age-Period-Cohort (BAPC) model is a Bayesian statistical method that analyzes and predicts demographic data by modeling the individual effects of age, period, and birth cohort (24). In the BAPC model, the age effect captures the variation in the risk of events—such as disease incidence or mortality—with increasing age. The period effect reflects the influence of environmental, policy, or other external factors that impact all individuals at a specific point in time. The cohort effect accounts for the unique characteristics and experiences shared by individuals born during a particular time period. We employed the BAPC R software package to fit the model and project global ASIR by gender and age groups up to 2050 (25).

### Auto Regressive Integrated Moving Average

In this study, we employed the Autoregressive Integrated Moving Average (ARIMA) model to predict the ASIR and ASDR of diseases. The ARIMA model is a commonly used time series analysis method that can capture trends and seasonal variations in the data for forecasting (26). The core concept of the model is to model time series data through three main components: Autoregression (AR), Differencing (I), and Moving Average (MA). The autoregressive component relies on the linear relationship with historical data, the differencing component is used to remove the trend in the data, and the moving average component corrects the model using past errors. To ensure the accuracy of the model, we performed stationarity tests during the data preprocessing phase and optimized the model parameters to achieve accurate predictions over a 15-year period.

### Statistical analysis

The burden of CRC was quantified over time and by region, sex, and age using ASR, which includes ASIR, ASMR, and ASDR. These measures take into account variations in the age structure of populations, aiming to eliminate the impact of population age composition and ensure the comparability of research indicators. In the GBD database, these indicators are estimated using the world population age standard calculated with the following formula: ASR= $\frac{\sum_{i=1}^{A}a_{i}w_{i}}{\sum_{i=1}^{A}w_{i}}\times 100,000$ , which is the sum of the age-specific rates (ai, where i represents the ith age class) and the number of persons (or weight) (wi) in the same age subgroup i of the selected reference standard population, dividing the sum of the standard population.

In addition, a generalized linear regression model was used to calculate the Estimated Annual Percentage Change (EAPC) to assess the annual average variation of ASR. The model establishes the relationship between the natural logarithm (ln) of ASR and time using the following equation, thereby capturing the temporal pattern of ASR changes. The formula for determining the EAPC and its 95% uncertainty interval (UI) is as follows: $EAPC=100*\left\{exp\left[\frac{ln\left(ASR\right)-\alpha -\epsilon }{year}\right]-1\right\}$ . Here, ln (ASR) represents the natural logarithm of ASR. A positive EAPC with the lower bound of the 95% CI indicates an upward trend in ASR, while a negative EAPC with the upper bound of the 95% CI indicates a downward trend in ASR (27).

The estimates and 95% UIs for metrics used to assess the burden of CRC were derived from the GBD 2021database (https://ghdx.healthdata.org/gbd-2021). Joinpoint regression analysis (using Joinpoint Com-mand Line Version 4.5.0.1, USA National Cancer Institute Surveillance Research Program) was employed to analyze trends of ASRs from 1990 to 2021. All statistical analyses were performed using R software (ver-sion 4.4.1; Bell Laboratories, formerly AT&T, now LucentTechnologies).

## Results

### Global burden analysis from 1990 to 2021

**Figure 1:**
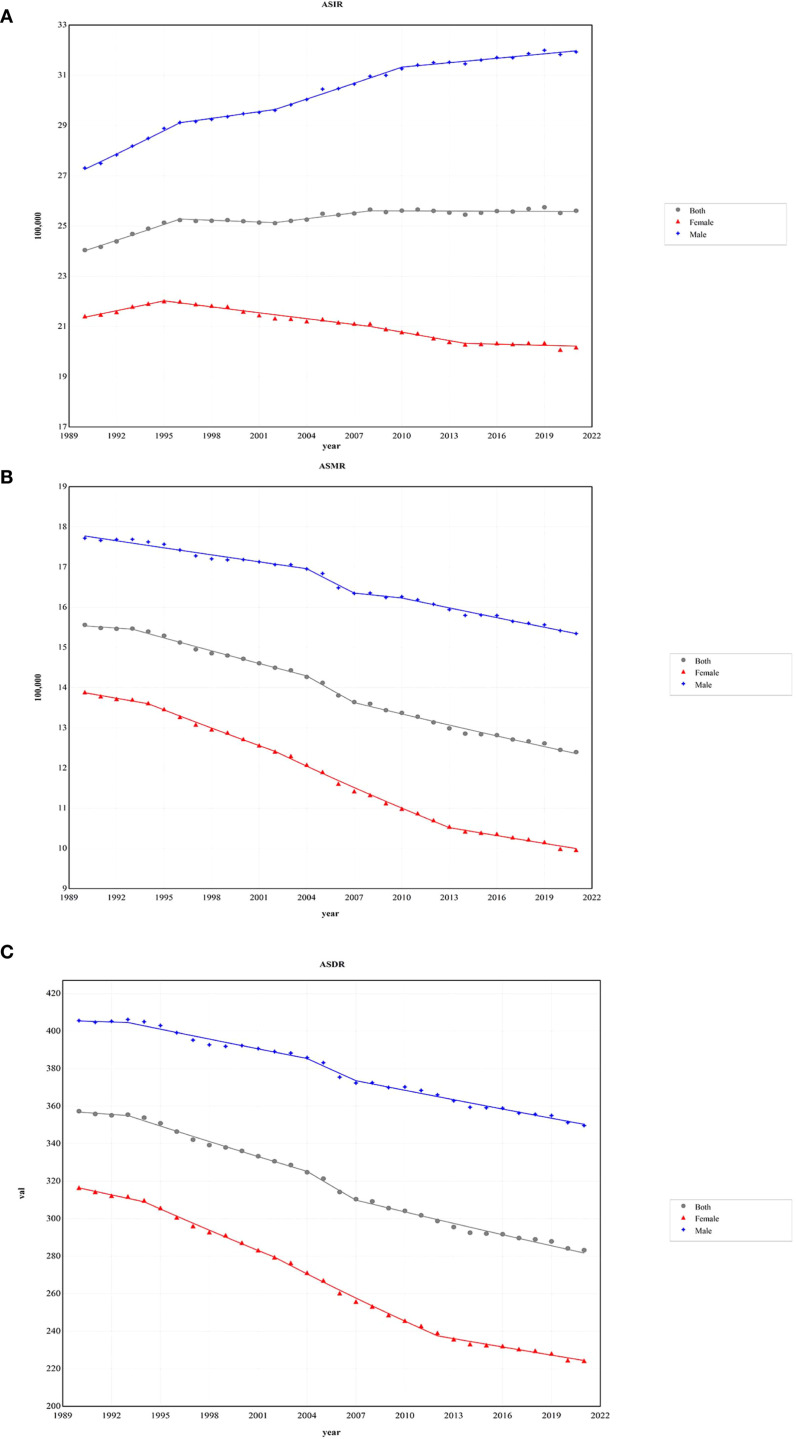
The age-standardized rates of CRC during 1990 - 2021 by sex. **(A)** The global ASIR of CRC during 1990 - 2021 by sex. **(B)** The global ASMR of CRC during 1990 - 2021 by sex. **(C)** The global ASDR of CRC during 1990 - 2021 by sex. ASIR, age-standardized incidence rate; ASMR, age-standardized deaths rate; ASDR, age-standardized disability-adjusted life-year rate; CRC, colorectal cancer.

In 2021, there were 2,194.14×10³ incidence cases of CRC, and the 95% UI ranged from 2,001.27×10³ to 2,359.39×10³, representing a significant increase of 139.39% compared to 1990. The ASIR of CRC in 2021 was 25.61/100,000 (95% UI 23.32/100,000 - 27.52/100,000), whereas in 1990, it was 24.04/100,000 (95% UI 22.54/100,000 - 25.01/100,000). The EAPC data suggest a slight increase in ASIR from 1990 to 2021, with an EAPC of 0.15 (95% CI 0.12-0.19), leading to an overall increase of approximately 6.55% in ASIR. In males, the number of CRC incidence cases in 2021 was 1,263.46×10³ (95% UI 1,146.5×10³ - 1,400.38×10³), showing a 35.76% increase compared to females. From 1990 to 2021, the annual incidence growth rate was 168.96% in males and 108.29% in females. Nevertheless, the EAPC data indicate a declining trend in the ASIR for females, with an EAPC of -0.29 (95% CI -0.34 to -0.25), while the ASIR for males reveals an increasing trend, with an EAPC of 0.50 (95% CI 0.45 to 0.54) (**Figure 1A**).

From 1990 to 2021, the number of CRC-related deaths increased by 83.07%. In 2021, the total number of CRC deaths was 1,044.07×10³ (95% UI 950.19×10³ - 1,120.17×10³), with 581.56×10³ (95% UI 528.25×10³ - 641.42×10³) in males and 462.51×10³ (95% UI 407.3×10³ - 503.54×10³) in females. Despite the rise in the number of deaths, the ASMR for CRC decreased by 20.31%, from 15.56/100,000 (95% UI 14.49/100,000 - 16.31/100,000) in 1990 to 12.4/100,000 (95% UI 11.24/100,000 - 13.31/100,000) in 2021. Additionally, females exhibited lower mortality figures and ASMR levels than males, with 462.51×10³ deaths (95% UI 407.3×10³ - 503.54×10³) and an ASMR of 9.96/100,000 (95% UI 8.78/100,000 - 10.84/100,000) (**Figure 1B**).

Globally, the DALYs for CRC increased from 14,396.66×10³ (95% UI 13,568.75×10³ - 15,166.58×10³) in 1990 to 24,401.1×10³ (95% UI 22,689.37×10³ - 26,161.52×10³) in 2021, representing an increase of 69.49%. Despite this increase, the ASDR for CRC declined, with an EAPC of -0.83 (95% CI -0.87 to -0.80). Compared to males, females experienced a greater reduction in ASDR, showing an EAPC of -1.25 (95% CI -1.30 to -1.19) (**Figure 1C**) (**Table 1**).

**Table 1:** Global incidence, deaths, and DALYs of colorectal cancer (CRC) from 1990 to 2021.

Year	Both	Male	Female
1990			
Incidence/1000(95%UI)	916.58(866.24-951.89)	469.76(445.31-492.3)	446.82(414.14-472.55)
Deaths/1000(95%UI)	570.32(536.54-597.67)	287.71(269.82-304.98)	282.61(258.89-301.42)
DALYs /1000(95%UI)	14396.66(13568.75-15166.58)	7609.7(7037.9-8139.91)	6786.95(6292.86-7304.11)
ASIR/100,000 persons(95%UI)	24.04(22.54-25.01)	27.31(25.89-28.51)	21.41(19.75-22.62)
ASMR/100,000 persons(95%UI)	15.56(14.49-16.31)	17.72(16.67-18.68)	13.89(12.68-14.8)
ASDR/100,000 persons(95%UI)	357.33(336.62-375.74)	405.58(378.49-431.93)	316.54(292.93-340.38)
2021			
Incidence/1000(95%UI)	2194.14(2001.27-2359.39)	1263.46(1146.5-1400.38)	930.68(824.67-1017.65)
Deaths/1000(95%UI)	1044.07(950.19-1120.17)	581.56(528.25-641.42)	462.51(407.3-503.54)
DALYs /1000(95%UI)	24401.1(22689.37-26161.52)	14167.25(12782.33-15683.97)	10233.85(9257.56-11064.62)
ASIR/100,000 persons(95%UI)	25.61(23.32-27.52)	31.93(29.04-35.26)	20.17(17.86-22.05)
ASMR/100,000 persons(95%UI)	12.4(11.24-13.31)	15.35(13.94-16.87)	9.96(8.78-10.84)
ASDR/100,000 persons(95%UI)	283.24(263.11-303.33)	349.67(316.68-386.64)	224.3(203.21-242.65)
1990-2021			
Incidence (%)	139.39	168.96	108.29
Death (%)	83.07	102.13	63.66
DALYs (%)	69.49	86.17	50.79
EAPC of ASIR (95% CI)	0.15(0.12 to 0.19)	0.50(0.45 to 0.54)	-0.29(-0.34 to -0.25)
EAPC of ASMR (95% CI)	-0.81(-0.84 to -0.77)	-0.50(-0.52 to -0.47)	-1.19(-1.24 to -1.14)
EAPC of ASDR (95% CI)	-0.83(-0.87 to -0.80)	-0.52(-0.55 to -0.50)	-1.25(-1.30 to -1.19)

EAPC, estimated annual percentage changes; ASIR, age-standardized incidence rate; ASMR, age-standardized mortality rate; ASDR, age-standardized disability-adjusted life-year rate, UI, uncertainty interval.

### Regional incidence, mortality, and DALYs

**Figure 2:**
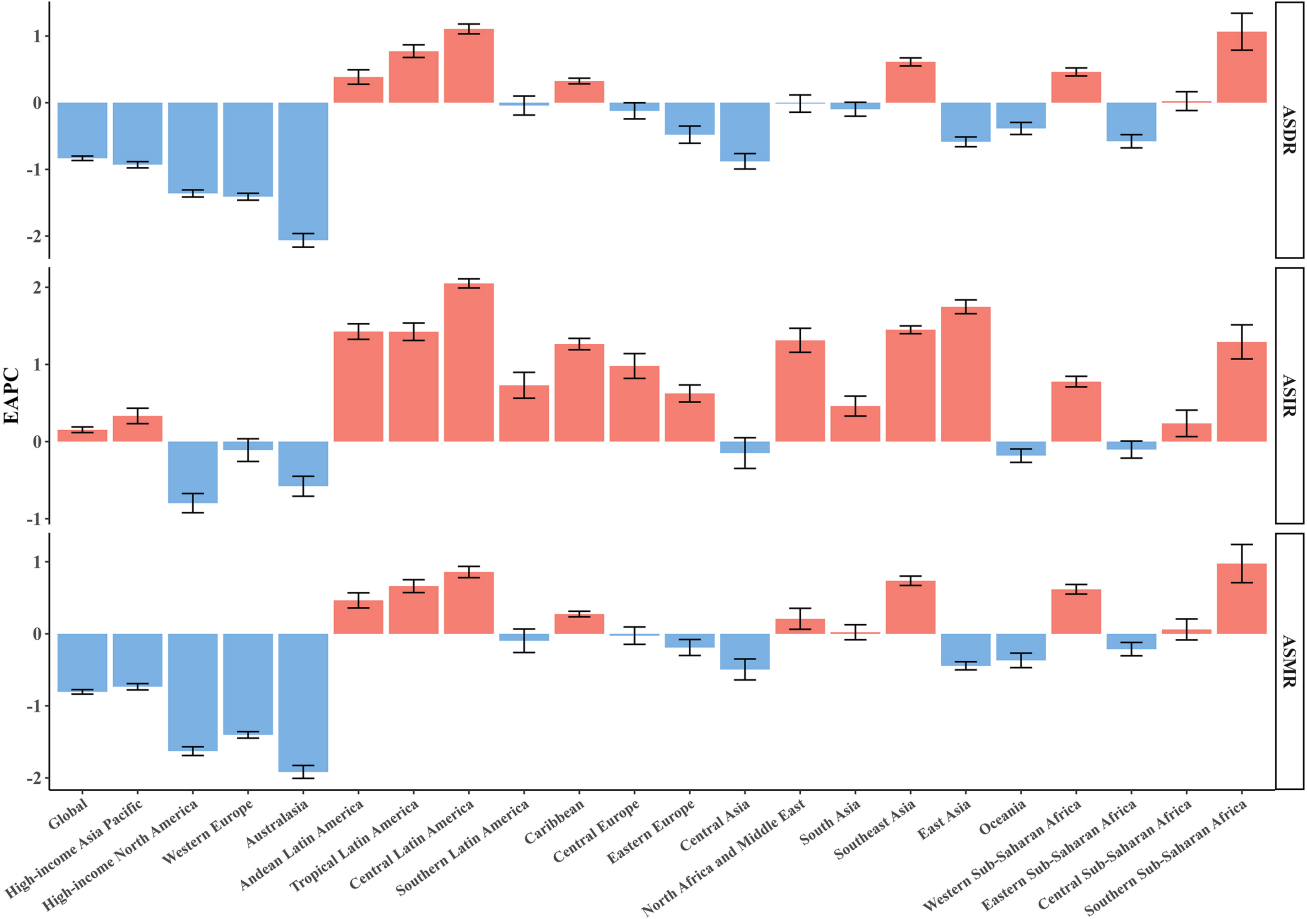
EAPCs of the ASRs for CRC in 21 regions. ASR, age-standardized rate; ASDR, age-standardized disability-adjusted life-year rate; ASIR, age-standardized incidence rate; ASMR, age-standardized mortality rate; EAPC, estimated annual percentage change; CRC, colorectal cancer.

In 2021, the five regions with the highest CRC incidence rates were East Asia, Western Europe, high-income North America, high-income Asia-Pacific, and Southeast Asia(**Table 2**). Among these, East Asia recorded the highest ASIR for CRC, at 684.93 per 100,000 (95% UI 559.52/100,000 - 823.3/100,000) (**Supplementary Table S1**). As depicted in **Figure 2** only six out of the 21 regions experienced a statistically significant decline in ASIR (EAPC< 0). Among the regions where ASIR increased, Central America had the largest rise (EAPC = 2.05, 95% UI 1.99 to 2.11), followed by East Asia and Southeast Asia, which saw EAPCs of 1.75 (95% UI 1.65 to 1.84) and 1.45 (95% UI 1.40 to 1.50), respectively. In contrast, the region of high-income North America reported the greatest reduction in ASIR (EAPC = -0.80; 95% UI -0.93 to -0.67) (**Supplementary Table S2**) (**Figure 2**).

**Table 2:** Incidence, deaths, and DALYs for colorectal cancer across 21 regions in 2021.

Location	Incidence/1000 (95%UI)			Deaths/1000 (95%UI)			DALYs/1000 (95%UI)		
	Both	Male	Female	Both	Male	Female	Both	Male	Female
Andean Latin America	8.45(6.7-10.53)	4.01(3.17-5.01)	4.44(3.44-5.59)	5.78(4.6-7.05)	2.62(2.06-3.23)	3.15(2.44-3.94)	136.18(108.53-167.98)	64.22(50.37-79.98)	71.96(55.8-90.99)
Australasia	23.28(20.5-26.41)	12.88(11.55-14.44)	10.4(8.77-12.12)	8.28(7.18-9.41)	4.48(3.97-4.99)	3.79(3.12-4.46)	166.74(147.52-186.89)	94.16(84.35-104.24)	72.58(62.19-83.75)
Caribbean	18.48(16.07-20.94)	8.98(7.71-10.28)	9.5(8.19-10.84)	7.88(6.87-8.97)	3.69(3.19-4.21)	4.19(3.61-4.74)	179.75(155.87-205.42)	87.46(75.27-100.31)	92.29(79.6-105.86)
Central Asia	8.89(7.94-9.81)	4.7(4.22-5.19)	4.19(3.7-4.63)	6.15(5.49-6.77)	3.22(2.89-3.55)	2.92(2.6-3.24)	169.95(151.54-187.71)	92.06(82.25-102.07)	77.89(68.93-86.92)
Central Europe	85.87(79.29-92.79)	50.74(46.58-54.56)	35.13(31.85-38.34)	51.84(47.75-55.68)	29.65(27.31-31.72)	22.19(19.92-24.11)	1087.56(1003.63-1168.38)	654.66(601.4-701.63)	432.91(394.05-468.76)
Central Latin America	44.55(39.67-49.72)	27.28(23.96-30.71)	17.28(15.04-19.57)	22.93(20.34-25.52)	11.46(10.08-12.85)	11.48(10.01-12.9)	594.12(529.27-662.37)	307.31(270.74-345.96)	286.81(250.38-326.05)
Central Sub-Saharan Africa	4.21(3.21-5.57)	2.22(1.7-3.15)	1.99(1.44-2.7)	3.69(2.8-4.93)	1.93(1.48-2.77)	1.75(1.26-2.41)	109.62(82.69-146.97)	58.71(44.12-85.42)	50.91(36.04-69.84)
East Asia	684.93(559.52-823.3)	434.92(335.59-557.41)	250(191.88-316.82)	287.88(235.56-343.28)	181.74(141.26-233.67)	106.14(82.11-133.91)	7149(5822.94-8561.08)	4670.84(3618.69-6028.07)	2478.16(1922.58-3147.97)
Eastern Europe	113.25(104.41-122.49)	52.08(46.32-57.53)	61.17(54.78-67.32)	64.37(59.12-69.82)	29.96(26.66-33.06)	34.41(30.66-38)	1465.1(1343.98-1600.59)	728.28(645.72-808.28)	736.83(659.14-818.41)
Eastern Sub-Saharan Africa	17.95(15.71-20.78)	10.4(8.74-12.56)	7.56(6.43-8.91)	15.97(13.94-18.32)	9.28(7.78-11.14)	6.69(5.74-7.84)	444.25(385.8-525.46)	257.61(215.55-316.87)	186.64(157.87-223.41)
High-income Asia Pacific	207.28(179.5-223.33)	120.86(110.81-127.92)	86.42(68.62-97.02)	80.69(67.27-88)	41.91(37.84-44.24)	38.78(28.95-44.41)	1441.63(1272.5-1549.76)	825.74(766.95-873.75)	615.89(498.76-688.15)
High-income North America	244.68(226.55-256.38)	132.66(125.1-138.48)	112.03(100.4-119.31)	85.87(77.87-90.93)	45.05(42.18-47.12)	40.82(35.59-43.98)	1907.93(1788.11-2003.82)	1064.2(1013.26-1109.01)	843.73(771.64-895.28)
North Africa and Middle East	66.09(58.13-74.94)	34.06(29.21-39.1)	32.03(27.63-36.55)	37.39(32.76-42.27)	20.88(17.78-23.99)	16.51(14.19-18.92)	1012.65(886.2-1154.5)	565.73(479.89-650.9)	446.92(383.64-517.3)
Oceania	0.48(0.41-0.56)	0.23(0.19-0.27)	0.25(0.21-0.3)	0.38(0.32-0.45)	0.18(0.15-0.21)	0.2(0.16-0.24)	11.64(9.76-13.75)	5.55(4.58-6.47)	6.09(4.96-7.36)
South Asia	85.12(76.61-95.25)	43.68(37.09-50.62)	41.43(35.39-48)	66.94(60.2-74.84)	34.58(29.42-40.01)	32.37(27.74-37.6)	1908.67(1711.98-2155.06)	963.9(817.97-1128.59)	944.77(808.41-1105.69)
Southeast Asia	116.94(101.26-132.26)	67.64(55.68-79.39)	49.3(42.18-57.04)	79.42(68.45-89.29)	45.32(37.94-52.81)	34.1(29.33-39.34)	2166.65(1868.88-2456.35)	1269.88(1048.3-1488.16)	896.77(768.38-1051.3)
Southern Latin America	24.75(21.92-27.56)	13.19(11.65-14.9)	11.55(10.08-13.05)	16.12(14.31-18)	8.4(7.44-9.46)	7.72(6.64-8.7)	349.58(311.37-391.89)	191.25(168.87-216.24)	158.33(138.62-178.02)
Southern Sub-Saharan Africa	7.62(6.88-8.47)	3.85(3.33-4.46)	3.77(3.35-4.24)	6.13(5.55-6.79)	3.03(2.64-3.48)	3.1(2.76-3.49)	166.06(149.96-186.75)	86.43(74.53-101.42)	79.63(70.27-89.87)
Tropical Latin America	44.24(40.86-47.14)	22.6(21.01-24.15)	21.65(19.53-23.23)	29.41(26.98-31.34)	14.49(13.4-15.49)	14.92(13.33-16.05)	744.96(694.96-786.85)	376.37(352.6-400.47)	368.59(337.75-393.49)
Western Europe	375.46(337.71-401.85)	210.49(195.07-223.28)	164.97(141.41-179.23)	156.64(136.86-169.94)	84.34(77.28-90.1)	72.29(59.28-80.1)	2912.36(2641.02-3112.52)	1660.03(1547.4-1761.99)	1252.33(1086.12-1366.66)
Western Sub-Saharan Africa	11.62(9.67-13.67)	6(4.91-7.25)	5.62(4.45-6.89)	10.31(8.67-12.1)	5.34(4.42-6.43)	4.97(3.99-6.04)	276.69(224.57-328.43)	142.86(116.01-174.89)	133.84(103.41-166.69)

DALYs, disability-adjusted life-years; UI, uncertainty interval.

Across all regions, East Asia had the highest number of CRC-related deaths [287.88×10³ (95% UI 235.56×10³ – 343.28×10³)]. The highest ASMR was recorded in Central Europe [22.58/100,000 (95% UI 20.81/100,000 - 24.28/100,000)], followed by South America [18.13/100,000 (95% UI 16.12/100,000 - 20.25/100,000)] and Eastern Europe [18.05/100,000 (95% UI 16.58/100,000 - 19.56/100,000)]. From 1990 to 2021, Australasia exhibited the largest decline in ASMR (EAPC = -1.92; 95% CI -2.01 to -1.82), while Southern Sub-Saharan Africa experienced the largest increase in ASMR (EAPC = 1.29; 95% CI 1.06 to 1.52).

Similarly to incidence and mortality rates, East Asia also reported the highest CRC DALYs [7,149×10³ (95% UI 5,822.94×10³ - 8,561.08×10³)]. The highest ASDR was observed in Central Europe, at 506.48/100,000 (95% UI 467.97/100,000 - 544.57/100,000), followed by Eastern Europe [424.54/100,000 (95% UI 389.69/100,000 - 463.67/100,000)] and Southern Latin America [407.99/100,000 (95% UI 363.05/100,000 - 457.56/100,000)]. From 1990 to 2021, Central Latin America showed the greatest increase in ASDR (EAPC = 1.11; 95% CI 1.03 to 1.18), while Australasia experienced the largest decrease (EAPC = -2.06; 95% CI -2.17 to -1.96).

### National incidence, mortality, and DALYs

**Figure 3:**
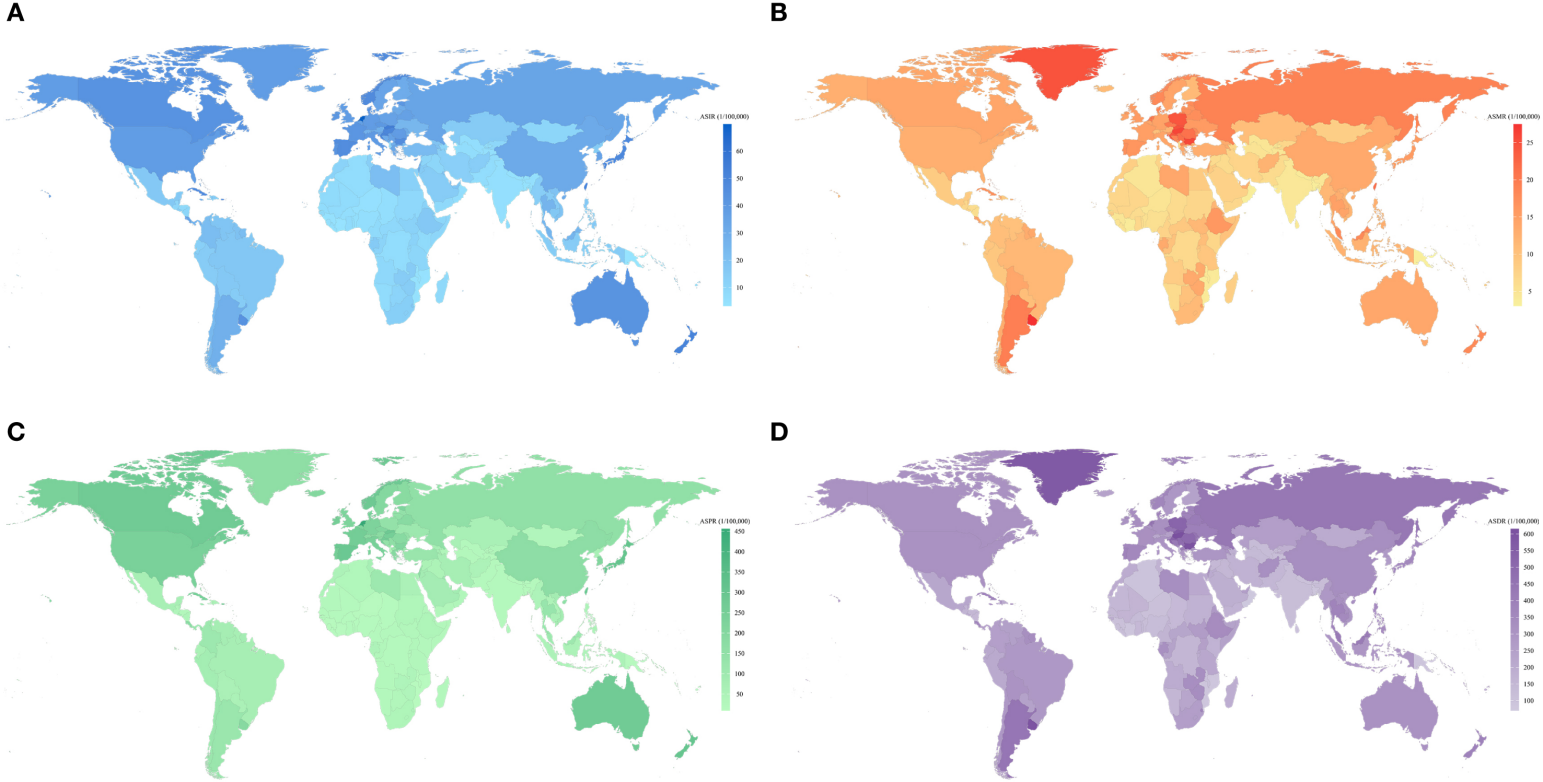
Global map of ASIR **(A)**, ASMR **(B)**,ASPR **(C)** and ASDR **(D)** of CRC for both sexes by 204 countries and territories in 2021. ASIR, age-standardized incidence rate; ASMR, age-standardized mortality rate; ASDR, age-standardized disability-adjusted life-year rate; ASPR, age-standardized prevalence rate; CRC, colorectal cancer.

In 2021, the three countries with the highest CRC incidence were China [658,321.4 (95% UI 798,063–531,995)], the United States [214,114.6 (95% UI 225,186.7–197,870.7)], and Japan [171,043.3 (95% UI 184,889–148,912.5)] (**Supplementary Table S3**). The highest ASIRs for CRC were observed in the Netherlands [69.80/100,000 (95% UI 62.21/100,000–76.79/100,000)], Monaco [68.33/100,000 (95% UI 54.05/100,000–83.19/100,000)], and Bermuda [61.79/100,000 (95% UI 51.46/100,000–77.11/100,000)] (**Supplementary Table S4**) (**Figure 3A**). Between 1990 and 2021, the most pronounced increases in CRC ASIR were recorded in Costa Rica (EAPC = 3.05; 95% CI 2.82 to 3.28), Lesotho (EAPC = 3.02; 95% CI 2.58 to 3.46), Cabo Verde (EAPC = 2.93; 95% CI 2.54 to 3.32), Egypt (EAPC = 2.82; 95% CI 2.56 to 3.08), and El Salvador (EAPC = 2.79; 95% CI 2.50 to 3.09). A declining trend in ASIR (EAPC< 0) was observed in 38 countries and regions, with the most notable reductions seen in Tajikistan (EAPC = -1.52; 95% CI -1.75 to -1.30), Austria (EAPC = -1.25; 95% CI -1.40 to -1.10), Ethiopia (EAPC = -1.15; 95% CI -1.37 to -0.93), Rwanda (EAPC = -0.97; 95% CI -1.28 to -0.66), and Greenland (EAPC = -0.95; 95% CI -1.04 to -0.87) (**Supplementary Table S5**).

The highest number of CRC-related deaths was reported in China [275,129.23 (95% UI 223,378.58–330,960.39)], the United States [75,087.06 (95% UI 68,061.57–79,710.66)], and Japan [67,923.61 (95% UI 56,450.94–74,359.76)]. The highest ASMRs for CRC were recorded in Uruguay [27.46/100,000 (95% UI 24.25/100,000–30.91/100,000)], Hungary [26.01/100,000 (95% UI 21.73/100,000–31.13/100,000)], and Bulgaria (**Figure 3B**). Between 1990 and 2021, the most substantial increases in CRC ASMR were observed in Lesotho (EAPC = 2.96; 95% UI 2.51 to 3.41), Cabo Verde (EAPC = 2.49; 95% UI 2.06 to 2.92), and Egypt (EAPC = 2.15; 95% UI 1.84 to 2.46). In contrast, Austria (EAPC = -2.42; 95% UI -2.48 to -2.37), Germany (EAPC = -2.18; 95% UI -2.30 to -2.06), and Israel (EAPC = -2.13; 95% UI -2.45 to -1.81) exhibited the most significant declines in CRC ASMR.

The highest DALYs were recorded in China [6,848,389.89 (95% UI 5,513,406.57−8,284,228.27)], the United States of America [1,692,400.79 (95% UI 1,586,159.48−1,776,257.87)], and India [1,541,696.22 (95% UI 1,369,023.49−1,755,414.62)]. Hungary [614.96/100,000 (95% UI 519.37/100,000−736.20/100,000)], Bulgaria [605.00/100,000 (95% UI 493.22/100,000−726.67/100,000)], and Uruguay [598.78/100,000 (95% UI 533.29/100,000−672.27/100,000)] had the highest ASDRs for CRC (**Figure 3D**). The greatest increase in ASDR occurred in Lesotho (EPAC = 3.20; 95% CI 2.70 to 3.69), Cabo Verde (EPAC = 2.14; 95% CI 1.73 to 2.55), and Paraguay (EPAC = 1.99; 95% CI 1.81 to 2.18). However, the greatest decrease in ASDR occurred in Maldives (EPAC=-2.49; 95% CI -2.63 to -2.35), Austria (EPAC=-2.46; 95% CI -2.52 to -2.40), and Singapore (EPAC=-2.18; 95% CI -2.40 to -1.97). Furthermore, China and India had the largest number of incident cases, deaths, and DALYs. Nauru and Haiti had the highest ASMR and ASDR for CRC.

### Burden of CRC based on SDI

**Figure 4:**
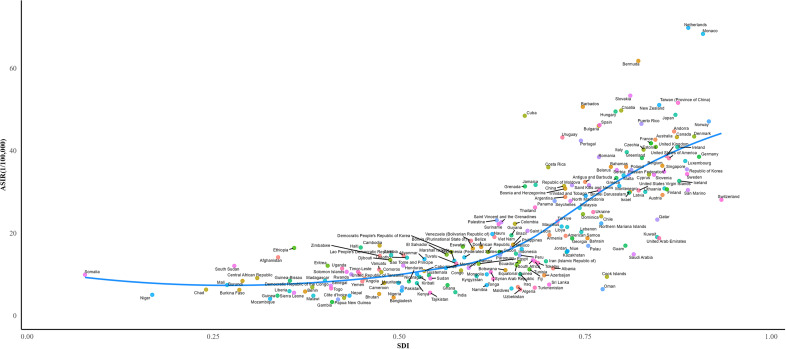
ASIR for CRC of 204 countries and territories in 2021 according to the SDI. ASIR, age-standardized incidence rate; SDI, socio-demographic index.

The majority of incidence cases, deaths, and DALYs were primarily concentrated in regions with middle-high and high SDI levels(**Table 3**). ASIR exhibited a significant positive correlation with SDI across all regions (**Figure 4**). ASMR and ASDR were positively correlated with SDI when< 0.8, showing a marked upward trend with increasing SDI levels. However, when the index exceeded 0.8, the correlation between ASMR, ASDR, and SDI turned negative, meaning that as the index increased, ASMR and ASDR levels declined (**Supplementary Figure S2A**). In Australasia, high-income North America, and Western Europe, ASIR, ASMR, and ASDR all demonstrated a significant negative correlation with SDI. Similarly, ASMR and ASDR in Eastern Europe and high-income Asia-Pacific regions also exhibited a notable negative correlation (**Supplementary Figures S3A-C**).

**Table 3:** EAPC of ASIR, ASDR, and ASMR for CRC in countries with five SDI levels from 1990 to 2021.

Region	ASIR/100,000 persons (95% UI) (1990/2021)	EAPC of ASIR (95%CI)	ASMR/100,000 persons (95% UI) (1990/2021)	EAPC of ASMR (95%CI)	ASDR/100,000 persons (95% UI) (1990/2021)	EAPC of ASDR (95%CI)
Low SDI	7.32(5.82-8.36)/7.39(6.65-8.19)	-0.06(-0.18 to 0.06)	7.21(5.75-8.21)/6.88(6.18-7.62)	-0.22(-0.33 to -0.12)	181.87(142.04-208.32)/162.29(145.25-181.89)	-0.49(-0.59 to -0.39)
Low-middle SDI	6.15(5.36-6.94)/8.19(7.51-8.96)	0.96(0.93 to 0.99)	5.72(4.99-6.45)/6.55(6.02-7.17)	0.48(0.45 to 0.51)	149.42(129.02-169.5)/166.47(151.97-182.5)	0.37(0.34 to 0.40)
Middle SDI	12.9(11.65-14.16)/19.55(17.14-22.04)	1.38(1.30 to 1.47)	10.79(9.76-11.82)/10.64(9.42-11.84)	-0.10(-0.13 to -0.06)	273.63(246.6-302.13)/259.35(230.17-288.24)	-0.22(-0.28 to -0.17)
High-middle SDI	25.58(24.12-26.81)/34(30.33-37.95)	0.93(0.89 to 0.98)	18.12(17.02-19.01)/15.71(14.14-17.25)	-0.52(-0.59 to -0.46)	436.82(410.01-460.37)/364.61(329.26-404.77)	-0.69(-0.74 to -0.63)
High SDI	42.79(40.55-44.07)/40.52(37.45-42.45)	-0.25(-0.36 to -0.15)	21.86(20.43-22.64)/15.02(13.58-15.92)	-1.31(-1.35 to -1.27)	490.5(470.41-505.01)/338.23(316.75-354.91)	-1.28(-1.32 to -1.25)

EAPC, estimated annual percentage changes; ASIR, age-standardized incidence rate; ASMR, age-standardized mortality rate; ASDR, age-standardized disability-adjusted life-year rate; CRC, colorectal cancer; SDI, socio-demographic index, UI uncertainty interval.

The EAPC was calculated for incidence, mortality, and DALY rates among age groups with a high CRC burden across different SDI regions from 1990 to 2021, revealing significant variations in EAPC across SDI regions (**Supplementary Figure S4**). Against the backdrop of a global decline in mortality rates [EAPC in male:-0.50(95%CI -0.52 to -0.47); EAPC in female: -1.19 (95%CI -1.24 to -1.14)] the most pronounced decrease in mortality was observed in females aged 70–74 years in high-SDI regions (EAPC = -2.01; 95% CI: -2.11 to -1.91). Notably, low-middle SDI regions exhibited a significant gender disparity. In middle SDI regions, the mortality rate among males aged 60–64 years showed a notable increase (EAPC = 0.39; 95% CI 0.33 to 0.45), whereas females in the same age group exhibited a declining trend (EAPC = -0.89; 95% CI -0.99 to -0.79). The changes in DALYs closely mirrored the trend in mortality. In high SDI regions, females aged 65–69 years had an EAPC for DALYs of -1.88 (95% CI: -1.95 to -1.81), while males of the same age group in lower-middle SDI regions exhibited a significant increase in DALYs (EAPC = 0.56; 95% CI: 0.48 to 0.63).The incidence trends showed a stark divergence: in middle SDI regions, the estimate annual increase in incidence among males aged 70–74 years reached 2.22% (95% CI: 2.16–2.28), significantly higher than in other regions, whereas females aged 70–74 years in high SDI regions experienced the largest decline in incidence (EAPC = -0.87, 95% CI: -1.00 to -0.74). Overall, the burden of CRC in terms of DALYs and mortality demonstrated a downward trend in high SDI, high-middle, and low SDI regions, whereas low-middle SDI regions exhibited an increasing trend. In terms of incidence, CRC showed a significant rise in low-middle, middle, and high-middle SDI regions.

**Figure 5:**
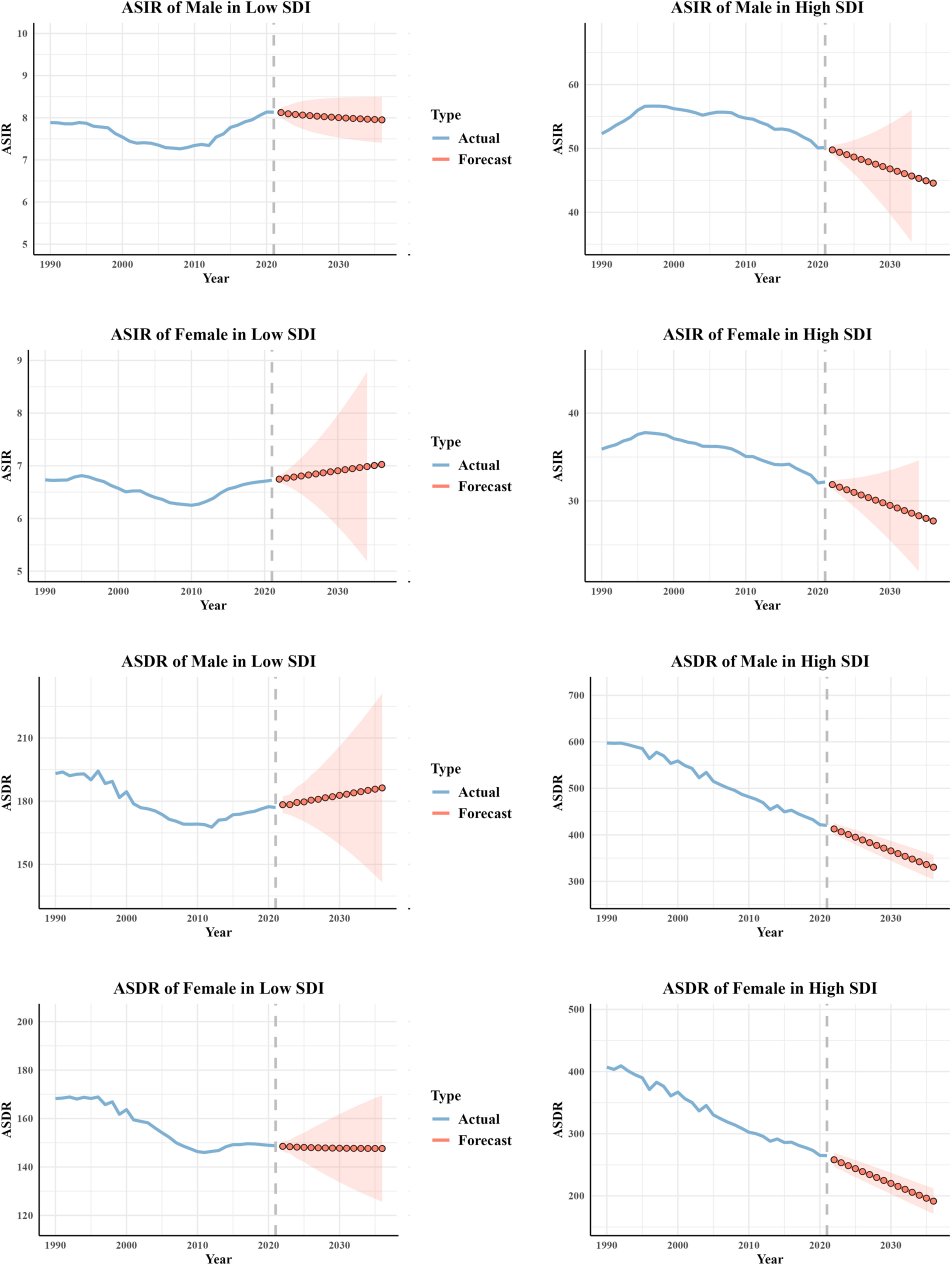
Prediction of CRC ASIR and ASDR for different genders in low SDI and high SDI regions over the next 15 years. ASIR, age-standardized incidence rate; ASMR, age-standardized mortality rate; ASDR, age-standardized disability-adjusted life-year rate; CRC, colorectal cancer.

The ARIMA model was employed to forecast ASIR and ASDR in low and high SDI regions over the next 15 years. The results indicated a slight increase in ASIR for both males and females in low-SDI regions, whereas a declining trend was observed in areas with high development levels. Correspondingly, ASDR in less developed regions remained stable or showed a slight increase, particularly among males. In contrast, more developed regions exhibited a continuous decline in ASDR, with the downward trend especially pronounced in the male population. (**Figure 5**).

### Burden of CRC based on age and sex

In 2021, the incidence of CRC was predominantly observed in the 60–79 age subgroups, with the highest concentration of prevalent cases found in the 55–74 age group (**Supplementary Figure S5A**). The incidence cases and ASIR for males exceeded those for females in all age groups, except for the 90 and above age group. A similar pattern was seen for male incidence, mortality, ASMR, and, age-standardized prevalence rate (ASPR) all of which were higher than those for females in nearly all age groups. The number of prevalent cases and deaths from CRC in females was only observed to be higher than that in males in the age group of 85 and above. Overall, the incidence, prevalence, and mortality exhibited the highest rates in the age groups of 70-74, 65−69, and 70–74 years, respectively. Therefore, the disease burden of CRC was predominantly concentrated within the age group of 65–74 years (**Supplementary Figures S5B, C**).

### Attributable risk factors

In 2021, the DALYs attributable to all risk factors for CRC amounted to 1378.40×10^4^ (95% UI: 903.87×10^4^- 1753.78×10^4^) (**Table 4**). Across all genders, the primary risk factors for CRC were diet low in whole grains, diet low in milk, and diet high in red meat. Compared to other behavioral and metabolic risks, dietary risks emerged as the leading risk factor for CRC(**Supplementary Table S8**). Diet low in whole grains was identified as the predominant risk factor contributing to CRC-related disability and mortality.

**Table 4:** Attributable DALYs and ASDR by colorectal cancer risk factors in 2021.

Risk factor	DALYs/10,000 (95% UI)			ASDR/100,000 persons (95% UI)		
	Both	Male	Female	Both	Male	Female
All risk factors	1378.4 (903.87 - 1753.78)	806.39 (537.97 - 1019.85)	572.01 (359.02 - 739.81)	159.81 (104.72 - 203.37)	198.83 (132.93 - 251.89)	124.97 (78.32 - 161.66)
Behavioral risks						
Alcohol use	142.53 (112.25 - 176.94)	119.78 (93.28 - 148.89)	22.75 (17.41 - 28.87)	16.44 (12.95 - 20.43)	29.14 (22.67 - 36.29)	4.99 (3.83 - 6.34)
Tobacco						
Smoking	123.57 (77.36 - 170.82)	104.97 (66.27 - 146.47)	18.59 (11.6 - 26.05)	14.12 (8.84 - 19.5)	25.2 (15.92 - 34.99)	4.06 (2.53 - 5.68)
Dietary risks						
Diet high in processed meat	130.16 (-31.03 - 266.41)	72.29 (-17.3 - 149.54)	57.87 (-13.84 - 119.27)	15.11 (-3.6 - 30.93)	17.9 (-4.27 - 37.03)	12.63 (-3.02 - 26.02)
Diet high in red meat	355.22 (-0.13 - 721.4)	208.28 (-0.08 - 428.57)	146.94 (-0.05 - 298.12)	41.19 (-0.02 - 83.67)	51.39 (-0.02 - 105.75)	32.12 (-0.01 - 65.18)
Diet low in calcium	212.89 (156.55 - 267.25)	93.97 (68.01 - 124.19)	118.93 (86.57 - 150.21)	24.7 (18.17 - 31.02)	22.95 (16.61 - 30.33)	26.13 (19.01 - 32.99)
Diet low in fiber	30.57 (13.51 - 46.99)	16.96 (7.46 - 26.34)	13.61 (6.01 - 21.05)	3.58 (1.58 - 5.5)	4.21 (1.85 - 6.56)	3 (1.32 - 4.64)
Diet low in milk	370.65 (101.06 - 613.8)	171.28 (46.79 - 289.65)	199.37 (54.23 - 329.45)	42.99 (11.73 - 71.23)	42.02 (11.49 - 70.99)	43.69 (11.87 - 72.18)
Diet low in whole grains	432.72 (175.49 - 657.82)	252.8 (102.09 - 387.64)	179.92 (73.63 - 271.75)	50.19 (20.37 - 76.3)	62.39 (25.2 - 95.64)	39.36 (16.1 - 59.45)
Low physical activity	132.97 (83.12 - 183)	57.44 (34.84 - 80.07)	75.53 (46.39 - 102.37)	15.69 (9.81 - 21.54)	15.03 (9.21 - 20.93)	16.29 (9.99 - 22.09)
Metabolic risks						
High body-mass index	236.47 (102.16 - 375.23)	126.89 (54.48 - 202.23)	109.58 (47.43 - 172.8)	27.33 (11.8 - 43.37)	31.09 (13.36 - 49.47)	23.96 (10.36 - 37.75)
High fasting plasma glucose	175.09 (90.06 - 265.8)	102.99 (52.07 - 157.38)	72.1 (36.85 - 109.43)	20.31 (10.46 - 30.81)	25.76 (13.05 - 39.44)	15.59 (7.97 - 23.67)

ASDR, age-standardized disability-adjusted life-year rate; UI, uncertainty interval.

**Figure 6:**
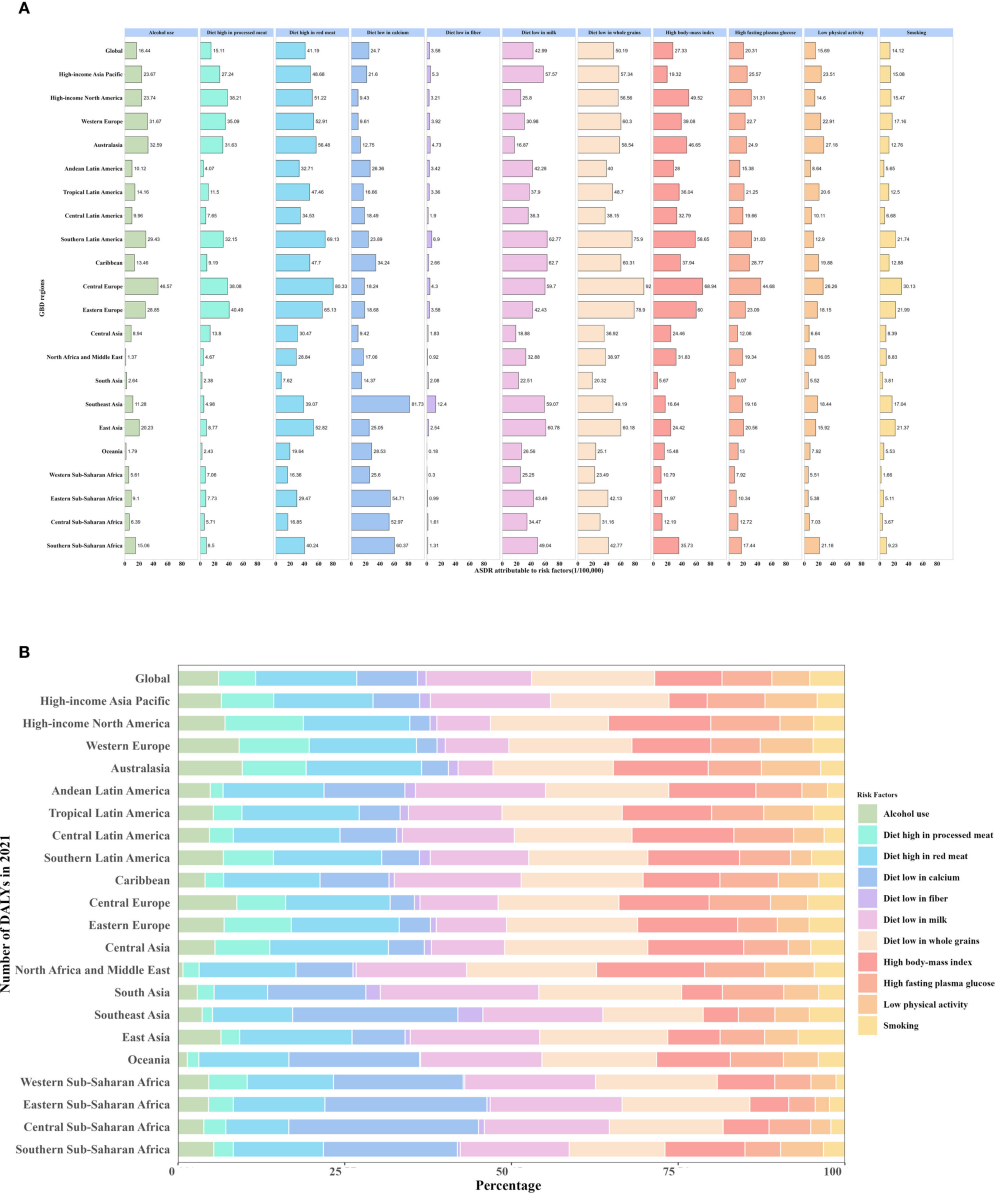
Attributable DALYs by CRC risk factors in 21 regions in 2021. **(A)** Numbers of ASDRs attributable to CRC risk factors in 21 regions in 2021. **(B)** Percentage of DALYs attributable to CRC risk factors in 21 regions in 2021. ASDR, age-standardized disability-adjusted life-year rate; CRC, colorectal cancer.

Based on GBD 2021, the impact of risk factors on DALYs varies significantly across different SDI regions and geographical areas. Across all regions, the proportion of DALYs attributed to inadequate whole grain intake was relatively similar, ranging from 18.0% to 19.4%. In middle-high and high SDI regions, metabolic risk factors contributed more to DALYs compared to other regions, particularly, high body-mass index (BMI) accounted for 11.3% and 11.6% of DALYs, while high fasting plasma glucose accounted for 7.3% and 8.63%, respectively. Additionally, diet high in red meat was a prominent risk factor in these regions, contributing 16.4% in middle-high SDI regions and 15.8% in high SDI regions. In contrast, low-middle and low SDI regions were more susceptible to the impact of dietary imbalances, with low calcium intake emerging as a major risk factor for CRC, accounting for 17.3% and 23.4% of DALYs, respectively. As SDI levels decreased, the proportion of CRC risk attributed to low calcium intake and dairy deficiency gradually increased (**Supplementary Figure S6**). From geographical perspective, the DALYs risk associated with inadequate calcium intake was significantly higher than the global average (9.1%) in Eastern Sub-Saharan Africa (24.3%), Southeast Asia (24.8%), and Central Sub-Saharan Africa (28.5%). Meanwhile, the risk attributed to insufficient milk intake in South Asia was also notably high at 23.8%, exceeding the global average (15.8%). However, in these regions, the proportion of risk associated with high red meat intake was lower than the global average (8.0% *vs*. 15.2% globally). In contrast, in High-income North America (3.0%/8.0%), Australasia (3.1%/9.5%), Western Europe (4.0%/5.2%), Central Europe (3.6%/11.7%), Eastern Europe (4.7%/10.5%), and Central Asia (5.4%/11.0%), the risks associated with low calcium and low milk intake were significantly lower than the global average (9.1%/15.8%) (**Figure 6**).

Similarly, notable differences in mortality attributable to risk factors were observed across regions with different SDI levels. The proportion of risk attributed to insufficient whole grain intake was relatively consistent across regions with varying SDI levels, ranging from 18.0% to 19.2%. Metabolic risk factors were more prominent in middle-high and high SDI regions, where high BMI contributed 11.1% and 10.9% of DALYs, and high fasting plasma glucose accounted for 7.8% and 9.1%. Additionally, high red meat intake was also a significant risk factor in these SDI regions, contributing 16.2% and 15.7%. In contrast, low-middle and low SDI regions were more susceptible to the impact of an unbalanced diet, with low calcium intake emerging as a major CRC risk factor, accounting for 17.6% and 24.0%, respectively. Moreover, as SDI levels decreased, the proportion of CRC risk attributed to low calcium intake and dairy deficiency gradually increased (**Supplementary Figure S7**). In terms of geographical differences, the proportion of DALYs risk attributed to insufficient calcium intake was significantly higher than the global average (8.8%) in Southeast Asia (24.8%), Eastern Sub-Saharan Africa (25.0%), and Central Sub-Saharan Africa (28.6%). Similarly, the risk proportion associated with insufficient milk intake in South Asia was notably high (22.9%, Global 15.6%), whereas the proportion of risk attributed to high red meat intake was lower than the global average (7.8%, Global 15.1%).In High-income North America (3.4%/8.4%), Australasia (3.4%/9.9%), Western Europe (4.4%/5.8%), Central Europe (4.0%/12.0%), Eastern Europe (5.1%/10.7%), and Central Asia (5.5%/11.0%), the risks associated with low calcium and low milk intake were considerably lower than the global average (8.8%/15.6%) (**Supplementary Figure S8**).

**Figure 7:**
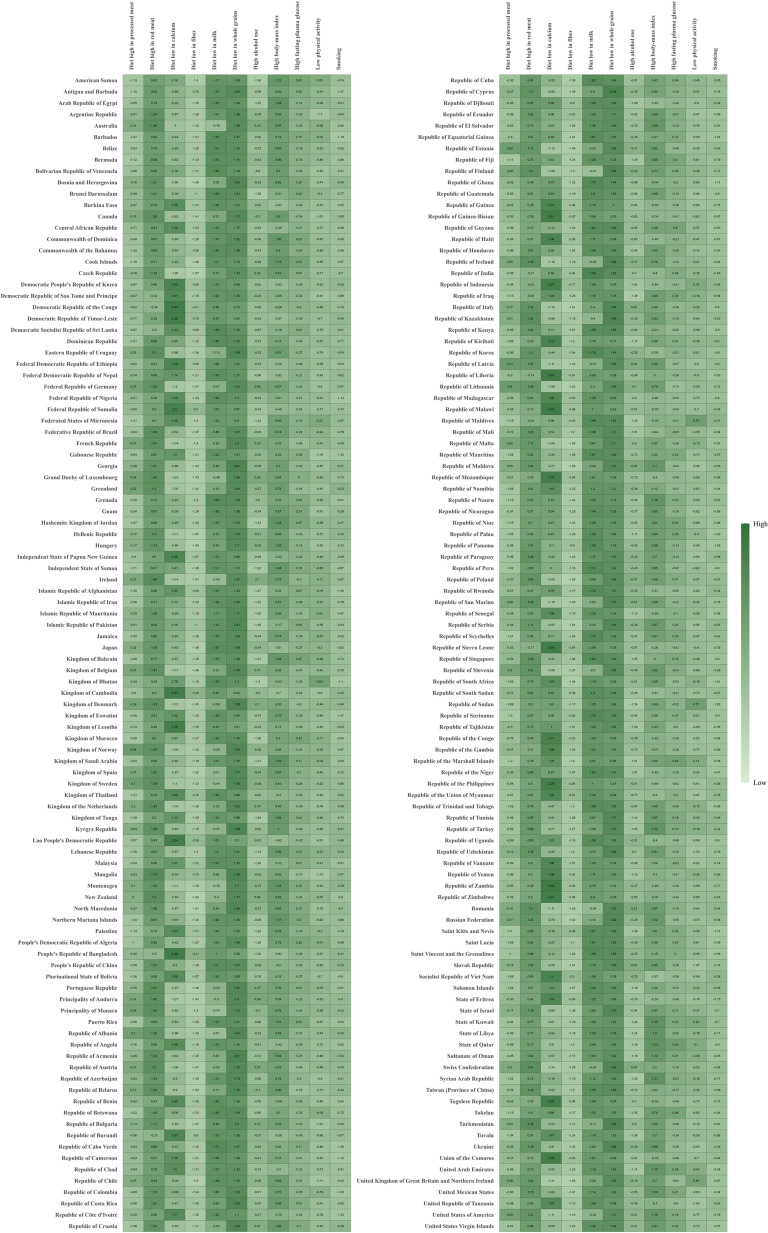
Attributable age-standardized DALYs rates by CRC risk factors in 204 countries and territories in 2021.The darker the green color, the higher the value. CRC, colorectal cancer; DALYs, disability-adjusted life-year.

Among the 204 countries and territories worldwide, diet low in whole grains, milk, and diet high in red meat were also the primary risk factors contributing to CRC-related mortality and DALYs. In a few regions, such as the Kingdom of Cambodia, the People’s Republic of Bangladesh, and the Republic of Sierra Leone, the risk associated with insufficient calcium intake was also noteworthy (**Figure 7**) (**Supplementary Figure S9**).

### Cross-country inequality analysis

**Figure 8:**
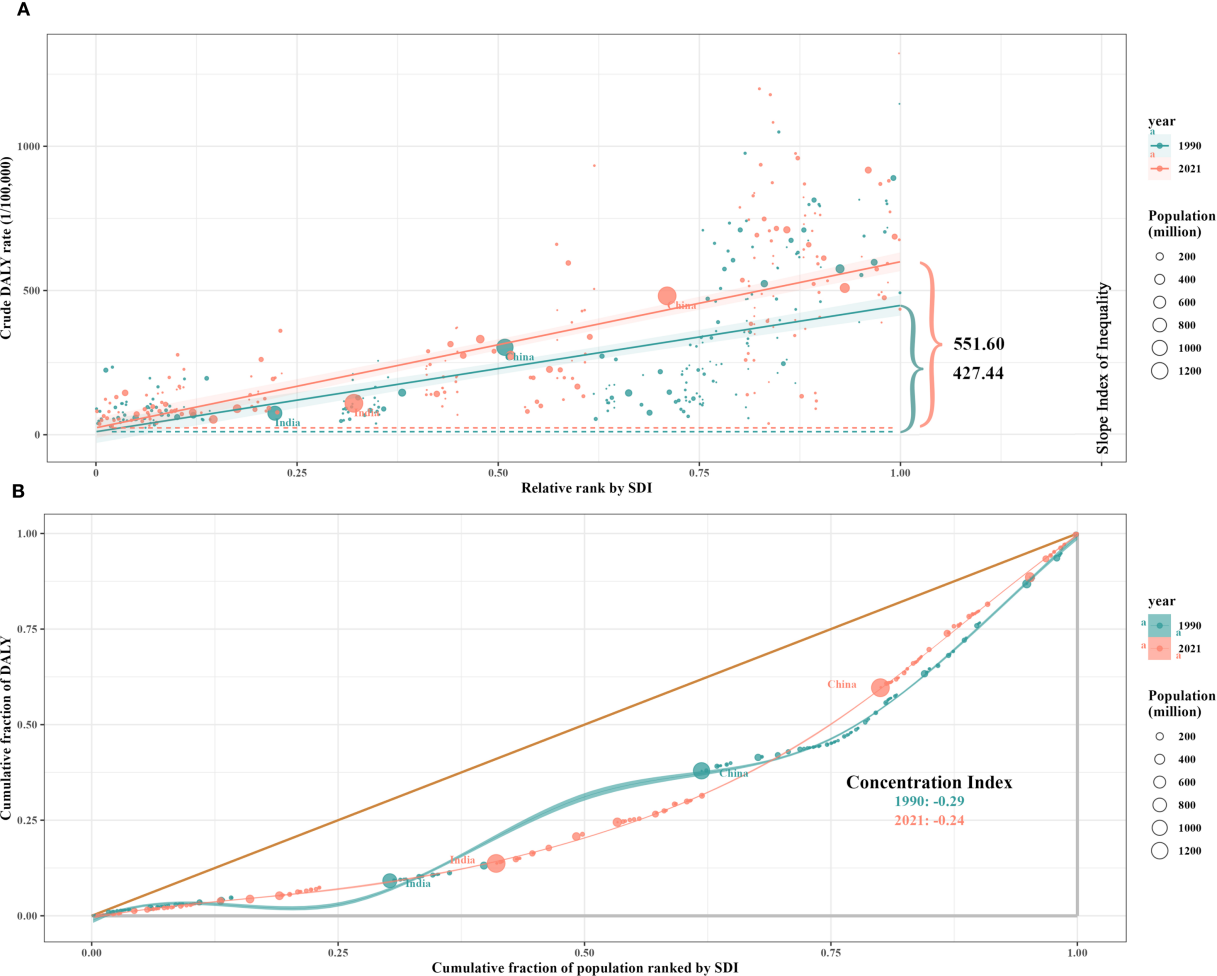
SDI-related health inequality regression **(A)** and concentration **(B)** curves for the DALYs of CRC worldwide, 1990 and 2021. SDI, socio-demographic index; DALYs, disability-adjusted life-years; CRC, colorectal cancer.

Significant absolute and relative SDI-associated inequalities in CRC burden were observed, with remarkable increases in these inequalities over time (**Figure 8**). The DALYs disproportionately concentrated in countries with higher sociodemographic development levels. As shown by the slope index of inequality, the difference in DALYs per 100,000 between the highest and lowest SDI countries exceeded 421.77 (95% CI: 362.98–512.76) in 1990, and increased to 551.60 (95% CI: 510.17–641.22) in 2021. Furthermore, the concentration index, which measures relative gradient inequality, was -0.29 (95% CI: -0.36 to -0.21) in 1990 and -0.24 (95% CI: -0.33 to -0.16) in 2021, indicating a significant imbalance in the distribution of the burden across countries with different SDI levels.

### Frontier analysis

**Figure 9:**
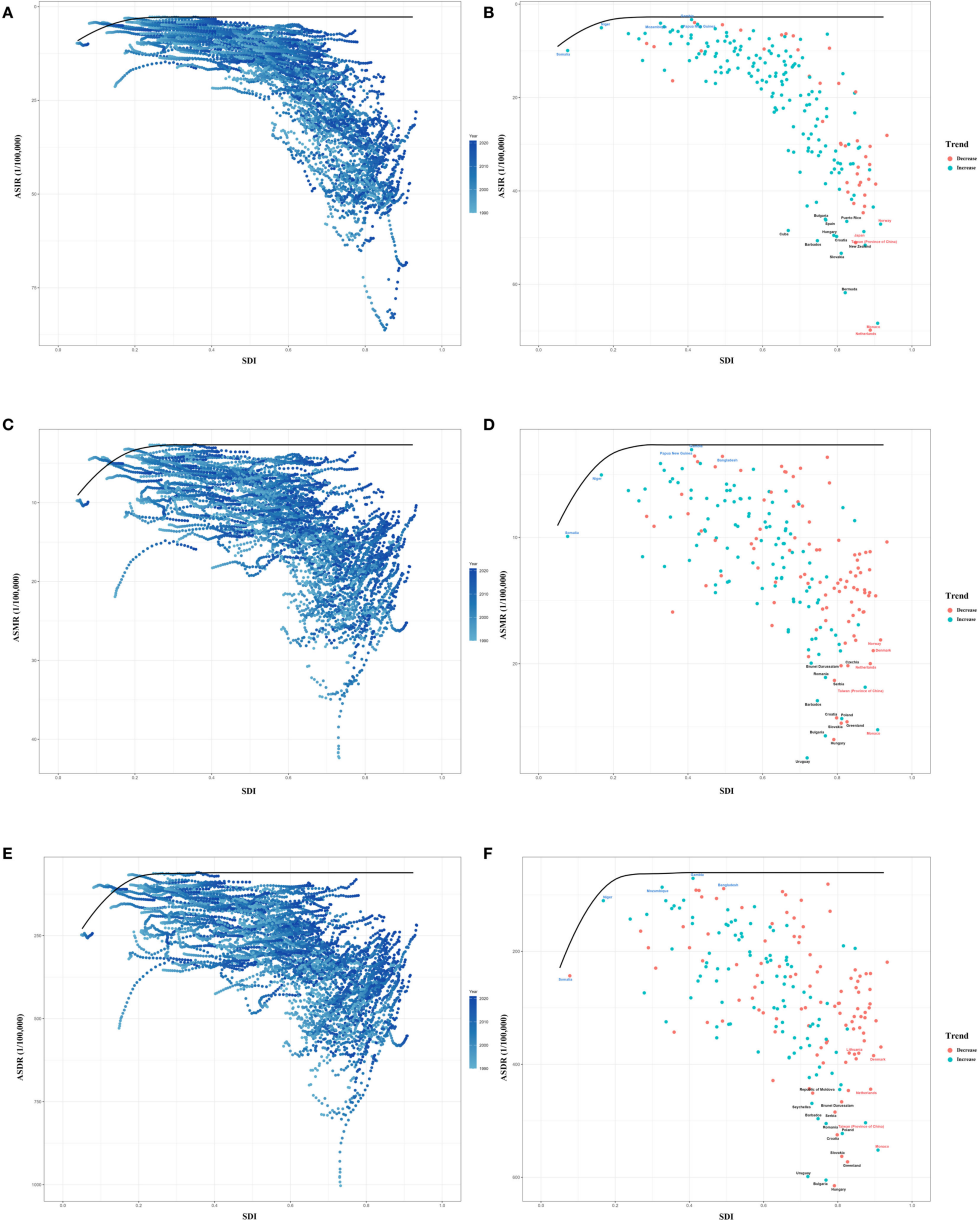
Frontier analysis of ASR from 1990 to 2021. **(A, B)** Frontier analysis of ASIR from 1990 to 2021. **(C, D)** Frontier analysis of ASMR from 1990 to 2021. **(E, F)** Frontier analysis of ASDR from 1990 to 2021. ASIR, age-standardized incidence rate; ASMR, age-standardized mortality rate; ASDR, age-standardized disability-adjusted life-year rate.

We used data on ASIR, ASDR, ASMR and SDI between 1990 and 2021 to calculate the effective difference from the frontier for each country and territory. The results of the frontier analysis are visualized in **Figures 9A, C, E**. The black line represents the lowest achievable ASR value for each country or territory at the current SDI level. The distance between each point and the black line indicates the potential for future reductions in disease burden, with greater distances signifying greater potential. Across the six plots, the scatter points form an inverted triangle, with lower values on the left and higher values on the right. As SDI increases, the range of point distribution also expands, suggesting that countries with higher SDI levels may have greater potential for reducing ASR. **Figures 9B, D, F** identify the 15 worst-performing countries globally, the five best-performing countries with low SDI level, and the five worst-performing countries with high SDI level. Detailed results of the analysis are presented in the **Table 5**, **Supplementary Tables S9, S10** (**Figure 9**) (**Table 5**) (**Supplementary Tables S9, S10**).

**Table 5:** Frontier analysis of ASIR across 204 countries and territories.

Location name	Year	Val	SDI	Frontier	Eff_diff	Trend
15 worst-performing countries and territories in the frontier analysis of ASIR globally						
Netherlands	2021	69.80	0.89	2.75	67.05	Decrease
Monaco	2021	68.33	0.91	2.75	65.58	Increase
Bermuda	2021	61.79	0.82	2.75	59.04	Increase
Slovakia	2021	53.35	0.81	2.75	50.60	Increase
Taiwan (Province of China)	2021	51.62	0.87	2.75	48.87	Increase
New Zealand	2021	51.08	0.85	2.75	48.33	Decrease
Barbados	2021	50.65	0.75	2.75	47.90	Increase
Croatia	2021	49.75	0.80	2.75	47.00	Increase
Hungary	2021	49.51	0.79	2.75	46.76	Increase
Japan	2021	48.70	0.87	2.75	45.96	Increase
Cuba	2021	48.47	0.67	2.75	45.72	Increase
Norway	2021	47.11	0.92	2.75	44.35	Increase
Puerto Rico	2021	46.52	0.83	2.75	43.77	Increase
Spain	2021	46.22	0.77	2.75	43.47	Increase
Bulgaria	2021	46.04	0.77	2.75	43.29	Increase
5 worst-performing countries and territories in the frontier analysis of ASIR with high SDI level						
Netherlands	2021	69.80	0.89	2.75	67.05	Decrease
Monaco	2021	68.33	0.91	2.75	65.58	Increase
Taiwan (Province of China)	2021	51.62	0.87	2.75	48.87	Increase
Japan	2021	48.70	0.87	2.75	45.96	Increase
Norway	2021	47.11	0.92	2.75	44.35	Increase
5 best-performing countries and territories in the frontier analysis of ASIR with low SDI level						
Somalia	2021	9.93	0.08	9.67	0.26	Increase
Gambia	2021	3.31	0.41	2.75	0.56	Increase
Niger	2021	5.06	0.17	4.18	0.88	Increase
Papua New Guinea	2021	3.94	0.42	2.75	1.19	Decrease
Mozambique	2021	4.08	0.33	2.75	1.33	Increase

ASIR, age-standardized incidence rate; SDI, socio-demographic index.

### Projections of CRC incidence rate to 2050

**Figure 10:**
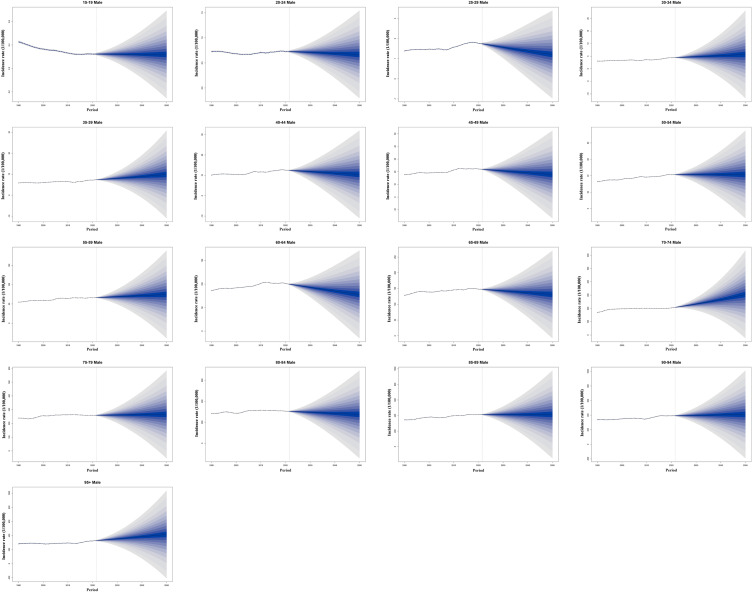
Historical trends and future predictions of global male CRC incidence rates across different age groups from 1990 to 2050. CRC, colorectal cancer.

From 2022 to 2050, the incidence of CRC among individuals aged 15 to 95+ years is projected to increase significantly. By 2050, approximately 3,775/100,000 new CRC cases are expected, with about 2,454/100,000 in males and 1,321/100,000 in females. Compared to the incidence rate of 2,194/100,000 in 2021, the burden of CRC is anticipated to rise substantially, exhibiting a significant upward trend(**Supplementary Tables S11, S12**). For males, the incidence of CRC exhibited a gradually increasing trend across most age groups, with the most pronounced growth observed in the 70–74 age group. It is interesting to discover that a significant decline in incidence was recorded in the 25–29 and 60–64 age groups (**Figure 10**). Among females, the incidence of CRC showed a generally decreasing trend across most age groups, particularly in the 85–89 and 95+ age groups. Notably, an evident increase in CRC incidence was observed only in the 70–74 age group (**Supplementary Figure S10**).

## Discussion

This study reveals significant changes in the global and regional burden of CRC, along with its associated risk factors from 1990 to 2021.CRC is a severe global disease that leads to both mortality and disability. We examined the trends and disparities in disease burden across different regions from 1990 to 2021, conducted a comprehensive analysis of CRC risk factors, and projected future incidence trends across various age subgroups. While previous studies have reported the burden and risk factors of CRC either globally or in specific regions, this study is the first to comprehensively integrate the current burden, associated risk factors, and future projections of CRC at both global and regional levels.

From 1990 to 2021, although the ASIR of CRC showed only a slight increase (EAPC = 0.15), the global number of incident cases surged by 139.39%, primarily driven by the combined effects of population aging and growth (28). The incidence also exhibited significant differences between sexes. The ASIR in males showed a notable increasing trend (EAPC = 0.50), whereas females experienced an overall decline in incidence (EAPC = -0.29). This phenomenon may reflect the combined influence of smoking rate (global average: 32% in males *vs* 7% in females) (29), occupational sedentary behavior (a higher proportion of males engaged in office work), and the protective effects of sex hormones (30). Notably, CRC prevalence was higher in females aged 85 and above than in their male counterparts, potentially due to postmenopausal hormonal changes that weaken the protective effect against CRC (30, 31). This observation aligns with studies suggesting that estrogen replacement therapy may reduce CRC risk (32, 33). Notably, the ASMR declined by 20.31%, which was closely associated with the widespread implementation of global CRC screening programs (in the United States, colonoscopy screening coverage increased from 21% in 2000 to 60%) (34–36), advancements in surgical techniques (such as laparoscopic surgery reducing perioperative mortality) (37, 38), and the application of targeted therapies such as anti-EGFR monoclonal antibodies (39). However, the number of deaths still increased by 83.07%, highlighting the continued rise in the absolute burden of the disease, particularly in regions with limited medical resources.

After comparing the disease burden in different regions, we found that East Asia has become the epicenter of the CRC burden. In 2021, China and Japan accounted for 30% and 7.8% of global CRC incidence cases, respectively (3, 5). Beyond the impact of population size and aging (40), the adverse lifestyle changes and environmental exposures associated with rapid urbanization also warrant attention. In 2018, GBD collaborators reported that processed meat consumption among Chinese residents exceeded the recommended safe intake of World Health Organization by 2.4 times, while whole grain consumption was only 40% of the recommended amount (41). Additionally, insufficient screening coverage has led to a low rate of early CRC diagnosis, with some patients already having lost the optimal window for treatment at the time of diagnosis, further exacerbating the disease burden (3, 42, 43).

Although an overall decline in CRC mortality worldwide was observed, the disease burden remains worsening in some low SDI countries. In regions with a high SDI level (SDI > 0.8), ASMR and ASDR decline as SDI increases, reflecting the positive impact of screening programs (44–46), health literacy (47), and healthcare accessibility (the Healthcare Access and Quality Index [HAQ] generally exceeds 80 in high SDI countries, whereas it typically falls below 50 in low SDI countries) (3). In contrast, in low-middle SDI regions, ASIR rises with increasing SDI, which is closely linked to the westernization of dietary patterns (48, 49), the rising prevalence of obesity (50, 51), and the lack of screening programs. The gap in DALYs between the highest and lowest SDI countries expanded from 427.44/100,000 (95% CI: 362.98/100,000–512.76/100,000) in 1990 to 551.6/100,000 (95% CI: 510.17/100,000–641.22/100,000) in 2021, indicating that health inequality remains severe and is worsening. A concentration index of -0.24 indicates that populations in less developed regions experience a comparatively lower burden of CRC. Notably, the ARIMA model predictions in this study indicate that over the next 15 years, ASIR and ASDR of CRC will continue to rise in low SDI regions while declining in high SDI regions.

Compared to high-income countries, less developed regions often encounter a range of structural barriers in managing the increasing burden of disease. These challenges include limited healthcare resources, inadequate infrastructure, inefficient supply chains, insufficient health education coverage, and poor affordability of nutritious diets (52–55). Collectively, these constraints not only impede the delivery of effective interventions but also contribute to the escalating health burden among local populations. To address rising risk of CRC in countries with low SDI level, global and regional health interventions are necessary to improve screening, diagnosis, and treatment infrastructure in these countries while strengthening public health education to reduce CRC mortality burden and narrow health disparities between countries and regions.

Nowadays, economically developed countries and territories generally recommend routine CRC screening for high-risk age groups (50–75 years) to facilitate early diagnosis and treatment, which is one of the most effective measures for reducing mortality and DALYs (43, 56). Since the 1990s, the United States has actively promoted CRC screening by establishing multidisciplinary expert panels to regularly update and evaluate screening guidelines (57–59). In addition, federal and state-level policies have supported the inclusion of screening services in health insurance coverage, thereby lowering barriers to screening. The Colorectal Cancer Control Program (CRCCP), led by the Centers for Disease Control and Prevention (CDC), has provided screening services for low-income and uninsured populations, effectively increasing screening rates (60). Since 2005, Austria has included fecal occult blood testing (FOBT) in its universal health insurance program and has gradually expanded colonoscopy screening over subsequent years. The establishment of a nationwide electronic health record system has further improved the organization and continuity of these screening programs (61). The experience of Austria and other European countries with established screening programs, such as the Czech Republic and Germany, demonstrates a marked reduction in incidence and mortality (62). These measures all contribute to reducing the disease burden caused by CRC and improving the health level of the public.

To evaluate each country’s performance and potential in reducing the disease burden, we conducted a frontier analysis, which showed that high SDI countries have greater potential for disease burden reduction (with the average distance to the frontier line being 2.3 times that of low SDI countries), which is related to their well-established healthcare systems. However, it is noteworthy that some economically advanced countries perform below the expected level, indicating insufficient policy implementation effectiveness and the need for strengthened quality control.

Although high SDI countries typically have abundant healthcare resources, internal health disparities may limit their potential to effectively control disease burden. For example, in the United States, the life expectancy gap between high-income and low-income groups can reach up to 15 years, and significant inequalities in cancer mortality have been observed across different racial groups (63–65). These stratified disparities lead to the “average performance” of some high SDI countries masking the high risks faced by vulnerable groups. Insufficient policy implementation effectiveness can also result in an increased disease burden. Taking Hungary as an example, its ASMR in 2021 was 26.01/100,000, significantly higher than that of neighboring countries with the same SDI level, such as Austria (11.8/100,000) and the Czech Republic (20.16/100,000). The core issues include a misallocation of healthcare resources and insufficient preventive investments, primary care physicians being far below the European Union average, and the relatively low proportion of government health expenditure in the country (66).

To effectively reduce the disease burden, it is essential to identify its associated risk factors to achieve a reduction in incidence. However, in most cases of CRC, the lack of clear genetic or familial background suggests that postnatal factors such as diet, lifestyle, and environment may play an important role (2, 67). Therefore, further epidemiological research and risk factor analysis are crucial for a deeper understanding of potential mechanisms and the development of effective prevention strategies. Previous studies have demonstrated that an unbalanced diet plays a crucial role in CRC development (68–70). In our study, dietary risks accounted for 65.5% of the CRC-attributable disease burden. Currently, some developed countries have begun adjusting their food policies to guide domestic dietary patterns, aiming to reduce the potential disease burden caused by unhealthy diets (71–74). Although sugar, sodium, and fat have long been the primary focus of dietary policies (75), we found that diet low in whole grains (18.5%), diet low in milk (15.8%), and diet high in red meat (15.2%) are the primary driving factors of CRC-related dietary risks. Notably, the composition of risk factors exhibits significant regional differences. In middle-high and high SDI regions, diet high in processed meat and red meat impose a substantial burden on CRC-related DALYs. Although these risk factors do not account for a predominant proportion of the disease burden among all risks, the absolute DALYs attributed to these factors are significantly higher in middle-high and high SDI regions compared to middle-low and low SDI regions. Studies have shown that the consumption of red meat and processed meat increases the risk of CRC (76). Meanwhile, a reduction in the intake of red meat and processed meat has been associated with a significant decrease in the incidence of CRC, cardiovascular diseases, type 2 diabetes, and all-cause death (77). It is foreseeable that with continued urbanization, industrialization, and economic growth, the CRC-related disease burden linked to high consumption of processed and red meat will continue to increase in less-developed territories.

In high-income regions, metabolic risk factors are also prominent, with high BMI contributing to 11.6% of DALYs. This may be related to the secretion of pro-inflammatory factors such as IL-6 and leptin by adipocytes, which promote the formation of the tumor microenvironment (78–80). Similarly, high fasting blood glucose contributed 8.6% of DALYs, aligning with research findings linking certain metabolic diseases to an increased risk of CRC (81, 82). And in less-developed regions, nutritional deficiencies dominate, with inadequate calcium intake contributing to 23.4% of attributable DALYs by CRC risk factors. Studies have shown that calcium ions can mitigate intestinal mucosal damage by binding to secondary bile acids. Insufficient calcium intake increases the risk of mucosal damage, thereby elevating the risk of CRC (83, 84). A study reported that the average dietary calcium intake in Southeast Asia and some African countries is as low as 200 mg/d, whereas in high-income countries, it ranges from approximately 600 to 800 mg/d (41). For instance, in sub-Saharan Africa, the per capita calcium intake (400 mg/d) is only 40% of the recommended level (1000 mg/d) (85), which corresponds to the region’s high CRC-related DALYs (60.37/100,000) and mortality rate (2.62/100,000) due to calcium deficiency. The disparity between high- and low-income countries can be attributed to various factors, including but not limited to the accessibility of calcium-rich foods and regional dietary habits (86). Insufficient dairy supply is a major risk factor for low dietary calcium intake, particularly in low-income African countries (87). Notably, between 1990 and 2010, the global consumption of unhealthy foods outpaced the growth of healthy foods, significantly increasing the risk of CRC (88). A healthy lifestyle and dietary changes are among the most important strategies for preventing CRC and can effectively reduce the disease burden associated with CRC globally (41).These factors collectively contribute to the high CRC burden due to calcium deficiency in low-SDI regions. Improving dietary structures, increasing the availability of calcium-rich foods, and implementing targeted nutritional interventions for high-risk populations may effectively reduce CRC incidence and mortality in these areas.

As a global public health challenge, accurately predicting the future epidemiological trends of CRC holds significant public health value for formulating effective prevention and control policies, optimizing the allocation of medical resources, and enhancing early screening coverage among high-risk populations. Predictions based on the Bayesian Age-Period-Cohort (BAPC) model indicate that by 2050, the global CRC incidence rate will reach 3,775/100,000, with men aged 70–74 identified as a key high-risk group. Our study found that while CRC incidence rates have shown varying degrees of increase across most age groups, the projected incidence for the 25–29 and 60–64 age groups exhibits a notable downward trend. This decline may be associated with improvements in lifestyle and dietary patterns among younger populations. Tobacco and alcohol are well-established high-risk factors for cancer (16, 89), and the global decline in adolescent smoking rates and excessive alcohol consumption has likely contributed to a reduction in cancer incidence risk. Research suggests that inappropriate antibiotic use increases the risk of colorectal adenocarcinoma, particularly rectal adenocarcinoma (90). With the increasing regulation of antibiotic use across different countries and regions, the CRC risk associated with antibiotic exposure is expected to decrease accordingly. Although genetic susceptibility is associated with the most significant increases in cancer risk, the majority of CRC cases are sporadic rather than familial (2, 67). This underscores the need to focus not only on genetic factors but also on the contribution of other risk factors to CRC development. For the 60–64 age group, the observed downward trend in future CRC incidence may be linked to the expansion of CRC screening programs and the preventive effects of certain medications (91, 92). Nevertheless, it is undeniable that, from an overall perspective, the burden of CRC will continue to rise in the future, making the establishment of a stratified prevention and control system increasingly urgent.

In terms of CRC screening, although colonoscopy remains the most direct and effective method, its reliance on skilled endoscopists and robust infrastructure often makes it unfeasible in many transitioning countries. Moreover, the detection of early-stage CRC still heavily depends on the endoscopist’s experience, which can lead to missed diagnoses. Recent advances in artificial intelligence offer promising solutions to these challenges. Deep learning algorithms have demonstrated excellent performance in the classification and automated diagnosis of histopathological images, contributing to improved accuracy and efficiency in CRC detection. These technologies may also support clinical decision-making by assisting in the triage of positive screening results and the formulation of personalized treatment strategies (93–97). Apart from colonoscopy, an increasing body of evidence suggests that non-invasive procedures, such as fecal immunochemical testing, offer high specificity, good sensitivity, and ease of operation, making them potentially more cost-effective for CRC screening in many transitional regions (98–100). Moreover, for the vast majority of residents, non-invasive screening methods such as fecal immunochemical testing are more acceptable, allowing for broader coverage of high-risk populations for CRC and effectively improving its screening rates.

We call on capable countries to increase medical assistance to low-income countries to collectively alleviate the global burden of CRC. Meanwhile, middle- and low-income countries can adopt cost-effective strategies, such as enhancing public health awareness through health education, promoting healthy dietary habits to reduce risk factors, and implementing affordable screening methods (such as fecal occult blood testing in high-risk areas) to improve early diagnosis rates. Tailoring intervention measures to the developmental level of each country is crucial for advancing global CRC prevention and effectively reducing its disease burden (**Table 6**).

**Table 6:** Colorectal cancer control strategies by SDI level.

SDI level	Public health recommendations
High SDI	Promote high-precision screening tools (e.g., colonoscopy + AI assistance)
	Optimize screening coverage and quality
	Encourage personalized prevention strategies based on individual risk factors
High-middle SDI	Expand screening coverage
	Improve screening compliance and follow-up rates
	Introduce cost-effective early detection technologies
Middle SDI	Develop national screening guidelines
	Strengthen healthcare workforce and infrastructure
	Implement pilot screening programs
Low-middle SDI	Increase public awareness and education
	Explore low-cost screening methods (e.g., fecal immunochemical test)
	Enhance access to basic medical services
Low SDI	Focus on primary prevention (e.g., healthy diet, anti-smoking campaigns)
	Strengthen health education and community mobilization
	Seek international support for technical and financial aid

SDI, socio-demographic index.

This study has several limitations. First, cancer registration systems remain incomplete in some countries, particularly in low- and middle-income regions, which may lead to discrepancies between the GBD 2021 estimates and the actual disease burden (101). Second, some emerging risk factors were not included in our analysis, such as the alterations in intestinal microbial composition (102, 103) and various environmental exposures (104). Additionally, the forecasting models did not fully account for the potential effects of the COVID-19 pandemic on cancer screening and healthcare services (105), which may have introduced some degree of bias in the estimation of CRC burden. Future research should focus on establishing multi-center prospective cohorts, promoting international health data sharing, and integrating multi-omics data, including genomics, metabolomics, and microbiomics, to develop more dynamic and accurate risk prediction models. Such efforts will provide stronger scientific evidence for the precise prevention and control of colorectal cancer.

## Conclusion

This study provides essential insights into CRC, offering valuable implications for global public health. In recent decades, the burden of CRC has risen significantly, presenting a substantial challenge to global public health. To reduce its societal impact, public health policies should be tailored to the geographical distribution, epidemiological trends, and key risk factors associated with CRC. Furthermore, optimizing resource allocation and enhancing preventive measures and early screening strategies are essential.

## Data Availability

The original contributions presented in the study are included in the article/**Supplementary Material**, further inquiries can be directed to the corresponding author/s.
