# Supplementary material for: Global, regional, national burden of colorectal cancer from 1990 to 2021, with projections of incidence to 2050: a systematic analysis of the global burden of disease study 2021
Source: Front Oncol. 2025 Oct 6;15:1597847. doi: 10.3389/fonc.2025.1597847 (PMC12535885; doi:10.3389/fonc.2025.1597847)
Supplement: Supplementary file 1 [file Supplementaryfile1.pdf]

Table.S1 ASIR, ASMR, and ASDR for colorectal cancer across 21 regions in 2021

Location	ASIR/100,000 persons (95%UI)			ASMR/100,000 persons (95%UI)			ASDR/100,000 persons (95%UI)		
	Both	Male	Female	Both	Male	Female	Both	Male	Female
Andean Latin America	14.39(11.39-17.89)	14.34(11.33-17.86)	14.4(11.17-18.11)	10(7.98-12.18)	9.65(7.58-11.78)	10.3(7.97-12.87)	226.76(180.59-279.8)	222.65(175.17-276.65)	230.1(178.23-291.03)
Australasia	43.97(38.91-49.55)	51.92(46.76-58.05)	36.75(31.68-42.55)	14.62(12.77-16.52)	17.51(15.55-19.48)	12.07(10.21-14)	327.19(290.9-364.68)	391.62(352.65-434.01)	268.36(231.99-307.52)
Caribbean	34.33(29.87-38.86)	35.83(30.83-41.02)	33(28.48-37.63)	14.57(12.72-16.6)	14.91(12.89-16.99)	14.23(12.24-16.12)	335.02(290.41-383.11)	346.61(298.47-397.31)	324.23(278.88-372.63)
Central Asia	10.82(9.69-11.9)	13.12(11.83-14.45)	9.13(8.1-10.06)	7.87(7.06-8.65)	9.65(8.71-10.6)	6.61(5.89-7.29)	196.55(175.59-216.68)	238.03(213.87-262.52)	164.4(146.06-182.72)
Central Europe	38.82(35.71-41.96)	53.39(49.07-57.47)	27.77(25.26-30.29)	22.58(20.81-24.28)	31.54(29.02-33.73)	16.19(14.62-17.55)	506.48(467.97-544.57)	696.98(640.42-746.97)	356.47(325.96-386.41)
Central Latin America	17.74(15.75-19.81)	23.61(20.73-26.53)	12.75(11.11-14.42)	9.3(8.26-10.35)	10.16(8.96-11.39)	8.56(7.47-9.62)	231.95(206.64-258.57)	258.1(227.87-290.6)	209.09(182.44-237.73)
Central Sub-Saharan Africa	7.87(6.09-10.53)	9.31(7.21-13.14)	6.82(4.92-9.3)	7.45(5.77-10.14)	8.88(6.86-12.82)	6.44(4.6-9.05)	177.79(135.17-238.59)	208(158.93-296.88)	154.25(110.67-212.26)
East Asia	31.6(25.9-37.85)	42.43(32.99-54.03)	22.02(16.9-27.9)	13.78(11.33-16.35)	19.11(15.02-24.35)	9.48(7.31-11.97)	334.5(274.01-399.93)	455.92(356.04-583.37)	222.78(172.6-282.97)
Eastern Europe	32.11(29.59-34.73)	39.32(35.01-43.21)	27.89(24.99-30.86)	18.05(16.58-19.56)	23.44(20.89-25.75)	15.03(13.45-16.67)	424.54(389.69-463.67)	541.35(480.46-599.53)	351.34(313.61-392.62)
Eastern Sub-Saharan Africa	11.23(9.84-12.77)	14.16(11.94-16.51)	8.81(7.56-10.27)	10.76(9.41-12.15)	13.75(11.62-15.93)	8.34(7.17-9.71)	242.13(211.3-279.08)	300.45(251.88-360.08)	191.83(163.09-225.17)
High-income Asia Pacific	44.89(40.2-47.85)	59.28(54.93-62.75)	32.4(27.41-35.44)	14.99(13.03-16.09)	19.19(17.53-20.27)	11.46(9.26-12.78)	331.48(303.85-352.47)	424.87(396.43-449.67)	248.46(215.58-270.22)
High-income North America	38.75(36.13-40.48)	45.82(43.2-47.8)	32.45(29.63-34.24)	12.95(11.89-13.65)	15.39(14.4-16.09)	10.86(9.69-11.56)	316.04(298.52-330.84)	379.92(362.41-395.86)	258.37(240.6-272.39)
North Africa and Middle East	14.43(12.67-16.35)	14.96(12.84-17.22)	13.97(12.09-15.89)	8.95(7.85-10.08)	9.89(8.44-11.35)	8.01(6.9-9.11)	209.04(183.16-237.28)	230.39(195.7-264.81)	187.57(161.62-215.53)
Oceania	6.44(5.55-7.42)	6.13(5.16-7.01)	6.82(5.73-8.06)	5.58(4.8-6.42)	5.4(4.56-6.19)	5.82(4.9-6.9)	136.65(116.1-159.45)	128.39(107.96-148.24)	146.03(120.87-173.79)
South Asia	5.65(5.08-6.3)	5.99(5.11-6.88)	5.35(4.58-6.19)	4.63(4.17-5.16)	4.97(4.25-5.72)	4.33(3.71-5.02)	120.37(108.3-135.3)	124.54(105.99-144.95)	116.92(100.46-136.41)
Southeast Asia	17.7(15.41-19.89)	21.97(18.27-25.56)	14.06(12.02-16.21)	12.74(11.12-14.26)	15.87(13.42-18.39)	10.18(8.72-11.71)	313.37(270.75-353.54)	389.31(323.46-454.02)	247.02(212.19-288.06)
Southern Latin America	28.32(25.1-31.53)	34.55(30.5-38.95)	23.41(20.5-26.38)	18.13(16.12-20.25)	22.32(19.75-25.08)	14.91(12.93-16.73)	407.99(363.05-457.56)	499.48(441.2-564.84)	333.66(294.63-376)
Southern Sub-Saharan Africa	13.45(12.16-14.83)	16.23(14.07-18.55)	11.54(10.24-12.92)	11.47(10.37-12.61)	13.82(12.04-15.68)	9.9(8.78-11.08)	270.64(244.94-301.85)	326.03(285.03-375.59)	230.43(203.81-259.4)
Tropical Latin America	17.17(15.82-18.31)	19.49(18.04-20.86)	15.3(13.81-16.41)	11.58(10.59-12.34)	12.92(11.88-13.81)	10.52(9.4-11.31)	286.22(266.67-302.45)	316.3(295.95-336.6)	261.56(239.85-279.12)
Western Europe	40.54(37.17-43.07)	50.48(47.12-53.45)	32.12(28.65-34.4)	15.11(13.53-16.25)	19.22(17.72-20.47)	11.83(10.13-12.9)	326.82(301.67-347.23)	411.22(384.6-435.23)	254.09(228.11-272.91)
Western Sub-Saharan Africa	6.29(5.36-7.3)	6.88(5.74-8.19)	5.75(4.7-6.91)	5.98(5.12-6.88)	6.55(5.49-7.78)	5.48(4.54-6.53)	132.05(110.39-155.48)	145.15(119.96-175.81)	120.37(95.97-147.09)

ASIR age-standardized incidence rate, ASMR age-standardized mortality rate, ASDR age-standardized disability-adjusted life-year rate, UI uncertainty interval

Table.S2 EAPC of ASIR, ASMR, and ASDR for colorectal cancer in 21 regions from 1990 to 2021

Location	EAPC of ASIR (95%CI)			EAPC of ASMR (95%CI)			EAPC of ASDR (95%CI)		
	Both	Male	Female	Both	Male	Female	Both	Male	Female
Andean Latin America	1.43(1.32 to 1.53)	1.74(1.62 to 1.85)	1.18(1.04 to 1.31)	0.46(0.35 to 0.57)	0.74(0.63 to 0.85)	0.25(0.11 to 0.40)	0.39(0.27 to 0.50)	0.63(0.52 to 0.75)	0.18(0.04 to 0.32)
Australasia	-0.58(-0.71 to -0.44)	-0.59(-0.73 to -0.45)	-0.62(-0.76 to -0.49)	-1.92(-2.01 to -1.82)	-1.94(-2.05 to -1.83)	-1.97(-2.05 to -1.89)	-2.06(-2.17 to -1.96)	-2.05(-2.17 to -1.93)	-2.13(-2.22 to -2.03)
Caribbean	1.26(1.19 to 1.34)	1.46(1.37 to 1.55)	1.08(1.00 to 1.16)	0.27(0.23 to 0.31)	0.45(0.39 to 0.51)	0.11(0.07 to 0.15)	0.33(0.28 to 0.37)	0.51(0.44 to 0.57)	0.16(0.11 to 0.20)
Central Asia	-0.15(-0.36 to 0.06)	0.01(-0.25 to 0.27)	-0.38(-0.55 to -0.22)	-0.50(-0.65 to -0.34)	-0.34(-0.54 to -0.15)	-0.72(-0.84 to -0.60)	-0.88(-1.00 to -0.76)	-0.70(-0.88 to -0.52)	-1.12(-1.20 to -1.04)
Central Europe	0.98(0.81 to 1.15)	1.33(1.15 to 1.52)	0.49(0.32 to 0.65)	-0.03(-0.15 to 0.10)	0.40(0.27 to 0.54)	-0.56(-0.69 to -0.43)	-0.12(-0.25 to 0.00)	0.24(0.10 to 0.39)	-0.64(-0.76 to -0.52)
Central Latin America	2.05(1.99 to 2.11)	2.67(2.59 to 2.75)	1.29(1.23 to 1.35)	0.86(0.78 to 0.94)	1.38(1.29 to 1.46)	0.39(0.31 to 0.48)	1.11(1.03 to 1.18)	1.56(1.49 to 1.63)	0.67(0.59 to 0.76)
Central Sub-Saharan Africa	0.24(0.06 to 0.41)	0.33(0.10 to 0.56)	0.22(0.11 to 0.34)	0.06(-0.09 to 0.21)	0.17(-0.02 to 0.37)	0.04(-0.05 to 0.13)	0.02(-0.12 to 0.17)	0.08(-0.12 to 0.28)	0.01(-0.07 to 0.10)
East Asia	1.75(1.65 to 1.84)	2.30(2.19 to 2.41)	0.88(0.78 to 0.98)	-0.44(-0.50 to -0.39)	0.13(0.07 to 0.19)	-1.30(-1.41 to -1.19)	-0.59(-0.66 to -0.51)	0.03(-0.03 to 0.10)	-1.56(-1.69 to -1.44)
Eastern Europe	0.62(0.51 to 0.74)	0.72(0.60 to 0.84)	0.43(0.31 to 0.55)	-0.19(-0.31 to -0.07)	-0.12(-0.26 to 0.02)	-0.40(-0.50 to -0.30)	-0.48(-0.61 to -0.35)	-0.31(-0.46 to -0.16)	-0.74(-0.87 to -0.62)
Eastern Sub-Saharan Africa	-0.10(-0.22 to 0.01)	0.12(0.01 to 0.23)	-0.26(-0.38 to -0.14)	-0.21(-0.31 to -0.12)	0.04(-0.05 to 0.13)	-0.38(-0.48 to -0.28)	-0.58(-0.68 to -0.47)	-0.41(-0.51 to -0.31)	-0.69(-0.80 to -0.59)
High-income Asia Pacific	0.33(0.23 to 0.44)	0.33(0.21 to 0.46)	0.14(0.05 to 0.22)	-0.73(-0.78 to -0.69)	-0.77(-0.83 to -0.71)	-0.89(-0.95 to -0.84)	-0.93(-0.98 to -0.88)	-0.89(-0.95 to -0.82)	-1.14(-1.19 to -1.10)
High-income North America	-0.80(-0.93 to -0.67)	-0.87(-0.99 to -0.76)	-0.82(-0.97 to -0.68)	-1.63(-1.69 to -1.57)	-1.73(-1.79 to -1.67)	-1.65(-1.72 to -1.58)	-1.36(-1.42 to -1.31)	-1.40(-1.45 to -1.34)	-1.43(-1.49 to -1.37)
North Africa and Middle East	1.31(1.15 to 1.48)	1.34(1.19 to 1.49)	1.29(1.12 to 1.47)	0.21(0.06 to 0.36)	0.32(0.20 to 0.45)	0.07(-0.11 to 0.25)	-0.01(-0.15 to 0.12)	0.15(0.03 to 0.27)	-0.21(-0.35 to -0.06)
Oceania	-0.18(-0.27 to -0.09)	-0.20(-0.31 to -0.09)	-0.15(-0.23 to -0.08)	-0.37(-0.47 to -0.26)	-0.38(-0.51 to -0.25)	-0.35(-0.44 to -0.26)	-0.39(-0.48 to -0.29)	-0.41(-0.52 to -0.29)	-0.36(-0.44 to -0.28)
South Asia	0.46(0.33 to 0.60)	0.66(0.55 to 0.77)	0.27(0.10 to 0.44)	0.02(-0.09 to 0.13)	0.22(0.12 to 0.32)	-0.16(-0.30 to -0.03)	-0.10(-0.21 to 0.01)	0.10(0.01 to 0.20)	-0.30(-0.44 to -0.15)
Southeast Asia	1.45(1.40 to 1.50)	1.89(1.84 to 1.93)	0.94(0.86 to 1.01)	0.74(0.67 to 0.80)	1.15(1.10 to 1.21)	0.27(0.18 to 0.36)	0.61(0.55 to 0.67)	1.07(1.02 to 1.12)	0.06(-0.02 to 0.15)
Southern Latin America	0.73(0.56 to 0.90)	0.84(0.64 to 1.04)	0.61(0.46 to 0.77)	-0.10(-0.27 to 0.07)	0.07(-0.13 to 0.26)	-0.25(-0.41 to -0.10)	-0.04(-0.19 to 0.11)	0.05(-0.13 to 0.22)	-0.15(-0.27 to -0.03)
Southern Sub-Saharan Africa	1.29(1.06 to 1.52)	1.29(1.00 to 1.58)	1.37(1.17 to 1.58)	0.97(0.70 to 1.25)	1.01(0.69 to 1.34)	1.02(0.78 to 1.27)	1.06(0.78 to 1.35)	0.98(0.62 to 1.33)	1.24(0.97 to 1.51)
Tropical Latin America	1.42(1.31 to 1.54)	1.82(1.70 to 1.94)	1.07(0.94 to 1.19)	0.66(0.57 to 0.75)	1.06(0.96 to 1.15)	0.32(0.22 to 0.43)	0.77(0.67 to 0.87)	1.10(0.99 to 1.20)	0.48(0.37 to 0.58)
Western Europe	-0.11(-0.26 to 0.04)	-0.03(-0.21 to 0.15)	-0.35(-0.46 to -0.23)	-1.40(-1.45 to -1.36)	-1.30(-1.36 to -1.23)	-1.67(-1.71 to -1.62)	-1.41(-1.46 to -1.36)	-1.32(-1.40 to -1.25)	-1.64(-1.69 to -1.60)
Western Sub-Saharan Africa	0.78(0.71 to 0.85)	0.79(0.70 to 0.88)	0.79(0.73 to 0.85)	0.62(0.55 to 0.69)	0.61(0.52 to 0.69)	0.65(0.59 to 0.71)	0.46(0.40 to 0.52)	0.51(0.43 to 0.59)	0.44(0.39 to 0.49)

ASIR age-standardized incidence rate, ASMR age-standardized mortality rate, ASDR age-standardized disability-adjusted life-year rate, EAPC estimate annual percentage change, CI confidence interval

Table.S3 Regional incidence, deaths, and DALYs of colorectal cancer across 204 countries in 2021

Location	Incidence/1000 (95%UI)			Deaths/1000 (95%UI)			DALYs/1000 (95%UI)		
	Both	Male	Female	Both	Male	Female	Both	Male	Female
Afghanistan	1.55 (0.67-2.45)	0.42 (0.24-0.7)	1.13 (0.38-1.88)	1.22 (0.56-1.91)	0.36 (0.21-0.61)	0.86 (0.31-1.39)	40.78 (17.12-64.99)	11.66 (6.76-19.69)	29.12 (9.25-48.6)
Albania	0.5 (0.39-0.67)	0.29 (0.21-0.38)	0.21 (0.14-0.32)	0.33 (0.25-0.43)	0.18 (0.14-0.24)	0.14 (0.09-0.2)	6.7 (5.15-8.97)	3.84 (2.82-5.19)	2.86 (1.88-4.18)
Algeria	2.36 (1.82-3.03)	1.21 (0.94-1.56)	1.14 (0.81-1.62)	1.43 (1.13-1.8)	0.82 (0.63-1.05)	0.62 (0.44-0.88)	35.66 (27.59-45.7)	19.86 (15.35-25.58)	15.8 (11.26-22.27)
American Samoa	0.01 (0.01-0.01)	0 (0-0.01)	0 (0-0.01)	0.01 (0.01-0.01)	0 (0-0)	0 (0-0)	0.18 (0.15-0.22)	0.1 (0.08-0.12)	0.08 (0.06-0.1)
Andorra	0.07 (0.05-0.09)	0.05 (0.03-0.07)	0.02 (0.01-0.02)	0.03 (0.02-0.03)	0.02 (0.01-0.03)	0.01 (0-0.01)	0.53 (0.37-0.71)	0.4 (0.28-0.56)	0.13 (0.09-0.18)
Angola	1.04 (0.76-1.38)	0.58 (0.41-0.78)	0.46 (0.3-0.68)	0.9 (0.66-1.2)	0.51 (0.36-0.68)	0.4 (0.26-0.6)	27.22 (19.42-36.79)	15.37 (10.54-20.9)	11.85 (7.57-17.97)
Antigua and Barbuda	0.03 (0.03-0.04)	0.01 (0.01-0.02)	0.02 (0.02-0.02)	0.01 (0.01-0.02)	0.01 (0.01-0.01)	0.01 (0.01-0.01)	0.34 (0.31-0.37)	0.15 (0.13-0.16)	0.2 (0.18-0.21)
Argentina	16.15 (14.16-18.14)	8.8 (7.66-10.08)	7.34 (6.33-8.35)	11.07 (9.75-12.45)	5.9 (5.12-6.74)	5.17 (4.43-5.87)	243.77 (213.62-274.73)	135.95 (118.23-156.08)	107.83 (93.81-123.76)
Armenia	0.85 (0.74-0.97)	0.42 (0.37-0.48)	0.43 (0.37-0.49)	0.57 (0.5-0.65)	0.27 (0.24-0.32)	0.3 (0.26-0.34)	12.94 (11.31-14.86)	6.63 (5.83-7.64)	6.31 (5.46-7.27)
Australia	18.94 (16.59-21.68)	10.51 (9.35-11.88)	8.43 (7.07-9.92)	6.67 (5.73-7.64)	3.62 (3.18-4.1)	3.05 (2.5-3.62)	134.82 (118.21-153.42)	76.35 (68.08-85.39)	58.47 (49.36-68.33)
Austria	5.24 (4.58-6.01)	3 (2.63-3.39)	2.24 (1.88-2.63)	2.35 (2.01-2.7)	1.29 (1.14-1.46)	1.06 (0.86-1.25)	44.31 (39-50.54)	25.82 (22.96-29.1)	18.49 (15.8-21.51)
Azerbaijan	1.03 (0.78-1.31)	0.57 (0.41-0.74)	0.46 (0.34-0.61)	0.71 (0.54-0.9)	0.39 (0.28-0.51)	0.32 (0.24-0.42)	20.52 (15.35-26.25)	11.52 (8.21-15.2)	8.99 (6.6-11.83)
Bahamas	0.15 (0.12-0.18)	0.08 (0.06-0.1)	0.07 (0.05-0.08)	0.07 (0.06-0.09)	0.04 (0.03-0.05)	0.03 (0.03-0.04)	1.86 (1.49-2.33)	1.05 (0.82-1.34)	0.81 (0.64-1)
Bahrain	0.16 (0.13-0.22)	0.1 (0.07-0.14)	0.07 (0.05-0.09)	0.07 (0.06-0.1)	0.05 (0.04-0.07)	0.03 (0.02-0.04)	2.21 (1.74-3.08)	1.45 (1.12-2.11)	0.76 (0.57-1.03)
Bangladesh	6.09 (4.49-8.43)	3.35 (2.39-4.82)	2.75 (1.91-3.99)	4.75 (3.54-6.61)	2.67 (1.93-3.82)	2.08 (1.45-3.08)	128.05 (92.94-180.92)	68.61 (48.66-102.05)	59.44 (41.74-89.02)
Barbados	0.26 (0.21-0.32)	0.13 (0.1-0.16)	0.13 (0.1-0.16)	0.12 (0.09-0.15)	0.06 (0.04-0.07)	0.06 (0.05-0.08)	2.51 (1.95-3.1)	1.25 (0.94-1.58)	1.25 (0.99-1.55)
Belarus	5.81 (4.4-7.41)	2.88 (2.18-3.64)	2.93 (2.21-3.74)	2.81 (2.15-3.53)	1.38 (1.06-1.71)	1.43 (1.09-1.83)	65.92 (49.88-84.09)	34.15 (26-42.84)	31.77 (24.01-40.67)
Belgium	8.47 (7.38-9.57)	4.65 (4.01-5.24)	3.82 (3.19-4.46)	3.68 (3.1-4.19)	1.92 (1.67-2.17)	1.75 (1.42-2.04)	67.95 (59.2-76.5)	37.86 (32.97-42.98)	30.09 (25.55-34.54)
Belize	0.05 (0.04-0.06)	0.03 (0.02-0.03)	0.02 (0.02-0.03)	0.03 (0.02-0.03)	0.01 (0.01-0.02)	0.01 (0.01-0.01)	0.7 (0.6-0.81)	0.36 (0.3-0.43)	0.34 (0.29-0.39)
Benin	0.28 (0.21-0.38)	0.15 (0.11-0.2)	0.13 (0.1-0.18)	0.26 (0.2-0.34)	0.13 (0.1-0.18)	0.12 (0.09-0.17)	6.75 (4.93-9.04)	3.62 (2.67-4.85)	3.13 (2.14-4.46)
Bermuda	0.08 (0.07-0.1)	0.05 (0.04-0.06)	0.04 (0.03-0.05)	0.03 (0.02-0.03)	0.01 (0.01-0.02)	0.01 (0.01-0.02)	0.52 (0.44-0.64)	0.28 (0.23-0.35)	0.24 (0.19-0.31)
Bhutan	0.03 (0.02-0.04)	0.02 (0.01-0.02)	0.02 (0.01-0.02)	0.03 (0.02-0.03)	0.01 (0.01-0.02)	0.01 (0.01-0.02)	0.68 (0.49-0.89)	0.36 (0.21-0.49)	0.32 (0.23-0.44)
Bolivia (Plurinational State of Bolivia)	1.43 (0.98-2)	0.6 (0.42-0.82)	0.84 (0.48-1.26)	1.18 (0.8-1.63)	0.48 (0.33-0.66)	0.7 (0.4-1.05)	28.73 (19.71-40.35)	12.01 (8.24-16.37)	16.73 (9.83-25.34)
Bosnia and Herzegovina	1.93 (1.49-2.42)	1.15 (0.85-1.52)	0.78 (0.59-1)	1.21 (0.95-1.49)	0.7 (0.52-0.91)	0.51 (0.39-0.65)	26.03 (19.97-32.6)	15.68 (11.4-20.94)	10.35 (7.96-12.97)
Botswana	0.15 (0.11-0.21)	0.08 (0.06-0.12)	0.07 (0.05-0.11)	0.13 (0.1-0.18)	0.07 (0.05-0.1)	0.06 (0.04-0.09)	3.55 (2.54-5.18)	2 (1.35-2.85)	1.54 (1-2.47)
Brazil	43.39 (40.05-46.28)	22.14 (20.54-23.63)	21.26 (19.16-22.83)	28.82 (26.43-30.7)	14.18 (13.13-15.16)	14.64 (13.06-15.76)	730.35 (681.57-772.15)	368.5 (344.81-391.73)	361.85 (331.91-386.82)
Brunei Darussalam	0.1 (0.09-0.12)	0.06 (0.05-0.07)	0.04 (0.03-0.06)	0.06 (0.05-0.07)	0.04 (0.03-0.04)	0.03 (0.02-0.04)	1.79 (1.51-2.11)	1.04 (0.83-1.25)	0.76 (0.59-0.97)
Bulgaria	6.49 (5.33-7.84)	3.81 (3.12-4.67)	2.67 (2.16-3.2)	3.74 (3.05-4.47)	2.17 (1.78-2.6)	1.57 (1.27-1.88)	81.4 (66.58-97.73)	48.85 (39.99-59.03)	32.55 (26.52-38.84)

Burkina Faso	0.54 (0.4-0.7)	0.28 (0.2-0.38)	0.26 (0.19-0.34)	0.5 (0.37-0.65)	0.26 (0.18-0.35)	0.25 (0.17-0.32)	13.03 (9.46-17)	6.75 (4.72-9.48)	6.28 (4.28-8.44)
Burundi	0.4 (0.28-0.56)	0.23 (0.16-0.35)	0.17 (0.12-0.23)	0.36 (0.26-0.52)	0.21 (0.15-0.32)	0.15 (0.11-0.21)	10.58 (7.42-15.18)	6.12 (4.19-9.68)	4.46 (3.11-6.31)
Cabo Verde	0.05 (0.04-0.06)	0.02 (0.02-0.03)	0.02 (0.02-0.03)	0.04 (0.03-0.05)	0.02 (0.01-0.02)	0.02 (0.01-0.02)	0.8 (0.62-0.96)	0.45 (0.33-0.56)	0.35 (0.27-0.43)
Cambodia	2.11 (1.57-2.76)	1.09 (0.79-1.46)	1.03 (0.71-1.41)	1.67 (1.25-2.18)	0.86 (0.62-1.16)	0.82 (0.57-1.11)	47.24 (34.95-61.84)	25.01 (17.72-33.66)	22.22 (15.32-30.54)
Cameroon	0.94 (0.65-1.28)	0.52 (0.35-0.74)	0.41 (0.29-0.59)	0.82 (0.57-1.11)	0.46 (0.31-0.64)	0.37 (0.26-0.52)	22.78 (15.31-31.64)	12.83 (8.33-18.09)	9.94 (6.72-14.35)
Canada	30.54 (26.97-34.05)	17.12 (15.34-18.9)	13.41 (11.51-15.37)	10.76 (9.39-12.1)	5.66 (5.09-6.23)	5.1 (4.3-5.91)	215.09 (191.66-238.15)	121.05 (110.07-133.36)	94.04 (82.07-106.66)
Central African Republic	0.2 (0.13-0.29)	0.11 (0.07-0.17)	0.09 (0.05-0.15)	0.19 (0.13-0.27)	0.1 (0.06-0.15)	0.09 (0.05-0.14)	6.02 (3.93-8.84)	3.33 (1.99-5.14)	2.69 (1.55-4.6)
Chad	0.35 (0.26-0.45)	0.19 (0.14-0.25)	0.15 (0.11-0.21)	0.32 (0.24-0.42)	0.18 (0.13-0.24)	0.14 (0.1-0.2)	8.9 (6.55-11.68)	4.87 (3.51-6.43)	4.03 (2.78-5.59)
Chile	6.18 (5.4-7)	3.15 (2.77-3.57)	3.03 (2.62-3.5)	3.41 (2.99-3.84)	1.69 (1.49-1.89)	1.72 (1.45-1.97)	74.02 (65.14-83.18)	38.57 (34.17-43.43)	35.45 (30.94-40.5)
China	658.32 (532-798.06)	419.01 (319.83-541.67)	239.31 (181.75-305.84)	275.13 (223.38-330.96)	174.4 (133.84-226.28)	100.73 (76.6-128.09)	6848.39 (5513.41-8284.23)	4488.27 (3427.06-5852.47)	2360.12 (1798.15-3027.36)
Colombia	12.24 (10.08-14.97)	7.19 (5.81-8.93)	5.05 (4.16-6.02)	5.78 (4.77-7)	2.67 (2.15-3.3)	3.11 (2.56-3.71)	142.01 (116.97-171.04)	67.82 (54.3-83.65)	74.19 (61.66-88.25)
Comoros	0.05 (0.04-0.07)	0.03 (0.02-0.04)	0.02 (0.02-0.03)	0.04 (0.03-0.06)	0.02 (0.01-0.03)	0.02 (0.01-0.03)	1.19 (0.85-1.65)	0.61 (0.39-0.89)	0.58 (0.37-0.84)
Congo	0.29 (0.23-0.38)	0.16 (0.12-0.21)	0.14 (0.09-0.19)	0.25 (0.19-0.32)	0.13 (0.1-0.17)	0.11 (0.08-0.16)	7.47 (5.63-9.75)	4.02 (3.01-5.38)	3.45 (2.37-4.9)
Cook Islands	0 (0-0)	0 (0-0)	0 (0-0)	0 (0-0)	0 (0-0)	0 (0-0)	0.03 (0.03-0.04)	0.02 (0.01-0.02)	0.02 (0.01-0.02)
Costa Rica	1.97 (1.71-2.32)	1.26 (1.08-1.48)	0.71 (0.62-0.83)	0.83 (0.72-0.96)	0.42 (0.36-0.49)	0.41 (0.35-0.48)	20.36 (17.64-23.65)	10.78 (9.3-12.55)	9.58 (8.16-11.23)
Croatia	4.48 (3.82-5.16)	2.59 (2.15-3.03)	1.89 (1.6-2.19)	2.31 (1.96-2.67)	1.34 (1.13-1.58)	0.96 (0.82-1.12)	45.57 (39.33-52.19)	27.87 (23.54-32.48)	17.71 (15.03-20.5)
Cuba	9.46 (7.94-11.03)	4.18 (3.41-4.93)	5.28 (4.42-6.26)	3.53 (3.01-4.08)	1.51 (1.27-1.77)	2.02 (1.7-2.36)	74.4 (62.86-86.95)	32.94 (27.37-39.03)	41.46 (34.7-48.97)
Cyprus	0.71 (0.59-0.85)	0.41 (0.33-0.5)	0.3 (0.23-0.38)	0.25 (0.21-0.3)	0.14 (0.12-0.17)	0.11 (0.08-0.14)	4.98 (4.17-6.02)	2.95 (2.42-3.62)	2.03 (1.59-2.59)
Czechia	8.64 (7.24-10.24)	5.31 (4.4-6.35)	3.34 (2.76-3.97)	4.53 (3.8-5.35)	2.67 (2.26-3.17)	1.85 (1.55-2.19)	92.44 (77.34-109.8)	57.19 (47.76-68.09)	35.24 (29.22-41.77)
Côte d'Ivoire	0.47 (0.34-0.66)	0.24 (0.17-0.37)	0.23 (0.16-0.31)	0.4 (0.29-0.56)	0.2 (0.15-0.3)	0.2 (0.14-0.27)	11.49 (8.14-16.46)	5.93 (4.18-9.28)	5.56 (3.86-7.68)
Democratic People's Republic of Korea	5.15 (3.46-7.55)	2.75 (1.86-4.33)	2.4 (1.48-3.79)	3.41 (2.28-5.06)	1.75 (1.19-2.76)	1.66 (1.02-2.62)	95.1 (63.45-143.7)	53.55 (35.61-86.12)	41.55 (25.49-67.08)
Democratic Republic of the Congo	2.45 (1.7-3.52)	1.23 (0.79-1.99)	1.22 (0.79-1.77)	2.17 (1.5-3.15)	1.08 (0.69-1.81)	1.09 (0.7-1.61)	63.85 (43.73-93.52)	32.84 (20.89-55.29)	31 (19.67-45.9)
Denmark	5.27 (4.54-6.01)	2.83 (2.4-3.24)	2.45 (2.1-2.8)	2.45 (2.11-2.76)	1.27 (1.08-1.46)	1.18 (1-1.34)	45.21 (39.1-50.74)	24.69 (21.46-27.96)	20.52 (17.64-23.15)
Djibouti	0.08 (0.06-0.12)	0.05 (0.03-0.08)	0.03 (0.02-0.04)	0.07 (0.05-0.1)	0.04 (0.03-0.06)	0.03 (0.02-0.04)	2.07 (1.43-2.99)	1.33 (0.87-1.96)	0.74 (0.47-1.14)
Dominica	0.02 (0.02-0.02)	0.01 (0.01-0.01)	0.01 (0.01-0.01)	0.01 (0.01-0.01)	0.01 (0-0.01)	0.01 (0-0.01)	0.28 (0.23-0.34)	0.15 (0.12-0.19)	0.13 (0.1-0.16)
Dominican Republic	1.55 (1.21-1.97)	0.83 (0.59-1.12)	0.72 (0.52-0.96)	0.9 (0.7-1.15)	0.47 (0.34-0.64)	0.43 (0.31-0.58)	22.07 (17.24-28.38)	12.09 (8.72-16.08)	9.98 (7.39-13.23)
Ecuador	2.12 (1.65-2.69)	1.01 (0.75-1.33)	1.11 (0.86-1.43)	1.5 (1.18-1.9)	0.69 (0.52-0.9)	0.81 (0.63-1.03)	35.83 (27.57-45.73)	17.07 (12.86-22.32)	18.76 (14.41-24.27)
Egypt	8.12 (6.62-9.96)	4.06 (3.23-5.11)	4.06 (3.2-5.14)	5.01 (4.08-6.13)	2.75 (2.19-3.4)	2.27 (1.81-2.84)	149.5 (121.97-183)	82.14 (65.09-101.53)	67.36 (53.13-85.62)
El Salvador	0.91 (0.74-1.13)	0.5 (0.38-0.62)	0.42 (0.33-0.52)	0.51 (0.41-0.63)	0.22 (0.17-0.27)	0.29 (0.23-0.36)	12.74 (10.19-15.57)	5.97 (4.55-7.41)	6.77 (5.28-8.37)
Equatorial Guinea	0.06 (0.04-0.09)	0.04 (0.02-0.05)	0.03 (0.02-0.04)	0.05 (0.03-0.07)	0.03 (0.02-0.04)	0.02 (0.01-0.03)	1.45 (0.95-2.1)	0.84 (0.54-1.23)	0.6 (0.34-1.02)

Eritrea	0.33<br>(0.24-0.44)	0.16<br>(0.11-0.24)	0.17<br>(0.12-0.23)	0.29<br>(0.21-0.39)	0.14<br>(0.09-0.21)	0.15<br>(0.11-0.2)	9.05<br>(6.52-12.43)	4.65<br>(2.99-6.99)	4.4<br>(3.08-6.04)
Estonia	1.12<br>(0.91-1.33)	0.58<br>(0.47-0.67)	0.55<br>(0.43-0.66)	0.53<br>(0.43-0.62)	0.27<br>(0.22-0.31)	0.26<br>(0.21-0.31)	10.06<br>(8.19-11.78)	5.45<br>(4.5-6.31)	4.61<br>(3.68-5.48)
Eswatini	0.09<br>(0.06-0.13)	0.05<br>(0.03-0.07)	0.04<br>(0.02-0.06)	0.08<br>(0.05-0.11)	0.04<br>(0.03-0.06)	0.04<br>(0.02-0.05)	2.4<br>(1.52-3.34)	1.42<br>(0.88-1.97)	0.99<br>(0.56-1.53)
Ethiopia	6.7<br>(5.58-8.04)	4.55<br>(3.57-5.85)	2.16<br>(1.72-2.72)	6.12<br>(5.09-7.35)	4.18<br>(3.28-5.35)	1.93<br>(1.56-2.44)	158.08<br>(130.52-190.98)	106.63<br>(83.23-137.68)	51.45<br>(40.52-65.81)
Fiji	0.08<br>(0.06-0.11)	0.04<br>(0.03-0.06)	0.04<br>(0.03-0.05)	0.07<br>(0.05-0.09)	0.04<br>(0.02-0.05)	0.03<br>(0.02-0.04)	1.81<br>(1.32-2.38)	0.99<br>(0.59-1.34)	0.82<br>(0.61-1.11)
Finland	3.89<br>(3.38-4.37)	2.1<br>(1.83-2.37)	1.79<br>(1.51-2.05)	1.58<br>(1.35-1.8)	0.81<br>(0.7-0.92)	0.77<br>(0.63-0.89)	29.54<br>(25.81-33.22)	16.24<br>(14.3-18.2)	13.3<br>(11.31-15.01)
France	58.1<br>(49.41-66.07)	31.45<br>(27.22-35.49)	26.64<br>(21.8-30.7)	24.58<br>(20.49-28.21)	12.7<br>(10.86-14.39)	11.88<br>(9.47-13.93)	437.36<br>(375.39-495.05)	243.94<br>(212.94-274.08)	193.42<br>(160.51-221.18)
Gabon	0.16<br>(0.11-0.21)	0.1<br>(0.07-0.13)	0.06<br>(0.04-0.08)	0.13<br>(0.09-0.17)	0.08<br>(0.06-0.1)	0.05<br>(0.03-0.07)	3.61<br>(2.57-4.83)	2.3<br>(1.6-3.09)	1.31<br>(0.87-1.86)
Gambia	0.03<br>(0.02-0.04)	0.01<br>(0.01-0.02)	0.02<br>(0.01-0.02)	0.03<br>(0.02-0.04)	0.01<br>(0.01-0.02)	0.02<br>(0.01-0.02)	0.75<br>(0.56-1)	0.34<br>(0.24-0.48)	0.41<br>(0.28-0.56)
Georgia	1.09<br>(0.94-1.26)	0.62<br>(0.53-0.72)	0.47<br>(0.41-0.55)	0.8<br>(0.68-0.91)	0.44<br>(0.38-0.5)	0.36<br>(0.31-0.41)	18.6<br>(15.91-21.53)	10.86<br>(9.28-12.56)	7.74<br>(6.64-8.97)
Germany	73.77<br>(62.96-83.62)	41.21<br>(35.86-46.71)	32.56<br>(26.61-37.43)	30.92<br>(25.82-35.2)	16.54<br>(14.36-18.71)	14.37<br>(11.62-16.74)	586.27<br>(514.07-658.73)	335.21<br>(296.08-378.05)	251.07<br>(213-287.13)
Ghana	1.15<br>(0.9-1.45)	0.51<br>(0.39-0.65)	0.65<br>(0.47-0.85)	0.99<br>(0.77-1.24)	0.43<br>(0.33-0.55)	0.56<br>(0.42-0.73)	26.62<br>(20.38-34.05)	11.81<br>(8.96-15.27)	14.8<br>(10.87-19.7)
Greece	8.05<br>(7.05-9.01)	4.56<br>(4.06-5.13)	3.49<br>(2.98-3.91)	3.88<br>(3.36-4.32)	2.13<br>(1.88-2.37)	1.75<br>(1.47-1.97)	68.38<br>(60.46-75.65)	39.29<br>(35.41-43.58)	29.09<br>(25.24-32.56)
Greenland	0.03<br>(0.02-0.03)	0.02<br>(0.01-0.02)	0.01<br>(0.01-0.01)	0.02<br>(0.01-0.02)	0.01<br>(0.01-0.01)	0.01<br>(0.01-0.01)	0.41<br>(0.34-0.49)	0.24<br>(0.19-0.29)	0.17<br>(0.13-0.22)
Grenada	0.04<br>(0.03-0.04)	0.02<br>(0.02-0.02)	0.02<br>(0.01-0.02)	0.02<br>(0.02-0.02)	0.01<br>(0.01-0.01)	0.01<br>(0.01-0.01)	0.44<br>(0.37-0.52)	0.24<br>(0.19-0.29)	0.2<br>(0.18-0.24)
Guam	0.04<br>(0.03-0.04)	0.02<br>(0.02-0.03)	0.01<br>(0.01-0.02)	0.02<br>(0.02-0.02)	0.01<br>(0.01-0.02)	0.01<br>(0.01-0.01)	0.59<br>(0.52-0.68)	0.38<br>(0.32-0.44)	0.22<br>(0.17-0.26)
Guatemala	1.09<br>(0.93-1.28)	0.58<br>(0.49-0.7)	0.5<br>(0.43-0.59)	0.74<br>(0.63-0.86)	0.34<br>(0.29-0.41)	0.4<br>(0.34-0.46)	19.71<br>(16.78-23.07)	9.38<br>(7.92-11.14)	10.32<br>(8.77-12.01)
Guinea	0.27<br>(0.2-0.36)	0.16<br>(0.12-0.23)	0.11<br>(0.08-0.15)	0.25<br>(0.18-0.34)	0.15<br>(0.11-0.21)	0.1<br>(0.07-0.14)	6.69<br>(4.79-9.2)	3.96<br>(2.88-5.66)	2.73<br>(1.91-4.05)
Guinea-Bissau	0.06<br>(0.04-0.07)	0.03<br>(0.02-0.04)	0.03<br>(0.02-0.04)	0.05<br>(0.04-0.07)	0.03<br>(0.02-0.04)	0.03<br>(0.02-0.04)	1.61<br>(1.17-2.09)	0.82<br>(0.6-1.1)	0.79<br>(0.54-1.11)
Guyana	0.13<br>(0.09-0.16)	0.07<br>(0.05-0.09)	0.06<br>(0.04-0.08)	0.08<br>(0.06-0.1)	0.04<br>(0.03-0.06)	0.04<br>(0.03-0.05)	2.2<br>(1.65-2.87)	1.2<br>(0.89-1.58)	1<br>(0.74-1.29)
Haiti	1.16<br>(0.78-1.66)	0.48<br>(0.33-0.71)	0.68<br>(0.37-1.06)	0.9<br>(0.61-1.28)	0.38<br>(0.26-0.55)	0.52<br>(0.29-0.81)	25.55<br>(17.16-36.5)	10.58<br>(7.15-15.72)	14.97<br>(8.12-23.19)
Honduras	0.52<br>(0.41-0.68)	0.25<br>(0.19-0.33)	0.28<br>(0.2-0.39)	0.38<br>(0.3-0.49)	0.15<br>(0.12-0.2)	0.22<br>(0.16-0.31)	9.93<br>(7.61-13.11)	3.93<br>(3.01-5.23)	6<br>(4.3-8.34)
Hungary	9.41<br>(7.84-11.26)	5.64<br>(4.69-6.72)	3.77<br>(3.14-4.5)	5.21<br>(4.37-6.24)	2.98<br>(2.5-3.58)	2.23<br>(1.84-2.67)	113.14<br>(95.76-135.4)	68.57<br>(57.6-82.03)	44.57<br>(37.51-53.19)
Iceland	0.19<br>(0.16-0.22)	0.1<br>(0.08-0.12)	0.09<br>(0.08-0.1)	0.07<br>(0.06-0.08)	0.04<br>(0.03-0.04)	0.03<br>(0.03-0.04)	1.36<br>(1.17-1.58)	0.74<br>(0.63-0.86)	0.62<br>(0.53-0.72)
India	69.41<br>(61.76-78.52)	35.43<br>(29.19-41.7)	33.98<br>(28.52-40.35)	54.02<br>(48.11-61.28)	27.68<br>(22.91-32.71)	26.34<br>(22.12-31.36)	1541.7<br>(1369.02-1755.41)	774.88<br>(640.68-923.96)	766.82<br>(642.4-911.59)
Indonesia	35.78<br>(26.68-45)	19.78<br>(13.54-26.72)	16<br>(11.09-22.44)	27.11<br>(20.43-33.82)	14.98<br>(10.39-20.09)	12.12<br>(8.41-16.76)	767.83<br>(572.47-973.19)	429.26<br>(295.41-589.2)	338.57<br>(232.57-475.39)
Iran (Islamic Republic of Iran)	10.42<br>(9.03-11.68)	5.18<br>(3.9-5.92)	5.25<br>(4.55-5.94)	5.41<br>(4.63-6.01)	2.99<br>(2.3-3.4)	2.42<br>(2.12-2.75)	142.54<br>(122.42-158.79)	79.52<br>(62.06-90.86)	63.02<br>(54.98-71.77)
Iraq	2.51<br>(1.89-3.24)	1.36<br>(0.98-1.76)	1.15<br>(0.86-1.56)	1.41<br>(1.07-1.77)	0.84<br>(0.61-1.08)	0.57<br>(0.43-0.74)	41.15<br>(30.69-53.22)	24.43<br>(17.73-31.96)	16.72<br>(12.3-22.45)
Ireland	3.24<br>(2.78-3.7)	1.98<br>(1.69-2.28)	1.26<br>(1.05-1.47)	1.18<br>(1-1.35)	0.71<br>(0.6-0.82)	0.47<br>(0.38-0.55)	23.97<br>(20.73-27.2)	14.82<br>(12.77-17.1)	9.15<br>(7.65-10.54)
Israel	3.71<br>(3.08-4.29)	1.99<br>(1.66-2.33)	1.72<br>(1.4-1.98)	1.76<br>(1.42-2.05)	0.91<br>(0.75-1.06)	0.85<br>(0.67-0.99)	32.99<br>(27.95-37.85)	18<br>(15.17-20.76)	14.99<br>(12.43-17.17)

Italy	56.71 (49.89-61.49)	32.09 (29.02-34.44)	24.62 (20.66-27.45)	23.43 (19.95-25.63)	12.78 (11.54-13.66)	10.65 (8.46-11.99)	427.74 (381.47-461.6)	244.56 (225.59-259.87)	183.19 (155.76-202.95)
Jamaica	0.98 (0.74-1.3)	0.52 (0.37-0.71)	0.46 (0.35-0.59)	0.47 (0.37-0.62)	0.24 (0.18-0.33)	0.23 (0.18-0.29)	10.91 (8.26-14.55)	5.8 (4.2-7.91)	5.11 (3.94-6.59)
Japan	171.04 (148.91-184.89)	98.95 (90.77-104.6)	72.09 (56.59-81.79)	67.92 (56.45-74.36)	34.81 (31.7-36.62)	33.11 (24.56-37.93)	1171.59 (1020.79-1259.68)	664.04 (614.92-698.21)	507.55 (405.2-566.57)
Jordan	1.21 (0.93-1.65)	0.65 (0.49-0.87)	0.56 (0.4-0.83)	0.59 (0.46-0.8)	0.35 (0.27-0.47)	0.24 (0.17-0.36)	17.1 (13.06-23.27)	10.31 (7.71-13.97)	6.79 (4.82-10.02)
Kazakhstan	2.8 (2.38-3.27)	1.45 (1.23-1.68)	1.36 (1.12-1.58)	1.82 (1.55-2.12)	0.92 (0.79-1.07)	0.89 (0.74-1.04)	49.1 (42.01-56.79)	26 (22.24-30.07)	23.1 (19.31-27.01)
Kenya	1.8 (1.48-2.27)	0.82 (0.63-1.07)	0.98 (0.73-1.32)	1.52 (1.25-1.91)	0.68 (0.53-0.89)	0.84 (0.62-1.12)	43.75 (35.79-55.87)	20.15 (15.38-26.9)	23.6 (17.22-32.44)
Kiribati	0.01 (0.01-0.01)	0 (0-0.01)	0 (0-0)	0.01 (0-0.01)	0 (0-0)	0 (0-0)	0.18 (0.13-0.25)	0.1 (0.07-0.15)	0.08 (0.06-0.11)
Kuwait	0.61 (0.49-0.76)	0.37 (0.28-0.48)	0.24 (0.2-0.29)	0.23 (0.19-0.29)	0.16 (0.13-0.21)	0.07 (0.06-0.08)	6.72 (5.36-8.44)	4.56 (3.51-5.94)	2.17 (1.8-2.61)
Kyrgyzstan	0.47 (0.39-0.57)	0.24 (0.19-0.29)	0.23 (0.19-0.28)	0.33 (0.27-0.39)	0.16 (0.13-0.2)	0.16 (0.13-0.19)	9.61 (7.88-11.53)	5.01 (4.07-6.07)	4.6 (3.76-5.55)
Lao People's Democratic Republic	0.69 (0.49-0.91)	0.37 (0.26-0.52)	0.32 (0.21-0.43)	0.57 (0.41-0.76)	0.31 (0.22-0.43)	0.26 (0.17-0.35)	16.73 (11.67-22.48)	9.18 (6.22-12.87)	7.54 (4.89-10.3)
Latvia	1.18 (0.97-1.41)	0.57 (0.47-0.67)	0.61 (0.49-0.75)	0.7 (0.58-0.84)	0.34 (0.27-0.4)	0.37 (0.3-0.44)	14.21 (11.64-17)	7.33 (5.99-8.56)	6.88 (5.55-8.42)
Lebanon	1.24 (1-1.58)	0.7 (0.56-0.9)	0.54 (0.41-0.75)	0.7 (0.57-0.88)	0.42 (0.34-0.55)	0.27 (0.2-0.39)	14.29 (11.66-18.13)	8.7 (6.96-11.23)	5.59 (4.23-7.76)
Lesotho	0.15 (0.1-0.21)	0.07 (0.05-0.1)	0.08 (0.05-0.12)	0.13 (0.09-0.19)	0.06 (0.04-0.09)	0.07 (0.05-0.11)	3.79 (2.59-5.5)	1.9 (1.28-2.74)	1.89 (1.18-3.02)
Liberia	0.12 (0.07-0.18)	0.06 (0.03-0.1)	0.06 (0.04-0.09)	0.1 (0.06-0.16)	0.05 (0.03-0.09)	0.05 (0.03-0.08)	2.92 (1.77-4.52)	1.47 (0.81-2.51)	1.45 (0.87-2.22)
Libya	1.17 (0.85-1.59)	0.59 (0.41-0.81)	0.59 (0.42-0.81)	0.69 (0.5-0.92)	0.38 (0.27-0.52)	0.3 (0.22-0.42)	19.36 (14.09-26.54)	10.64 (7.46-14.64)	8.72 (6.14-12.24)
Lithuania	1.78 (1.47-2.09)	0.93 (0.77-1.09)	0.85 (0.69-1.01)	1.04 (0.86-1.24)	0.52 (0.43-0.61)	0.52 (0.43-0.63)	20.79 (17.22-24.51)	11.07 (9.18-13.02)	9.71 (7.93-11.65)
Luxembourg	0.4 (0.36-0.45)	0.23 (0.2-0.26)	0.18 (0.15-0.2)	0.17 (0.15-0.19)	0.09 (0.08-0.1)	0.08 (0.07-0.09)	3.19 (2.81-3.55)	1.84 (1.6-2.06)	1.35 (1.16-1.53)
Madagascar	0.94 (0.67-1.23)	0.46 (0.31-0.64)	0.48 (0.34-0.64)	0.82 (0.59-1.07)	0.41 (0.27-0.56)	0.41 (0.3-0.55)	25.3 (18.06-33.19)	12.34 (8.24-17.11)	12.95 (9.23-17.67)
Malawi	0.36 (0.27-0.49)	0.18 (0.13-0.26)	0.18 (0.12-0.26)	0.31 (0.24-0.43)	0.16 (0.12-0.22)	0.16 (0.11-0.23)	9.13 (7-12.94)	4.7 (3.43-6.93)	4.43 (2.95-6.73)
Malaysia	7.36 (6.41-8.29)	4.23 (3.47-4.98)	3.13 (2.62-3.7)	4.78 (4.15-5.34)	2.76 (2.26-3.25)	2.01 (1.69-2.4)	122.3 (106.77-136.77)	71.27 (58.71-83.97)	51.03 (43.06-60.02)
Maldives	0.02 (0.02-0.03)	0.01 (0.01-0.02)	0.01 (0.01-0.01)	0.01 (0.01-0.02)	0.01 (0.01-0.01)	0.01 (0-0.01)	0.36 (0.29-0.45)	0.23 (0.17-0.29)	0.13 (0.1-0.17)
Mali	0.63 (0.48-0.8)	0.38 (0.28-0.5)	0.25 (0.18-0.34)	0.56 (0.43-0.72)	0.35 (0.25-0.46)	0.22 (0.16-0.3)	15.64 (11.83-20.13)	9.29 (6.78-12.32)	6.35 (4.55-9.14)
Malta	0.33 (0.28-0.38)	0.16 (0.14-0.19)	0.17 (0.14-0.2)	0.14 (0.12-0.16)	0.07 (0.06-0.08)	0.07 (0.06-0.08)	2.74 (2.33-3.14)	1.42 (1.22-1.65)	1.32 (1.09-1.54)
Marshall Islands	0 (0-0.01)	0 (0-0)	0 (0-0)	0 (0-0)	0 (0-0)	0 (0-0)	0.12 (0.09-0.16)	0.07 (0.04-0.09)	0.05 (0.03-0.08)
Mauritania	0.16 (0.12-0.22)	0.08 (0.05-0.11)	0.08 (0.06-0.11)	0.14 (0.1-0.18)	0.07 (0.05-0.1)	0.07 (0.05-0.09)	3.35 (2.44-4.58)	1.62 (1.09-2.38)	1.73 (1.2-2.35)
Mauritius	0.39 (0.36-0.43)	0.23 (0.21-0.25)	0.17 (0.15-0.18)	0.25 (0.23-0.27)	0.14 (0.13-0.15)	0.11 (0.1-0.12)	6.43 (5.89-6.91)	3.82 (3.48-4.1)	2.61 (2.35-2.86)
Mexico	20.66 (18.13-23.26)	13.17 (10.93-15.6)	7.49 (6.32-8.69)	10.93 (9.65-12.24)	5.84 (4.9-6.91)	5.09 (4.32-5.86)	292.72 (257.98-329.41)	160.99 (134.22-190.16)	131.72 (110.75-153.91)
Micronesia (Federated States of Micronesia)	0.01 (0.01-0.01)	0.01 (0-0.01)	0.01 (0-0.01)	0.01 (0.01-0.01)	0 (0-0.01)	0 (0-0.01)	0.24 (0.17-0.34)	0.13 (0.09-0.19)	0.11 (0.07-0.17)
Monaco	0.07 (0.05-0.08)	0.04 (0.03-0.05)	0.03 (0.02-0.04)	0.03 (0.02-0.03)	0.02 (0.01-0.02)	0.01 (0.01-0.02)	0.5 (0.4-0.6)	0.3 (0.24-0.36)	0.21 (0.15-0.26)
Mongolia	0.24 (0.19-0.3)	0.12 (0.08-0.17)	0.12 (0.08-0.15)	0.18 (0.14-0.22)	0.09 (0.06-0.12)	0.09 (0.06-0.12)	5.4 (4.2-6.65)	2.85 (1.98-3.86)	2.55 (1.83-3.33)

Montenegro	0.31<br>(0.26-0.37)	0.19<br>(0.15-0.24)	0.12<br>(0.09-0.15)	0.17<br>(0.14-0.2)	0.1<br>(0.08-0.12)	0.07<br>(0.05-0.09)	3.67<br>(3.02-4.48)	2.27<br>(1.78-2.84)	1.39<br>(1.06-1.77)
Morocco	4<br>(2.88-5.34)	1.77<br>(1.25-2.41)	2.23<br>(1.4-3.31)	2.72<br>(1.99-3.53)	1.34<br>(0.95-1.81)	1.39<br>(0.89-2.02)	71.88<br>(52.06-95.04)	33.51<br>(23.26-45.36)	38.37<br>(23.54-57.56)
Mozambique	0.39<br>(0.29-0.5)	0.21<br>(0.16-0.27)	0.18<br>(0.12-0.24)	0.37<br>(0.27-0.46)	0.2<br>(0.15-0.25)	0.17<br>(0.12-0.23)	9.54<br>(7.06-12.39)	5.43<br>(4.14-6.97)	4.1<br>(2.7-5.68)
Myanmar	6.68<br>(4.9-8.66)	3.45<br>(2.46-4.57)	3.23<br>(2.21-4.33)	5.37<br>(3.96-6.92)	2.76<br>(1.99-3.61)	2.61<br>(1.78-3.49)	147.25<br>(107.48-193)	77.43<br>(54.68-103.74)	69.82<br>(47.16-94.11)
Namibia	0.1<br>(0.07-0.13)	0.06<br>(0.04-0.07)	0.05<br>(0.03-0.06)	0.08<br>(0.06-0.11)	0.04<br>(0.03-0.06)	0.04<br>(0.03-0.05)	2.39<br>(1.68-3.19)	1.34<br>(0.98-1.76)	1.05<br>(0.69-1.47)
Nauru	0<br>(0-0)	0<br>(0-0)	0<br>(0-0)	0<br>(0-0)	0<br>(0-0)	0<br>(0-0)	0.03<br>(0.02-0.04)	0.02<br>(0.01-0.02)	0.01<br>(0.01-0.02)
Nepal	1.13<br>(0.84-1.5)	0.55<br>(0.37-0.82)	0.58<br>(0.41-0.8)	0.92<br>(0.68-1.22)	0.46<br>(0.31-0.69)	0.47<br>(0.33-0.63)	25.51<br>(18.73-33.83)	12.12<br>(8.19-18.24)	13.39<br>(9.21-18.28)
Netherlands	24.52<br>(21.69-27.07)	14.1<br>(12.75-15.5)	10.42<br>(8.92-11.97)	7.44<br>(6.51-8.3)	4.12<br>(3.7-4.57)	3.33<br>(2.79-3.82)	151.49<br>(135.28-167.03)	86.77<br>(78.71-95.68)	64.73<br>(55.87-73.52)
New Zealand	4.34<br>(3.76-4.95)	2.37<br>(2.08-2.69)	1.97<br>(1.65-2.31)	1.6<br>(1.38-1.82)	0.86<br>(0.75-0.97)	0.74<br>(0.61-0.87)	31.92<br>(27.86-36.06)	17.81<br>(15.89-19.86)	14.12<br>(12.06-16.37)
Nicaragua	0.5<br>(0.4-0.61)	0.28<br>(0.21-0.36)	0.22<br>(0.17-0.28)	0.28<br>(0.23-0.34)	0.13<br>(0.1-0.16)	0.15<br>(0.12-0.19)	7.56<br>(6.07-9.24)	3.62<br>(2.77-4.62)	3.94<br>(3-5.06)
Niger	0.38<br>(0.27-0.52)	0.18<br>(0.12-0.27)	0.2<br>(0.13-0.29)	0.35<br>(0.25-0.48)	0.17<br>(0.11-0.25)	0.18<br>(0.12-0.27)	9.47<br>(6.65-13.04)	4.45<br>(2.91-6.75)	5.02<br>(3.17-7.3)
Nigeria	5.22<br>(4.01-6.63)	2.66<br>(1.88-3.58)	2.55<br>(1.76-3.62)	4.62<br>(3.64-5.76)	2.38<br>(1.69-3.17)	2.24<br>(1.6-3.1)	122.93<br>(91.48-158.97)	62.65<br>(44.05-84.8)	60.28<br>(40.77-86.51)
Niue	0<br>(0-0)	0<br>(0-0)	0<br>(0-0)	0<br>(0-0)	0<br>(0-0)	0<br>(0-0)	0.01<br>(0-0.01)	0<br>(0-0)	0<br>(0-0)
North Macedonia	0.95<br>(0.74-1.16)	0.55<br>(0.42-0.7)	0.4<br>(0.3-0.53)	0.59<br>(0.47-0.71)	0.33<br>(0.26-0.42)	0.25<br>(0.19-0.34)	13.34<br>(10.51-16.4)	7.79<br>(6.02-9.91)	5.55<br>(4.15-7.4)
Northern Mariana Islands	0.01<br>(0.01-0.01)	0.01<br>(0.01-0.01)	0<br>(0-0)	0.01<br>(0.01-0.01)	0<br>(0-0.01)	0<br>(0-0)	0.19<br>(0.16-0.22)	0.13<br>(0.1-0.15)	0.07<br>(0.05-0.08)
Norway	4.8<br>(4.27-5.2)	2.5<br>(2.28-2.71)	2.3<br>(1.96-2.54)	1.99<br>(1.72-2.16)	0.99<br>(0.89-1.06)	1<br>(0.83-1.11)	36.61<br>(32.95-39.35)	19.65<br>(18.09-21.14)	16.96<br>(14.75-18.5)
Oman	0.13<br>(0.1-0.18)	0.08<br>(0.06-0.12)	0.05<br>(0.03-0.06)	0.06<br>(0.05-0.08)	0.04<br>(0.03-0.06)	0.02<br>(0.01-0.02)	1.83<br>(1.35-2.53)	1.3<br>(0.93-1.85)	0.53<br>(0.39-0.72)
Pakistan	8.45<br>(6.89-10.54)	4.34<br>(3.21-5.84)	4.11<br>(3.01-5.55)	7.23<br>(5.93-9.02)	3.75<br>(2.77-5.07)	3.47<br>(2.55-4.62)	212.74<br>(169.93-266.86)	107.93<br>(78.79-147.28)	104.8<br>(75.95-141.85)
Palau	0<br>(0-0)	0<br>(0-0)	0<br>(0-0)	0<br>(0-0)	0<br>(0-0)	0<br>(0-0)	0.06<br>(0.05-0.08)	0.01<br>(0.01-0.02)	0.05<br>(0.04-0.07)
Palestine	0.59<br>(0.49-0.71)	0.29<br>(0.23-0.35)	0.3<br>(0.24-0.38)	0.34<br>(0.28-0.4)	0.18<br>(0.14-0.22)	0.16<br>(0.13-0.19)	9.44<br>(7.75-11.32)	5.09<br>(3.96-6.19)	4.34<br>(3.5-5.33)
Panama	1.23<br>(0.94-1.51)	0.85<br>(0.63-1.06)	0.38<br>(0.3-0.46)	0.5<br>(0.38-0.6)	0.25<br>(0.19-0.31)	0.24<br>(0.19-0.29)	12.03<br>(9.27-14.75)	6.4<br>(4.78-7.93)	5.63<br>(4.44-6.85)
Papua New Guinea	0.21<br>(0.16-0.27)	0.09<br>(0.07-0.11)	0.12<br>(0.09-0.16)	0.17<br>(0.13-0.22)	0.07<br>(0.06-0.1)	0.1<br>(0.07-0.13)	5.7<br>(4.33-7.24)	2.38<br>(1.8-3.04)	3.32<br>(2.45-4.28)
Paraguay	0.85<br>(0.66-1.1)	0.46<br>(0.33-0.62)	0.39<br>(0.28-0.51)	0.59<br>(0.46-0.75)	0.31<br>(0.22-0.41)	0.28<br>(0.21-0.37)	14.61<br>(11.24-18.66)	7.87<br>(5.66-10.4)	6.74<br>(4.94-8.9)
Peru	4.9<br>(3.65-6.47)	2.4<br>(1.7-3.23)	2.49<br>(1.8-3.3)	3.1<br>(2.31-3.99)	1.46<br>(1.04-1.89)	1.64<br>(1.2-2.16)	71.62<br>(53.41-94.5)	35.14<br>(24.69-47.68)	36.48<br>(26.36-48.49)
Philippines	14.07<br>(11.84-16.62)	8.25<br>(6.37-10.36)	5.82<br>(4.67-7.2)	10.58<br>(8.92-12.37)	6.12<br>(4.74-7.71)	4.46<br>(3.56-5.48)	311.02<br>(259.6-365.69)	187.51<br>(144.56-234.98)	123.51<br>(97.03-154.16)
Poland	25.42<br>(22.83-27.67)	14.68<br>(13.13-16.26)	10.74<br>(9.39-11.95)	18.18<br>(16.29-19.76)	10.04<br>(8.97-11.08)	8.14<br>(7.04-9.06)	366.68<br>(332.27-397.99)	216.45<br>(193.01-238.26)	150.24<br>(131.56-167.75)
Portugal	9.84<br>(8.4-11.27)	5.85<br>(5.07-6.71)	3.99<br>(3.31-4.61)	4.69<br>(3.97-5.32)	2.65<br>(2.29-3.01)	2.03<br>(1.64-2.38)	87.87<br>(75.97-100.29)	52.67<br>(45.87-59.99)	35.2<br>(29.35-40.51)
Puerto Rico	3.08<br>(2.55-3.71)	1.79<br>(1.45-2.21)	1.29<br>(1.06-1.54)	1<br>(0.84-1.21)	0.54<br>(0.45-0.66)	0.46<br>(0.38-0.54)	21.16<br>(17.67-25.43)	12.32<br>(10.08-15.04)	8.83<br>(7.28-10.49)
Qatar	0.23<br>(0.17-0.31)	0.13<br>(0.09-0.18)	0.1<br>(0.07-0.14)	0.08<br>(0.06-0.11)	0.05<br>(0.04-0.07)	0.03<br>(0.02-0.04)	2.68<br>(1.98-3.71)	1.77<br>(1.27-2.54)	0.92<br>(0.66-1.26)
Republic of Korea	33.14<br>(27.17-39.02)	20.19<br>(16.14-24.5)	12.95<br>(9.76-16.63)	11.66<br>(9.6-13.74)	6.52<br>(5.21-7.85)	5.14<br>(3.81-6.76)	244.91<br>(204.21-286.79)	147.83<br>(119.92-178.87)	97.08<br>(74.65-124.25)

Republic of Moldova	1.9<br>(1.61-2.25)	1.13<br>(0.94-1.34)	0.77<br>(0.65-0.91)	1.09<br>(0.92-1.28)	0.65<br>(0.54-0.77)	0.44<br>(0.38-0.51)	26.79<br>(22.64-31.41)	16.42<br>(13.73-19.45)	10.37<br>(8.89-12.08)
Romania	14.07<br>(11.8-16.88)	8.12<br>(6.83-9.71)	5.95<br>(4.91-7.2)	8.03<br>(6.76-9.5)	4.69<br>(3.99-5.52)	3.34<br>(2.81-4)	177.26<br>(150.6-210.78)	107.7<br>(91.95-127.43)	69.56<br>(58.76-83.48)
Russian Federation	82.02<br>(75.11-88.82)	36.44<br>(31.97-40.12)	45.58<br>(40.68-50.14)	45.88<br>(42.13-49.67)	20.57<br>(18.16-22.68)	25.31<br>(22.53-27.7)	1029.23<br>(944.84-1113.83)	497.31<br>(437.23-550.12)	531.92<br>(473.73-584.03)
Rwanda	0.6<br>(0.42-0.81)	0.31<br>(0.2-0.45)	0.29<br>(0.18-0.42)	0.53<br>(0.37-0.72)	0.27<br>(0.17-0.39)	0.26<br>(0.15-0.37)	14.87<br>(10.27-20.52)	7.76<br>(5-11.31)	7.11<br>(4.24-10.28)
Saint Kitts and Nevis	0.02<br>(0.02-0.03)	0.01<br>(0.01-0.02)	0.01<br>(0.01-0.01)	0.01<br>(0.01-0.01)	0.01<br>(0-0.01)	0<br>(0-0.01)	0.26<br>(0.21-0.31)	0.16<br>(0.13-0.19)	0.1<br>(0.08-0.12)
Saint Lucia	0.05<br>(0.04-0.06)	0.03<br>(0.02-0.03)	0.02<br>(0.02-0.03)	0.03<br>(0.02-0.03)	0.01<br>(0.01-0.02)	0.01<br>(0.01-0.02)	0.58<br>(0.48-0.71)	0.32<br>(0.25-0.39)	0.27<br>(0.22-0.32)
Saint Vincent and the Grenadines	0.03<br>(0.03-0.04)	0.02<br>(0.02-0.02)	0.01<br>(0.01-0.02)	0.02<br>(0.02-0.02)	0.01<br>(0.01-0.01)	0.01<br>(0.01-0.01)	0.42<br>(0.37-0.49)	0.23<br>(0.2-0.27)	0.19<br>(0.17-0.22)
Samoa	0.02<br>(0.01-0.02)	0.01<br>(0.01-0.01)	0.01<br>(0.01-0.01)	0.01<br>(0.01-0.02)	0.01<br>(0-0.01)	0.01<br>(0.01-0.01)	0.33<br>(0.26-0.41)	0.14<br>(0.1-0.18)	0.2<br>(0.15-0.25)
San Marino	0.02<br>(0.02-0.03)	0.01<br>(0.01-0.02)	0.01<br>(0.01-0.01)	0.01<br>(0.01-0.01)	0.01<br>(0-0.01)	0<br>(0-0.01)	0.17<br>(0.11-0.24)	0.1<br>(0.07-0.14)	0.07<br>(0.05-0.11)
Sao Tome and Principe	0.01<br>(0.01-0.02)	0.01<br>(0.01-0.01)	0<br>(0-0)	0.01<br>(0.01-0.01)	0.01<br>(0.01-0.01)	0<br>(0-0)	0.26<br>(0.21-0.34)	0.2<br>(0.15-0.27)	0.06<br>(0.05-0.08)
Saudi Arabia	3.46<br>(2.65-4.33)	1.95<br>(1.47-2.52)	1.51<br>(1.12-1.96)	1.45<br>(1.14-1.82)	0.99<br>(0.76-1.28)	0.46<br>(0.34-0.59)	50.31<br>(38.17-65.03)	33.83<br>(25.11-45.32)	16.47<br>(12.11-21.57)
Senegal	0.53<br>(0.4-0.7)	0.28<br>(0.21-0.38)	0.25<br>(0.18-0.33)	0.48<br>(0.36-0.63)	0.25<br>(0.19-0.33)	0.23<br>(0.17-0.3)	12.12<br>(9.11-16.1)	6.41<br>(4.75-8.85)	5.71<br>(4.1-7.62)
Serbia	5.79<br>(4.7-7.05)	3.69<br>(2.87-4.66)	2.09<br>(1.58-2.73)	3.63<br>(2.95-4.4)	2.14<br>(1.68-2.66)	1.49<br>(1.13-1.92)	77.94<br>(62.83-94.86)	47.16<br>(36.78-59.54)	30.78<br>(23.28-39.88)
Seychelles	0.03<br>(0.03-0.04)	0.02<br>(0.01-0.02)	0.01<br>(0.01-0.02)	0.02<br>(0.02-0.03)	0.01<br>(0.01-0.01)	0.01<br>(0.01-0.01)	0.56<br>(0.48-0.64)	0.32<br>(0.27-0.38)	0.24<br>(0.2-0.28)
Sierra Leone	0.2<br>(0.15-0.27)	0.1<br>(0.07-0.14)	0.1<br>(0.07-0.13)	0.18<br>(0.13-0.25)	0.09<br>(0.07-0.13)	0.09<br>(0.06-0.12)	4.83<br>(3.49-6.57)	2.47<br>(1.72-3.53)	2.36<br>(1.65-3.25)
Singapore	2.99<br>(2.58-3.41)	1.66<br>(1.45-1.9)	1.33<br>(1.12-1.55)	1.04<br>(0.9-1.19)	0.54<br>(0.48-0.61)	0.5<br>(0.42-0.58)	23.34<br>(20.59-26.39)	12.84<br>(11.35-14.53)	10.5<br>(9.08-12.11)
Slovakia	5.13<br>(4.19-6.11)	3.03<br>(2.38-3.78)	2.1<br>(1.56-2.75)	2.39<br>(1.94-2.85)	1.4<br>(1.1-1.74)	0.99<br>(0.73-1.31)	52.94<br>(43.61-63.34)	32.5<br>(25.53-40.41)	20.44<br>(15.32-26.85)
Slovenia	1.5<br>(1.23-1.85)	0.94<br>(0.77-1.15)	0.56<br>(0.44-0.7)	0.78<br>(0.63-0.96)	0.47<br>(0.38-0.56)	0.32<br>(0.25-0.39)	14.62<br>(11.84-17.89)	9.27<br>(7.49-11.36)	5.36<br>(4.24-6.65)
Solomon Islands	0.04<br>(0.03-0.05)	0.02<br>(0.01-0.03)	0.02<br>(0.01-0.03)	0.03<br>(0.02-0.04)	0.02<br>(0.01-0.02)	0.02<br>(0.01-0.02)	0.98<br>(0.67-1.31)	0.51<br>(0.36-0.74)	0.47<br>(0.31-0.7)
Somalia	0.6<br>(0.38-0.9)	0.28<br>(0.14-0.49)	0.32<br>(0.17-0.51)	0.55<br>(0.36-0.83)	0.26<br>(0.13-0.44)	0.3<br>(0.16-0.47)	17.56<br>(11.26-27.03)	8.42<br>(4.36-14.7)	9.15<br>(4.81-14.69)
South Africa	6.13<br>(5.49-6.89)	3.17<br>(2.72-3.73)	2.97<br>(2.58-3.37)	4.83<br>(4.35-5.38)	2.44<br>(2.11-2.85)	2.39<br>(2.09-2.71)	127.81<br>(114.22-144.69)	68.42<br>(58.47-81.76)	59.39<br>(51.57-68.25)
South Sudan	0.46<br>(0.31-0.61)	0.27<br>(0.17-0.37)	0.19<br>(0.12-0.27)	0.4<br>(0.28-0.54)	0.24<br>(0.16-0.33)	0.17<br>(0.11-0.23)	11.89<br>(8.21-15.94)	6.87<br>(4.45-9.52)	5.02<br>(3.25-7.09)
Spain	44.61<br>(38.46-50.14)	26.97<br>(23.54-30.18)	17.64<br>(14.48-20.42)	18.17<br>(15.27-20.68)	10.63<br>(9.22-12.01)	7.54<br>(5.91-8.86)	340.72<br>(294.87-383.57)	208.66<br>(183.15-233.21)	132.06<br>(108.51-152.4)
Sri Lanka	2.05<br>(1.35-2.87)	1.04<br>(0.63-1.48)	1.01<br>(0.66-1.42)	1.18<br>(0.78-1.64)	0.6<br>(0.37-0.84)	0.59<br>(0.4-0.83)	29.74<br>(19.28-41.88)	15.59<br>(9.51-22.55)	14.15<br>(9.24-20.12)
Sudan	1.9<br>(1.25-2.73)	0.89<br>(0.57-1.36)	1.01<br>(0.6-1.57)	1.33<br>(0.89-1.92)	0.69<br>(0.44-1.06)	0.64<br>(0.38-0.97)	40.01<br>(25.65-59.28)	19.75<br>(12.25-31.11)	20.26<br>(11.45-31.84)
Suriname	0.14<br>(0.11-0.18)	0.08<br>(0.06-0.1)	0.06<br>(0.05-0.08)	0.09<br>(0.06-0.11)	0.05<br>(0.03-0.06)	0.04<br>(0.03-0.05)	2.14<br>(1.63-2.69)	1.21<br>(0.87-1.57)	0.93<br>(0.71-1.21)
Sweden	7.58<br>(6.36-8.86)	3.94<br>(3.27-4.71)	3.64<br>(2.95-4.39)	3.49<br>(2.85-4.1)	1.73<br>(1.45-2.07)	1.76<br>(1.37-2.14)	61.59<br>(51.47-71.4)	32.23<br>(27.06-38.27)	29.36<br>(23.88-35)
Switzerland	5.12<br>(4.28-5.91)	2.85<br>(2.43-3.3)	2.27<br>(1.84-2.63)	2.09<br>(1.71-2.44)	1.14<br>(0.97-1.32)	0.95<br>(0.74-1.12)	38.83<br>(32.79-44.77)	22.09<br>(19.04-25.43)	16.74<br>(13.49-19.36)
Syrian Arab Republic	1.39<br>(1-1.9)	0.67<br>(0.46-0.96)	0.72<br>(0.51-0.98)	0.76<br>(0.55-1.04)	0.41<br>(0.28-0.58)	0.35<br>(0.25-0.47)	20.67<br>(14.85-28.74)	10.96<br>(7.5-15.81)	9.71<br>(6.86-13.11)
Taiwan (Province of China)	21.46<br>(18.75-24.09)	13.16<br>(11.5-14.83)	8.29<br>(7.18-9.35)	9.34<br>(8.06-10.55)	5.59<br>(4.86-6.32)	3.75<br>(3.15-4.33)	205.5<br>(180.53-229.55)	129.01<br>(114.03-144.7)	76.49<br>(66.91-85.46)

Tajikistan	0.34 (0.26-0.45)	0.19 (0.13-0.27)	0.15 (0.1-0.21)	0.27 (0.2-0.35)	0.15 (0.11-0.21)	0.12 (0.07-0.16)	8.36 (6.18-11.71)	4.75 (3.3-7.15)	3.6 (2.23-5.29)
Thailand	28.04 (21.23-35.85)	16.94 (11.53-22.47)	11.1 (8.45-14.27)	15.99 (12.22-20.39)	9.43 (6.35-12.51)	6.56 (4.98-8.32)	397.08 (298.45-509.79)	247.77 (168.69-331.52)	149.31 (113.39-191.64)
Timor-Leste	0.09 (0.07-0.12)	0.04 (0.03-0.06)	0.05 (0.03-0.07)	0.08 (0.06-0.1)	0.04 (0.03-0.05)	0.04 (0.03-0.06)	2.04 (1.53-2.69)	1 (0.69-1.3)	1.05 (0.76-1.48)
Togo	0.23 (0.16-0.33)	0.12 (0.08-0.17)	0.12 (0.08-0.17)	0.2 (0.13-0.28)	0.1 (0.07-0.14)	0.1 (0.07-0.15)	5.76 (3.78-8.17)	2.91 (1.95-4.16)	2.85 (1.82-4.27)
Tokelau	0 (0-0)	0 (0-0)	0 (0-0)	0 (0-0)	0 (0-0)	0 (0-0)	0 (0-0)	0 (0-0)	0 (0-0)
Tonga	0.01 (0.01-0.01)	0 (0-0)	0 (0-0.01)	0.01 (0-0.01)	0 (0-0)	0 (0-0)	0.13 (0.1-0.17)	0.06 (0.04-0.07)	0.08 (0.06-0.1)
Trinidad and Tobago	0.59 (0.44-0.75)	0.32 (0.24-0.41)	0.27 (0.2-0.34)	0.28 (0.21-0.35)	0.15 (0.11-0.19)	0.13 (0.1-0.16)	6.8 (5.13-8.75)	3.84 (2.87-4.98)	2.96 (2.27-3.8)
Trkiye	1.75 (1.25-2.4)	0.92 (0.64-1.3)	0.83 (0.57-1.14)	0.96 (0.7-1.28)	0.55 (0.39-0.76)	0.41 (0.28-0.56)	23.41 (16.66-32.39)	13.48 (9.36-19.29)	9.93 (6.79-13.94)
Tunisia	0.28 (0.21-0.38)	0.15 (0.11-0.2)	0.13 (0.1-0.18)	0.21 (0.16-0.27)	0.11 (0.08-0.14)	0.1 (0.07-0.13)	6.28 (4.73-8.27)	3.42 (2.59-4.47)	2.85 (2.13-3.81)
Turkmenistan	0 (0-0)	0 (0-0)	0 (0-0)	0 (0-0)	0 (0-0)	0 (0-0)	0.03 (0.02-0.04)	0.01 (0.01-0.02)	0.01 (0.01-0.02)
Tuvalu	21.37 (17.11-26.28)	11.81 (9.07-14.86)	9.56 (7.35-11.89)	11.63 (9.36-14.23)	6.83 (5.31-8.55)	4.81 (3.68-5.99)	283.68 (227.59-349.14)	172.04 (132.11-218.13)	111.64 (85.82-137.78)
Uganda	1.68 (1.27-2.24)	0.89 (0.65-1.2)	0.79 (0.57-1.08)	1.45 (1.11-1.94)	0.76 (0.56-1.03)	0.69 (0.5-0.94)	42.26 (31.03-57.92)	22.93 (16.5-31.59)	19.34 (13.37-26.64)
Ukraine	19.45 (14.56-25.2)	9.55 (6.35-13.76)	9.9 (6.85-14.15)	12.32 (9.19-15.85)	6.24 (4.14-8.77)	6.08 (4.18-8.48)	298.11 (217.35-388.05)	156.54 (103.32-221.7)	141.56 (95.09-201.66)
United Arab Emirates	0.64 (0.46-0.94)	0.35 (0.23-0.56)	0.29 (0.21-0.41)	0.33 (0.24-0.49)	0.19 (0.13-0.31)	0.13 (0.1-0.19)	11.27 (8.02-16.89)	7.04 (4.65-11.43)	4.22 (3.08-6.08)
United Kingdom	50.41 (46.09-52.74)	27.25 (25.53-28.46)	23.16 (20.51-24.6)	22.13 (19.88-23.36)	11.57 (10.75-12.07)	10.56 (9.12-11.34)	415.46 (384.3-434.09)	228.33 (214.42-238.1)	187.13 (168.87-197.94)
United Republic of Tanzania	2.54 (1.88-3.28)	1.35 (0.9-1.8)	1.19 (0.86-1.6)	2.23 (1.65-2.91)	1.18 (0.79-1.58)	1.05 (0.76-1.44)	61.62 (45.05-81.81)	33.16 (22.16-45.67)	28.47 (20.45-39.43)
United States of America	214.11 (197.87-225.19)	115.51 (108.86-120.78)	98.6 (88.1-104.98)	75.09 (68.06-79.71)	39.38 (36.79-41.23)	35.71 (31.05-38.54)	1692.4 (1586.16-1776.26)	942.89 (894.86-983.33)	749.51 (684.02-794.91)
United States Virgin Islands	0.05 (0.04-0.07)	0.03 (0.02-0.04)	0.02 (0.01-0.03)	0.02 (0.02-0.03)	0.01 (0.01-0.02)	0.01 (0.01-0.01)	0.53 (0.4-0.68)	0.32 (0.23-0.43)	0.21 (0.15-0.3)
Uruguay	2.42 (2.14-2.73)	1.24 (1.08-1.4)	1.18 (1.02-1.35)	1.64 (1.44-1.85)	0.81 (0.71-0.91)	0.83 (0.71-0.95)	31.77 (28.21-35.69)	16.73 (14.81-18.75)	15.04 (13.07-17.06)
Uzbekistan	1.78 (1.43-2.19)	0.94 (0.75-1.16)	0.83 (0.66-1.03)	1.27 (1.02-1.56)	0.68 (0.54-0.83)	0.59 (0.47-0.72)	39.16 (31.82-47.76)	21.02 (16.98-25.57)	18.14 (14.51-22.08)
Vanuatu	0.02 (0.01-0.03)	0.01 (0.01-0.01)	0.01 (0.01-0.01)	0.02 (0.01-0.02)	0.01 (0.01-0.01)	0.01 (0.01-0.01)	0.49 (0.36-0.64)	0.26 (0.19-0.36)	0.23 (0.15-0.34)
Venezuela (Bolivarian Republic of Venezuela)	5.43 (3.99-7.07)	3.19 (2.3-4.3)	2.24 (1.59-3.03)	2.99 (2.23-3.87)	1.43 (1.02-1.9)	1.56 (1.12-2.11)	77.06 (56.52-100.62)	38.42 (27.41-51.44)	38.64 (27.62-52.88)
Viet Nam	19.44 (14.78-23.61)	12.09 (9.22-14.74)	7.36 (5.24-9.79)	11.68 (9-14.15)	7.23 (5.46-8.8)	4.45 (3.24-5.85)	315.04 (238.35-387.37)	199.72 (148.34-245.26)	115.32 (82.52-153.51)
Yemen	1.22 (0.79-1.77)	0.56 (0.36-0.84)	0.66 (0.39-1)	0.92 (0.61-1.35)	0.46 (0.3-0.69)	0.47 (0.28-0.71)	27.24 (17.87-40.48)	13.17 (8.38-20)	14.07 (8.12-21.66)
Zambia	1.03 (0.63-1.98)	0.61 (0.37-1.35)	0.41 (0.25-0.77)	0.88 (0.55-1.66)	0.53 (0.32-1.13)	0.36 (0.22-0.65)	26.97 (16.17-54.83)	16.3 (9.65-37.86)	10.67 (6.24-21.1)
Zimbabwe	0.99 (0.75-1.27)	0.42 (0.32-0.54)	0.57 (0.41-0.77)	0.87 (0.67-1.12)	0.37 (0.28-0.47)	0.51 (0.37-0.67)	26.12 (19.47-34.41)	11.35 (8.63-15)	14.77 (10.28-20.33)

DALYs disability-adjusted life-years, UI uncertainty interval

Table.S4 ASIR, ASMR, and ASDR for colorectal cancer across 204 countries in 2021

Location	ASIR/100,000 persons (95%UI)			ASMR/100,000 persons (95%UI)			ASDR/100,000 persons (95%UI)		
	**Both**	**Male**	**Female**	**Both**	**Male**	**Female**	**Both**	**Male**	**Female**
Afghanistan	14.16 (6.79-21.48)	8.82 (5.24-14.5)	18.71 (7.26-29.64)	12.3 (6.2-18.55)	8.24 (5.01-13.34)	15.79 (6.5-24.52)	324.71 (146.82-503.12)	207.4 (119.91-347.45)	425.29 (152.86-694.16)
Albania	11.48 (8.85-15.39)	13.79 (10.27-18.37)	9.43 (6.23-13.94)	7.52 (5.79-9.81)	9.09 (6.74-11.96)	6.17 (4.07-8.93)	156.46 (120.27-209.47)	187.05 (137.25-253.21)	129.06 (84.65-189.01)
Algeria	6.95 (5.39-8.79)	7.07 (5.5-9.08)	6.86 (4.93-9.65)	4.8 (3.78-5.99)	5.19 (3.98-6.67)	4.45 (3.21-6.27)	99.94 (78.4-126.74)	109.21 (84.36-139.91)	90.86 (64.87-128.83)
American Samoa	19.36 (16.14-23.75)	20.65 (16.64-25.88)	18.1 (14.36-23)	15.48 (12.96-18.89)	16.69 (13.32-21.11)	14.35 (11.54-18.31)	366.75 (306.83-450.38)	398.6 (325.12-497.1)	334.95 (264.64-426.75)
Andorra	44.68 (30.29-60.44)	67.63 (45.17-94)	21.61 (14.5-30.44)	15.91 (11.45-21.2)	24.76 (17.36-33.21)	7.58 (5.1-10.75)	344.77 (238.41-466.3)	523.45 (359.92-727.58)	163.5 (110.36-232.5)
Angola	9.04 (6.63-11.83)	11.57 (8.33-15.32)	7.14 (4.67-10.63)	8.53 (6.26-11.21)	10.99 (7.94-14.49)	6.72 (4.36-10.25)	203.35 (148.46-271.46)	257.85 (182.18-343.35)	160.62 (103.64-244.21)
Antigua and Barbuda	32.44 (29.54-35.42)	29.44 (26.1-33.1)	34.89 (31.22-38.94)	14.95 (13.61-16.34)	13.34 (12.01-14.87)	16.08 (14.57-17.52)	320.56 (294.2-346.47)	290.15 (261.45-321.2)	345.2 (312.1-378.35)
Argentina	28.79 (25.21-32.36)	36.03 (31.33-41.18)	23.14 (20.03-26.3)	19.44 (17.08-21.88)	24.54 (21.32-28.04)	15.58 (13.4-17.74)	443.27 (387.89-500.57)	553.77 (481.85-634.41)	353.93 (306.49-406.29)
Armenia	19.55 (17.07-22.33)	23.09 (20.16-26.43)	16.86 (14.65-19.26)	13.12 (11.5-15.07)	15.32 (13.51-17.63)	11.49 (9.99-13.14)	301.97 (265.01-345.93)	362.95 (320.28-417.63)	253.96 (220.09-292.43)
Australia	42.68 (37.58-48.59)	50.47 (45.09-56.85)	35.59 (30.2-41.55)	13.98 (12.19-15.9)	16.78 (14.75-18.97)	11.5 (9.6-13.51)	315.46 (278.06-356.65)	378.52 (338.17-422.21)	257.78 (220.02-300.56)
Austria	29.27 (25.66-33.38)	37.57 (33.04-42.52)	22.5 (19.17-26.2)	11.8 (10.26-13.52)	15.55 (13.78-17.59)	8.91 (7.53-10.4)	253.07 (223.6-287.33)	329.55 (293.5-371.26)	188.62 (161.32-217.98)
Azerbaijan	9.79 (7.42-12.32)	12.09 (8.73-15.78)	8.03 (6-10.47)	7.18 (5.46-8.98)	9.01 (6.57-11.74)	5.84 (4.45-7.51)	184.5 (138.24-234.39)	227.61 (161.4-296)	149.74 (110.54-194.37)
Bahamas	36.11 (28.86-44.63)	44.14 (34.43-55.2)	29.54 (23.66-36.21)	18.47 (15.15-22.54)	22.34 (17.8-27.55)	15.37 (12.5-18.5)	444.71 (357.74-553.53)	546.36 (428.99-687.74)	359.09 (285.78-440.51)
Bahrain	18.79 (15.12-25.02)	20.8 (16.53-28.5)	17.75 (13.2-23.5)	10.98 (8.94-14.46)	13.27 (10.68-17.69)	8.99 (6.76-11.84)	227.1 (183.24-307.76)	262.11 (207.8-364)	193.83 (146.49-259.36)
Bangladesh	4.41 (3.29-6.03)	4.77 (3.46-6.76)	3.99 (2.77-5.75)	3.58 (2.71-4.89)	3.94 (2.86-5.5)	3.17 (2.22-4.64)	88.88 (65.03-124.89)	94.76 (67.73-138.81)	81.98 (57.88-121.22)
Barbados	50.65 (39.74-62.59)	55.73 (41.91-69.99)	46.33 (36.28-58.02)	22.93 (18.1-27.93)	24.59 (18.7-30.8)	21.45 (17.12-26.15)	496.33 (387.48-616.82)	547.99 (410.94-691.49)	451.06 (356.18-563.52)
Belarus	36.09 (27.38-45.99)	47.64 (36.29-60.05)	29.59 (22.37-37.86)	17.32 (13.2-21.73)	24.2 (18.87-29.83)	13.77 (10.42-17.59)	415.9 (314.4-530.09)	555.24 (424.16-693.77)	333.18 (250.19-427.29)
Belgium	36.35 (31.83-40.73)	44.33 (38.3-49.8)	29.64 (25.47-34.26)	14.13 (12.2-15.98)	17.6 (15.35-19.85)	11.37 (9.58-13.05)	301.55 (264.48-338.67)	370.53 (324.05-420.39)	242.15 (210.97-274.98)
Belize	16.66 (14.53-19.41)	16.97 (14.14-20.29)	16.29 (14.1-18.94)	9.2 (7.95-10.64)	9.29 (7.77-11.01)	9.08 (7.87-10.58)	218.78 (188.42-255.02)	224.79 (186.27-268.2)	212.26 (185.02-246.16)
Benin	5.85 (4.47-7.48)	6.71 (5.17-8.78)	5.1 (3.75-6.84)	5.66 (4.39-7.17)	6.46 (5.01-8.39)	4.97 (3.69-6.63)	123.35 (93.2-162.85)	142.18 (107.92-186.73)	106.72 (76.05-149.41)
Bermuda	61.79 (51.46-77.11)	76.5 (62.62-94.01)	49.4 (40.16-63.15)	18.37 (15.27-22.59)	21.94 (18.04-26.81)	15.43 (12.48-19.52)	396.54 (329.53-490.21)	487.18 (397.05-599.12)	317.89 (259.98-404.39)
Bhutan	5.26 (3.97-6.84)	5.54 (3.39-7.53)	5 (3.55-6.7)	4.41 (3.31-5.68)	4.73 (2.91-6.42)	4.1 (2.95-5.49)	106.36 (78.43-138.93)	110.28 (66.66-151.51)	102.83 (73.06-139.33)
Bolivia (Plurinational State of Bolivia)	16.31 (11.17-22.63)	14.38 (10.03-19.83)	17.97 (10.41-26.86)	14.04 (9.59-19.38)	12.13 (8.5-16.83)	15.65 (9.03-23.21)	308.93 (211.81-431.97)	270.37 (187.82-370.8)	343.09 (200.86-517.53)
Bosnia and Herzegovina	30.79 (23.78-38.32)	41.38 (30.48-54.03)	22.37 (17.06-28.04)	18.9 (14.84-23.39)	25.39 (18.85-32.94)	13.91 (10.63-17.6)	423.43 (323.22-528.91)	567.99 (413.83-751.09)	305.39 (233.13-380.54)
Botswana	11.05 (8.43-14.95)	14 (10.12-18.46)	8.93 (6.14-12.96)	10.08 (7.76-13.38)	12.75 (9.25-16.7)	8.21 (5.77-11.8)	226.73 (167.8-313.81)	288.31 (203.79-391.74)	179.88 (119.67-274.21)
Brazil	17.23 (15.86-18.39)	19.56 (18.1-20.9)	15.36 (13.84-16.49)	11.6 (10.6-12.37)	12.95 (11.9-13.83)	10.54 (9.41-11.34)	287.32 (267.76-303.88)	317.43 (296.21-337.51)	262.68 (241.06-280.79)
Brunei Darussalam	30.04 (25.37-35.53)	36.4 (29.93-43.26)	25.25 (20.02-32.38)	20.16 (17.04-23.91)	24.82 (20.33-29.79)	17.22 (13.63-22.02)	466.43 (393.67-547.69)	552.06 (452.5-659.82)	396.74 (310.76-508.73)
Bulgaria	46.04 (37.65-55.79)	63.03 (51.31-77.09)	33.5 (27.13-40.2)	25.71 (20.96-30.73)	36.26 (29.79-43.37)	18.4 (14.88-22.05)	605 (493.22-726.67)	827.31 (677.68-999.32)	434.37 (353.85-522.13)

Burkina Faso	6.26 (4.71-7.98)	6.77 (4.96-9.2)	5.76 (4.14-7.53)	6.17 (4.7-7.85)	6.6 (4.86-8.97)	5.73 (4.07-7.48)	134.58 (99.55-173.68)	147.34 (105.54-203.55)	122.48 (84.45-161.67)
Burundi	8.43 (6.15-11.61)	9.46 (6.46-14.13)	7.36 (5.29-10.16)	8.33 (6.11-11.45)	9.4 (6.51-13.87)	7.26 (5.29-9.97)	193.62 (138.14-275.06)	216.46 (149.37-330.4)	169.2 (119.66-236.03)
Cabo Verde	10.83 (8.47-13.09)	13.95 (9.88-17.44)	8.52 (6.58-10.52)	9.12 (7.01-10.81)	11.36 (7.79-14.09)	7.46 (5.69-9.1)	179.56 (140.03-216)	236.63 (169.53-296.77)	136.38 (105.72-167.98)
Cambodia	16.99 (12.67-21.96)	20.89 (15.47-27.44)	14.35 (9.97-19.45)	14.37 (10.8-18.37)	17.9 (13.35-23.35)	12.07 (8.35-16.08)	353.09 (262.55-456.87)	435.01 (315.44-582.44)	294.64 (203.49-401.06)
Cameroon	7.91 (5.68-10.58)	9.13 (6.35-12.6)	6.75 (4.86-9.44)	7.48 (5.44-9.94)	8.58 (6.07-11.88)	6.44 (4.65-8.94)	167.2 (116.22-226.65)	194.6 (132.52-271.34)	141.14 (97.95-200.68)
Canada	43.29 (38.58-47.88)	52.81 (47.69-58.1)	34.7 (30.17-39.52)	14.15 (12.47-15.8)	17.01 (15.26-18.75)	11.69 (10.01-13.43)	317.47 (285.08-351.22)	386.7 (351.53-425.49)	254.6 (225.02-285.27)
Central African Republic	9.11 (6.39-12.71)	10.82 (6.89-16.37)	7.81 (4.83-12.68)	9.12 (6.4-12.86)	10.82 (6.92-16.05)	7.85 (4.95-12.63)	229.71 (154.29-327.17)	273.22 (172.43-414.24)	194.54 (117.72-323.04)
Chad	6.32 (4.73-8.2)	6.48 (4.63-8.44)	6.05 (4.22-8.32)	6.29 (4.79-8.11)	6.42 (4.68-8.39)	6.05 (4.3-8.36)	142.86 (106.23-186.72)	147.21 (106.35-193.16)	136.26 (94.61-187.67)
Chile	24.09 (21.07-27.25)	27.5 (24.28-31.12)	21.33 (18.49-24.44)	13.13 (11.54-14.8)	14.99 (13.27-16.72)	11.66 (9.96-13.3)	292.24 (258.58-329.15)	335.84 (297.73-377.35)	256.04 (224.39-292.11)
China	31.44 (25.53-37.97)	42.24 (32.56-54.26)	21.87 (16.58-27.92)	13.64 (11.09-16.31)	18.95 (14.65-24.34)	9.34 (7.1-11.88)	331.73 (267.78-400.7)	452.83 (349.19-585.28)	220.01 (167.51-282.02)
Colombia	22.28 (18.37-27.26)	29.08 (23.58-36.03)	16.73 (13.83-19.94)	10.49 (8.66-12.68)	10.92 (8.8-13.52)	10.14 (8.38-12.07)	258.01 (212.77-310.81)	270.05 (216.04-332.88)	248.17 (206.24-294.74)
Comoros	10.56 (7.55-13.96)	11.83 (7.69-16.44)	9.53 (6.36-13.28)	10.04 (7.17-13.4)	11.37 (7.43-15.57)	8.98 (5.96-12.61)	230.84 (164.12-314.31)	255.67 (165.93-365.21)	210.44 (136.62-302.32)
Congo	10.97 (8.68-13.79)	12.51 (9.65-16.18)	9.68 (6.94-12.9)	10.1 (8.03-12.53)	11.65 (9.03-14.96)	8.81 (6.37-11.43)	242.56 (187.26-310.42)	272.59 (209.85-351.16)	216.49 (151.83-297.06)
Cook Islands	9.43 (7.53-11.93)	9.48 (7.55-11.85)	9.37 (6.76-12.78)	5.68 (4.59-7.09)	5.75 (4.66-7.07)	5.61 (4.13-7.56)	129.05 (102.56-164.07)	133.77 (106.36-167.99)	124.46 (91.36-171.12)
Costa Rica	35.99 (31.18-42.31)	50.15 (42.87-58.63)	23.93 (20.68-28.02)	15.13 (13.09-17.48)	16.83 (14.53-19.57)	13.67 (11.63-15.99)	370.71 (321.5-430.24)	425.08 (366.81-494.22)	323.43 (275.54-378.96)
Croatia	49.75 (42.4-57.25)	67.22 (56.14-78.24)	36.52 (30.52-42.67)	24.29 (20.78-27.97)	34.82 (29.42-40.82)	16.85 (14.37-19.5)	524.86 (450.88-601.57)	736.52 (623.87-854.11)	359.59 (298.56-422.33)
Cuba	48.47 (40.77-56.59)	46.13 (37.61-54.56)	50.59 (42.21-60.13)	17.47 (14.89-20.22)	16.5 (13.81-19.4)	18.26 (15.36-21.38)	388.5 (327.7-454.12)	368.45 (306.07-437.37)	406.33 (339.08-479.98)
Cyprus	34.23 (28.58-40.59)	42.02 (34.5-51.09)	27.38 (21.17-34.09)	12.75 (10.76-14.93)	15.86 (13.21-19)	10.19 (8.23-12.57)	247.79 (208.91-294.36)	310.51 (256.14-377.27)	192.42 (152.9-240.05)
Czechia	40.24 (33.58-47.83)	56.05 (46.71-66.94)	27.76 (22.94-33.08)	20.16 (16.98-23.84)	28.32 (24.05-33.55)	14.07 (11.79-16.67)	446.39 (371.66-531.64)	616.28 (515.79-734.17)	306.79 (254.43-363.6)
Côte d'Ivoire	4.31 (3.27-5.89)	4.21 (3.13-6.1)	4.4 (3.24-5.92)	4 (3.06-5.39)	3.9 (2.93-5.54)	4.08 (3.06-5.43)	92.14 (67.87-128.91)	90.58 (65.76-135.28)	93.48 (67.09-126.72)
Democratic People's Republic of Korea	15.43 (10.42-22.53)	19 (12.95-29.46)	12.62 (7.77-19.89)	10.51 (7.05-15.44)	13.13 (8.94-20.04)	8.63 (5.31-13.58)	283 (189.17-427.86)	353.96 (239.17-555.8)	223.55 (137.58-362.51)
Democratic Republic of the Congo	6.9 (4.78-9.92)	7.75 (4.91-12.92)	6.29 (4.09-9.22)	6.59 (4.52-9.67)	7.46 (4.75-12.8)	5.99 (3.88-8.93)	156.42 (107.84-227.19)	174.39 (111.56-293.06)	142.79 (92.07-210.83)
Denmark	43.45 (37.3-49.33)	50.29 (43.13-57.14)	37.3 (31.93-42.72)	18.97 (16.41-21.34)	22.21 (19.04-25.52)	16.19 (13.86-18.27)	384.64 (333.8-432.46)	451.72 (394.89-508.17)	323.96 (279.08-366.28)
Djibouti	13.5 (9.66-18.59)	16.14 (10.78-22.55)	10.68 (7.18-15.58)	12.66 (9.13-17.12)	15.27 (10.27-21.42)	9.98 (6.82-14.52)	290.23 (205.38-408.8)	345.76 (227.76-502.54)	227.77 (149.45-333.81)
Dominica	24.62 (20.79-29.66)	27.66 (22.19-34.53)	21.71 (17.34-27.04)	15.18 (12.81-17.99)	16.77 (13.54-20.7)	13.63 (10.91-17.04)	331.61 (278.95-402.29)	374.96 (297.66-467.56)	287.22 (229.48-359.19)
Dominican Republic	15.45 (12.13-19.74)	17.25 (12.28-23.19)	13.73 (10.08-18.33)	9.14 (7.1-11.73)	10.02 (7.22-13.68)	8.28 (6.02-11.09)	215.86 (168.9-277.61)	243.07 (174.16-325.26)	189.58 (140.39-250.96)
Ecuador	13.04 (10.17-16.5)	13.16 (9.83-17.25)	12.91 (10-16.57)	9.46 (7.42-11.88)	9.36 (7.08-12.13)	9.53 (7.43-12.08)	215.97 (166.95-275.35)	215.87 (162.97-282.01)	215.71 (166.1-278.88)
Egypt	12.57 (10.41-15.33)	11.66 (9.36-14.37)	14.29 (11.42-17.68)	8.78 (7.29-10.58)	8.56 (6.94-10.44)	9.84 (7.89-12.2)	212.77 (174.34-258.8)	215.76 (172.26-266.19)	217.57 (175.44-272.82)
El Salvador	14.81 (12.01-18.31)	19.29 (14.8-24.1)	11.53 (9.06-14.35)	8.11 (6.54-9.9)	8.38 (6.46-10.26)	7.9 (6.23-9.74)	206.97 (165.35-253.14)	229.98 (175.25-286.26)	189.62 (147.91-234.37)
Equatorial Guinea	12.25 (8.33-17.07)	16.48 (11.17-22.8)	9.22 (5.4-14.75)	10.49 (7.24-14.52)	14.27 (9.6-19.71)	7.82 (4.67-12.53)	245.07 (165.22-345.96)	327.31 (216.93-459.63)	184.15 (107.19-300.55)

Eritrea	12.11 (9.05-15.78)	14.03 (9.37-20.22)	10.87 (7.77-14.55)	11.82 (8.89-15.28)	13.74 (9.14-19.93)	10.63 (7.6-14.05)	282.31 (208.73-376.44)	327.68 (217.7-481.24)	250.33 (176.69-337.87)
Estonia	40.93 (33.39-48.65)	56.92 (46.65-65.98)	31.44 (24.79-38.5)	17.8 (14.52-20.87)	26.4 (21.81-30.65)	13.15 (10.5-15.64)	382.11 (310.79-447)	544.93 (448.87-630.34)	281.26 (222.76-335.28)
Eswatini	16.74 (10.96-22.46)	22.46 (13.97-30.79)	12.89 (7.76-18.41)	15.24 (10.05-20.14)	20.22 (12.58-27.64)	11.97 (7.31-16.8)	378.16 (242.58-517.06)	517.34 (322.23-717.46)	278.85 (165.02-415.28)
Ethiopia	16.41 (13.7-19.54)	22.1 (17.41-28.21)	10.55 (8.57-13.24)	15.91 (13.33-19.01)	21.57 (17.13-27.41)	10.09 (8.18-12.63)	343.04 (284.97-415.16)	460.17 (360.14-593.93)	222.64 (178.44-281.59)
Fiji	11.22 (8.35-14.5)	13.49 (7.84-18)	9.74 (7.36-12.91)	9.72 (7.29-12.58)	12.15 (7.06-16.05)	8.29 (6.31-10.94)	226.96 (167.3-295.51)	268.52 (157.25-358.87)	196.26 (147.26-261.46)
Finland	30.63 (26.84-34.28)	36.66 (31.97-41.54)	25.46 (21.75-28.89)	11.32 (9.78-12.77)	13.69 (11.84-15.47)	9.33 (7.86-10.57)	243.95 (215.21-273.85)	295.81 (261.21-333.3)	198.17 (171.81-223.07)
France	41.8 (36.04-47.25)	51.86 (45.14-58.62)	33.56 (28.54-38.34)	15.12 (12.88-17.1)	19.53 (16.78-21.98)	11.74 (9.66-13.47)	324.49 (282.4-367.06)	413.7 (361.77-465.98)	250.43 (215.28-283.46)
Gabon	15.45 (11.31-20.01)	20.79 (14.89-26.64)	11.03 (7.48-15.53)	13.54 (9.99-17.31)	18.41 (13.38-23.65)	9.72 (6.67-13.51)	319.35 (230.62-421.91)	427.9 (302.53-555.08)	224.23 (151.29-316.02)
Gambia	3.31 (2.54-4.27)	3.13 (2.27-4.29)	3.46 (2.52-4.64)	3.05 (2.34-3.93)	2.9 (2.11-3.95)	3.17 (2.31-4.3)	70.75 (53.17-92.31)	67.08 (48.02-93.29)	74.2 (52.82-101.71)
Georgia	18.73 (16.08-21.69)	26.1 (22.38-30.21)	13.54 (11.56-15.83)	13.3 (11.42-15.29)	18.69 (16.02-21.42)	9.68 (8.36-11.18)	328.78 (281.26-381.66)	458.56 (392.93-529.27)	231.99 (197.73-269.83)
Germany	38.53 (33.72-43.31)	47.82 (41.89-53.94)	30.49 (26.04-34.84)	14.63 (12.49-16.47)	18.39 (16.08-20.71)	11.53 (9.65-13.22)	323.01 (286.33-360.79)	405.24 (360.96-453.44)	250.21 (217.37-284.67)
Ghana	7.41 (5.81-9.21)	7.52 (5.88-9.39)	7.3 (5.49-9.5)	6.89 (5.39-8.51)	6.97 (5.47-8.64)	6.78 (5.15-8.72)	150.82 (117.21-189.48)	153.34 (117.91-193.95)	148.93 (110.44-196.78)
Greece	33.38 (29.96-37.16)	42.01 (37.4-47.12)	26.12 (22.99-29.33)	14.03 (12.29-15.52)	17.73 (15.79-19.72)	11.05 (9.56-12.36)	297.27 (268.42-326.68)	376.77 (342.66-416.53)	229.5 (204.75-254.41)
Greenland	38.22 (32.36-45.88)	41.4 (34.45-50.41)	34.64 (26.58-43.61)	24.6 (20.85-29.54)	26.82 (22.33-32.87)	22.25 (17.14-28.15)	572.67 (484.22-684)	616.76 (511.12-749.85)	522.07 (406.51-659.86)
Grenada	31.36 (26.48-36.76)	36.46 (30-43.89)	27.85 (24.04-32.38)	17.32 (14.67-20.21)	20.33 (16.9-23.94)	15.42 (13.28-17.77)	387.52 (324.2-453.25)	446.9 (364.71-533.17)	341.53 (293.44-394.6)
Guam	16.97 (14.62-19.62)	22.02 (18.75-25.83)	12.4 (9.7-14.85)	10.23 (8.77-11.85)	13.77 (11.65-16.08)	7.19 (5.53-8.64)	291.86 (253.74-332.83)	382.13 (326.16-445.23)	206.8 (165.79-244.89)
Guatemala	9.79 (8.42-11.48)	11.25 (9.48-13.45)	8.5 (7.3-9.91)	6.93 (5.98-8.05)	6.78 (5.75-8.08)	7.04 (6.07-8.15)	168.37 (143.72-196.84)	170.6 (143.87-203)	166.32 (141.89-193.47)
Guinea	4.85 (3.63-6.48)	5.85 (4.41-8.2)	3.81 (2.77-5.31)	4.69 (3.52-6.25)	5.69 (4.28-7.9)	3.66 (2.69-5.06)	110.34 (80.27-151.15)	132.34 (97-188.62)	87.66 (62.41-126.24)
Guinea-Bissau	8.48 (6.36-10.64)	9.37 (6.97-12.11)	7.69 (5.4-10.28)	8.39 (6.31-10.47)	9.21 (6.96-11.88)	7.65 (5.48-10.14)	195.76 (146.32-248.83)	217.65 (162.26-288.58)	176.45 (122.58-239.07)
Guyana	19.48 (14.82-25.11)	21.85 (16.57-28.84)	17.34 (13.08-22.28)	13.25 (10.17-16.9)	14.59 (11.06-19)	12.02 (9.19-15.13)	325.81 (246.26-422.11)	372.06 (277.9-487.91)	283.49 (212.43-361.67)
Haiti	16.53 (11.29-23.36)	14.47 (9.84-20.97)	18.43 (10.25-28.71)	13.83 (9.56-19.25)	12.09 (8.26-17.58)	15.44 (8.87-23.74)	325.88 (222.11-464.01)	285.34 (193.68-421.41)	363.13 (201.96-562.18)
Honduras	8.32 (6.62-10.76)	8.54 (6.56-11.57)	8.12 (5.92-11.27)	6.27 (4.99-8.15)	5.52 (4.26-7.38)	6.92 (5.01-9.49)	147.97 (116.1-194.46)	127.88 (98.74-172.38)	165.47 (120.7-228.62)
Hungary	49.51 (40.97-59.24)	71.34 (59.32-84.94)	33.69 (28.19-40.01)	26.01 (21.73-31.13)	37.82 (31.67-45.29)	17.97 (14.98-21.45)	614.96 (519.37-736.2)	879.42 (738.27-1052.23)	417.05 (348.55-498.94)
Iceland	32.67 (28.14-38.09)	35.99 (30.71-42.37)	29.58 (25.4-34.52)	11.19 (9.38-13.03)	12.57 (10.62-14.73)	9.92 (8.16-11.57)	239.6 (207.01-275.8)	272.58 (232.44-316.94)	207.97 (179.44-241.74)
India	5.69 (5.05-6.45)	6.06 (5.03-7.11)	5.4 (4.53-6.4)	4.6 (4.09-5.21)	4.97 (4.14-5.84)	4.31 (3.63-5.12)	120.93 (107.62-137.15)	125.21 (103.1-148.27)	117.75 (98.71-140.18)
Indonesia	15.03 (11.4-18.74)	17.17 (11.84-23.03)	13.1 (9.12-18.02)	12.39 (9.5-15.3)	14.29 (10.01-18.98)	10.72 (7.54-14.63)	297.58 (223.31-371.79)	341.03 (235.31-456.29)	257.25 (177.74-355.81)
Iran (Islamic Republic of Iran)	13.19 (11.44-14.76)	13.3 (9.96-15.21)	13.13 (11.4-14.86)	7.37 (6.31-8.19)	8.08 (6.17-9.23)	6.68 (5.83-7.6)	174.3 (149.14-193.93)	193.97 (150.31-221.52)	155.07 (135.39-176.15)
Iraq	10.04 (7.6-12.68)	11.51 (8.34-14.73)	8.83 (6.77-11.64)	6.33 (4.84-7.83)	7.91 (5.73-9.99)	4.93 (3.82-6.28)	154.27 (116.39-193.92)	187.48 (135.95-240.46)	123.47 (92.44-161.76)
Ireland	40.89 (35.16-46.57)	52.88 (45.23-60.81)	30.15 (25.17-35.01)	14.38 (12.21-16.4)	19.01 (16.12-21.95)	10.46 (8.54-12.02)	308.17 (267.9-349.96)	402.55 (348.06-464.88)	222.78 (188.59-255.65)
Israel	29.81 (25.05-34.37)	35.25 (29.65-41.06)	25.15 (20.84-28.89)	13.35 (10.94-15.48)	15.74 (13.12-18.36)	11.34 (9.14-13.09)	270.88 (231-310.26)	323.73 (274.58-371.99)	224.78 (189.6-256.7)

Italy	39.65 (35.64-42.66)	50.3 (46.16-53.9)	30.89 (26.9-33.83)	14.28 (12.57-15.43)	18.65 (16.99-19.87)	10.95 (9.21-12.09)	309.66 (284.37-331.08)	395.45 (366.92-419.21)	238.36 (211.97-258.91)
Jamaica	31.7 (23.99-42.39)	35.1 (25.56-48.49)	28.46 (21.74-36.54)	15.09 (11.62-19.81)	16.59 (12.21-22.45)	13.62 (10.67-17.25)	353.13 (266.97-471.23)	390.19 (283.39-531.8)	317.08 (243.76-409.41)
Japan	48.7 (44.07-51.75)	63.96 (59.31-67.26)	35.3 (30.02-38.58)	15.89 (13.97-16.97)	20.24 (18.75-21.17)	12.17 (9.93-13.42)	358.44 (327.93-378.99)	457.21 (430.95-478.23)	269.77 (236.36-289.62)
Jordan	15.51 (11.84-20.96)	16.04 (12.17-21.28)	15.04 (10.81-22.2)	8.78 (6.7-11.64)	9.76 (7.43-12.98)	7.75 (5.57-11.41)	205.95 (158.78-277.99)	231.74 (175.27-308.57)	177.56 (127.39-263.6)
Kazakhstan	15.43 (13.06-17.98)	19.73 (16.8-22.81)	12.75 (10.59-14.85)	10.53 (8.9-12.23)	13.68 (11.67-15.88)	8.66 (7.21-10.11)	261.66 (224.14-303.16)	331.89 (283.75-384.71)	214.84 (180.1-250.85)
Kenya	7.89 (6.48-9.79)	7.66 (5.98-9.88)	8.05 (6.02-10.65)	7.18 (5.91-8.86)	6.97 (5.43-8.95)	7.31 (5.48-9.66)	171.46 (141.02-215.33)	166.89 (129.12-218.1)	175.41 (129.24-236.43)
Kiribati	9.43 (7.43-12.48)	11.8 (8.8-16.8)	7.74 (5.83-10.13)	9 (7.25-11.87)	11.33 (8.55-16.01)	7.43 (5.62-9.51)	223.53 (170.29-299.18)	283.19 (208.59-404.26)	177.68 (131.49-236.77)
Kuwait	19.05 (15.4-23.23)	21.54 (16.71-27.43)	15.39 (12.61-18.43)	8.67 (7.06-10.72)	10.61 (8.37-13.54)	5.94 (4.79-7.1)	194.76 (155.3-241.81)	236.76 (184.23-302.92)	136.68 (113.14-162.55)
Kyrgyzstan	9.61 (7.84-11.52)	11.21 (9.01-13.66)	8.48 (6.99-10.16)	7.03 (5.66-8.56)	8.33 (6.7-10.24)	6.17 (5.05-7.45)	181.61 (148.95-218.19)	211.98 (172.02-257.36)	158.59 (130.28-190.33)
Lao People's Democratic Republic	14.7 (10.62-19.11)	16.6 (11.69-22.59)	12.99 (8.74-17.43)	13.03 (9.55-16.89)	14.85 (10.49-20.24)	11.43 (7.93-15.15)	323.48 (229.5-429.29)	366.09 (254.21-503.46)	284 (187.79-383.36)
Latvia	30.02 (24.5-35.79)	40.23 (32.77-47.14)	24.02 (19.03-29.47)	16.66 (13.6-19.9)	23.53 (19.08-27.87)	12.96 (10.41-15.9)	380.02 (310.97-453.77)	519.3 (424.08-607.77)	293.73 (233.23-362.45)
Lebanon	20.3 (16.48-25.89)	24.95 (19.9-32.1)	16.39 (12.54-22.69)	10.99 (8.98-14.02)	14.57 (11.63-18.9)	7.96 (5.96-11.21)	236.4 (193.55-298.75)	313.23 (249.76-404.74)	171.38 (130.11-238.19)
Lesotho	14.06 (10.03-19.74)	16.58 (11.63-23.07)	12.3 (7.99-18.86)	13.47 (9.64-19.03)	15.64 (11.01-21.78)	11.91 (7.8-17.82)	328.58 (228.45-473.52)	397.5 (271.64-563.39)	279.93 (174.9-441.37)
Liberia	5.94 (3.78-8.87)	5.89 (3.4-9.8)	6.01 (3.6-8.87)	5.58 (3.6-8.29)	5.51 (3.2-9.11)	5.65 (3.44-8.27)	124.19 (77.79-186.56)	123.25 (69.92-206.64)	125.42 (74.71-186.95)
Libya	21.49 (15.65-28.68)	22.27 (15.46-30.43)	20.82 (14.96-28.41)	13.95 (10.27-18.59)	15.77 (10.84-21.17)	12.16 (8.7-16.75)	332.66 (242.52-449.39)	368.09 (257.83-503.15)	297.6 (211.43-412.75)
Lithuania	30.85 (25.55-36.25)	44.12 (36.63-51.96)	22.91 (18.64-27.3)	16.92 (13.9-19.95)	24.44 (20.12-28.91)	12.8 (10.42-15.27)	380.34 (315.77-449.63)	532.92 (441.29-628.65)	285.25 (233.73-344.57)
Luxembourg	37.51 (33.2-42.15)	46.35 (40.57-52.06)	29.65 (25.54-33.9)	14.62 (12.82-16.25)	18.31 (16.08-20.4)	11.63 (9.82-13.28)	299.97 (264.08-334.57)	375.19 (328.22-419.81)	231.81 (200.22-262.47)
Madagascar	8.48 (6.16-10.94)	8.94 (6.1-12.16)	8.1 (5.91-10.9)	8.12 (5.92-10.46)	8.67 (5.95-11.79)	7.67 (5.62-10.15)	193.53 (139.73-252.26)	201.14 (137.03-277.43)	187.05 (135.41-251.09)
Malawi	4.84 (3.81-6.5)	5.44 (4.12-7.53)	4.36 (3.17-6.23)	4.55 (3.57-6.08)	5.11 (3.89-7.04)	4.11 (3.02-5.84)	109.53 (85.08-150.62)	123.89 (91.88-176.35)	97.69 (67.76-143.46)
Malaysia	26.12 (22.65-29.53)	29.95 (24.38-35.36)	22.44 (18.82-26.58)	18.01 (15.58-20.27)	20.75 (16.76-24.35)	15.44 (12.89-18.57)	418.71 (364.59-469.07)	485.89 (398.72-572.38)	353.47 (298.26-417.11)
Maldives	6.59 (5.32-8.14)	7.11 (5.31-9.04)	5.97 (4.6-7.75)	4.37 (3.53-5.34)	4.81 (3.64-6.05)	3.87 (3-4.99)	94.22 (76.2-117.52)	103.05 (77.28-130.62)	83.28 (64.21-109.36)
Mali	7.43 (5.79-9.3)	8.96 (6.66-11.77)	5.8 (4.38-7.86)	7.1 (5.55-8.96)	8.63 (6.45-11.3)	5.46 (4.17-7.37)	164.24 (125.81-208.77)	195.5 (143.11-257.26)	130.98 (96.72-182.53)
Malta	34.01 (28.75-38.87)	35.93 (30.69-41.74)	32.36 (26.76-37.6)	13.62 (11.31-15.67)	15.21 (13.01-17.68)	12.26 (9.94-14.2)	292.12 (249.33-335.41)	324.01 (277.02-376.24)	263.37 (221.13-303.74)
Marshall Islands	13.31 (9.85-17.4)	14.01 (10.08-19.36)	12.61 (8.11-17.02)	12.01 (9.12-15.47)	12.7 (9.22-17.48)	11.32 (7.49-15.19)	299.61 (218.73-393.72)	319.56 (228.2-453.47)	279.29 (173.96-383.48)
Mauritania	7.93 (5.79-10.59)	7.76 (5.26-10.99)	8.09 (5.71-10.67)	7.09 (5.18-9.5)	6.96 (4.77-9.86)	7.22 (5.1-9.55)	152.11 (111.06-206.82)	148.35 (99.79-215.26)	155.33 (108.39-207.81)
Mauritius	21.62 (19.58-23.31)	26.97 (24.45-29.15)	16.98 (15.14-18.54)	14.25 (12.99-15.31)	17.9 (16.31-19.27)	11.22 (10.06-12.22)	354.37 (324.38-380.39)	449.1 (408.6-484.11)	270.09 (242.96-295.06)
Mexico	16.16 (14.2-18.2)	22.11 (18.33-26.17)	11 (9.31-12.72)	8.78 (7.77-9.81)	10.08 (8.44-11.88)	7.66 (6.53-8.78)	223.11 (196.93-250.75)	261.23 (218.08-308.49)	189.43 (159.63-220.81)
Micronesia (Federated States of Micronesia)	14.14 (10.28-19.12)	14.79 (10.1-20.96)	13.47 (9.07-18.85)	12.42 (9-16.65)	13.04 (9.07-18.45)	11.81 (8.02-16.46)	303.82 (219.46-415.67)	325.01 (223.91-465.79)	282.51 (186.5-399.98)
Monaco	68.33 (54.05-83.19)	86.6 (69.96-106.48)	52.38 (36.95-67.44)	25.23 (20.13-30.6)	32.6 (25.95-39.76)	19.15 (13.91-24.86)	551.88 (440.65-676.5)	710.38 (568.02-876.19)	411.26 (293.82-537.46)
Mongolia	10.01 (7.82-12.45)	11.69 (8.11-15.82)	8.77 (6.19-11.52)	8.12 (6.35-10.04)	9.51 (6.63-12.67)	7.11 (4.96-9.23)	207.23 (162.14-256.08)	243.39 (169.18-327.91)	179.09 (126.92-234.19)

Montenegro	31.46 (26.15-38.3)	43.7 (34.63-54.36)	21.9 (16.9-28.23)	17.74 (14.86-20.97)	24.7 (19.91-30.24)	12.64 (9.68-15.82)	378.8 (314.03-462.92)	523.66 (413.83-652.21)	262.13 (199.52-333.36)
Morocco	11.56 (8.38-15.19)	10.64 (7.52-14.49)	12.5 (7.99-18.38)	8.33 (6.2-10.64)	8.49 (6.02-11.49)	8.2 (5.43-11.77)	200.91 (146.47-263.3)	192.39 (134.92-259.11)	209.66 (130.8-310.46)
Mozambique	4.08 (3.11-5.07)	4.96 (3.72-6.15)	3.39 (2.36-4.54)	4.15 (3.15-5.18)	4.98 (3.78-6.18)	3.51 (2.44-4.74)	86.42 (64.64-109.87)	107.75 (79.82-135.66)	69.35 (46.82-94.34)
Myanmar	13.71 (10.09-17.58)	16.33 (11.72-21.56)	11.77 (8.07-15.76)	11.56 (8.58-14.83)	13.92 (10.03-18.28)	9.86 (6.76-13.18)	286.48 (209.56-372.14)	341.86 (245.75-451.56)	244.33 (166.33-329.79)
Namibia	7.4 (5.5-9.48)	9.46 (7.32-11.77)	5.96 (4.04-8.06)	6.37 (4.83-8.03)	8.14 (6.43-9.98)	5.16 (3.58-6.86)	157.04 (113.73-204.68)	202.07 (153.23-255.38)	123.64 (82.02-170.41)
Nauru	19.8 (12.26-26.38)	22.47 (13.5-30.53)	17.61 (10.53-24.36)	16.98 (10.74-22.3)	19.33 (11.86-26)	15.09 (9.08-20.71)	428.75 (258.39-574.72)	495.18 (283.39-675.3)	372.71 (218.35-527.29)
Nepal	4.85 (3.61-6.39)	5.07 (3.42-7.61)	4.64 (3.27-6.32)	4.14 (3.09-5.45)	4.42 (3-6.67)	3.9 (2.74-5.3)	103.55 (76.75-136.66)	106.3 (71.89-158.87)	100.86 (70.09-137.9)
Netherlands	69.8 (62.21-76.79)	85.8 (77.64-93.96)	56.02 (48.19-63.69)	19.99 (17.63-22.18)	24.78 (22.19-27.53)	16.14 (13.8-18.43)	444.07 (399.16-488.14)	539.87 (491.24-593.38)	359.57 (312.82-405.22)
New Zealand	51.08 (44.46-58.12)	59.88 (52.81-67.74)	43.13 (36.42-50.42)	18.15 (15.68-20.59)	21.58 (18.9-24.27)	15.2 (12.78-17.63)	390.26 (342.89-440.26)	462.28 (413.09-514.7)	325.21 (279.16-375.27)
Nicaragua	10.08 (8.17-12.28)	12.48 (9.6-15.87)	8.1 (6.24-10.36)	5.89 (4.81-7.11)	5.96 (4.67-7.58)	5.8 (4.42-7.34)	145.04 (116.78-176.92)	150.53 (116.4-191.67)	140.06 (106.76-178.3)
Niger	5.06 (3.63-6.78)	4.92 (3.37-7.17)	5.17 (3.34-7.46)	5.05 (3.66-6.74)	4.9 (3.37-7.13)	5.17 (3.38-7.45)	110.28 (78.25-149.79)	107.2 (71.84-160.38)	112.66 (72.49-162.31)
Nigeria	6.08 (4.88-7.47)	6.74 (4.83-8.9)	5.51 (3.97-7.49)	5.78 (4.76-6.96)	6.41 (4.7-8.41)	5.22 (3.88-6.96)	126.42 (98.66-158.92)	140.73 (99.64-188.66)	114.22 (80.77-158.92)
Niue	15.64 (12.56-18.92)	18.82 (14.58-23.21)	13.05 (9.88-16.73)	12.03 (9.73-14.51)	14.74 (11.55-17.84)	9.99 (7.6-12.71)	281.49 (224.77-345.69)	340.51 (258.76-422.87)	230.95 (175.24-296.73)
North Macedonia	29.07 (23.07-35.39)	35.35 (27.32-44.72)	23.6 (17.7-31.25)	19.26 (15.65-23.16)	23.29 (18.47-28.69)	15.85 (12.13-20.69)	404.98 (321.47-494.2)	490.97 (383.78-615.57)	328.07 (245.78-435.25)
Northern Mariana Islands	22.37 (19.09-25.01)	28.08 (23.19-32.5)	16.75 (13.43-19.87)	15.57 (13.29-17.46)	19.91 (16.51-23.09)	11.53 (9.27-13.82)	358.92 (305.62-398.44)	454.72 (372.54-517.72)	260.75 (210.02-307.78)
Norway	47.11 (42.42-50.84)	52.89 (48.26-57.17)	41.56 (36.14-45.52)	18.12 (15.88-19.54)	20.54 (18.61-22.07)	15.87 (13.49-17.36)	369.37 (337.2-395.53)	425.16 (392.05-457.13)	315.57 (281.19-341.71)
Oman	6.43 (4.96-8.11)	8.07 (6.08-10.47)	5.07 (3.79-6.57)	3.65 (2.85-4.55)	5.08 (3.81-6.48)	2.46 (1.83-3.09)	81.01 (62.04-103.75)	105 (78.32-137.63)	56.84 (42.31-74.59)
Pakistan	6.88 (5.67-8.56)	6.87 (5.19-9.12)	6.88 (5.13-9.18)	6.29 (5.22-7.8)	6.33 (4.81-8.42)	6.24 (4.68-8.22)	154.08 (125.46-192.31)	153.14 (112.34-208.17)	154.98 (113.36-207.23)
Palau	16.94 (13.16-21.11)	4.71 (3.68-5.98)	30.41 (23.24-38.41)	13.55 (10.47-16.95)	3.5 (2.75-4.47)	24.83 (18.83-31.59)	290.2 (223.31-362.2)	92.56 (71.65-117.32)	509.74 (382.76-646.32)
Palestine	23.13 (19.17-27.26)	24.83 (19.69-29.81)	22.37 (18.05-27.22)	15.16 (12.68-17.71)	18.11 (14.55-21.57)	13.26 (10.85-15.89)	342.54 (282.99-404.33)	389.78 (305.94-469.44)	305.93 (247.87-374.52)
Panama	27.81 (21.29-34.22)	39.81 (29.49-49.6)	16.44 (13.13-20.23)	11.12 (8.57-13.52)	12.07 (9.1-14.77)	10.24 (8-12.45)	272.38 (209.78-334.02)	297.95 (222.56-368.73)	248.14 (195.59-301.36)
Papua New Guinea	3.94 (3.08-4.91)	3.34 (2.55-4.27)	4.63 (3.5-5.87)	3.56 (2.8-4.41)	3.13 (2.39-4.03)	4.05 (3.07-5.11)	91.65 (70.55-115.21)	75.79 (57.64-96.64)	109.72 (81.91-140.1)
Paraguay	14.76 (11.38-18.97)	17.03 (12.29-22.53)	12.75 (9.37-16.76)	10.57 (8.18-13.31)	12.01 (8.62-15.82)	9.33 (6.86-12.24)	243.96 (188.24-310.59)	275.16 (198.02-364.44)	215.73 (158.27-283.77)
Peru	14.65 (10.91-19.31)	15.02 (10.64-20.14)	14.28 (10.26-18.92)	9.32 (6.95-12)	9.25 (6.64-12.01)	9.35 (6.82-12.31)	210.6 (157.02-278.12)	214.27 (149.95-289.71)	206.75 (149.08-274.6)
Philippines	16.55 (14.01-19.38)	20.56 (15.97-25.75)	13.1 (10.53-16.13)	13.18 (11.18-15.35)	16.32 (12.75-20.53)	10.57 (8.55-12.92)	342.33 (287.17-402.3)	430.88 (333.74-542.77)	263.51 (208.12-326.8)
Poland	35.32 (31.88-38.43)	49 (43.85-54.21)	25.45 (22.25-28.32)	24.35 (21.86-26.45)	34.23 (30.62-37.75)	17.68 (15.44-19.72)	522.54 (474.13-567.42)	725.15 (647.15-797.43)	368.38 (324.98-412.02)
Portugal	42.44 (36.62-48.89)	57.26 (49.86-65.95)	30.51 (25.56-35.49)	17.32 (14.85-19.68)	23.93 (20.77-27.08)	12.33 (10.28-14.3)	383.54 (331.36-439.81)	523.9 (458.49-597.97)	270.17 (227.8-311.19)
Puerto Rico	46.52 (38.42-56.28)	61.49 (49.71-75.75)	33.96 (28.18-40.87)	13.48 (11.26-16.21)	17.28 (14.2-21.03)	10.34 (8.53-12.29)	337.6 (281.28-406.32)	447.29 (365.84-550.43)	244.02 (202.49-292.7)
Qatar	23.29 (17.72-31.38)	19.75 (14.33-28.94)	30.21 (22.65-40.69)	11.38 (8.7-15.34)	10.49 (7.86-15.45)	12.86 (9.66-17.01)	232.78 (176.63-318.28)	212.47 (155.23-315)	271.11 (201.51-362.8)
Republic of Korea	35.48 (29.08-41.81)	48.75 (38.47-59.21)	24.93 (18.94-31.82)	12.56 (10.32-14.83)	16.96 (13.38-20.56)	9.37 (7.06-12.2)	267.43 (223.39-313.79)	355.58 (285.9-430.32)	195.26 (152.49-248.45)

Republic of Moldova	31.64 (26.71-37.44)	45.83 (38.3-54.32)	21.82 (18.44-25.77)	18.07 (15.29-21.24)	26.94 (22.6-31.63)	12.17 (10.37-14.23)	450.95 (381.09-528.67)	661.47 (554.41-782.32)	301.06 (257.02-351.7)
Romania	38.46 (32.2-45.92)	51.62 (43.61-61.24)	28.4 (23.73-34.21)	21.09 (17.92-24.89)	29.66 (25.33-34.94)	14.83 (12.54-17.69)	504.85 (430.58-593.48)	697.35 (596.63-820.46)	351.78 (297.91-416.94)
Russian Federation	34.18 (31.3-37.02)	40.81 (35.86-44.86)	30.38 (27.14-33.44)	18.99 (17.47-20.56)	24.04 (21.32-26.46)	16.18 (14.4-17.72)	436.3 (400.45-472.48)	545.54 (481.31-602.46)	368.9 (327.36-405.94)
Rwanda	10.01 (7.04-13.31)	12.39 (8.12-17.44)	8.45 (5.08-11.68)	9.49 (6.69-12.68)	11.85 (7.71-16.65)	7.99 (4.73-11.02)	216.77 (151.91-293.35)	265.71 (171.74-377.46)	182.68 (108.8-259.42)
Saint Kitts and Nevis	31.64 (26.54-37.16)	40.71 (33.26-48.64)	23.9 (19.89-28.48)	17.13 (14.45-19.91)	21.54 (18.05-25.17)	13.46 (11.27-15.79)	370.59 (309.14-438.31)	478.43 (393.4-574.94)	274.32 (224.95-328.65)
Saint Lucia	20.58 (16.82-24.97)	23.55 (18.8-29.08)	17.91 (14.62-21.55)	11.03 (9.08-13.3)	12.47 (10.21-15.16)	9.76 (8.05-11.63)	244.76 (199.83-298.55)	280.68 (225.18-346.52)	211.2 (172.87-254.5)
Saint Vincent and the Grenadines	22.65 (19.93-26.01)	24.68 (21.31-28.63)	20.56 (17.89-24)	13.23 (11.65-15.05)	14.32 (12.49-16.49)	12.1 (10.55-13.82)	301.78 (264.09-348.32)	327.2 (285.45-378.36)	275.01 (239.38-319.54)
Samoa	11.59 (9.31-14.21)	9.61 (7.55-12.43)	13.53 (10.37-17.32)	9.35 (7.59-11.42)	7.94 (6.25-10.16)	10.7 (8.24-13.69)	221.44 (176.93-274.29)	183.65 (141.75-240.11)	259.77 (196.57-334.93)
San Marino	30.45 (20.08-43.72)	37.39 (23.96-53.16)	24.07 (15.34-35.63)	11.13 (7.46-15.39)	13.73 (9.19-19.19)	8.76 (5.58-13.06)	239.27 (152.4-336.44)	296.99 (187.97-418.66)	186.19 (118.46-280.21)
Sao Tome and Principe	12.15 (9.98-14.94)	20.14 (15.88-25.66)	5.44 (4.4-6.91)	10.85 (8.98-13.19)	18.07 (14.3-22.91)	4.95 (3.97-6.2)	231.19 (188.42-286.26)	378.76 (296.49-485.23)	102.66 (81.95-131.69)
Saudi Arabia	14.88 (12.12-18.16)	14.61 (11.58-17.92)	15.36 (11.48-19.75)	7.64 (6.29-9.36)	8.9 (7.24-10.89)	5.77 (4.3-7.41)	190.23 (152.15-235.94)	216.91 (170.61-274.35)	149.66 (111.37-193.26)
Senegal	7.26 (5.59-9.41)	8.02 (6.01-10.48)	6.55 (4.8-8.59)	6.9 (5.27-8.92)	7.6 (5.72-9.96)	6.24 (4.61-8.23)	150.77 (114.4-198.68)	166.97 (124.17-224.61)	135.48 (98.65-179.29)
Serbia	35.31 (28.47-43.1)	49.65 (38.58-62.59)	23.59 (17.75-31.03)	21.32 (17.31-25.92)	28.8 (22.79-35.67)	15.59 (11.73-20.24)	484.6 (388.9-590.27)	642.1 (503.69-807.99)	354.22 (263.85-463.37)
Seychelles	27.82 (23.85-31.88)	31.39 (25.82-37.34)	24.25 (20.39-28.43)	19.96 (17.19-22.88)	22.51 (18.57-26.61)	17.44 (14.66-20.32)	469.22 (403.01-539.66)	536.89 (444.04-631.85)	401.67 (339.61-470.78)
Sierra Leone	5.56 (4.16-7.37)	5.65 (4.05-7.81)	5.44 (3.86-7.42)	5.36 (4.03-7.11)	5.43 (3.89-7.48)	5.25 (3.71-7.16)	119.43 (87.67-161.53)	121.84 (84.94-172.66)	116.62 (81.49-160.1)
Singapore	34.92 (30.08-40.08)	40.14 (35.14-45.83)	30.11 (25.45-35.17)	12.43 (10.73-14.16)	14 (12.18-15.86)	11.04 (9.21-12.82)	272.39 (239.77-309.64)	306.3 (270.26-346.68)	240.55 (208.48-277.66)
Slovakia	53.35 (43.82-63.47)	74.42 (58.7-92.42)	38.02 (28.42-49.56)	24.71 (20.25-29.54)	35.76 (28.26-44.07)	17.19 (12.77-22.71)	563 (463.95-671)	795.88 (627.04-983.08)	386.77 (289.41-507.48)
Slovenia	34.16 (27.82-42.22)	48.91 (39.78-59.89)	22.26 (17.62-27.81)	16.18 (13.05-19.72)	23.93 (19.51-28.96)	10.67 (8.37-13.16)	337.4 (273.75-416.54)	485.12 (391.42-596.64)	216.36 (171.39-269.38)
Solomon Islands	10.63 (7.7-14.12)	10.51 (7.57-14.84)	10.74 (7.45-15.44)	9.73 (7.09-12.79)	9.62 (6.96-13.54)	9.82 (6.87-13.91)	243.85 (172.74-326.51)	247.04 (176.77-354)	240.46 (162.09-355.56)
Somalia	9.93 (6.34-14.7)	10.66 (5.51-18.07)	9.43 (5.09-14.7)	9.92 (6.38-14.54)	10.63 (5.66-17.86)	9.43 (5.06-14.61)	243.25 (156.39-363.26)	261.65 (137.54-448.67)	230.03 (122.27-360.59)
South Africa	13.46 (12.07-14.96)	16.64 (14.34-19.3)	11.27 (9.84-12.74)	11.19 (10.02-12.42)	13.88 (11.98-15.96)	9.41 (8.26-10.63)	262.56 (235.23-295.79)	325.18 (278.96-381.38)	216.72 (188.33-248.18)
South Sudan	12.06 (8.35-15.9)	13.71 (8.91-18.67)	10.24 (6.79-14.09)	11.53 (8.11-15.14)	13.15 (8.7-17.87)	9.72 (6.47-13.21)	273.52 (191.51-363.38)	310.54 (203.58-424.79)	232.99 (153.17-325.37)
Spain	46.22 (40.4-51.48)	62.73 (54.81-70.18)	32.74 (27.71-37.29)	16.59 (14.25-18.69)	23.36 (20.36-26.2)	11.46 (9.36-13.21)	361.91 (315.71-403.81)	495.23 (433.98-554.24)	251.55 (213.24-286.01)
Sri Lanka	7.54 (4.99-10.49)	8.58 (5.31-12.03)	6.7 (4.38-9.39)	4.52 (3.03-6.2)	5.23 (3.29-7.3)	3.98 (2.69-5.57)	109.54 (71.59-153.93)	127.32 (78.82-182.1)	94.89 (61.87-134.72)
Sudan	9.04 (6.06-12.8)	8.32 (5.39-12.52)	9.79 (5.99-14.79)	6.95 (4.73-9.8)	6.91 (4.48-10.41)	6.97 (4.27-10.2)	174.14 (115.92-252.47)	167.94 (105.52-259.66)	179.98 (106.93-276.56)
Suriname	22.2 (16.99-28.06)	26.52 (19.25-34.49)	18.48 (13.92-24.09)	13.84 (10.52-17.44)	16.3 (11.66-21.27)	11.73 (8.79-15.27)	333.1 (255.41-418.25)	400.31 (291.57-515.35)	273.35 (208.14-354.81)
Sweden	34.34 (29.04-40.05)	38.23 (31.75-45.6)	30.8 (25.29-36.72)	14.37 (11.88-16.75)	16.03 (13.44-19.07)	12.91 (10.3-15.47)	293.06 (249.23-339.71)	327.93 (277.1-389.72)	260.54 (216.47-307.79)
Switzerland	28.11 (24.03-32.46)	34.07 (29.34-39.4)	23.04 (19.19-26.66)	10.36 (8.65-12)	13.07 (11.1-15.07)	8.18 (6.49-9.54)	219.17 (188.16-250.76)	270.08 (234.33-309.14)	174.86 (146.55-200.48)
Syrian Arab Republic	10.56 (7.75-14.15)	9.95 (6.88-14.08)	11.24 (8.06-14.91)	6.41 (4.73-8.55)	6.56 (4.57-9.29)	6.42 (4.69-8.48)	150.89 (109.66-207.89)	156.93 (108.34-223.51)	145.97 (104.47-195.23)
Taiwan (Province of China)	51.62 (45.24-57.72)	67.48 (58.89-76.07)	37.44 (32.69-41.93)	21.86 (18.95-24.57)	28.71 (25.01-32.39)	16 (13.57-18.28)	503.5 (445.35-558.95)	670.23 (591.78-753.07)	354.19 (312.45-392.96)

Tajikistan	5.56 (4.29-6.93)	6.42 (4.57-8.84)	4.76 (3.17-6.74)	4.69 (3.63-5.88)	5.46 (3.84-7.5)	3.98 (2.64-5.73)	122.52 (92.92-159.66)	141.7 (99.71-199.2)	104.12 (66.48-147.86)
Thailand	26.25 (19.95-33.54)	34.52 (23.56-45.54)	19.01 (14.48-24.45)	14.89 (11.43-18.95)	19.46 (13.18-25.65)	10.99 (8.39-13.93)	380.26 (287.87-486.34)	512.89 (352.74-681.45)	263.01 (200.62-337.38)
Timor-Leste	10.66 (8.11-13.78)	10.37 (7.35-13.44)	10.96 (8.01-15.16)	9.4 (7.23-12.26)	9.25 (6.6-11.99)	9.58 (7.13-13.2)	227.22 (171.7-296.93)	222.92 (154.59-291.75)	232.12 (169.4-327.71)
Togo	6.61 (4.53-9.15)	7.61 (5.2-10.48)	5.88 (4.03-8.27)	6.26 (4.33-8.62)	7.15 (4.91-9.74)	5.61 (3.87-7.82)	139.97 (92.93-195.14)	162.42 (109.81-225.81)	123.39 (80.55-179.79)
Tokelau	12.79 (9.97-16.58)	12.74 (9.23-17.45)	12.85 (9.47-16.72)	10.03 (7.75-12.86)	10.23 (7.42-13.77)	9.87 (7.3-12.83)	240.72 (187.63-314.76)	240.5 (174.49-333.49)	241.67 (178.7-314.67)
Tonga	8.93 (6.93-11.36)	7.9 (6.19-10.17)	9.72 (7.32-12.99)	7.22 (5.65-9.12)	6.34 (5.02-8.05)	7.86 (5.98-10.51)	163.21 (125.84-209.31)	149.34 (116.83-190.58)	174 (130.25-231.91)
Trinidad and Tobago	30.36 (23.02-38.79)	34.77 (25.92-44.62)	26.39 (19.93-33.72)	14.56 (11.18-18.53)	16.93 (12.83-21.58)	12.44 (9.58-15.71)	355.66 (268.26-457.15)	415.94 (312.57-536.74)	298.96 (229.85-383.83)
Trkiye	13.13 (9.49-17.88)	14.52 (10.18-20.36)	11.94 (8.22-16.47)	7.52 (5.47-10)	9.17 (6.55-12.47)	6.09 (4.24-8.4)	173.62 (124.24-238.55)	207.09 (145.29-290.51)	142.91 (98.05-199.42)
Tunisia	6.79 (5.11-8.87)	8.11 (6.07-10.6)	5.81 (4.31-7.67)	5.18 (3.97-6.74)	6.31 (4.85-8.16)	4.38 (3.32-5.8)	140.91 (106.84-184.79)	166.95 (127.1-217.44)	120.27 (90.03-160.05)
Turkmenistan	12.64 (10.21-15.89)	12.92 (9.38-16.92)	12.35 (9.1-16.65)	10.88 (8.76-13.79)	11.22 (8.2-14.54)	10.57 (7.77-14.3)	263.31 (209.89-336.45)	273.66 (197.08-362.27)	253 (184.11-346.75)
Tuvalu	22.86 (18.36-28.06)	27.14 (21.01-33.96)	19.3 (14.84-23.95)	12.93 (10.42-15.75)	16.51 (12.9-20.57)	9.91 (7.58-12.37)	299.82 (240.5-368.43)	383.85 (298.08-483.03)	225.16 (174.05-277.2)
Uganda	11.44 (8.87-14.84)	13.88 (10.59-18.43)	9.63 (7.06-12.89)	10.64 (8.29-13.83)	12.93 (9.94-17.1)	8.99 (6.66-11.99)	252.54 (192.21-337.66)	307.69 (227.54-416.27)	209.53 (150.21-288.61)
Ukraine	25.12 (18.71-32.57)	32.09 (21.34-45.99)	20.96 (14.29-30.33)	15.67 (11.64-20.17)	21.37 (14.25-29.79)	12.38 (8.42-17.48)	397.57 (288.28-518.22)	523.84 (346.46-739.08)	315.95 (207.65-457.6)
United Arab Emirates	18.79 (14.27-26.88)	10.74 (7.5-17.03)	49.32 (37.37-69.01)	13.05 (9.99-18.53)	7.89 (5.53-12.59)	34.12 (25.92-47.33)	264.33 (200.81-376.61)	167.08 (117.36-265.29)	631.72 (480.18-880.03)
United Kingdom	38.75 (35.98-40.37)	45.46 (42.91-47.34)	32.83 (29.76-34.56)	15.72 (14.3-16.51)	18.66 (17.38-19.44)	13.24 (11.68-14.08)	335.26 (314.03-348.83)	397.57 (377.23-413.73)	279.34 (258.99-293)
United Republic of Tanzania	10.15 (7.58-12.91)	11.4 (7.76-15.02)	9.08 (6.65-12.3)	9.5 (7.18-12.14)	10.73 (7.33-14)	8.47 (6.23-11.57)	220.3 (162.36-289.53)	247.92 (165.95-334.5)	195.53 (140.8-266.84)
United States of America	38.17 (35.56-39.98)	44.91 (42.32-46.88)	32.16 (29.28-34)	12.79 (11.72-13.51)	15.16 (14.18-15.88)	10.74 (9.54-11.49)	315.59 (298.11-330.02)	378.66 (360.03-394.79)	258.62 (240.28-272.64)
United States Virgin Islands	30.36 (23.4-38.83)	40.95 (30.15-55.18)	21.73 (15.41-31.48)	13.95 (10.72-17.67)	19 (13.84-25.49)	10.24 (7.39-14.82)	330.77 (254.83-422.05)	450.27 (338.34-600.3)	229.08 (164.75-340.05)
Uruguay	43.22 (38.45-48.54)	53.73 (47.01-60.59)	35.63 (31.22-40.67)	27.46 (24.25-30.91)	34.6 (30.42-39.01)	22.52 (19.36-25.62)	598.78 (533.29-672.27)	742.58 (656.08-833.98)	490.42 (431.83-554.52)
Uzbekistan	6.34 (5.11-7.83)	7.4 (5.94-9.12)	5.52 (4.38-6.79)	4.82 (3.86-5.91)	5.75 (4.59-6.99)	4.14 (3.31-5.05)	131.07 (105.91-160.54)	151.76 (122.51-184.27)	114.18 (91.16-138.99)
Vanuatu	11.18 (8.62-14.41)	11.8 (8.76-15.77)	10.59 (7.16-15.36)	10.23 (7.9-13.11)	10.83 (7.97-14.53)	9.68 (6.58-14.02)	252.45 (192.41-326.02)	271.42 (200.27-366.14)	234.03 (155.33-339.83)
Venezuela (Bolivarian Republic of Venezuela)	18.16 (13.38-23.62)	23.29 (16.9-31.22)	13.86 (9.88-18.76)	10.25 (7.64-13.2)	10.88 (7.85-14.37)	9.71 (7.02-13.15)	254.94 (187.83-332.69)	273.78 (195.91-365.03)	238.76 (170.73-326.3)
Viet Nam	19.08 (14.66-23)	27.52 (21.18-32.99)	12.97 (9.33-17.13)	12.12 (9.45-14.63)	18.22 (14.02-21.89)	8.08 (5.92-10.54)	299.88 (228.83-365.46)	431.15 (325.6-524.64)	200.21 (144.28-264.96)
Yemen	8.33 (5.43-11.95)	8.04 (5.28-12.1)	8.63 (5.22-12.79)	6.91 (4.55-9.83)	7.08 (4.72-10.77)	6.76 (4.12-10.04)	169.25 (111.11-246.93)	169.47 (111.01-257.02)	169.29 (100.05-258.01)
Zambia	14.58 (9.44-26.19)	18.33 (11.7-36.98)	11.32 (7.18-19.82)	13.54 (8.97-23.41)	17.1 (11-33.12)	10.51 (6.84-17.98)	329.22 (207.45-621.59)	412.81 (253.92-878.71)	253.4 (155.78-465)
Zimbabwe	14.51 (11.28-18.18)	14.15 (11.14-17.91)	14.73 (10.85-19.16)	13.76 (10.88-17.01)	13.32 (10.54-16.73)	14.03 (10.47-17.98)	333.74 (255-427.34)	331.15 (257.37-425.39)	335.27 (241.38-446.44)

ASIR age-standardized incidence rate, ASMR age-standardized mortality rate, ASDR age-standardized disability-adjusted life-year rate, UI uncertainty interval

Table.S5 EAPC of ASIR, ASMR, and ASDR for colorectal cancer across 204 countries in 2021

Location	EAPC of ASIR (95%CI)			EAPC of ASMR (95%CI)			EAPC of ASDR (95%CI)		
	Both	Male	Female	Both	Male	Female	Both	Male	Female
Afghanistan	0.57 (0.46 to 0.67)	0.20 (0.09 to 0.31)	0.56 (0.45 to 0.67)	0.26 (0.19 to 0.33)	0.02 (-0.07 to 0.11)	0.21 (0.14 to 0.28)	0.16 (0.09 to 0.22)	-0.10 (-0.18 to -0.02)	0.14 (0.07 to 0.21)
Albania	1.31 (1.11 to 1.51)	0.97 (0.80 to 1.15)	1.46 (1.23 to 1.69)	0.25 (0.03 to 0.48)	-0.21 (-0.41 to -0.01)	0.46 (0.22 to 0.71)	0.11 (-0.10 to 0.32)	-0.11 (-0.30 to 0.09)	0.19 (-0.04 to 0.42)
Algeria	0.83 (0.67 to 0.99)	0.59 (0.47 to 0.71)	1.14 (0.91 to 1.36)	0.03 (-0.15 to 0.21)	-0.14 (-0.26 to -0.01)	0.31 (0.03 to 0.59)	-0.25 (-0.36 to -0.13)	-0.30 (-0.39 to -0.22)	-0.17 (-0.33 to 0.00)
American Samoa	0.45 (0.35 to 0.55)	0.20 (0.10 to 0.31)	0.75 (0.64 to 0.87)	0.22 (0.10 to 0.34)	-0.01 (-0.14 to 0.11)	0.48 (0.35 to 0.62)	0.22 (0.10 to 0.34)	-0.03 (-0.16 to 0.09)	0.55 (0.42 to 0.69)
Andorra	-0.07 (-0.34 to 0.20)	-0.02 (-0.34 to 0.31)	-0.01 (-0.26 to 0.24)	-1.11 (-1.37 to -0.85)	-1.03 (-1.36 to -0.69)	-1.02 (-1.18 to -0.86)	-1.16 (-1.40 to -0.92)	-1.09 (-1.40 to -0.79)	-1.09 (-1.25 to -0.93)
Angola	0.48 (0.34 to 0.62)	0.73 (0.54 to 0.93)	0.33 (0.25 to 0.42)	0.27 (0.17 to 0.38)	0.54 (0.37 to 0.70)	0.13 (0.07 to 0.20)	0.17 (0.05 to 0.28)	0.41 (0.23 to 0.59)	0.02 (-0.05 to 0.09)
Antigua and Barbuda	1.50 (1.32 to 1.68)	1.05 (0.75 to 1.35)	1.91 (1.69 to 2.12)	0.74 (0.55 to 0.93)	0.38 (0.05 to 0.71)	1.07 (0.85 to 1.29)	0.61 (0.45 to 0.77)	0.16 (-0.11 to 0.42)	1.00 (0.80 to 1.20)
Argentina	0.61 (0.37 to 0.85)	0.70 (0.42 to 0.98)	0.50 (0.30 to 0.71)	-0.06 (-0.28 to 0.16)	0.07 (-0.18 to 0.33)	-0.19 (-0.39 to 0.00)	0.00 (-0.20 to 0.21)	0.07 (-0.18 to 0.31)	-0.07 (-0.24 to 0.09)
Armenia	0.71 (0.47 to 0.94)	0.91 (0.56 to 1.27)	0.49 (0.29 to 0.70)	0.26 (0.05 to 0.48)	0.40 (0.07 to 0.72)	0.11 (-0.09 to 0.31)	-0.32 (-0.52 to -0.11)	-0.08 (-0.41 to 0.25)	-0.55 (-0.73 to -0.38)
Australia	-0.55 (-0.71 to -0.40)	-0.59 (-0.76 to -0.42)	-0.57 (-0.71 to -0.43)	-1.95 (-2.05 to -1.85)	-1.99 (-2.10 to -1.87)	-1.99 (-2.09 to -1.90)	-2.06 (-2.17 to -1.95)	-2.06 (-2.18 to -1.94)	-2.11 (-2.21 to -2.00)
Austria	-1.25 (-1.40 to -1.10)	-1.28 (-1.46 to -1.11)	-1.47 (-1.60 to -1.33)	-2.42 (-2.48 to -2.37)	-2.43 (-2.51 to -2.35)	-2.69 (-2.76 to -2.62)	-2.46 (-2.52 to -2.40)	-2.48 (-2.57 to -2.39)	-2.68 (-2.75 to -2.60)
Azerbaijan	0.13 (-0.21 to 0.47)	0.14 (-0.29 to 0.58)	-0.03 (-0.28 to 0.22)	-0.26 (-0.55 to 0.03)	-0.28 (-0.67 to 0.11)	-0.42 (-0.64 to -0.21)	-0.73 (-1.00 to -0.45)	-0.68 (-1.05 to -0.31)	-0.87 (-1.06 to -0.69)
Bahamas	1.49 (1.28 to 1.70)	1.60 (1.40 to 1.80)	1.36 (1.10 to 1.61)	0.80 (0.66 to 0.94)	0.90 (0.76 to 1.05)	0.68 (0.51 to 0.85)	0.65 (0.54 to 0.76)	0.75 (0.63 to 0.87)	0.52 (0.38 to 0.66)
Bahrain	0.31 (0.04 to 0.58)	0.11 (-0.20 to 0.42)	0.67 (0.45 to 0.89)	-0.90 (-1.22 to -0.58)	-0.98 (-1.33 to -0.62)	-0.80 (-1.08 to -0.51)	-1.16 (-1.43 to -0.90)	-1.20 (-1.51 to -0.88)	-1.10 (-1.29 to -0.90)
Bangladesh	-0.28 (-0.40 to -0.16)	-0.34 (-0.51 to -0.16)	-0.17 (-0.27 to -0.06)	-0.85 (-1.00 to -0.70)	-0.87 (-1.08 to -0.66)	-0.77 (-0.86 to -0.68)	-0.99 (-1.09 to -0.89)	-1.04 (-1.18 to -0.89)	-0.91 (-1.01 to -0.81)
Barbados	1.74 (1.45 to 2.04)	1.30 (1.03 to 1.57)	2.13 (1.73 to 2.53)	0.95 (0.69 to 1.21)	0.50 (0.25 to 0.75)	1.33 (0.98 to 1.69)	0.86 (0.62 to 1.09)	0.46 (0.26 to 0.67)	1.21 (0.87 to 1.55)
Belarus	0.68 (0.32 to 1.04)	1.09 (0.84 to 1.34)	0.31 (-0.23 to 0.85)	-0.47 (-0.78 to -0.15)	0.03 (-0.21 to 0.26)	-0.88 (-1.36 to -0.40)	-0.69 (-1.00 to -0.37)	-0.28 (-0.52 to -0.03)	-1.07 (-1.54 to -0.60)
Belgium	-0.65 (-0.79 to -0.50)	-0.57 (-0.76 to -0.39)	-0.87 (-0.98 to -0.76)	-1.80 (-1.90 to -1.71)	-1.75 (-1.88 to -1.62)	-2.03 (-2.12 to -1.94)	-1.76 (-1.88 to -1.65)	-1.69 (-1.84 to -1.54)	-1.98 (-2.08 to -1.89)
Belize	1.94 (1.52 to 2.36)	2.06 (1.56 to 2.57)	1.81 (1.45 to 2.16)	1.23 (0.73 to 1.72)	1.32 (0.75 to 1.90)	1.13 (0.70 to 1.56)	1.30 (0.83 to 1.78)	1.46 (0.91 to 2.01)	1.14 (0.74 to 1.53)
Benin	0.71 (0.61 to 0.81)	0.65 (0.54 to 0.76)	0.82 (0.72 to 0.92)	0.61 (0.51 to 0.70)	0.53 (0.42 to 0.63)	0.74 (0.64 to 0.85)	0.41 (0.31 to 0.51)	0.38 (0.26 to 0.49)	0.47 (0.38 to 0.57)
Bermuda	0.33 (0.18 to 0.47)	0.92 (0.62 to 1.23)	-0.38 (-0.61 to -0.15)	-1.25 (-1.43 to -1.07)	-0.69 (-1.00 to -0.39)	-1.82 (-2.07 to -1.56)	-1.26 (-1.44 to -1.08)	-0.74 (-1.04 to -0.44)	-1.87 (-2.13 to -1.62)
Bhutan	0.36 (0.22 to 0.50)	0.66 (0.49 to 0.84)	0.01 (-0.11 to 0.14)	-0.08 (-0.19 to 0.03)	0.22 (0.08 to 0.36)	-0.45 (-0.55 to -0.35)	-0.40 (-0.51 to -0.29)	-0.05 (-0.19 to 0.08)	-0.77 (-0.87 to -0.66)
Bolivia (Plurinational State of Bolivia)	0.62 (0.59 to 0.65)	0.87 (0.81 to 0.93)	0.47 (0.43 to 0.50)	0.19 (0.16 to 0.22)	0.41 (0.35 to 0.47)	0.06 (0.02 to 0.10)	-0.04 (-0.08 to 0.00)	0.18 (0.11 to 0.26)	-0.18 (-0.22 to -0.14)
Bosnia and Herzegovina	2.37 (2.06 to 2.68)	3.03 (2.63 to 3.43)	1.54 (1.32 to 1.76)	1.39 (1.19 to 1.60)	2.04 (1.74 to 2.35)	0.60 (0.48 to 0.73)	1.17 (0.94 to 1.40)	1.79 (1.48 to 2.10)	0.37 (0.21 to 0.52)
Botswana	0.34 (0.14 to 0.53)	0.21 (-0.18 to 0.60)	0.66 (0.16 to 1.17)	0.14 (-0.07 to 0.35)	0.05 (-0.37 to 0.48)	0.42 (-0.03 to 0.88)	-0.03 (-0.27 to 0.20)	-0.23 (-0.69 to 0.24)	0.39 (-0.13 to 0.91)
Brazil	1.40 (1.28 to 1.52)	1.79 (1.67 to 1.91)	1.05 (0.92 to 1.17)	0.63 (0.54 to 0.72)	1.02 (0.93 to 1.12)	0.30 (0.19 to 0.40)	0.75 (0.65 to 0.85)	1.07 (0.97 to 1.17)	0.46 (0.35 to 0.57)
Brunei Darussalam	-0.01 (-0.26 to 0.24)	0.25 (-0.08 to 0.59)	-0.13 (-0.32 to 0.06)	-0.50 (-0.81 to -0.19)	-0.16 (-0.58 to 0.27)	-0.64 (-0.88 to -0.41)	-0.72 (-1.03 to -0.41)	-0.52 (-0.89 to -0.15)	-0.83 (-1.09 to -0.56)
Bulgaria	1.88 (1.72 to 2.05)	2.39 (2.22 to 2.55)	1.34 (1.16 to 1.52)	1.05 (0.85 to 1.25)	1.59 (1.40 to 1.79)	0.51 (0.29 to 0.73)	0.85 (0.68 to 1.02)	1.35 (1.18 to 1.52)	0.28 (0.10 to 0.46)
Burkina Faso	0.88 (0.79 to 0.96)	1.01 (0.88 to 1.15)	0.75 (0.68 to 0.81)	0.81 (0.73 to 0.89)	0.93 (0.79 to 1.07)	0.69 (0.63 to 0.75)	0.66 (0.57 to 0.76)	0.81 (0.66 to 0.96)	0.52 (0.46 to 0.59)
Burundi	-0.68 (-0.84 to -0.53)	-0.48 (-0.67 to -0.30)	-0.98 (-1.13 to -0.83)	-0.71 (-0.86 to -0.57)	-0.49 (-0.66 to -0.33)	-1.02 (-1.16 to -0.87)	-1.01 (-1.18 to -0.84)	-0.82 (-1.02 to -0.63)	-1.30 (-1.47 to -1.13)
Cabo Verde	2.93 (2.54 to 3.32)	3.27 (2.74 to 3.81)	2.56 (2.20 to 2.92)	2.49 (2.06 to 2.92)	2.73 (2.15 to 3.32)	2.21 (1.82 to 2.60)	2.14 (1.73 to 2.55)	2.54 (2.02 to 3.06)	1.69 (1.30 to 2.08)
Cambodia	0.83 (0.73 to 0.92)	1.36 (1.25 to 1.48)	0.37 (0.28 to 0.47)	0.37 (0.30 to 0.45)	0.91 (0.81 to 1.01)	-0.08 (-0.16 to 0.00)	0.15 (0.09 to 0.22)	0.72 (0.65 to 0.79)	-0.36 (-0.44 to -0.28)
Cameroon	0.72 (0.64 to 0.79)	0.74 (0.66 to 0.82)	0.68 (0.59 to 0.77)	0.58 (0.50 to 0.67)	0.58 (0.50 to 0.66)	0.58 (0.49 to 0.67)	0.48 (0.39 to 0.56)	0.54 (0.44 to 0.63)	0.39 (0.30 to 0.48)
Canada	-0.05 (-0.26 to 0.17)	0.03 (-0.19 to 0.26)	-0.23 (-0.43 to -0.03)	-1.04 (-1.15 to -0.93)	-1.02 (-1.14 to -0.89)	-1.16 (-1.26 to -1.07)	-1.09 (-1.18 to -1.00)	-1.01 (-1.11 to -0.90)	-1.27 (-1.35 to -1.19)
Central African Republic	-0.11 (-0.17 to -0.05)	-0.01 (-0.06 to 0.05)	-0.16 (-0.25 to -0.07)	-0.13 (-0.20 to -0.07)	-0.02 (-0.08 to 0.03)	-0.18 (-0.27 to -0.09)	-0.17 (-0.24 to -0.10)	-0.09 (-0.15 to -0.04)	-0.22 (-0.31 to -0.12)
Chad	1.55 (1.44 to 1.66)	1.44 (1.34 to 1.54)	1.62 (1.49 to 1.75)	1.50 (1.37 to 1.62)	1.36 (1.25 to 1.48)	1.58 (1.44 to 1.72)	1.41 (1.29 to 1.54)	1.33 (1.21 to 1.45)	1.45 (1.31 to 1.59)
Chile	1.87 (1.75 to 2.00)	2.19 (2.02 to 2.35)	1.57 (1.44 to 1.69)	0.47 (0.35 to 0.59)	0.81 (0.64 to 0.97)	0.14 (0.02 to 0.26)	0.53 (0.40 to 0.67)	0.82 (0.64 to 1.00)	0.24 (0.13 to 0.36)

China	1.75 (1.64 to 1.87)	2.32 (2.19 to 2.45)	0.86 (0.74 to 0.98)	-0.49 (-0.55 to -0.43)	0.10 (0.04 to 0.16)	-1.38 (-1.49 to -1.26)	-0.62 (-0.71 to -0.54)	0.02 (-0.05 to 0.09)	-1.63 (-1.77 to -1.50)
Colombia	1.71 (1.57 to 1.84)	2.41 (2.26 to 2.57)	0.96 (0.81 to 1.10)	0.19 (0.04 to 0.35)	0.67 (0.48 to 0.86)	-0.20 (-0.35 to -0.05)	0.38 (0.22 to 0.54)	0.80 (0.63 to 0.98)	0.02 (-0.15 to 0.20)
Comoros	0.34 (0.22 to 0.45)	0.78 (0.60 to 0.95)	-0.06 (-0.14 to 0.02)	0.24 (0.13 to 0.34)	0.69 (0.53 to 0.84)	-0.17 (-0.25 to -0.10)	-0.06 (-0.20 to 0.09)	0.38 (0.19 to 0.58)	-0.45 (-0.58 to -0.33)
Congo	-0.06 (-0.19 to 0.08)	-0.29 (-0.51 to -0.08)	0.14 (0.04 to 0.25)	-0.29 (-0.41 to -0.18)	-0.49 (-0.67 to -0.31)	-0.14 (-0.24 to -0.04)	-0.42 (-0.55 to -0.29)	-0.72 (-0.93 to -0.51)	-0.15 (-0.28 to -0.02)
Cook Islands	-0.17 (-0.31 to -0.03)	0.08 (-0.04 to 0.20)	-0.42 (-0.64 to -0.20)	-1.19 (-1.35 to -1.03)	-0.98 (-1.11 to -0.85)	-1.40 (-1.64 to -1.15)	-1.17 (-1.34 to -0.99)	-0.86 (-0.97 to -0.75)	-1.47 (-1.74 to -1.20)
Costa Rica	3.05 (2.82 to 3.28)	4.06 (3.75 to 4.38)	1.71 (1.56 to 1.87)	1.80 (1.60 to 2.00)	2.87 (2.56 to 3.18)	0.88 (0.72 to 1.03)	1.99 (1.76 to 2.22)	3.08 (2.75 to 3.41)	0.99 (0.83 to 1.16)
Croatia	1.26 (1.07 to 1.46)	1.29 (1.08 to 1.51)	0.97 (0.78 to 1.16)	0.15 (-0.03 to 0.33)	0.19 (-0.02 to 0.40)	-0.21 (-0.38 to -0.03)	0.07 (-0.13 to 0.28)	0.16 (-0.07 to 0.40)	-0.30 (-0.49 to -0.11)
Cuba	1.50 (1.41 to 1.59)	1.61 (1.49 to 1.74)	1.39 (1.31 to 1.48)	0.45 (0.34 to 0.56)	0.53 (0.37 to 0.69)	0.36 (0.26 to 0.45)	0.41 (0.28 to 0.54)	0.52 (0.36 to 0.68)	0.30 (0.17 to 0.44)
Cyprus	1.10 (0.78 to 1.42)	1.35 (0.98 to 1.72)	0.75 (0.44 to 1.06)	-1.12 (-1.28 to -0.96)	-0.87 (-1.13 to -0.61)	-1.38 (-1.55 to -1.21)	-1.00 (-1.13 to -0.87)	-0.70 (-0.89 to -0.52)	-1.42 (-1.54 to -1.29)
Czechia	-0.88 (-1.17 to -0.58)	-0.82 (-1.14 to -0.50)	-1.15 (-1.43 to -0.87)	-2.05 (-2.25 to -1.85)	-1.97 (-2.19 to -1.74)	-2.33 (-2.52 to -2.15)	-2.15 (-2.33 to -1.96)	-2.11 (-2.32 to -1.89)	-2.38 (-2.54 to -2.23)
Côte d'Ivoire	0.12 (0.04 to 0.19)	-0.27 (-0.42 to -0.12)	0.57 (0.46 to 0.69)	-0.08 (-0.18 to 0.02)	-0.45 (-0.62 to -0.29)	0.35 (0.23 to 0.47)	-0.13 (-0.24 to -0.03)	-0.53 (-0.72 to -0.35)	0.36 (0.24 to 0.49)
Democratic People's Republic of Korea	0.58 (0.47 to 0.68)	0.62 (0.50 to 0.73)	0.40 (0.29 to 0.51)	-0.16 (-0.26 to -0.06)	-0.10 (-0.21 to 0.00)	-0.33 (-0.44 to -0.22)	-0.10 (-0.18 to -0.02)	-0.04 (-0.12 to 0.04)	-0.31 (-0.40 to -0.23)
Democratic Republic of the Congo	0.20 (-0.02 to 0.43)	0.20 (-0.08 to 0.47)	0.28 (0.11 to 0.44)	0.06 (-0.13 to 0.25)	0.07 (-0.17 to 0.32)	0.14 (0.00 to 0.27)	0.04 (-0.15 to 0.24)	0.02 (-0.22 to 0.27)	0.09 (-0.05 to 0.23)
Denmark	0.38 (0.07 to 0.69)	0.46 (0.12 to 0.80)	0.20 (-0.09 to 0.50)	-1.07 (-1.36 to -0.78)	-1.01 (-1.32 to -0.69)	-1.22 (-1.50 to -0.94)	-1.36 (-1.63 to -1.08)	-1.25 (-1.54 to -0.96)	-1.55 (-1.82 to -1.29)
Djibouti	1.09 (1.03 to 1.15)	1.36 (1.28 to 1.44)	0.63 (0.58 to 0.67)	0.96 (0.89 to 1.04)	1.25 (1.15 to 1.34)	0.51 (0.44 to 0.58)	0.77 (0.67 to 0.86)	1.04 (0.93 to 1.15)	0.29 (0.20 to 0.37)
Dominica	0.97 (0.86 to 1.08)	0.84 (0.72 to 0.96)	0.91 (0.78 to 1.05)	0.61 (0.54 to 0.68)	0.47 (0.40 to 0.54)	0.57 (0.48 to 0.66)	0.67 (0.60 to 0.74)	0.55 (0.48 to 0.61)	0.55 (0.47 to 0.63)
Dominican Republic	1.79 (1.56 to 2.01)	2.40 (2.14 to 2.66)	1.16 (0.92 to 1.39)	1.02 (0.80 to 1.23)	1.64 (1.43 to 1.85)	0.40 (0.12 to 0.67)	1.15 (1.00 to 1.30)	1.74 (1.58 to 1.90)	0.52 (0.35 to 0.70)
Ecuador	1.98 (1.66 to 2.30)	2.48 (2.09 to 2.86)	1.57 (1.28 to 1.86)	1.23 (0.90 to 1.56)	1.77 (1.38 to 2.15)	0.82 (0.50 to 1.14)	1.26 (0.94 to 1.59)	1.66 (1.28 to 2.05)	0.92 (0.61 to 1.23)
Egypt	2.82 (2.56 to 3.08)	2.34 (2.15 to 2.53)	3.49 (3.13 to 3.84)	2.15 (1.84 to 2.46)	1.79 (1.56 to 2.03)	2.83 (2.43 to 3.24)	1.80 (1.55 to 2.05)	1.44 (1.25 to 1.62)	2.34 (2.01 to 2.68)
El Salvador	2.79 (2.50 to 3.09)	3.39 (3.02 to 3.76)	2.23 (2.00 to 2.46)	1.52 (1.35 to 1.70)	1.78 (1.54 to 2.02)	1.32 (1.16 to 1.48)	1.56 (1.39 to 1.74)	1.98 (1.74 to 2.22)	1.22 (1.07 to 1.37)
Equatorial Guinea	1.47 (1.29 to 1.64)	1.72 (1.47 to 1.97)	1.19 (1.05 to 1.32)	0.88 (0.74 to 1.03)	1.17 (0.96 to 1.39)	0.56 (0.46 to 0.67)	0.60 (0.44 to 0.77)	0.83 (0.57 to 1.09)	0.34 (0.23 to 0.46)
Eritrea	0.62 (0.54 to 0.69)	0.80 (0.64 to 0.95)	0.53 (0.47 to 0.58)	0.58 (0.49 to 0.67)	0.78 (0.63 to 0.94)	0.48 (0.42 to 0.54)	0.31 (0.23 to 0.39)	0.45 (0.29 to 0.62)	0.23 (0.17 to 0.29)
Estonia	0.98 (0.80 to 1.16)	1.39 (1.19 to 1.59)	0.55 (0.36 to 0.74)	-0.41 (-0.57 to -0.25)	0.07 (-0.12 to 0.26)	-0.87 (-1.04 to -0.70)	-0.86 (-1.04 to -0.68)	-0.44 (-0.64 to -0.24)	-1.31 (-1.50 to -1.11)
Eswatini	1.41 (0.92 to 1.89)	1.84 (1.38 to 2.31)	1.05 (0.55 to 1.54)	1.26 (0.75 to 1.76)	1.68 (1.20 to 2.17)	0.93 (0.42 to 1.44)	1.41 (0.83 to 1.99)	1.86 (1.29 to 2.43)	0.98 (0.40 to 1.57)
Ethiopia	-1.15 (-1.37 to -0.93)	-0.99 (-1.27 to -0.72)	-1.43 (-1.65 to -1.20)	-1.32 (-1.51 to -1.12)	-1.17 (-1.42 to -0.93)	-1.62 (-1.83 to -1.41)	-1.74 (-1.95 to -1.53)	-1.56 (-1.81 to -1.30)	-2.03 (-2.25 to -1.80)
Fiji	0.43 (0.22 to 0.63)	0.55 (0.38 to 0.71)	0.37 (0.14 to 0.60)	0.28 (0.09 to 0.47)	0.42 (0.27 to 0.57)	0.24 (0.01 to 0.46)	0.18 (-0.05 to 0.40)	0.30 (0.11 to 0.49)	0.09 (-0.15 to 0.33)
Finland	0.54 (0.47 to 0.61)	0.60 (0.52 to 0.68)	0.30 (0.23 to 0.38)	-0.90 (-1.03 to -0.78)	-0.86 (-0.97 to -0.75)	-1.14 (-1.28 to -0.99)	-0.98 (-1.07 to -0.89)	-0.92 (-1.01 to -0.84)	-1.20 (-1.29 to -1.10)
France	0.29 (0.16 to 0.41)	0.07 (-0.07 to 0.22)	0.40 (0.30 to 0.50)	-1.46 (-1.52 to -1.41)	-1.62 (-1.67 to -1.57)	-1.45 (-1.51 to -1.39)	-1.37 (-1.43 to -1.31)	-1.51 (-1.58 to -1.45)	-1.30 (-1.35 to -1.24)
Gabon	0.28 (0.22 to 0.34)	0.38 (0.27 to 0.48)	0.08 (-0.08 to 0.24)	-0.03 (-0.08 to 0.03)	0.09 (0.02 to 0.16)	-0.22 (-0.40 to -0.04)	-0.14 (-0.20 to -0.08)	-0.07 (-0.15 to 0.01)	-0.35 (-0.54 to -0.16)
Gambia	0.51 (0.37 to 0.64)	0.54 (0.42 to 0.66)	0.45 (0.30 to 0.61)	0.35 (0.23 to 0.47)	0.39 (0.28 to 0.49)	0.30 (0.16 to 0.43)	0.28 (0.12 to 0.44)	0.31 (0.17 to 0.44)	0.22 (0.04 to 0.40)
Georgia	1.97 (1.52 to 2.43)	2.43 (1.94 to 2.93)	1.34 (0.89 to 1.80)	2.09 (1.59 to 2.60)	2.49 (1.95 to 3.04)	1.52 (1.02 to 2.03)	1.57 (1.15 to 1.99)	2.03 (1.56 to 2.51)	0.92 (0.52 to 1.32)
Germany	-0.91 (-1.08 to -0.75)	-0.75 (-0.93 to -0.57)	-1.30 (-1.44 to -1.15)	-2.18 (-2.30 to -2.06)	-2.02 (-2.13 to -1.90)	-2.57 (-2.71 to -2.42)	-2.07 (-2.19 to -1.96)	-1.93 (-2.04 to -1.82)	-2.44 (-2.59 to -2.29)
Ghana	1.27 (1.21 to 1.33)	1.82 (1.68 to 1.96)	0.78 (0.71 to 0.85)	1.11 (1.04 to 1.18)	1.62 (1.47 to 1.77)	0.66 (0.61 to 0.72)	0.96 (0.89 to 1.04)	1.61 (1.44 to 1.77)	0.41 (0.35 to 0.46)
Greece	-0.04 (-0.25 to 0.17)	0.43 (0.23 to 0.63)	-0.57 (-0.80 to -0.34)	-0.75 (-0.96 to -0.53)	-0.26 (-0.46 to -0.06)	-1.24 (-1.48 to -0.99)	-0.55 (-0.71 to -0.39)	-0.13 (-0.30 to 0.03)	-1.04 (-1.21 to -0.88)
Greenland	-0.95 (-1.04 to -0.87)	-0.50 (-0.58 to -0.41)	-1.49 (-1.60 to -1.38)	-1.79 (-1.88 to -1.70)	-1.33 (-1.41 to -1.25)	-2.30 (-2.42 to -2.17)	-1.83 (-1.90 to -1.75)	-1.42 (-1.49 to -1.35)	-2.34 (-2.45 to -2.22)
Grenada	1.63 (1.37 to 1.89)	1.87 (0.96 to 2.77)	1.74 (1.52 to 1.96)	1.07 (0.83 to 1.31)	1.49 (0.53 to 2.46)	1.16 (0.85 to 1.47)	0.83 (0.68 to 0.98)	1.00 (0.33 to 1.68)	0.89 (0.59 to 1.19)
Guam	0.01 (-0.27 to 0.29)	0.12 (-0.13 to 0.37)	-0.02 (-0.39 to 0.35)	-0.77 (-1.15 to -0.38)	-0.54 (-0.91 to -0.17)	-0.94 (-1.40 to -0.49)	-0.12 (-0.44 to 0.20)	-0.06 (-0.38 to 0.26)	-0.07 (-0.43 to 0.29)
Guatemala	1.87 (1.49 to 2.24)	2.49 (2.04 to 2.94)	1.29 (0.98 to 1.60)	0.97 (0.63 to 1.31)	1.40 (1.03 to 1.78)	0.64 (0.33 to 0.95)	1.23 (0.93 to 1.54)	1.71 (1.34 to 2.09)	0.84 (0.58 to 1.10)

Guinea	0.68 (0.59 to 0.76)	0.95 (0.82 to 1.08)	0.25 (0.20 to 0.30)	0.57 (0.48 to 0.66)	0.83 (0.69 to 0.97)	0.16 (0.11 to 0.22)	0.55 (0.45 to 0.64)	0.85 (0.70 to 1.00)	0.08 (0.03 to 0.13)
Guinea-Bissau	0.70 (0.62 to 0.78)	0.35 (0.26 to 0.44)	1.18 (1.09 to 1.26)	0.64 (0.56 to 0.72)	0.27 (0.19 to 0.35)	1.13 (1.04 to 1.23)	0.45 (0.37 to 0.52)	0.14 (0.06 to 0.21)	0.88 (0.78 to 0.97)
Guyana	1.21 (0.92 to 1.50)	1.25 (0.95 to 1.56)	1.17 (0.89 to 1.45)	0.76 (0.48 to 1.03)	0.79 (0.50 to 1.07)	0.74 (0.46 to 1.01)	0.88 (0.62 to 1.15)	0.95 (0.66 to 1.23)	0.82 (0.57 to 1.07)
Haiti	0.22 (0.15 to 0.29)	0.30 (0.21 to 0.40)	0.19 (0.06 to 0.32)	-0.09 (-0.16 to -0.02)	-0.01 (-0.11 to 0.09)	-0.11 (-0.23 to 0.01)	-0.12 (-0.19 to -0.05)	-0.02 (-0.14 to 0.09)	-0.18 (-0.30 to -0.06)
Honduras	1.93 (1.76 to 2.10)	2.28 (2.11 to 2.45)	1.63 (1.34 to 1.92)	1.50 (1.32 to 1.69)	1.70 (1.51 to 1.90)	1.37 (1.07 to 1.67)	1.17 (1.03 to 1.32)	1.36 (1.21 to 1.51)	1.02 (0.78 to 1.27)
Hungary	0.44 (0.13 to 0.75)	0.83 (0.51 to 1.15)	-0.12 (-0.46 to 0.21)	-0.63 (-0.87 to -0.39)	-0.22 (-0.47 to 0.03)	-1.17 (-1.43 to -0.90)	-0.53 (-0.76 to -0.30)	-0.17 (-0.41 to 0.07)	-1.06 (-1.32 to -0.79)
Iceland	-0.06 (-0.29 to 0.17)	0.18 (-0.11 to 0.48)	-0.39 (-0.59 to -0.19)	-1.02 (-1.15 to -0.88)	-0.86 (-1.07 to -0.66)	-1.24 (-1.37 to -1.12)	-1.20 (-1.30 to -1.09)	-0.96 (-1.12 to -0.80)	-1.56 (-1.67 to -1.44)
India	0.56 (0.39 to 0.73)	0.78 (0.64 to 0.92)	0.35 (0.14 to 0.56)	0.11 (-0.02 to 0.25)	0.34 (0.22 to 0.45)	-0.09 (-0.27 to 0.08)	-0.05 (-0.18 to 0.09)	0.18 (0.07 to 0.28)	-0.27 (-0.45 to -0.08)
Indonesia	1.33 (1.26 to 1.40)	1.85 (1.80 to 1.91)	0.80 (0.70 to 0.90)	0.98 (0.89 to 1.07)	1.50 (1.42 to 1.57)	0.47 (0.34 to 0.59)	0.72 (0.63 to 0.82)	1.27 (1.19 to 1.35)	0.15 (0.02 to 0.29)
Iran (Islamic Republic of Iran)	1.68 (1.45 to 1.92)	1.82 (1.59 to 2.05)	1.57 (1.32 to 1.82)	0.51 (0.30 to 0.73)	0.78 (0.56 to 1.00)	0.26 (0.02 to 0.49)	0.42 (0.19 to 0.64)	0.74 (0.51 to 0.96)	0.09 (-0.15 to 0.33)
Iraq	1.60 (1.32 to 1.87)	1.55 (1.27 to 1.83)	1.68 (1.39 to 1.97)	0.64 (0.42 to 0.85)	0.75 (0.53 to 0.97)	0.50 (0.28 to 0.72)	0.35 (0.19 to 0.51)	0.46 (0.30 to 0.63)	0.19 (0.01 to 0.37)
Ireland	0.00 (-0.12 to 0.13)	0.12 (0.00 to 0.24)	-0.29 (-0.44 to -0.14)	-1.72 (-1.81 to -1.64)	-1.61 (-1.70 to -1.51)	-2.01 (-2.10 to -1.92)	-1.84 (-1.92 to -1.75)	-1.70 (-1.81 to -1.60)	-2.15 (-2.23 to -2.06)
Israel	-0.83 (-1.20 to -0.45)	-0.57 (-0.95 to -0.19)	-1.13 (-1.51 to -0.75)	-2.13 (-2.45 to -1.81)	-1.93 (-2.26 to -1.61)	-2.37 (-2.70 to -2.04)	-2.12 (-2.43 to -1.81)	-1.85 (-2.18 to -1.53)	-2.46 (-2.77 to -2.15)
Italy	0.05 (-0.26 to 0.36)	0.15 (-0.20 to 0.50)	-0.18 (-0.45 to 0.09)	-1.10 (-1.22 to -0.98)	-0.96 (-1.12 to -0.80)	-1.35 (-1.44 to -1.27)	-1.22 (-1.36 to -1.07)	-1.10 (-1.28 to -0.92)	-1.45 (-1.56 to -1.34)
Jamaica	1.84 (1.44 to 2.23)	2.13 (1.60 to 2.66)	1.51 (1.23 to 1.80)	1.09 (0.74 to 1.43)	1.45 (0.97 to 1.93)	0.71 (0.44 to 0.98)	1.27 (0.87 to 1.67)	1.51 (0.98 to 2.05)	0.99 (0.70 to 1.29)
Japan	0.29 (0.19 to 0.39)	0.28 (0.17 to 0.39)	0.14 (0.06 to 0.22)	-0.64 (-0.70 to -0.57)	-0.69 (-0.75 to -0.64)	-0.76 (-0.84 to -0.68)	-0.77 (-0.84 to -0.71)	-0.76 (-0.83 to -0.70)	-0.93 (-1.01 to -0.85)
Jordan	0.64 (0.38 to 0.90)	0.63 (0.42 to 0.83)	0.68 (0.27 to 1.10)	-0.61 (-0.82 to -0.40)	-0.58 (-0.71 to -0.44)	-0.61 (-1.03 to -0.20)	-0.91 (-1.13 to -0.68)	-0.69 (-0.85 to -0.53)	-1.19 (-1.57 to -0.81)
Kazakhstan	-0.28 (-0.47 to -0.08)	-0.11 (-0.32 to 0.10)	-0.53 (-0.73 to -0.33)	-0.91 (-1.16 to -0.66)	-0.77 (-1.00 to -0.54)	-1.15 (-1.42 to -0.87)	-1.20 (-1.40 to -0.99)	-1.00 (-1.20 to -0.81)	-1.46 (-1.69 to -1.24)
Kenya	1.88 (1.68 to 2.09)	2.29 (2.01 to 2.57)	1.53 (1.29 to 1.78)	1.87 (1.63 to 2.11)	2.25 (1.95 to 2.55)	1.53 (1.26 to 1.80)	1.60 (1.38 to 1.83)	2.04 (1.72 to 2.37)	1.24 (1.01 to 1.47)
Kiribati	0.03 (-0.06 to 0.11)	-0.11 (-0.19 to -0.03)	0.24 (0.13 to 0.35)	-0.08 (-0.16 to 0.00)	-0.22 (-0.29 to -0.15)	0.15 (0.04 to 0.25)	-0.17 (-0.25 to -0.10)	-0.27 (-0.34 to -0.20)	0.02 (-0.07 to 0.11)
Kuwait	2.79 (2.27 to 3.30)	3.39 (2.89 to 3.88)	1.69 (1.01 to 2.38)	1.64 (1.15 to 2.13)	2.26 (1.78 to 2.75)	0.35 (-0.29 to 0.99)	1.40 (0.89 to 1.92)	2.11 (1.62 to 2.62)	0.05 (-0.62 to 0.73)
Kyrgyzstan	-0.77 (-1.02 to -0.52)	-0.68 (-0.98 to -0.38)	-0.90 (-1.14 to -0.66)	-1.18 (-1.39 to -0.97)	-1.09 (-1.35 to -0.82)	-1.31 (-1.52 to -1.11)	-1.42 (-1.58 to -1.26)	-1.32 (-1.52 to -1.12)	-1.55 (-1.72 to -1.38)
Lao People's Democratic Republic	0.18 (0.15 to 0.22)	0.48 (0.44 to 0.52)	-0.13 (-0.18 to -0.09)	-0.17 (-0.20 to -0.13)	0.15 (0.12 to 0.18)	-0.49 (-0.55 to -0.44)	-0.39 (-0.43 to -0.34)	-0.07 (-0.11 to -0.04)	-0.73 (-0.79 to -0.66)
Latvia	0.53 (0.39 to 0.68)	0.91 (0.75 to 1.08)	0.15 (-0.01 to 0.32)	-0.11 (-0.28 to 0.06)	0.30 (0.11 to 0.48)	-0.50 (-0.71 to -0.30)	-0.43 (-0.59 to -0.27)	-0.05 (-0.21 to 0.12)	-0.84 (-1.03 to -0.66)
Lebanon	1.13 (0.88 to 1.39)	1.72 (1.36 to 2.07)	0.47 (0.26 to 0.69)	-0.21 (-0.45 to 0.03)	0.45 (0.12 to 0.78)	-1.07 (-1.28 to -0.87)	-0.43 (-0.65 to -0.21)	0.30 (-0.03 to 0.64)	-1.34 (-1.50 to -1.18)
Lesotho	3.02 (2.58 to 3.46)	2.55 (2.25 to 2.86)	3.30 (2.73 to 3.87)	2.96 (2.51 to 3.41)	2.48 (2.16 to 2.80)	3.23 (2.66 to 3.80)	3.20 (2.70 to 3.69)	2.75 (2.37 to 3.13)	3.49 (2.86 to 4.12)
Liberia	0.89 (0.67 to 1.12)	0.67 (0.39 to 0.95)	1.17 (1.01 to 1.34)	0.63 (0.41 to 0.84)	0.38 (0.11 to 0.65)	0.93 (0.76 to 1.09)	0.55 (0.33 to 0.78)	0.36 (0.07 to 0.65)	0.80 (0.63 to 0.96)
Libya	1.32 (1.06 to 1.57)	1.28 (1.03 to 1.52)	1.34 (1.07 to 1.62)	0.50 (0.32 to 0.69)	0.61 (0.41 to 0.80)	0.39 (0.19 to 0.58)	0.42 (0.26 to 0.58)	0.56 (0.38 to 0.74)	0.26 (0.10 to 0.41)
Lithuania	0.56 (0.42 to 0.70)	1.04 (0.87 to 1.21)	0.07 (-0.06 to 0.20)	0.08 (-0.08 to 0.23)	0.58 (0.39 to 0.77)	-0.37 (-0.52 to -0.22)	-0.20 (-0.37 to -0.02)	0.26 (0.06 to 0.47)	-0.68 (-0.85 to -0.52)
Luxembourg	-0.48 (-0.80 to -0.17)	-0.51 (-0.86 to -0.17)	-0.61 (-0.94 to -0.29)	-1.91 (-2.08 to -1.74)	-1.90 (-2.12 to -1.68)	-2.04 (-2.21 to -1.87)	-2.09 (-2.27 to -1.90)	-2.11 (-2.32 to -1.89)	-2.21 (-2.41 to -2.01)
Madagascar	0.18 (0.06 to 0.30)	0.56 (0.43 to 0.70)	-0.18 (-0.30 to -0.05)	0.11 (0.00 to 0.23)	0.51 (0.38 to 0.64)	-0.25 (-0.36 to -0.13)	-0.06 (-0.17 to 0.06)	0.33 (0.20 to 0.46)	-0.41 (-0.53 to -0.28)
Malawi	0.40 (0.24 to 0.57)	0.86 (0.63 to 1.08)	0.00 (-0.12 to 0.12)	0.23 (0.06 to 0.40)	0.66 (0.43 to 0.90)	-0.15 (-0.27 to -0.02)	0.17 (-0.03 to 0.36)	0.66 (0.40 to 0.93)	-0.30 (-0.44 to -0.17)
Malaysia	0.89 (0.76 to 1.02)	1.02 (0.85 to 1.18)	0.70 (0.60 to 0.81)	0.10 (-0.04 to 0.23)	0.15 (-0.03 to 0.32)	0.00 (-0.10 to 0.10)	-0.01 (-0.18 to 0.15)	0.17 (-0.02 to 0.36)	-0.27 (-0.43 to -0.12)
Maldives	-0.86 (-0.95 to -0.77)	-0.37 (-0.48 to -0.27)	-1.47 (-1.60 to -1.34)	-2.01 (-2.13 to -1.89)	-1.50 (-1.60 to -1.40)	-2.65 (-2.83 to -2.47)	-2.49 (-2.63 to -2.35)	-1.94 (-2.06 to -1.83)	-3.18 (-3.37 to -2.99)
Mali	0.41 (0.30 to 0.52)	0.60 (0.43 to 0.77)	0.08 (0.04 to 0.12)	0.28 (0.18 to 0.39)	0.45 (0.28 to 0.62)	-0.04 (-0.08 to 0.00)	0.16 (0.05 to 0.27)	0.39 (0.22 to 0.57)	-0.20 (-0.25 to -0.16)
Malta	0.15 (0.02 to 0.28)	0.11 (-0.09 to 0.31)	0.12 (-0.01 to 0.25)	-1.12 (-1.23 to -1.01)	-1.07 (-1.23 to -0.92)	-1.25 (-1.41 to -1.08)	-1.14 (-1.23 to -1.04)	-1.10 (-1.24 to -0.95)	-1.28 (-1.41 to -1.14)
Marshall Islands	0.30 (0.24 to 0.36)	0.24 (0.12 to 0.36)	0.35 (0.22 to 0.48)	0.10 (0.04 to 0.17)	0.04 (-0.07 to 0.15)	0.14 (0.00 to 0.29)	0.12 (0.05 to 0.20)	0.08 (-0.02 to 0.17)	0.17 (0.01 to 0.33)
Mauritania	0.66 (0.47 to 0.86)	0.48 (0.16 to 0.80)	0.84 (0.74 to 0.93)	0.35 (0.18 to 0.52)	0.16 (-0.13 to 0.46)	0.53 (0.45 to 0.62)	0.11 (-0.06 to 0.28)	-0.07 (-0.37 to 0.23)	0.27 (0.19 to 0.35)

Mauritius	1.77 (1.55 to 1.99)	2.15 (1.82 to 2.48)	1.29 (1.08 to 1.51)	1.18 (0.98 to 1.38)	1.53 (1.24 to 1.83)	0.75 (0.52 to 0.97)	1.24 (1.02 to 1.45)	1.64 (1.35 to 1.94)	0.70 (0.47 to 0.94)
Mexico	2.28 (2.18 to 2.38)	2.94 (2.83 to 3.06)	1.35 (1.25 to 1.44)	1.13 (0.98 to 1.28)	1.77 (1.59 to 1.95)	0.44 (0.30 to 0.58)	1.55 (1.42 to 1.68)	2.05 (1.90 to 2.21)	0.99 (0.88 to 1.11)
Micronesia (Federated States of Micronesia)	0.22 (0.17 to 0.26)	0.27 (0.24 to 0.30)	0.15 (0.09 to 0.21)	-0.11 (-0.14 to -0.07)	-0.05 (-0.08 to -0.03)	-0.17 (-0.22 to -0.13)	-0.14 (-0.18 to -0.10)	-0.06 (-0.08 to -0.03)	-0.23 (-0.29 to -0.17)
Monaco	1.10 (0.93 to 1.26)	1.10 (0.94 to 1.26)	0.97 (0.80 to 1.14)	0.26 (0.16 to 0.37)	0.26 (0.16 to 0.37)	0.13 (0.03 to 0.23)	0.22 (0.11 to 0.33)	0.25 (0.14 to 0.35)	0.07 (-0.04 to 0.18)
Mongolia	0.55 (0.40 to 0.71)	1.01 (0.87 to 1.15)	0.11 (-0.07 to 0.29)	0.02 (-0.12 to 0.16)	0.43 (0.31 to 0.54)	-0.38 (-0.55 to -0.21)	-0.15 (-0.29 to 0.00)	0.39 (0.27 to 0.52)	-0.68 (-0.86 to -0.51)
Montenegro	1.32 (1.19 to 1.46)	1.35 (1.11 to 1.59)	1.19 (1.06 to 1.31)	0.90 (0.77 to 1.04)	0.85 (0.62 to 1.08)	0.87 (0.70 to 1.03)	0.56 (0.45 to 0.67)	0.59 (0.46 to 0.73)	0.43 (0.20 to 0.66)
Morocco	1.98 (1.86 to 2.11)	2.03 (1.82 to 2.24)	1.96 (1.85 to 2.06)	1.32 (1.21 to 1.43)	1.55 (1.38 to 1.73)	1.11 (1.00 to 1.22)	1.16 (1.06 to 1.26)	1.31 (1.14 to 1.47)	1.05 (0.93 to 1.16)
Mozambique	1.35 (1.20 to 1.50)	1.81 (1.62 to 2.00)	0.93 (0.75 to 1.10)	1.25 (1.09 to 1.42)	1.68 (1.49 to 1.88)	0.88 (0.70 to 1.06)	1.29 (1.12 to 1.47)	1.81 (1.59 to 2.03)	0.76 (0.58 to 0.93)
Myanmar	0.35 (0.30 to 0.41)	1.09 (1.05 to 1.13)	-0.30 (-0.41 to -0.19)	-0.08 (-0.15 to -0.01)	0.66 (0.64 to 0.68)	-0.72 (-0.84 to -0.59)	-0.33 (-0.42 to -0.25)	0.47 (0.44 to 0.50)	-1.06 (-1.20 to -0.91)
Namibia	0.70 (0.49 to 0.91)	1.18 (0.95 to 1.40)	0.26 (0.07 to 0.45)	0.38 (0.15 to 0.61)	0.84 (0.60 to 1.09)	-0.03 (-0.25 to 0.18)	0.30 (0.03 to 0.57)	0.80 (0.50 to 1.10)	-0.21 (-0.44 to 0.03)
Nauru	-0.09 (-0.19 to 0.01)	0.00 (-0.14 to 0.13)	-0.02 (-0.08 to 0.03)	-0.32 (-0.37 to -0.28)	-0.22 (-0.31 to -0.14)	-0.25 (-0.27 to -0.22)	-0.33 (-0.37 to -0.29)	-0.23 (-0.30 to -0.16)	-0.26 (-0.30 to -0.22)
Nepal	0.53 (0.17 to 0.89)	0.89 (0.52 to 1.27)	0.17 (-0.18 to 0.52)	0.14 (-0.21 to 0.49)	0.52 (0.16 to 0.87)	-0.23 (-0.58 to 0.11)	-0.03 (-0.39 to 0.32)	0.35 (-0.02 to 0.71)	-0.41 (-0.75 to -0.06)
Netherlands	-0.07 (-0.31 to 0.16)	-0.03 (-0.28 to 0.21)	-0.28 (-0.52 to -0.05)	-0.50 (-0.68 to -0.32)	-0.41 (-0.60 to -0.23)	-0.74 (-0.92 to -0.56)	-0.51 (-0.69 to -0.32)	-0.43 (-0.62 to -0.23)	-0.73 (-0.92 to -0.54)
New Zealand	-0.64 (-0.71 to -0.57)	-0.55 (-0.64 to -0.46)	-0.80 (-0.92 to -0.68)	-1.71 (-1.80 to -1.62)	-1.67 (-1.81 to -1.54)	-1.82 (-1.91 to -1.74)	-2.05 (-2.15 to -1.95)	-1.97 (-2.13 to -1.82)	-2.18 (-2.26 to -2.10)
Nicaragua	2.27 (1.98 to 2.57)	2.69 (2.38 to 3.00)	1.76 (1.50 to 2.02)	1.27 (0.98 to 1.56)	1.38 (1.08 to 1.67)	1.09 (0.81 to 1.36)	1.26 (1.00 to 1.52)	1.58 (1.29 to 1.88)	0.93 (0.70 to 1.16)
Niger	0.65 (0.54 to 0.76)	0.51 (0.33 to 0.69)	0.82 (0.76 to 0.87)	0.57 (0.47 to 0.68)	0.41 (0.24 to 0.58)	0.77 (0.71 to 0.82)	0.34 (0.24 to 0.44)	0.24 (0.06 to 0.41)	0.46 (0.42 to 0.51)
Nigeria	0.67 (0.59 to 0.76)	0.65 (0.55 to 0.76)	0.73 (0.65 to 0.81)	0.49 (0.41 to 0.57)	0.44 (0.35 to 0.54)	0.55 (0.47 to 0.62)	0.31 (0.23 to 0.38)	0.33 (0.25 to 0.42)	0.35 (0.28 to 0.42)
Niue	0.52 (0.49 to 0.56)	0.69 (0.65 to 0.73)	0.33 (0.27 to 0.39)	0.08 (0.05 to 0.11)	0.26 (0.23 to 0.29)	-0.12 (-0.17 to -0.07)	0.00 (-0.04 to 0.04)	0.16 (0.12 to 0.20)	-0.20 (-0.27 to -0.13)
North Macedonia	1.37 (0.99 to 1.74)	1.50 (1.09 to 1.91)	1.22 (0.85 to 1.58)	0.61 (0.25 to 0.97)	0.71 (0.32 to 1.10)	0.53 (0.18 to 0.89)	0.28 (-0.03 to 0.59)	0.43 (0.09 to 0.77)	0.10 (-0.19 to 0.38)
Northern Mariana Islands	0.25 (0.07 to 0.43)	0.69 (0.61 to 0.77)	-0.29 (-0.67 to 0.08)	0.00 (-0.23 to 0.23)	0.44 (0.30 to 0.57)	-0.53 (-0.96 to -0.11)	-0.02 (-0.26 to 0.23)	0.40 (0.26 to 0.54)	-0.55 (-1.00 to -0.10)
Norway	0.34 (0.17 to 0.52)	0.28 (0.11 to 0.45)	0.29 (0.09 to 0.49)	-0.98 (-1.07 to -0.89)	-1.14 (-1.23 to -1.05)	-0.94 (-1.05 to -0.83)	-1.22 (-1.31 to -1.12)	-1.27 (-1.36 to -1.19)	-1.27 (-1.41 to -1.14)
Oman	1.02 (0.80 to 1.25)	1.33 (1.12 to 1.55)	0.60 (0.39 to 0.82)	-0.01 (-0.24 to 0.22)	0.46 (0.22 to 0.69)	-0.72 (-0.92 to -0.52)	-0.35 (-0.62 to -0.09)	0.06 (-0.20 to 0.32)	-1.00 (-1.24 to -0.76)
Pakistan	0.66 (0.45 to 0.86)	0.86 (0.64 to 1.07)	0.43 (0.24 to 0.63)	0.46 (0.23 to 0.69)	0.66 (0.42 to 0.89)	0.24 (0.02 to 0.46)	0.40 (0.15 to 0.64)	0.61 (0.36 to 0.86)	0.16 (-0.07 to 0.39)
Palau	0.05 (-0.02 to 0.12)	-0.02 (-0.07 to 0.03)	0.24 (0.19 to 0.30)	-0.20 (-0.30 to -0.10)	-0.43 (-0.49 to -0.38)	0.10 (0.02 to 0.17)	-0.43 (-0.52 to -0.34)	-0.29 (-0.32 to -0.25)	-0.31 (-0.37 to -0.25)
Palestine	0.24 (0.13 to 0.35)	0.21 (0.03 to 0.38)	0.36 (0.24 to 0.49)	-0.54 (-0.71 to -0.38)	-0.42 (-0.67 to -0.17)	-0.56 (-0.72 to -0.41)	-0.65 (-0.78 to -0.52)	-0.53 (-0.71 to -0.35)	-0.74 (-0.87 to -0.60)
Panama	2.33 (2.25 to 2.41)	2.75 (2.64 to 2.86)	1.60 (1.49 to 1.71)	1.08 (0.99 to 1.17)	1.34 (1.22 to 1.47)	0.83 (0.71 to 0.95)	1.23 (1.12 to 1.33)	1.46 (1.32 to 1.59)	1.00 (0.88 to 1.11)
Papua New Guinea	-0.29 (-0.38 to -0.20)	-0.17 (-0.29 to -0.04)	-0.36 (-0.42 to -0.29)	-0.36 (-0.44 to -0.28)	-0.23 (-0.35 to -0.12)	-0.44 (-0.49 to -0.39)	-0.44 (-0.53 to -0.35)	-0.31 (-0.44 to -0.18)	-0.51 (-0.57 to -0.45)
Paraguay	2.59 (2.41 to 2.76)	3.15 (2.96 to 3.35)	2.02 (1.85 to 2.19)	2.07 (1.89 to 2.25)	2.64 (2.45 to 2.83)	1.54 (1.36 to 1.72)	1.99 (1.81 to 2.18)	2.55 (2.35 to 2.76)	1.43 (1.25 to 1.62)
Peru	1.50 (1.31 to 1.69)	1.72 (1.51 to 1.93)	1.32 (1.11 to 1.53)	0.26 (0.07 to 0.45)	0.44 (0.25 to 0.63)	0.13 (-0.09 to 0.34)	0.19 (-0.01 to 0.38)	0.36 (0.15 to 0.56)	0.04 (-0.17 to 0.25)
Philippines	1.32 (1.27 to 1.37)	1.55 (1.50 to 1.61)	1.07 (1.00 to 1.14)	1.06 (0.98 to 1.13)	1.35 (1.28 to 1.43)	0.74 (0.63 to 0.84)	1.02 (0.96 to 1.09)	1.17 (1.10 to 1.24)	0.88 (0.80 to 0.96)
Poland	1.11 (0.90 to 1.32)	1.56 (1.32 to 1.81)	0.54 (0.34 to 0.73)	0.19 (0.03 to 0.36)	0.74 (0.55 to 0.94)	-0.42 (-0.57 to -0.27)	0.03 (-0.13 to 0.19)	0.48 (0.29 to 0.68)	-0.58 (-0.72 to -0.44)
Portugal	0.94 (0.60 to 1.27)	1.24 (0.87 to 1.61)	0.46 (0.17 to 0.75)	-0.88 (-1.07 to -0.70)	-0.56 (-0.78 to -0.34)	-1.38 (-1.52 to -1.24)	-0.77 (-0.99 to -0.55)	-0.48 (-0.73 to -0.22)	-1.23 (-1.41 to -1.05)
Puerto Rico	1.61 (1.30 to 1.91)	1.90 (1.56 to 2.23)	1.23 (0.92 to 1.53)	-0.27 (-0.48 to -0.06)	0.02 (-0.23 to 0.28)	-0.61 (-0.82 to -0.41)	0.00 (-0.18 to 0.18)	0.23 (0.02 to 0.43)	-0.32 (-0.52 to -0.12)
Qatar	1.14 (0.55 to 1.72)	1.21 (0.47 to 1.95)	1.12 (0.65 to 1.58)	-0.62 (-1.31 to 0.07)	-0.43 (-1.27 to 0.42)	-0.86 (-1.42 to -0.29)	-0.65 (-1.26 to -0.03)	-0.40 (-1.15 to 0.35)	-1.03 (-1.50 to -0.56)
Republic of Korea	2.10 (1.71 to 2.50)	2.35 (1.93 to 2.77)	1.47 (1.11 to 1.83)	-0.54 (-0.70 to -0.38)	-0.38 (-0.57 to -0.20)	-1.00 (-1.15 to -0.85)	-0.87 (-1.05 to -0.69)	-0.61 (-0.83 to -0.40)	-1.43 (-1.58 to -1.29)
Republic of Moldova	1.30 (0.81 to 1.78)	1.77 (1.27 to 2.27)	0.60 (0.14 to 1.06)	0.46 (0.00 to 0.93)	0.88 (0.42 to 1.34)	-0.20 (-0.66 to 0.26)	0.44 (0.02 to 0.86)	0.98 (0.55 to 1.42)	-0.35 (-0.76 to 0.06)
Romania	2.63 (2.39 to 2.88)	3.12 (2.84 to 3.40)	2.06 (1.86 to 2.27)	1.42 (1.19 to 1.64)	1.99 (1.72 to 2.25)	0.75 (0.56 to 0.93)	1.17 (0.94 to 1.39)	1.68 (1.42 to 1.95)	0.49 (0.30 to 0.69)
Russian Federation	1.07 (0.93 to 1.22)	1.07 (0.92 to 1.22)	0.91 (0.77 to 1.05)	0.12 (-0.04 to 0.28)	0.05 (-0.13 to 0.24)	-0.04 (-0.19 to 0.10)	-0.21 (-0.39 to -0.03)	-0.10 (-0.29 to 0.09)	-0.46 (-0.63 to -0.29)

Rwanda	-0.97 (-1.28 to -0.66)	-0.46 (-0.77 to -0.16)	-1.35 (-1.66 to -1.05)	-1.14 (-1.43 to -0.85)	-0.60 (-0.88 to -0.32)	-1.53 (-1.82 to -1.24)	-1.60 (-1.93 to -1.27)	-1.13 (-1.46 to -0.80)	-1.99 (-2.31 to -1.67)
Saint Kitts and Nevis	1.66 (1.46 to 1.86)	1.96 (1.79 to 2.13)	1.10 (0.79 to 1.41)	0.54 (0.37 to 0.71)	0.65 (0.49 to 0.82)	0.15 (-0.12 to 0.42)	0.28 (0.12 to 0.44)	0.65 (0.45 to 0.86)	-0.36 (-0.56 to -0.16)
Saint Lucia	0.25 (0.12 to 0.39)	0.72 (0.60 to 0.84)	-0.31 (-0.50 to -0.11)	-0.78 (-1.01 to -0.55)	-0.32 (-0.52 to -0.13)	-1.30 (-1.58 to -1.01)	-0.55 (-0.73 to -0.38)	-0.10 (-0.24 to 0.04)	-1.10 (-1.34 to -0.86)
Saint Vincent and the Grenadines	1.00 (0.86 to 1.13)	1.31 (1.18 to 1.44)	0.65 (0.47 to 0.84)	0.44 (0.29 to 0.59)	0.79 (0.65 to 0.94)	0.09 (-0.11 to 0.30)	0.47 (0.34 to 0.60)	0.76 (0.62 to 0.90)	0.14 (-0.03 to 0.31)
Samoa	0.28 (0.22 to 0.34)	-0.08 (-0.15 to -0.01)	0.56 (0.50 to 0.63)	-0.10 (-0.17 to -0.04)	-0.43 (-0.51 to -0.36)	0.16 (0.09 to 0.22)	-0.02 (-0.10 to 0.06)	-0.38 (-0.48 to -0.28)	0.26 (0.19 to 0.34)
San Marino	-0.75 (-1.14 to -0.36)	-0.65 (-1.11 to -0.19)	-1.00 (-1.29 to -0.70)	-1.51 (-1.87 to -1.15)	-1.36 (-1.82 to -0.91)	-1.88 (-2.13 to -1.63)	-1.41 (-1.75 to -1.08)	-1.34 (-1.73 to -0.94)	-1.61 (-1.87 to -1.35)
Sao Tome and Principe	1.40 (1.34 to 1.46)	1.48 (1.30 to 1.66)	0.84 (0.72 to 0.96)	1.13 (1.05 to 1.20)	1.14 (0.95 to 1.34)	0.58 (0.45 to 0.72)	0.94 (0.86 to 1.01)	1.07 (0.96 to 1.19)	0.36 (0.18 to 0.55)
Saudi Arabia	2.79 (2.46 to 3.11)	2.53 (2.23 to 2.82)	3.08 (2.71 to 3.46)	1.08 (0.72 to 1.44)	1.12 (0.78 to 1.47)	0.81 (0.38 to 1.23)	1.18 (0.84 to 1.53)	1.26 (0.93 to 1.59)	0.90 (0.49 to 1.31)
Senegal	0.99 (0.84 to 1.14)	0.89 (0.69 to 1.08)	1.17 (1.06 to 1.28)	0.87 (0.73 to 1.02)	0.75 (0.56 to 0.95)	1.07 (0.96 to 1.18)	0.71 (0.55 to 0.87)	0.63 (0.43 to 0.84)	0.86 (0.74 to 0.99)
Serbia	0.59 (0.46 to 0.72)	1.03 (0.87 to 1.19)	-0.08 (-0.23 to 0.07)	-0.59 (-0.68 to -0.49)	-0.11 (-0.22 to 0.00)	-1.18 (-1.34 to -1.02)	-0.53 (-0.66 to -0.41)	-0.14 (-0.27 to -0.01)	-1.05 (-1.21 to -0.89)
Seychelles	1.31 (1.04 to 1.59)	1.14 (0.80 to 1.48)	1.42 (1.19 to 1.66)	0.75 (0.51 to 1.00)	0.59 (0.28 to 0.91)	0.85 (0.64 to 1.07)	0.57 (0.34 to 0.81)	0.41 (0.10 to 0.71)	0.68 (0.50 to 0.85)
Sierra Leone	1.04 (0.90 to 1.17)	0.55 (0.37 to 0.73)	1.65 (1.48 to 1.81)	0.91 (0.77 to 1.05)	0.42 (0.25 to 0.59)	1.50 (1.32 to 1.69)	0.89 (0.75 to 1.04)	0.40 (0.22 to 0.58)	1.52 (1.32 to 1.71)
Singapore	-0.10 (-0.26 to 0.07)	-0.11 (-0.30 to 0.09)	-0.15 (-0.30 to 0.01)	-2.03 (-2.23 to -1.82)	-2.04 (-2.26 to -1.82)	-2.05 (-2.25 to -1.85)	-2.18 (-2.40 to -1.97)	-2.21 (-2.44 to -1.98)	-2.20 (-2.40 to -2.00)
Slovakia	0.81 (0.72 to 0.91)	1.01 (0.86 to 1.16)	0.57 (0.51 to 0.64)	-0.30 (-0.39 to -0.20)	-0.03 (-0.18 to 0.12)	-0.58 (-0.65 to -0.51)	-0.49 (-0.59 to -0.39)	-0.30 (-0.46 to -0.15)	-0.76 (-0.83 to -0.68)
Slovenia	0.23 (-0.11 to 0.58)	0.52 (0.15 to 0.89)	-0.41 (-0.76 to -0.06)	-1.09 (-1.39 to -0.80)	-0.70 (-1.03 to -0.37)	-1.72 (-2.01 to -1.43)	-1.44 (-1.75 to -1.14)	-1.17 (-1.50 to -0.84)	-2.06 (-2.36 to -1.76)
Solomon Islands	0.26 (0.13 to 0.40)	0.17 (0.04 to 0.29)	0.34 (0.19 to 0.50)	0.07 (-0.05 to 0.19)	-0.02 (-0.13 to 0.08)	0.14 (0.01 to 0.27)	0.11 (-0.02 to 0.25)	0.03 (-0.09 to 0.14)	0.20 (0.04 to 0.37)
Somalia	0.19 (0.13 to 0.24)	0.45 (0.36 to 0.55)	0.00 (-0.03 to 0.03)	0.21 (0.14 to 0.27)	0.47 (0.37 to 0.58)	0.02 (-0.02 to 0.06)	0.04 (-0.02 to 0.09)	0.30 (0.21 to 0.39)	-0.16 (-0.19 to -0.13)
South Africa	1.33 (1.12 to 1.54)	1.42 (1.12 to 1.71)	1.30 (1.16 to 1.45)	0.94 (0.68 to 1.21)	1.09 (0.75 to 1.43)	0.88 (0.68 to 1.07)	0.98 (0.73 to 1.23)	0.99 (0.64 to 1.33)	1.02 (0.83 to 1.22)
South Sudan	0.76 (0.63 to 0.88)	1.00 (0.88 to 1.12)	0.48 (0.32 to 0.65)	0.67 (0.56 to 0.78)	0.91 (0.80 to 1.02)	0.39 (0.24 to 0.54)	0.55 (0.40 to 0.70)	0.80 (0.66 to 0.93)	0.29 (0.09 to 0.48)
Spain	0.88 (0.65 to 1.10)	1.17 (0.91 to 1.44)	0.36 (0.18 to 0.55)	-0.50 (-0.63 to -0.37)	-0.14 (-0.30 to 0.02)	-1.06 (-1.15 to -0.97)	-0.59 (-0.73 to -0.46)	-0.28 (-0.44 to -0.11)	-1.13 (-1.22 to -1.03)
Sri Lanka	1.35 (1.17 to 1.52)	1.63 (1.42 to 1.85)	1.13 (0.96 to 1.30)	0.08 (-0.09 to 0.25)	0.43 (0.20 to 0.65)	-0.17 (-0.32 to -0.02)	0.07 (-0.11 to 0.24)	0.35 (0.12 to 0.58)	-0.15 (-0.31 to 0.00)
Sudan	0.94 (0.85 to 1.03)	1.03 (0.92 to 1.14)	0.86 (0.79 to 0.92)	0.37 (0.30 to 0.44)	0.59 (0.50 to 0.68)	0.14 (0.10 to 0.19)	0.27 (0.20 to 0.34)	0.49 (0.41 to 0.57)	0.06 (0.02 to 0.11)
Suriname	1.19 (0.96 to 1.42)	1.61 (1.37 to 1.86)	0.75 (0.53 to 0.97)	0.63 (0.42 to 0.84)	1.03 (0.80 to 1.26)	0.22 (0.03 to 0.42)	0.59 (0.39 to 0.79)	0.99 (0.77 to 1.21)	0.15 (-0.04 to 0.35)
Sweden	0.10 (-0.15 to 0.35)	0.01 (-0.27 to 0.28)	0.10 (-0.12 to 0.33)	-0.78 (-0.94 to -0.61)	-0.94 (-1.12 to -0.75)	-0.72 (-0.86 to -0.57)	-0.91 (-1.08 to -0.75)	-1.00 (-1.19 to -0.81)	-0.91 (-1.06 to -0.75)
Switzerland	-0.39 (-0.69 to -0.09)	-0.46 (-0.82 to -0.10)	-0.44 (-0.68 to -0.19)	-1.29 (-1.46 to -1.12)	-1.33 (-1.55 to -1.11)	-1.38 (-1.52 to -1.24)	-1.49 (-1.67 to -1.31)	-1.55 (-1.78 to -1.32)	-1.53 (-1.67 to -1.39)
Syrian Arab Republic	0.82 (0.67 to 0.97)	0.89 (0.72 to 1.05)	0.78 (0.64 to 0.91)	-0.22 (-0.37 to -0.07)	0.01 (-0.16 to 0.17)	-0.37 (-0.50 to -0.24)	-0.53 (-0.71 to -0.34)	-0.31 (-0.49 to -0.13)	-0.72 (-0.90 to -0.55)
Taiwan (Province of China)	1.74 (1.31 to 2.16)	2.20 (1.78 to 2.61)	1.25 (0.80 to 1.70)	0.48 (0.15 to 0.80)	0.92 (0.62 to 1.22)	0.03 (-0.31 to 0.38)	0.21 (-0.05 to 0.47)	0.65 (0.38 to 0.91)	-0.25 (-0.51 to 0.01)
Tajikistan	-1.52 (-1.75 to -1.30)	-1.61 (-1.87 to -1.35)	-1.58 (-1.77 to -1.39)	-1.58 (-1.80 to -1.35)	-1.68 (-1.93 to -1.43)	-1.64 (-1.83 to -1.44)	-1.83 (-2.04 to -1.62)	-1.88 (-2.13 to -1.63)	-1.88 (-2.05 to -1.71)
Thailand	1.42 (1.29 to 1.55)	1.86 (1.75 to 1.98)	0.80 (0.62 to 0.97)	0.11 (-0.02 to 0.24)	0.47 (0.34 to 0.59)	-0.40 (-0.58 to -0.23)	0.26 (0.12 to 0.41)	0.75 (0.62 to 0.88)	-0.45 (-0.64 to -0.25)
Timor-Leste	0.81 (0.61 to 1.01)	1.34 (1.12 to 1.56)	0.34 (0.16 to 0.53)	0.45 (0.26 to 0.63)	0.98 (0.78 to 1.18)	-0.03 (-0.19 to 0.14)	0.32 (0.10 to 0.54)	0.86 (0.63 to 1.09)	-0.16 (-0.37 to 0.05)
Togo	1.17 (1.10 to 1.25)	1.29 (1.21 to 1.37)	1.19 (1.08 to 1.30)	1.06 (0.98 to 1.14)	1.14 (1.04 to 1.23)	1.10 (1.00 to 1.20)	0.95 (0.87 to 1.02)	1.10 (0.99 to 1.21)	0.91 (0.81 to 1.02)
Tokelau	0.21 (0.18 to 0.25)	0.38 (0.34 to 0.43)	0.08 (0.05 to 0.12)	-0.33 (-0.35 to -0.31)	-0.15 (-0.18 to -0.12)	-0.48 (-0.50 to -0.46)	-0.35 (-0.39 to -0.31)	-0.15 (-0.19 to -0.12)	-0.50 (-0.54 to -0.46)
Tonga	0.37 (0.26 to 0.49)	0.67 (0.45 to 0.89)	0.13 (0.08 to 0.19)	0.13 (0.01 to 0.25)	0.34 (0.11 to 0.57)	-0.06 (-0.12 to 0.00)	-0.02 (-0.12 to 0.09)	0.39 (0.19 to 0.58)	-0.33 (-0.38 to -0.28)
Trinidad and Tobago	1.01 (0.88 to 1.14)	1.34 (1.17 to 1.50)	0.67 (0.52 to 0.81)	-0.22 (-0.35 to -0.09)	0.15 (-0.02 to 0.32)	-0.60 (-0.75 to -0.46)	0.03 (-0.10 to 0.16)	0.36 (0.19 to 0.53)	-0.35 (-0.50 to -0.20)
Trkiye	0.80 (0.74 to 0.85)	0.93 (0.85 to 1.02)	0.70 (0.66 to 0.73)	-0.35 (-0.42 to -0.27)	-0.05 (-0.15 to 0.05)	-0.65 (-0.71 to -0.60)	-0.27 (-0.34 to -0.20)	0.05 (-0.05 to 0.14)	-0.61 (-0.65 to -0.57)
Tunisia	-0.30 (-0.84 to 0.24)	-0.19 (-0.71 to 0.33)	-0.48 (-1.05 to 0.09)	-0.66 (-1.17 to -0.16)	-0.54 (-1.03 to -0.06)	-0.86 (-1.39 to -0.31)	-0.69 (-1.19 to -0.19)	-0.57 (-1.06 to -0.08)	-0.87 (-1.40 to -0.34)
Turkmenistan	0.14 (0.11 to 0.17)	0.30 (0.28 to 0.32)	0.02 (-0.03 to 0.06)	-0.21 (-0.23 to -0.19)	-0.06 (-0.07 to -0.04)	-0.33 (-0.37 to -0.30)	-0.28 (-0.31 to -0.25)	-0.09 (-0.11 to -0.08)	-0.45 (-0.49 to -0.40)
Tuvalu	0.63 (0.29 to 0.97)	0.69 (0.42 to 0.97)	0.50 (0.13 to 0.87)	-0.82 (-1.17 to -0.46)	-0.68 (-0.94 to -0.43)	-1.08 (-1.51 to -0.66)	-1.12 (-1.45 to -0.79)	-0.81 (-1.09 to -0.53)	-1.60 (-1.96 to -1.23)

Uganda	-0.15<br>(-0.37 to 0.08)	0.04<br>(-0.14 to 0.22)	-0.21<br>(-0.51 to 0.08)	-0.32<br>(-0.55 to -0.09)	-0.13<br>(-0.32 to 0.06)	-0.38<br>(-0.68 to -0.08)	-0.39<br>(-0.65 to -0.12)	-0.20<br>(-0.43 to 0.02)	-0.48<br>(-0.81 to -0.14)
Ukraine	-0.72<br>(-0.84 to -0.60)	-0.46<br>(-0.59 to -0.33)	-0.99<br>(-1.12 to -0.87)	-1.06<br>(-1.20 to -0.91)	-0.79<br>(-0.96 to -0.63)	-1.38<br>(-1.52 to -1.24)	-1.21<br>(-1.37 to -1.05)	-0.97<br>(-1.14 to -0.79)	-1.48<br>(-1.62 to -1.34)
United Arab Emirates	1.20<br>(0.69 to 1.72)	-0.34<br>(-0.88 to 0.21)	3.33<br>(2.68 to 3.98)	0.79<br>(0.22 to 1.36)	-0.75<br>(-1.35 to -0.15)	3.21<br>(2.49 to 3.93)	-0.17<br>(-0.62 to 0.29)	-1.39<br>(-1.92 to -0.86)	1.68<br>(1.13 to 2.23)
United Kingdom	-0.24<br>(-0.31 to -0.17)	-0.37<br>(-0.46 to -0.28)	-0.22<br>(-0.31 to -0.13)	-1.45<br>(-1.56 to -1.34)	-1.58<br>(-1.66 to -1.51)	-1.44<br>(-1.61 to -1.28)	-1.56<br>(-1.67 to -1.45)	-1.67<br>(-1.75 to -1.59)	-1.54<br>(-1.71 to -1.37)
United Republic of Tanzania	0.39<br>(0.33 to 0.45)	0.61<br>(0.53 to 0.68)	0.18<br>(0.08 to 0.27)	0.28<br>(0.23 to 0.33)	0.51<br>(0.44 to 0.59)	0.07<br>(-0.01 to 0.15)	0.12<br>(0.07 to 0.18)	0.34<br>(0.27 to 0.42)	-0.11<br>(-0.20 to -0.02)
United States of America	-0.90<br>(-1.03 to -0.77)	-1.00<br>(-1.12 to -0.88)	-0.90<br>(-1.04 to -0.75)	-1.71<br>(-1.79 to -1.63)	-1.83<br>(-1.90 to -1.75)	-1.72<br>(-1.81 to -1.64)	-1.40<br>(-1.46 to -1.33)	-1.45<br>(-1.52 to -1.38)	-1.45<br>(-1.52 to -1.38)
United States Virgin Islands	-0.50<br>(-0.76 to -0.23)	0.21<br>(-0.09 to 0.51)	-1.31<br>(-1.58 to -1.04)	-1.23<br>(-1.47 to -0.99)	-0.46<br>(-0.71 to -0.21)	-2.00<br>(-2.25 to -1.75)	-1.01<br>(-1.22 to -0.80)	-0.32<br>(-0.55 to -0.09)	-1.88<br>(-2.09 to -1.67)
Uruguay	0.71<br>(0.57 to 0.84)	1.11<br>(0.96 to 1.26)	0.38<br>(0.25 to 0.50)	0.03<br>(-0.07 to 0.14)	0.50<br>(0.38 to 0.62)	-0.33<br>(-0.42 to -0.23)	-0.01<br>(-0.11 to 0.09)	0.33<br>(0.21 to 0.44)	-0.31<br>(-0.41 to -0.22)
Uzbekistan	-0.48<br>(-0.97 to 0.00)	-0.27<br>(-0.77 to 0.24)	-0.72<br>(-1.18 to -0.25)	-0.73<br>(-1.15 to -0.30)	-0.47<br>(-0.90 to -0.03)	-0.99<br>(-1.41 to -0.58)	-0.91<br>(-1.35 to -0.48)	-0.74<br>(-1.21 to -0.27)	-1.11<br>(-1.51 to -0.71)
Vanuatu	0.01<br>(-0.07 to 0.09)	0.08<br>(0.01 to 0.15)	-0.03<br>(-0.13 to 0.06)	-0.10<br>(-0.16 to -0.03)	-0.03<br>(-0.09 to 0.03)	-0.14<br>(-0.22 to -0.06)	-0.11<br>(-0.21 to -0.01)	-0.04<br>(-0.12 to 0.04)	-0.16<br>(-0.29 to -0.04)
Venezuela (Bolivarian Republic of Venezuela)	1.41<br>(1.21 to 1.60)	1.75<br>(1.44 to 2.06)	1.00<br>(0.87 to 1.12)	0.49<br>(0.38 to 0.60)	0.74<br>(0.51 to 0.96)	0.28<br>(0.17 to 0.39)	0.58<br>(0.47 to 0.70)	0.79<br>(0.58 to 1.00)	0.40<br>(0.25 to 0.54)
Viet Nam	2.51<br>(2.40 to 2.62)	2.72<br>(2.60 to 2.84)	2.10<br>(1.98 to 2.21)	1.46<br>(1.33 to 1.59)	1.69<br>(1.55 to 1.83)	1.07<br>(0.93 to 1.20)	1.38<br>(1.27 to 1.49)	1.66<br>(1.54 to 1.78)	0.86<br>(0.76 to 0.96)
Yemen	0.58<br>(0.49 to 0.67)	0.49<br>(0.40 to 0.58)	0.63<br>(0.55 to 0.72)	0.16<br>(0.10 to 0.23)	0.19<br>(0.12 to 0.26)	0.11<br>(0.05 to 0.16)	0.05<br>(-0.03 to 0.12)	0.05<br>(-0.03 to 0.13)	0.02<br>(-0.05 to 0.10)
Zambia	1.18<br>(1.08 to 1.28)	1.92<br>(1.83 to 2.00)	0.38<br>(0.16 to 0.61)	0.99<br>(0.90 to 1.08)	1.73<br>(1.62 to 1.84)	0.21<br>(0.02 to 0.40)	0.88<br>(0.79 to 0.98)	1.65<br>(1.52 to 1.78)	0.02<br>(-0.18 to 0.22)
Zimbabwe	1.17<br>(0.70 to 1.64)	0.49<br>(0.09 to 0.89)	1.86<br>(1.22 to 2.51)	1.24<br>(0.79 to 1.70)	0.57<br>(0.21 to 0.93)	1.90<br>(1.27 to 2.52)	1.53<br>(1.00 to 2.07)	0.83<br>(0.38 to 1.27)	2.29<br>(1.56 to 3.03)

EAPC estimate annual percentage change, ASIR age-standardized incidence rate, ASMR age-standardized mortality rate, ASDR age-standardized disability-adjusted life-year rate, CI confidence interval

Table.S6 Global burden of disease study risk hierarchy with levels

Risk	Level
All risk factors	0
Behavioral risks	1
Alcohol use	2
Tobacco	2
Smoking	3
Dietary risks	2
Diet high in processed meat	3
Diet high in red meat	3
Diet low in calcium	3
Diet low in fiber	3
Diet low in milk	3
Diet low in whole grains	3
Low physical activity	2
Metabolic risks	1
High body-mass index	2
High fasting plasma glucose	2

Table.S7 Definition of all risk factors

Risk	Definition
All risk factors	
Behavioral risks	
Alcohol use	Theoretical minimum risk exposure level (TMREL) for high alcoh use is associated with the lowest overall mortality attributable to the risk, in a given location and year. Given varying exposure-response curves for different mean annual zones, as well as spatially and temporally varying cause compositions, the Global Burden of Disease Study (GBD) estimates TMRELs by year and location and does not use a globally uniform TMREL. High alcoh use exposure is defined as exposure to more alcohol intake than this TMREL
Tobacco	
Smoking	Including current smoking of any tobacco product and former smoking of any tobacco product.
Dietary risks	
Diet high in processed meat	Average daily consumption (in grams per day) of meat preserved by smoking, curing, salting, or addition of chemical preservatives more than 0 g/day.
Diet high in red meat	Average daily consumption (in grams per day) of unprocessed red meat more than 0–200 g/day, including pork and bovine meats such as beef, pork, lamb, and goat, but excluding all processed meats,poultry, fish, and eggs
Diet low in calcium	Average daily consumption (in grams per day) of calcium from all sources, including milk, yoghurt, and cheese less than 0.72–0.86 g/day (males),1.1–1.2 g/day (females).
Diet low in fiber	Average daily consumption (in grams per day) of fibre from all sources including fruits,vegetables, grains, legumes, and pulses less than 22–25 g/day.
Diet low in milk	Average daily consumption (in grams per day) of dairy milk including non-fat, low-fat, and full-fat milk, but excluding plant-based milks, fermented milk products such as buttermilk, and other dairy products such as Cheese, less than 280–340 g/day (males), 500–610 g/day (females)
Diet low in whole grains	Average daily consumption (in grams per day) of whole grains (bran, germ, and endosperm in their natural proportion) from breakfast cereals, bread, rice, pasta, biscuits, muffins, tortillas, pancakes, and other sources less than 160–210 g/day.
Low physical activity	Low physical activity is defined as objectively measured, total physical activity less than 3600 to 4400 MET-minutes per week. Use frequency, duration, and intensity of activity to calculate total metabolic equivalent (MET)-minutes per week. MET is the ratio of the working metabolic rate to the resting metabolic rate. One MET is equivalent to 1 kcal/kg/hour and is equal to the energy cost of sitting quietly. A MET is also defined as the oxygen uptake in ml/kg/min with one MET equal to the oxygen cost of sitting quietly, around 3.5 ml/kg/min.
Metabolic risks	
High body-mass index	High body-mass index (BMI) for adults (ages 20+) is defined as BMI greater than 20 to 23 kg/m^2. High BMI for children and adolescents (ages 2–19) is defined as being overweight or obese based on International Obesity Task Force standards.
High fasting plasma glucose	High fasting plasma glucose (FPG) is measured as the mean FPG in a population, where FPG is a continuous exposure in units of mmol/L. Since FPG is along a continuum, we define high FPG as any level above the theoretical minimum-risk exposure level (TMREL), which is 4.9–5.3 mmol/L.

Table.S8 Attributable deaths and ASMR by colorectal cancer risk factors in 2021

Risk factor	Deaths/10,000 (95% UI)			ASMR/100,000 persons (95% UI)		
	Both	Male	Female	Both	Male	Female
All risk factors	59.6<br>(39.02 - 76.05)	33.27<br>(22.33 - 42.05)	26.33<br>(16.74 - 34.05)	7.08<br>(4.63 - 9.04)	8.77<br>(5.85 - 11.12)	5.66<br>(3.6 - 7.32)
Behavioral risks						
Alcohol use	5.61<br>(4.37 - 6.99)	4.61<br>(3.57 - 5.74)	1<br>(0.74 - 1.29)	0.66<br>(0.51 - 0.82)	1.19<br>(0.91 - 1.48)	0.22<br>(0.16 - 0.28)
Tobacco						
Smoking	4.76<br>(2.97 - 6.6)	4<br>(2.5 - 5.55)	0.76<br>(0.47 - 1.08)	0.55<br>(0.34 - 0.77)	1.01<br>(0.63 - 1.4)	0.16<br>(0.1 - 0.23)
Dietary risks						
Diet high in processed meat	5.71<br>(-1.34 - 11.77)	3.02<br>(-0.72 - 6.2)	2.69<br>(-0.63 - 5.55)	0.68<br>(-0.16 - 1.4)	0.8<br>(-0.19 - 1.65)	0.58<br>(-0.14 - 1.19)
Diet high in red meat	15.3<br>(-0.01 - 31.42)	8.58<br>(0 - 17.57)	6.72<br>(0 - 13.6)	1.82<br>(0 - 3.74)	2.26<br>(0 - 4.64)	1.45<br>(0 - 2.93)
Diet low in calcium	8.91<br>(6.5 - 11.23)	3.7<br>(2.68 - 4.91)	5.21<br>(3.73 - 6.65)	1.06<br>(0.77 - 1.33)	0.96<br>(0.69 - 1.27)	1.13<br>(0.81 - 1.44)
Diet low in fiber	1.31<br>(0.58 - 2.03)	0.69<br>(0.3 - 1.07)	0.63<br>(0.28 - 0.97)	0.16<br>(0.07 - 0.24)	0.18<br>(0.08 - 0.28)	0.14<br>(0.06 - 0.21)
Diet low in milk	15.76<br>(4.3 - 26.25)	6.87<br>(1.89 - 11.58)	8.88<br>(2.44 - 14.69)	1.87<br>(0.51 - 3.12)	1.8<br>(0.49 - 3.04)	1.91<br>(0.53 - 3.17)
Diet low in whole grains	18.63<br>(7.61 - 28.48)	10.43<br>(4.21 - 15.93)	8.19<br>(3.42 - 12.34)	2.21<br>(0.91 - 3.38)	2.75<br>(1.11 - 4.2)	1.76<br>(0.74 - 2.66)
Low physical activity	7.15<br>(4.48 - 9.82)	2.98<br>(1.84 - 4.2)	4.16<br>(2.58 - 5.78)	0.87<br>(0.55 - 1.19)	0.84<br>(0.52 - 1.19)	0.89<br>(0.55 - 1.23)
Metabolic risks						
High body-mass index	9.93<br>(4.3 - 15.79)	5.1<br>(2.19 - 8.12)	4.83<br>(2.09 - 7.68)	0.98<br>(0.51 - 1.49)	1.25<br>(0.64 - 1.9)	0.77<br>(0.39 - 1.16)
High fasting plasma glucose	8.24<br>(4.24 - 12.54)	4.66<br>(2.38 - 7.11)	3.59<br>(1.83 - 5.44)	1.17<br>(0.51 - 1.87)	1.33<br>(0.57 - 2.13)	1.04<br>(0.45 - 1.65)

ASMR age-standardized mortality rate,UI uncertainty interval

Table.S9 Frontier analysis of ASMR across 204 countries and territories

Location name	year	val	SDI	frontier	potential	trend
15 worst-performing countries and territories in the frontier analysis of ASMR globally						
Uruguay	2021	27.46	0.72	2.67	24.79	Increase
Hungary	2021	26.01	0.79	2.67	23.34	Decrease
Bulgaria	2021	25.71	0.77	2.67	23.04	Increase
Monaco	2021	25.23	0.91	2.67	22.56	Increase
Slovakia	2021	24.71	0.81	2.67	22.04	Decrease
Greenland	2021	24.60	0.83	2.67	21.93	Decrease
Poland	2021	24.35	0.81	2.67	21.68	Increase
Croatia	2021	24.29	0.80	2.67	21.62	Decrease
Barbados	2021	22.93	0.75	2.67	20.26	Increase
Taiwan (Province of China)	2021	21.86	0.87	2.67	19.19	Increase
Serbia	2021	21.32	0.79	2.67	18.66	Decrease
Romania	2021	21.09	0.77	2.67	18.42	Increase
Brunei Darussalam	2021	20.16	0.81	2.67	17.49	Decrease
Czechia	2021	20.16	0.83	2.67	17.49	Decrease
Netherlands	2021	19.99	0.89	2.67	17.32	Decrease
5 worst-performing countries and territories in the frontier analysis of ASMR with high SDI level						
Monaco	2021	25.23	0.91	2.67	22.56	Increase
Taiwan (Province of China)	2021	21.86	0.87	2.67	19.19	Increase
Netherlands	2021	19.99	0.89	2.67	17.32	Decrease
Denmark	2021	18.97	0.90	2.67	16.30	Decrease
Norway	2021	18.12	0.92	2.67	15.45	Decrease
5 best-performing countries and territories in the frontier analysis of ASMR with low SDI level						
Somalia	2021	9.92	0.08	9.65	0.27	Increase
Gambia	2021	3.05	0.41	2.67	0.38	Increase
Niger	2021	5.05	0.17	4.25	0.80	Increase
Papua New Guinea	2021	3.56	0.42	2.67	0.89	Decrease
Bangladesh	2021	3.58	0.49	2.67	0.91	Decrease

ASMR age-standardized mortality rate, SDI socio-demographic index

Table.S10 Frontier analysis of ASDR across 204 countries and territories

Location name	year	val	SDI	frontier	potential	trend
15 worst-performing countries and territories in the frontier analysis of ASDR globally						
Hungary	2021	614.96	0.79	60.90	554.06	Decrease
Bulgaria	2021	605.00	0.77	60.38	544.62	Increase
Uruguay	2021	598.78	0.72	60.18	538.60	Increase
Greenland	2021	572.67	0.83	61.98	510.68	Decrease
Slovakia	2021	563.00	0.81	62.10	500.89	Decrease
Monaco	2021	551.88	0.91	60.52	491.36	Increase
Croatia	2021	524.86	0.80	60.50	464.37	Decrease
Poland	2021	522.54	0.81	60.17	462.36	Increase
Romania	2021	504.85	0.77	60.76	444.09	Increase
Taiwan (Province of China)	2021	503.50	0.87	60.67	442.83	Increase
Barbados	2021	496.33	0.75	60.24	436.09	Increase
Serbia	2021	484.60	0.79	60.17	424.42	Decrease
Seychelles	2021	469.22	0.73	60.20	409.02	Increase
Brunei Darussalam	2021	466.43	0.81	60.50	405.93	Decrease
Republic of Moldova	2021	450.95	0.73	60.95	390.00	Decrease
5 worst-performing countries and territories in the frontier analysis of ASDR with high SDI level						
Monaco	2021	551.88	0.91	60.52	491.36	Increase
Taiwan (Province of China)	2021	503.50	0.87	60.67	442.83	Increase
Netherlands	2021	444.07	0.89	60.17	383.90	Decrease
Denmark	2021	384.64	0.90	61.79	322.84	Decrease
Lithuania	2021	380.34	0.86	60.75	319.58	Decrease
5 best-performing countries and territories in the frontier analysis of ASDR with low SDI level						
Somalia	2021	243.25	0.08	243.10	0.15	Decrease
Gambia	2021	70.75	0.41	60.99	9.76	Increase
Niger	2021	110.28	0.17	97.72	12.56	Increase
Mozambique	2021	86.42	0.33	62.59	23.83	Increase
Bangladesh	2021	88.88	0.49	61.64	27.24	Decrease

ASDR age-standardized disability-adjusted life-year rate, SDI socio-demographic index

Table.S11 BAPC analysis of incidence rate (per 100,000) in males from 2022 to 2050

	15-19	20-24	25-29	30-34	35-39	40-44	45-49	50-54	55-59	60-64	65-69	70-74	75-79	80-84	85-89	90-94	95+
1990	0.43(0.01)	0.73(0.01)	1.39(0.01)	3.05(0.02)	5.82(0.03)	10.11(0.04)	17.7(0.06)	32.9(0.09)	54.65(0.12)	86.05(0.16)	128.65(0.23)	170.64(0.33)	238.39(0.48)	281.06(0.71)	343.78(1.26)	336.83(2.35)	281.71(4.16)
1991	0.42(0)	0.73(0.01)	1.41(0.01)	3.06(0.02)	5.9(0.03)	10.34(0.04)	17.82(0.06)	33.23(0.08)	54.99(0.12)	87.24(0.16)	129.86(0.23)	172.83(0.32)	237.98(0.47)	281.88(0.68)	343.23(1.15)	337.1(2)	284.77(3.33)
1992	0.41(0)	0.73(0.01)	1.43(0.01)	3.06(0.02)	5.94(0.03)	10.48(0.04)	17.97(0.06)	33.65(0.08)	55.64(0.12)	88.62(0.16)	132.14(0.22)	177.7(0.32)	237.44(0.46)	284.97(0.68)	344.79(1.13)	337.15(1.94)	286.94(3.1)
1993	0.41(0)	0.73(0.01)	1.45(0.01)	3.12(0.02)	5.98(0.03)	10.59(0.04)	18.27(0.06)	34.24(0.08)	56.82(0.12)	89.8(0.16)	134.51(0.22)	183.73(0.32)	235.25(0.46)	286.91(0.67)	345.93(1.11)	336.11(1.89)	287.69(3.04)
1994	0.4(0)	0.73(0.01)	1.45(0.01)	3.17(0.02)	5.98(0.03)	10.66(0.04)	18.61(0.06)	34.72(0.08)	57.66(0.12)	90.39(0.16)	136.65(0.22)	188.84(0.32)	232.55(0.45)	291.2(0.67)	349.27(1.1)	335.9(1.84)	289.17(3)
1995	0.39(0)	0.72(0.01)	1.45(0.01)	3.21(0.02)	5.93(0.02)	10.67(0.04)	19.05(0.05)	34.92(0.08)	58.26(0.12)	91.02(0.16)	138.88(0.22)	192.62(0.32)	234.62(0.45)	296.9(0.67)	357.27(1.1)	337.03(1.8)	290.25(2.96)
1996	0.38(0)	0.71(0.01)	1.46(0.01)	3.25(0.02)	5.86(0.02)	10.63(0.04)	19.3(0.05)	34.87(0.08)	58.67(0.12)	91.27(0.16)	140.31(0.22)	194.31(0.31)	238.02(0.45)	298.47(0.67)	364.45(1.1)	338.13(1.77)	289.66(2.89)
1997	0.38(0)	0.71(0.01)	1.46(0.01)	3.28(0.02)	5.83(0.02)	10.6(0.04)	19.25(0.05)	34.94(0.08)	58.64(0.12)	90.95(0.16)	140.15(0.22)	194.58(0.31)	242(0.44)	296.1(0.66)	368.21(1.09)	339.85(1.74)	288.63(2.82)
1998	0.37(0)	0.69(0.01)	1.45(0.01)	3.27(0.02)	5.82(0.02)	10.54(0.04)	19.1(0.05)	34.82(0.08)	58.84(0.12)	91.08(0.16)	139.94(0.22)	195.69(0.3)	248(0.44)	291.55(0.65)	373(1.08)	342.17(1.72)	285.83(2.73)
1999	0.37(0)	0.69(0.01)	1.46(0.01)	3.3(0.02)	5.89(0.02)	10.5(0.03)	19.02(0.05)	35.08(0.08)	59.32(0.12)	91.45(0.15)	139.14(0.21)	196(0.3)	253.41(0.43)	287.12(0.64)	376.99(1.07)	346.58(1.71)	284.04(2.66)
2000	0.37(0)	0.68(0.01)	1.48(0.01)	3.38(0.02)	6.03(0.02)	10.46(0.03)	19.1(0.05)	35.77(0.08)	59.45(0.12)	91.9(0.15)	139.11(0.21)	196.78(0.29)	254.87(0.43)	285.58(0.63)	379.33(1.07)	348.48(1.69)	281.24(2.58)
2001	0.36(0)	0.68(0.01)	1.47(0.01)	3.39(0.02)	6.13(0.02)	10.41(0.03)	19.17(0.05)	36.36(0.08)	59.4(0.12)	92.36(0.15)	138.72(0.21)	196.86(0.29)	254.07(0.42)	286.58(0.62)	381.11(1.06)	351.81(1.67)	279.67(2.52)
2002	0.36(0)	0.67(0.01)	1.47(0.01)	3.42(0.02)	6.2(0.02)	10.42(0.03)	19.22(0.05)	36.59(0.08)	59.53(0.11)	92.96(0.15)	138.99(0.21)	197.22(0.29)	254.31(0.41)	290.47(0.61)	376.85(1.05)	353.32(1.66)	280.19(2.47)
2003	0.36(0)	0.67(0.01)	1.48(0.01)	3.49(0.02)	6.26(0.02)	10.4(0.03)	19.37(0.05)	36.63(0.08)	59.62(0.11)	93.41(0.15)	140.59(0.21)	198.2(0.29)	255.97(0.4)	297.94(0.6)	374.5(1.03)	356.6(1.64)	282.68(2.43)
2004	0.35(0)	0.67(0.01)	1.49(0.01)	3.5(0.02)	6.34(0.02)	10.59(0.03)	19.43(0.05)	36.83(0.07)	60.5(0.11)	94.05(0.15)	141.46(0.21)	198.67(0.28)	257.82(0.4)	305.21(0.6)	370.27(1.02)	356.66(1.62)	284.58(2.4)
2005	0.35(0)	0.67(0.01)	1.47(0.01)	3.43(0.02)	6.4(0.02)	10.91(0.03)	19.45(0.05)	37.43(0.07)	62.83(0.11)	94.91(0.15)	142.97(0.21)	200.89(0.28)	260.71(0.39)	309.62(0.59)	373.08(1)	358.43(1.61)	287.39(2.38)
2006	0.35(0)	0.67(0.01)	1.44(0.01)	3.31(0.02)	6.42(0.02)	11.35(0.03)	19.28(0.05)	38.05(0.07)	64.3(0.11)	94.84(0.15)	142.18(0.2)	199.88(0.28)	260.62(0.39)	309.45(0.58)	372.32(0.98)	356.36(1.58)	289.17(2.35)
2007	0.34(0)	0.68(0.01)	1.45(0.01)	3.28(0.02)	6.49(0.02)	11.76(0.03)	19.36(0.05)	38.63(0.07)	64.89(0.11)	95.96(0.15)	142.41(0.2)	199.86(0.28)	260.54(0.38)	309.88(0.56)	379.4(0.96)	354.84(1.56)	289.65(2.32)
2008	0.34(0)	0.69(0.01)	1.48(0.01)	3.35(0.02)	6.55(0.02)	11.78(0.03)	19.97(0.05)	38.93(0.07)	65.04(0.11)	97.76(0.15)	143.83(0.2)	201.21(0.27)	262.21(0.38)	311.88(0.55)	388.54(0.95)	350.56(1.53)	291.38(2.29)
2009	0.33(0)	0.7(0.01)	1.53(0.01)	3.51(0.02)	6.52(0.02)	11.6(0.03)	20.74(0.05)	38.46(0.07)	64.06(0.1)	99.17(0.15)	144.54(0.2)	200.49(0.27)	261.75(0.37)	312.07(0.54)	393.76(0.93)	345.69(1.5)	291.54(2.25)
2010	0.33(0)	0.71(0.01)	1.57(0.01)	3.59(0.02)	6.47(0.02)	11.67(0.03)	21.39(0.05)	38.39(0.07)	64.22(0.1)	101.54(0.14)	145.75(0.2)	201.26(0.27)	262.28(0.37)	314.14(0.53)	396.52(0.91)	349.26(1.47)	292.93(2.22)
2011	0.33(0)	0.7(0.01)	1.6(0.01)	3.54(0.02)	6.31(0.02)	11.65(0.03)	21.95(0.05)	38.57(0.07)	65.04(0.1)	103.35(0.14)	145.74(0.2)	200.99(0.27)	262.25(0.36)	313.96(0.52)	398.33(0.89)	357.17(1.44)	291.29(2.17)
2012	0.32(0)	0.7(0.01)	1.64(0.01)	3.5(0.02)	6.16(0.02)	11.56(0.03)	22.58(0.05)	38.75(0.07)	65.72(0.1)	103.87(0.14)	146.28(0.2)	200.37(0.26)	262.82(0.36)	312.69(0.51)	399.69(0.87)	365.32(1.42)	289.59(2.13)
2013	0.32(0)	0.7(0.01)	1.69(0.01)	3.57(0.02)	6.2(0.02)	11.69(0.03)	22.73(0.05)	38.8(0.07)	65.78(0.1)	103.72(0.14)	146.12(0.19)	199.88(0.26)	262.88(0.36)	311.59(0.51)	399.67(0.85)	373.36(1.4)	288.42(2.11)
2014	0.32(0)	0.72(0.01)	1.73(0.01)	3.69(0.02)	6.42(0.02)	12.04(0.03)	22.32(0.05)	38.96(0.07)	65.5(0.1)	102.26(0.13)	145.6(0.19)	199.32(0.26)	261.68(0.35)	310.77(0.5)	400.63(0.83)	380.73(1.39)	290.6(2.09)
2015	0.32(0)	0.72(0.01)	1.76(0.01)	3.79(0.02)	6.54(0.02)	12.18(0.03)	22.22(0.05)	39.36(0.07)	65.6(0.1)	101.86(0.13)	147.81(0.18)	198.74(0.25)	260.89(0.35)	311.11(0.49)	407.94(0.82)	388.69(1.37)	297.02(2.07)
2016	0.32(0)	0.72(0.01)	1.79(0.01)	3.9(0.02)	6.63(0.02)	12.28(0.03)	22.36(0.05)	39.73(0.07)	65.52(0.1)	102(0.13)	149.78(0.18)	197.51(0.25)	260.09(0.34)	310.33(0.48)	412.45(0.81)	391.93(1.35)	303.69(2.06)
2017	0.32(0)	0.73(0.01)	1.81(0.01)	4.07(0.02)	6.88(0.02)	12.5(0.03)	22.29(0.05)	40.41(0.07)	65.11(0.1)	101.71(0.13)	149.42(0.18)	198.22(0.25)	258.55(0.34)	307.92(0.47)	411.04(0.8)	390.46(1.31)	311.34(2.06)
2018	0.32(0)	0.74(0.01)	1.82(0.01)	4.24(0.02)	7.1(0.02)	12.68(0.03)	22.25(0.05)	41.1(0.07)	65.44(0.09)	102.2(0.13)	149.82(0.17)	200.16(0.24)	258.69(0.33)	308.29(0.47)	409.72(0.78)	389.19(1.27)	317.29(2.04)
2019	0.32(0)	0.74(0.01)	1.8(0.01)	4.36(0.02)	7.21(0.02)	12.71(0.03)	22.46(0.05)	41.55(0.07)	66.1(0.09)	102.56(0.13)	149.46(0.17)	201.51(0.24)	258.77(0.33)	307.82(0.46)	412.07(0.77)	390.63(1.25)	320.95(2.01)
2020	0.32(0)	0.73(0.01)	1.77(0.01)	4.38(0.02)	7.19(0.02)	12.54(0.03)	22.2(0.05)	41.26(0.07)	65.72(0.09)	100.97(0.13)	148.06(0.17)	203.45(0.23)	258.57(0.33)	305.16(0.45)	410.95(0.76)	390.29(1.22)	322.21(1.99)
2021	0.32(0)	0.73(0.01)	1.75(0.01)	4.42(0.02)	7.31(0.02)	12.51(0.03)	22.06(0.05)	41.36(0.07)	66.28(0.09)	100.23(0.13)	148.06(0.17)	206.97(0.23)	258.98(0.33)	304.81(0.45)	411.55(0.76)	391.62(1.26)	325.56(2.16)
2022	0.32(0.01)	0.73(0.02)	1.73(0.04)	4.47(0.11)	7.38(0.13)	12.43(0.21)	21.92(0.35)	41.43(0.52)	66.49(0.9)	99.51(1.18)	147.39(1.64)	209.26(2.35)	259.06(3.07)	303.52(4.1)	411.52(5.41)	391.92(5.84)	328.8(6.12)
2023	0.32(0.01)	0.73(0.02)	1.71(0.06)	4.51(0.21)	7.46(0.24)	12.35(0.38)	21.76(0.63)	41.46(0.87)	66.76(1.58)	98.67(1.93)	146.79(2.62)	212.06(3.84)	259.26(5.08)	302.42(7.12)	411.58(9.06)	392.49(9.62)	331.62(9.45)
2024	0.32(0.01)	0.72(0.03)	1.69(0.08)	4.55(0.33)	7.54(0.37)	12.28(0.59)	21.6(0.97)	41.47(1.3)	67.03(2.42)	97.83(2.86)	146.19(3.83)	214.89(5.73)	259.46(7.59)	301.34(10.85)	411.68(13.62)	393.09(14.34)	334.44(13.63)
2025	0.32(0.02)	0.72(0.04)	1.67(0.11)	4.59(0.46)	7.62(0.53)	12.2(0.83)	21.45(1.36)	41.48(1.8)	67.3(3.4)	96.98(3.91)	145.61(5.23)	217.76(7.97)	259.65(10.5)	300.26(15.12)	411.82(18.87)	393.73(19.79)	337.3(18.51)
2026	0.32(0.02)	0.72(0.05)	1.65(0.14)	4.63(0.62)	7.7(0.71)	12.13(1.09)	21.29(1.79)	41.49(2.35)	67.58(4.5)	96.15(5.07)	145.04(6.78)	220.67(10.53)	259.85(13.75)	299.17(19.86)	411.93(24.73)	394.37(25.89)	340.19(24.01)
2027	0.32(0.02)	0.72(0.06)	1.63(0.17)	4.67(0.79)	7.78(0.91)	12.05(1.37)	21.14(2.25)	41.5(2.96)	67.86(5.71)	95.34(6.32)	144.46(8.48)	223.63(13.39)	260.05(17.32)	298.08(25.02)	411.99(31.17)	394.98(32.58)	343.1(30.1)
2028	0.32(0.03)	0.72(0.07)	1.61(0.2)	4.71(0.98)	7.86(1.13)	11.98(1.67)	20.99(2.74)	41.52(3.62)	68.14(7.03)	94.53(7.66)	143.86(10.29)	226.64(16.56)	260.25(21.18)	297.01(30.56)	412.03(38.1)	395.58(39.82)	346.02(36.75)
2029	0.32(0.03)	0.71(0.08)	1.59(0.24)	4.75(1.19)	7.95(1.37)	11.91(1.99)	20.84(3.26)	41.53(4.32)	68.43(8.44)	93.71(9.06)	143.27(12.22)	229.69(20.01)	260.46(25.3)	295.94(36.43)	412.12(45.52)	396.21(47.56)	348.93(43.93)

2030	0.32(0.04)	0.71(0.09)	1.57(0.28)	4.79(1.41)	8.03(1.62)	11.83(2.33)	20.69(3.81)	41.54(5.06)	68.71(9.95)	92.89(10.52)	142.68(14.25)	232.78(23.74)	260.66(29.68)	294.87(42.61)	412.24(53.37)	396.85(55.78)	351.87(51.64)
2031	0.32(0.04)	0.71(0.11)	1.55(0.31)	4.83(1.64)	8.11(1.89)	11.76(2.67)	20.54(4.37)	41.54(5.85)	68.99(11.56)	92.09(12.05)	142.11(16.39)	235.92(27.78)	260.87(34.31)	293.81(49.09)	412.35(61.66)	397.47(64.47)	354.85(59.87)
2032	0.32(0.05)	0.71(0.12)	1.53(0.35)	4.87(1.89)	8.2(2.19)	11.69(3.04)	20.39(4.96)	41.55(6.67)	69.28(13.25)	91.3(13.63)	141.53(18.62)	239.11(32.11)	261.07(39.18)	292.75(55.86)	412.43(70.38)	398.08(73.63)	357.85(68.65)
2033	0.32(0.06)	0.7(0.13)	1.51(0.39)	4.92(2.15)	8.28(2.5)	11.61(3.41)	20.24(5.57)	41.56(7.54)	69.56(15.04)	90.51(15.26)	140.95(20.94)	242.35(36.76)	261.29(44.29)	291.7(62.89)	412.5(79.5)	398.68(83.22)	360.86(77.94)
2034	0.32(0.06)	0.7(0.15)	1.49(0.43)	4.96(2.43)	8.37(2.82)	11.54(3.79)	20.1(6.19)	41.57(8.44)	69.85(16.91)	89.73(16.93)	140.37(23.34)	245.63(41.71)	261.5(49.63)	290.65(70.17)	412.59(89.02)	399.3(93.26)	363.86(87.76)
2035	0.32(0.07)	0.7(0.17)	1.47(0.47)	5(2.73)	8.46(3.17)	11.47(4.19)	19.95(6.83)	41.57(9.38)	70.14(18.87)	88.95(18.65)	139.78(25.83)	248.97(46.98)	261.71(55.19)	289.6(77.7)	412.67(98.91)	399.94(103.71)	366.87(98.09)
2036	0.32(0.07)	0.7(0.18)	1.45(0.52)	5.05(3.04)	8.55(3.54)	11.4(4.6)	19.81(7.48)	41.57(10.35)	70.44(20.91)	88.17(20.41)	139.2(28.4)	252.36(52.57)	261.93(60.97)	288.55(85.45)	412.76(109.18)	400.57(114.59)	369.89(108.95)
2037	0.32(0.08)	0.7(0.2)	1.43(0.56)	5.09(3.36)	8.64(3.92)	11.33(5.01)	19.66(8.15)	41.57(11.36)	70.73(23.05)	87.4(22.2)	138.62(31.05)	255.79(58.5)	262.15(66.98)	287.51(93.43)	412.85(119.83)	401.21(125.89)	372.94(120.35)
2038	0.32(0.09)	0.69(0.22)	1.41(0.6)	5.13(3.69)	8.73(4.32)	11.25(5.43)	19.52(8.83)	41.57(12.4)	71.02(25.26)	86.64(24.03)	138.05(33.78)	259.28(64.76)	262.37(73.2)	286.48(101.63)	412.94(130.84)	401.86(137.6)	376.02(132.29)
2039	0.32(0.1)	0.69(0.23)	1.4(0.64)	5.18(4.05)	8.82(4.74)	11.18(5.87)	19.38(9.52)	41.58(13.48)	71.32(27.56)	85.88(25.89)	137.48(36.58)	262.81(71.37)	262.58(79.63)	285.44(110.02)	413.02(142.2)	402.5(149.7)	379.12(144.76)
2040	0.32(0.1)	0.69(0.25)	1.38(0.69)	5.22(4.41)	8.91(5.18)	11.11(6.3)	19.24(10.23)	41.58(14.58)	71.62(29.95)	85.13(27.78)	136.9(39.44)	266.39(78.33)	262.8(86.26)	284.41(118.61)	413.11(153.9)	403.14(162.19)	382.24(157.77)
2041	0.32(0.11)	0.69(0.27)	1.36(0.73)	5.27(4.79)	9(5.64)	11.04(6.75)	19.1(10.94)	41.58(15.72)	71.92(32.41)	84.39(29.69)	136.34(42.37)	270.01(85.63)	263.02(93.09)	283.39(127.38)	413.2(165.92)	403.78(175.05)	385.39(171.31)
2042	0.32(0.12)	0.69(0.29)	1.34(0.77)	5.31(5.19)	9.1(6.12)	10.97(7.2)	18.96(11.66)	41.58(16.89)	72.22(34.96)	83.65(31.62)	135.77(45.35)	273.69(93.3)	263.24(100.11)	282.37(136.32)	413.29(178.27)	404.43(188.29)	388.57(185.38)
2043	0.32(0.13)	0.68(0.31)	1.33(0.81)	5.36(5.6)	9.19(6.62)	10.91(7.66)	18.82(12.39)	41.58(18.09)	72.52(37.58)	82.92(33.57)	135.21(48.4)	277.42(101.32)	263.46(107.32)	281.35(145.43)	413.37(190.93)	405.08(201.89)	391.77(199.99)
2044	0.32(0.14)	0.68(0.33)	1.31(0.86)	5.4(6.03)	9.29(7.14)	10.84(8.12)	18.69(13.13)	41.58(19.32)	72.82(40.29)	82.2(35.54)	134.65(51.49)	281.19(109.72)	263.68(114.71)	280.34(154.69)	413.46(203.9)	405.72(215.85)	395(215.15)
2045	0.32(0.15)	0.68(0.34)	1.29(0.9)	5.45(6.47)	9.38(7.67)	10.77(8.59)	18.55(13.88)	41.58(20.57)	73.12(43.07)	81.48(37.52)	134.09(54.64)	285.02(118.49)	263.89(122.28)	279.33(164.1)	413.55(217.16)	406.37(230.15)	398.25(230.84)
2046	0.32(0.15)	0.68(0.36)	1.28(0.94)	5.5(6.92)	9.48(8.23)	10.7(9.07)	18.42(14.63)	41.59(21.86)	73.43(45.94)	80.77(39.52)	133.53(57.83)	288.9(127.65)	264.11(130.03)	278.32(173.65)	413.63(230.71)	407.02(244.81)	401.54(247.08)
2047	0.32(0.16)	0.67(0.38)	1.26(0.99)	5.55(7.39)	9.58(8.81)	10.64(9.54)	18.28(15.38)	41.59(23.17)	73.73(48.88)	80.07(41.53)	132.98(61.07)	292.84(137.19)	264.33(137.95)	277.32(183.34)	413.72(244.55)	407.67(259.8)	404.85(263.88)
2048	0.32(0.17)	0.67(0.41)	1.24(1.03)	5.59(7.88)	9.68(9.41)	10.57(10.02)	18.15(16.14)	41.59(24.5)	74.04(51.9)	79.37(43.54)	132.43(64.35)	296.82(147.14)	264.55(146.04)	276.32(193.16)	413.81(258.67)	408.32(275.14)	408.18(281.22)
2049	0.32(0.18)	0.67(0.43)	1.23(1.07)	5.64(8.38)	9.78(10.03)	10.5(10.51)	18.02(16.91)	41.59(25.86)	74.35(55)	78.67(45.57)	131.88(67.68)	300.86(157.49)	264.77(154.3)	275.32(203.11)	413.9(273.07)	408.97(290.8)	411.55(299.13)
2050	0.32(0.19)	0.67(0.45)	1.21(1.11)	5.69(8.9)	9.88(10.67)	10.44(11)	17.89(17.68)	41.59(27.25)	74.66(58.17)	77.99(47.59)	131.33(71.04)	304.96(168.25)	264.99(162.73)	274.33(213.17)	413.98(287.74)	409.63(306.8)	414.94(317.6)

BAPC Bayesian age-period-cohort

Table.S12 BAPC analysis of incidence rate (per 100,000) in females from 2022 to 2050

	15-19	20-24	25-29	30-34	35-39	40-44	45-49	50-54	55-59	60-64	65-69	70-74	75-79	80-84	85-89	90-94	95+
1990	0.34(0)	0.65(0.01)	1.25(0.01)	2.66(0.02)	5.04(0.02)	9.03(0.04)	15.4(0.06)	27.78(0.08)	42.13(0.1)	63.16(0.14)	92.7(0.18)	125.52(0.25)	183.4(0.35)	225.98(0.5)	287.14(0.83)	316.06(1.53)	295.69(2.8)
1991	0.34(0)	0.65(0.01)	1.26(0.01)	2.65(0.02)	5.06(0.02)	9.16(0.04)	15.45(0.05)	27.98(0.08)	42.29(0.1)	63.26(0.13)	92.84(0.18)	126.54(0.25)	183.62(0.34)	226.64(0.48)	286.76(0.78)	314.93(1.35)	302.34(2.35)
1992	0.33(0)	0.65(0.01)	1.27(0.01)	2.65(0.02)	5.07(0.02)	9.19(0.03)	15.43(0.05)	28.1(0.08)	42.49(0.1)	63.4(0.13)	92.92(0.18)	128.81(0.24)	183.04(0.34)	228.59(0.48)	288.1(0.76)	316.65(1.32)	309.51(2.32)
1993	0.33(0)	0.65(0.01)	1.27(0.01)	2.67(0.02)	5.09(0.02)	9.25(0.03)	15.59(0.05)	28.42(0.08)	43.26(0.1)	63.77(0.13)	93.72(0.17)	132.66(0.24)	180.93(0.34)	230.1(0.47)	292.08(0.76)	320.14(1.3)	316(2.32)
1994	0.32(0)	0.64(0.01)	1.27(0.01)	2.7(0.02)	5.07(0.02)	9.26(0.03)	15.77(0.05)	28.52(0.08)	43.81(0.1)	63.43(0.13)	93.92(0.17)	135.66(0.24)	178.23(0.34)	232.92(0.47)	295.88(0.75)	322.09(1.27)	319.08(2.29)
1995	0.32(0)	0.64(0.01)	1.27(0.01)	2.73(0.02)	5.05(0.02)	9.27(0.03)	16.02(0.05)	28.53(0.08)	43.85(0.1)	62.86(0.13)	93.73(0.17)	136.68(0.24)	177.91(0.33)	236.55(0.47)	301.59(0.75)	324.66(1.25)	322.63(2.26)
1996	0.32(0)	0.64(0.01)	1.27(0.01)	2.75(0.02)	5.02(0.02)	9.25(0.03)	16.13(0.05)	28.32(0.07)	43.78(0.1)	62.34(0.13)	93.13(0.17)	135.86(0.23)	178.57(0.33)	237.63(0.47)	305.46(0.74)	327.64(1.23)	323.75(2.21)
1997	0.32(0)	0.63(0.01)	1.26(0.01)	2.75(0.02)	4.99(0.02)	9.17(0.03)	16.07(0.05)	28.19(0.07)	43.62(0.1)	61.83(0.13)	92.37(0.17)	134.68(0.23)	179.75(0.32)	235.11(0.47)	305.95(0.73)	328.07(1.21)	322.71(2.16)
1998	0.31(0)	0.63(0.01)	1.26(0.01)	2.73(0.02)	4.97(0.02)	9.14(0.03)	15.87(0.05)	28.09(0.07)	43.39(0.1)	61.54(0.12)	91.72(0.17)	134.14(0.23)	182.77(0.32)	231.52(0.47)	306.78(0.72)	328.87(1.19)	319.54(2.1)
1999	0.31(0)	0.63(0.01)	1.25(0.01)	2.74(0.02)	4.99(0.02)	9.12(0.03)	15.78(0.05)	28.12(0.07)	43.32(0.1)	61.88(0.12)	91.18(0.16)	133.5(0.22)	185.35(0.32)	225.17(0.46)	307.83(0.71)	330.13(1.17)	315.83(2.04)
2000	0.31(0)	0.62(0.01)	1.24(0.01)	2.72(0.02)	4.97(0.02)	8.95(0.03)	15.61(0.05)	28.29(0.07)	42.93(0.1)	61.4(0.12)	89.95(0.16)	131.67(0.22)	184.3(0.31)	221.31(0.45)	307(0.71)	331.09(1.15)	312.55(1.98)
2001	0.3(0)	0.61(0.01)	1.22(0.01)	2.68(0.02)	4.96(0.02)	8.82(0.03)	15.58(0.05)	28.54(0.07)	42.5(0.1)	61.18(0.12)	89.04(0.16)	130.3(0.22)	182.14(0.3)	219.89(0.44)	306.14(0.7)	331.67(1.13)	308.57(1.92)
2002	0.3(0)	0.6(0.01)	1.2(0.01)	2.65(0.02)	4.93(0.02)	8.72(0.03)	15.55(0.04)	28.38(0.07)	42.26(0.09)	61.17(0.12)	88.21(0.16)	129.39(0.21)	180.48(0.3)	221.01(0.43)	302.04(0.7)	331.43(1.12)	304.77(1.86)
2003	0.29(0)	0.59(0.01)	1.18(0.01)	2.66(0.02)	4.87(0.02)	8.63(0.03)	15.58(0.04)	28.25(0.07)	42.33(0.09)	61.54(0.12)	88.57(0.16)	128.49(0.21)	179.18(0.29)	224.56(0.42)	297.19(0.69)	331.06(1.1)	302.84(1.81)
2004	0.29(0)	0.59(0.01)	1.17(0.01)	2.63(0.02)	4.82(0.02)	8.6(0.03)	15.57(0.04)	27.93(0.06)	42.43(0.09)	61.25(0.12)	88.73(0.16)	127.5(0.21)	177.57(0.29)	227.96(0.41)	289.88(0.67)	329.94(1.08)	300.8(1.77)
2005	0.28(0)	0.59(0.01)	1.16(0.01)	2.6(0.02)	4.79(0.02)	8.71(0.03)	15.59(0.04)	28.07(0.06)	43.23(0.09)	61.24(0.12)	89.37(0.15)	127.74(0.21)	177.83(0.28)	229.06(0.41)	287.27(0.66)	329.55(1.07)	300.65(1.73)
2006	0.28(0)	0.58(0.01)	1.15(0.01)	2.54(0.02)	4.77(0.02)	8.82(0.03)	15.36(0.04)	28.14(0.06)	43.7(0.09)	60.68(0.12)	88.54(0.15)	126.27(0.2)	176.66(0.28)	226.84(0.4)	287(0.64)	328.29(1.06)	302.7(1.7)
2007	0.28(0)	0.58(0.01)	1.15(0.01)	2.52(0.02)	4.73(0.02)	8.91(0.03)	15.32(0.04)	28.24(0.06)	43.69(0.09)	60.92(0.12)	88.17(0.15)	125.43(0.2)	174.94(0.28)	224.95(0.39)	290.07(0.63)	324.8(1.04)	307.47(1.69)
2008	0.27(0)	0.59(0.01)	1.19(0.01)	2.57(0.02)	4.71(0.02)	8.88(0.03)	15.49(0.04)	28.09(0.06)	43.25(0.08)	61.17(0.11)	87.96(0.15)	124.98(0.2)	174.33(0.27)	224.29(0.38)	295.84(0.62)	320.05(1.03)	311.68(1.67)
2009	0.27(0)	0.6(0.01)	1.24(0.01)	2.62(0.02)	4.69(0.02)	8.72(0.03)	15.7(0.04)	27.76(0.06)	42.15(0.08)	61.08(0.11)	87.51(0.15)	123.62(0.2)	171.32(0.27)	220.56(0.37)	297.27(0.6)	310.21(0.99)	312.59(1.64)
2010	0.27(0)	0.6(0.01)	1.25(0.01)	2.64(0.02)	4.66(0.02)	8.6(0.03)	15.71(0.04)	27.65(0.06)	41.6(0.08)	61.82(0.11)	87.02(0.15)	122.71(0.19)	169.31(0.26)	218.56(0.37)	294.39(0.59)	306.86(0.97)	314.42(1.61)
2011	0.27(0)	0.6(0.01)	1.25(0.01)	2.66(0.02)	4.64(0.02)	8.58(0.03)	15.59(0.04)	27.55(0.06)	41.56(0.08)	62.47(0.11)	86.37(0.15)	121.76(0.19)	167.3(0.26)	218.68(0.36)	293.23(0.57)	309.99(0.94)	315.82(1.6)
2012	0.26(0)	0.6(0.01)	1.25(0.01)	2.65(0.02)	4.64(0.02)	8.59(0.03)	15.41(0.04)	27.43(0.06)	41.69(0.08)	61.71(0.1)	85.52(0.14)	120.77(0.19)	164.94(0.25)	215.87(0.36)	288.36(0.56)	311.9(0.91)	313.99(1.57)
2013	0.26(0)	0.59(0.01)	1.26(0.01)	2.66(0.02)	4.66(0.02)	8.66(0.03)	15.38(0.04)	27.24(0.06)	41.48(0.08)	61.05(0.1)	85.03(0.14)	119.97(0.19)	163.74(0.25)	212.38(0.35)	284.51(0.55)	315.13(0.89)	310.43(1.54)
2014	0.26(0)	0.59(0.01)	1.25(0.01)	2.65(0.02)	4.67(0.02)	8.77(0.03)	15.29(0.04)	27.24(0.06)	41.72(0.08)	60.44(0.1)	84.59(0.14)	119.15(0.19)	162.95(0.25)	209.09(0.34)	282.35(0.53)	318.44(0.88)	309.08(1.51)
2015	0.26(0)	0.59(0.01)	1.25(0.01)	2.65(0.02)	4.71(0.02)	8.83(0.03)	15.2(0.04)	27.47(0.06)	42(0.08)	60.23(0.1)	85.64(0.13)	118.78(0.18)	162.06(0.24)	206.89(0.34)	282.97(0.53)	320.25(0.86)	313.67(1.49)
2016	0.26(0)	0.59(0.01)	1.25(0.01)	2.69(0.02)	4.76(0.02)	8.86(0.03)	15.27(0.04)	27.44(0.06)	42.11(0.08)	60.41(0.1)	86.68(0.13)	118.42(0.18)	162.06(0.24)	205.13(0.33)	284.25(0.52)	317.83(0.84)	318.39(1.46)
2017	0.26(0)	0.58(0.01)	1.25(0.01)	2.73(0.02)	4.79(0.02)	8.9(0.03)	15.4(0.04)	27.46(0.05)	41.89(0.08)	60.63(0.1)	86.99(0.13)	118.56(0.18)	162.05(0.24)	201.6(0.32)	280.83(0.51)	314.5(0.82)	328.04(1.45)
2018	0.26(0)	0.58(0.01)	1.25(0.01)	2.76(0.02)	4.81(0.02)	8.93(0.03)	15.56(0.04)	27.56(0.05)	42.05(0.07)	61.17(0.1)	87.39(0.13)	119(0.17)	162.19(0.24)	200.49(0.32)	277.55(0.5)	312.9(0.8)	334.66(1.43)
2019	0.25(0)	0.57(0.01)	1.24(0.01)	2.78(0.02)	4.84(0.02)	8.87(0.03)	15.57(0.04)	27.46(0.05)	42(0.07)	61.3(0.1)	87.18(0.12)	120.14(0.17)	162.28(0.24)	200.51(0.31)	275.03(0.49)	310.66(0.79)	333.86(1.4)
2020	0.25(0)	0.57(0.01)	1.22(0.01)	2.77(0.02)	4.8(0.02)	8.73(0.03)	15.37(0.04)	27.11(0.05)	41.36(0.07)	60.09(0.09)	85.98(0.12)	120.54(0.17)	160.86(0.23)	198.65(0.31)	267.95(0.48)	304.51(0.77)	327.86(1.35)
2021	0.25(0)	0.57(0.01)	1.2(0.01)	2.78(0.02)	4.79(0.02)	8.72(0.03)	15.3(0.04)	27.23(0.05)	41.61(0.07)	60.09(0.09)	87.02(0.12)	123.33(0.17)	161.59(0.23)	198.16(0.31)	264.03(0.48)	303.54(0.78)	321.54(1.39)
2022	0.25(0.01)	0.57(0.01)	1.19(0.03)	2.79(0.05)	4.79(0.06)	8.65(0.13)	15.21(0.21)	27.11(0.3)	41.44(0.55)	59.72(0.71)	86.75(0.99)	124.64(1.48)	161.27(1.93)	197.22(2.75)	259.14(3.65)	300.15(4.13)	316.61(5.84)
2023	0.25(0.01)	0.57(0.02)	1.17(0.04)	2.79(0.08)	4.78(0.09)	8.59(0.21)	15.11(0.34)	27.02(0.47)	41.35(0.93)	59.34(1.13)	86.76(1.58)	126.43(2.47)	161.18(3.17)	196.37(4.83)	254.43(6.26)	297.29(6.77)	310.87(9.85)
2024	0.25(0.01)	0.56(0.02)	1.16(0.06)	2.8(0.11)	4.77(0.13)	8.53(0.31)	15.01(0.5)	26.93(0.67)	41.26(1.41)	58.96(1.65)	86.76(2.32)	128.25(3.73)	161.07(4.73)	195.54(7.4)	249.82(9.42)	294.43(10.02)	305.17(14.65)
2025	0.25(0.01)	0.56(0.03)	1.14(0.08)	2.8(0.15)	4.76(0.17)	8.47(0.43)	14.9(0.69)	26.85(0.91)	41.17(1.96)	58.58(2.25)	86.77(3.18)	130.1(5.22)	160.95(6.52)	194.72(10.33)	245.27(12.95)	291.58(13.7)	299.56(20)
2026	0.25(0.01)	0.56(0.04)	1.13(0.1)	2.81(0.2)	4.75(0.22)	8.41(0.56)	14.8(0.89)	26.77(1.17)	41.09(2.56)	58.21(2.92)	86.79(4.14)	131.99(6.92)	160.85(8.53)	193.9(13.59)	240.83(16.77)	288.78(17.74)	294.07(25.77)
2027	0.25(0.02)	0.56(0.05)	1.11(0.12)	2.81(0.24)	4.73(0.27)	8.35(0.7)	14.7(1.12)	26.68(1.46)	41(3.23)	57.85(3.64)	86.81(5.19)	133.91(8.82)	160.75(10.72)	193.08(17.12)	236.44(20.82)	286.02(22.1)	288.7(31.89)
2028	0.25(0.02)	0.56(0.05)	1.1(0.14)	2.82(0.3)	4.72(0.32)	8.29(0.85)	14.6(1.35)	26.6(1.76)	40.92(3.94)	57.48(4.41)	86.82(6.33)	135.85(10.92)	160.65(13.09)	192.26(20.91)	232.12(25.06)	283.29(26.73)	283.42(38.27)
2029	0.25(0.02)	0.56(0.06)	1.08(0.17)	2.82(0.35)	4.71(0.38)	8.23(1)	14.5(1.6)	26.52(2.09)	40.83(4.7)	57.11(5.22)	86.82(7.54)	137.82(13.22)	160.55(15.61)	191.46(24.93)	227.89(29.44)	280.59(31.59)	278.18(44.86)

2030	0.25(0.03)	0.55(0.07)	1.07(0.19)	2.83(0.41)	4.7(0.44)	8.17(1.17)	14.4(1.86)	26.43(2.44)	40.74(5.51)	56.73(6.07)	86.82(8.83)	139.82(15.71)	160.43(18.29)	190.65(29.15)	223.74(33.93)	277.88(36.66)	273.03(51.6)
2031	0.25(0.03)	0.55(0.08)	1.05(0.22)	2.83(0.48)	4.69(0.51)	8.12(1.34)	14.3(2.14)	26.35(2.8)	40.66(6.35)	56.37(6.96)	86.84(10.19)	141.87(18.41)	160.33(21.1)	189.85(33.57)	219.67(38.52)	275.21(41.91)	267.98(58.48)
2032	0.25(0.04)	0.55(0.09)	1.04(0.25)	2.84(0.54)	4.67(0.57)	8.06(1.52)	14.2(2.42)	26.27(3.18)	40.57(7.23)	56(7.89)	86.86(11.62)	143.94(21.3)	160.23(24.07)	189.05(38.19)	215.67(43.18)	272.56(47.34)	263.04(65.47)
2033	0.24(0.04)	0.55(0.11)	1.02(0.27)	2.84(0.61)	4.66(0.64)	8(1.7)	14.1(2.71)	26.18(3.58)	40.49(8.15)	55.64(8.85)	86.87(13.13)	146.05(24.41)	160.13(27.16)	188.25(42.97)	211.74(47.9)	269.94(52.94)	258.18(72.54)
2034	0.24(0.04)	0.55(0.12)	1.01(0.3)	2.85(0.69)	4.65(0.72)	7.94(1.89)	14(3.02)	26.1(4)	40.4(9.11)	55.28(9.84)	86.87(14.69)	148.19(27.72)	160.03(30.39)	187.47(47.93)	207.87(52.66)	267.35(58.67)	253.38(79.64)
2035	0.24(0.05)	0.55(0.13)	1(0.33)	2.85(0.76)	4.64(0.8)	7.89(2.08)	13.9(3.33)	26.01(4.43)	40.32(10.09)	54.92(10.86)	86.88(16.33)	150.36(31.25)	159.92(33.74)	186.68(53.04)	204.07(57.45)	264.77(64.54)	248.65(86.77)
2036	0.24(0.05)	0.54(0.14)	0.98(0.36)	2.86(0.84)	4.62(0.88)	7.83(2.28)	13.8(3.64)	25.93(4.87)	40.23(11.12)	54.56(11.91)	86.88(18.03)	152.58(35)	159.81(37.22)	185.9(58.3)	200.34(62.25)	262.23(70.52)	244(93.91)
2037	0.24(0.06)	0.54(0.16)	0.97(0.39)	2.86(0.92)	4.61(0.96)	7.78(2.48)	13.71(3.97)	25.85(5.33)	40.15(12.17)	54.2(12.98)	86.89(19.79)	154.83(38.98)	159.71(40.82)	185.13(63.71)	196.68(67.05)	259.72(76.63)	239.44(101.05)
2038	0.24(0.06)	0.54(0.17)	0.96(0.42)	2.87(1.01)	4.6(1.04)	7.72(2.69)	13.61(4.3)	25.76(5.81)	40.06(13.26)	53.84(14.09)	86.9(21.62)	157.11(43.19)	159.6(44.55)	184.35(69.25)	193.09(71.85)	257.23(82.83)	234.96(108.16)
2039	0.24(0.07)	0.54(0.18)	0.94(0.45)	2.87(1.1)	4.58(1.13)	7.66(2.9)	13.51(4.64)	25.68(6.3)	39.98(14.38)	53.48(15.21)	86.91(23.5)	159.42(47.63)	159.5(48.38)	183.58(74.92)	189.56(76.64)	254.76(89.13)	230.57(115.24)
2040	0.24(0.07)	0.54(0.2)	0.93(0.48)	2.88(1.19)	4.57(1.22)	7.61(3.11)	13.42(4.99)	25.6(6.8)	39.9(15.52)	53.13(16.36)	86.91(25.45)	161.77(52.31)	159.39(52.33)	182.81(80.72)	186.1(81.39)	252.32(95.5)	226.26(122.27)
2041	0.24(0.08)	0.53(0.21)	0.92(0.5)	2.88(1.28)	4.56(1.31)	7.56(3.33)	13.32(5.34)	25.51(7.31)	39.81(16.7)	52.78(17.52)	86.92(27.45)	164.16(57.24)	159.29(56.39)	182.05(86.63)	182.7(86.12)	249.9(101.95)	222.02(129.24)
2042	0.24(0.08)	0.53(0.23)	0.91(0.53)	2.88(1.38)	4.55(1.41)	7.5(3.55)	13.23(5.69)	25.43(7.84)	39.73(17.9)	52.43(18.71)	86.93(29.51)	166.58(62.41)	159.18(60.55)	181.29(92.65)	179.36(90.8)	247.5(108.45)	217.87(136.14)
2043	0.24(0.09)	0.53(0.24)	0.89(0.56)	2.89(1.47)	4.53(1.5)	7.45(3.78)	13.13(6.05)	25.35(8.37)	39.65(19.12)	52.09(19.91)	86.94(31.62)	169.03(67.83)	159.08(64.81)	180.53(98.78)	176.08(95.43)	245.12(115)	213.8(142.95)
2044	0.24(0.1)	0.53(0.26)	0.88(0.59)	2.89(1.58)	4.52(1.6)	7.39(4)	13.04(6.41)	25.27(8.92)	39.56(20.37)	51.74(21.12)	86.95(33.78)	171.52(73.51)	158.98(69.17)	179.78(105)	172.86(100)	242.77(121.59)	209.8(149.67)
2045	0.24(0.1)	0.53(0.27)	0.87(0.62)	2.9(1.68)	4.51(1.7)	7.34(4.23)	12.95(6.78)	25.19(9.47)	39.48(21.65)	51.4(22.35)	86.95(36)	174.05(79.45)	158.87(73.62)	179.03(111.32)	169.7(104.51)	240.44(128.22)	205.88(156.3)
2046	0.24(0.11)	0.53(0.29)	0.86(0.65)	2.9(1.78)	4.5(1.8)	7.29(4.46)	12.86(7.15)	25.1(10.04)	39.4(22.94)	51.06(23.6)	86.96(38.26)	176.61(85.66)	158.77(78.17)	178.28(117.72)	166.6(108.96)	238.14(134.88)	202.03(162.81)
2047	0.24(0.11)	0.52(0.3)	0.85(0.68)	2.91(1.89)	4.48(1.91)	7.24(4.69)	12.77(7.52)	25.02(10.62)	39.32(24.26)	50.73(24.85)	86.97(40.57)	179.22(92.14)	158.66(82.81)	177.53(124.21)	163.56(113.34)	235.85(141.56)	198.25(169.22)
2048	0.23(0.12)	0.52(0.32)	0.83(0.71)	2.91(2)	4.47(2.01)	7.19(4.92)	12.68(7.9)	24.94(11.2)	39.23(25.6)	50.39(26.12)	86.98(42.93)	181.86(98.9)	158.56(87.53)	176.79(130.78)	160.57(117.64)	233.59(148.26)	194.54(175.51)
2049	0.23(0.13)	0.52(0.33)	0.82(0.74)	2.92(2.12)	4.46(2.12)	7.13(5.16)	12.59(8.28)	24.86(11.79)	39.15(26.96)	50.06(27.4)	86.99(45.34)	184.54(105.95)	158.46(92.34)	176.05(137.42)	157.64(121.87)	231.35(154.97)	190.91(181.68)
2050	0.23(0.13)	0.52(0.35)	0.81(0.76)	2.92(2.23)	4.45(2.23)	7.08(5.39)	12.5(8.66)	24.78(12.39)	39.07(28.34)	49.73(28.68)	86.99(47.79)	187.26(113.29)	158.35(97.24)	175.32(144.14)	154.75(126.02)	229.13(161.68)	187.34(187.72)

BAPC Bayesian age-period-cohort
