# Supplementary material for: Global, regional, national burden of colorectal cancer from 1990 to 2021, with projections of incidence to 2050: a systematic analysis of the global burden of disease study 2021
Source: Front Oncol. 2025 Oct 6;15:1597847. doi: 10.3389/fonc.2025.1597847 (PMC12535885; doi:10.3389/fonc.2025.1597847)
Supplement: Supplementary file 2 [file Supplementaryfile2.pdf]

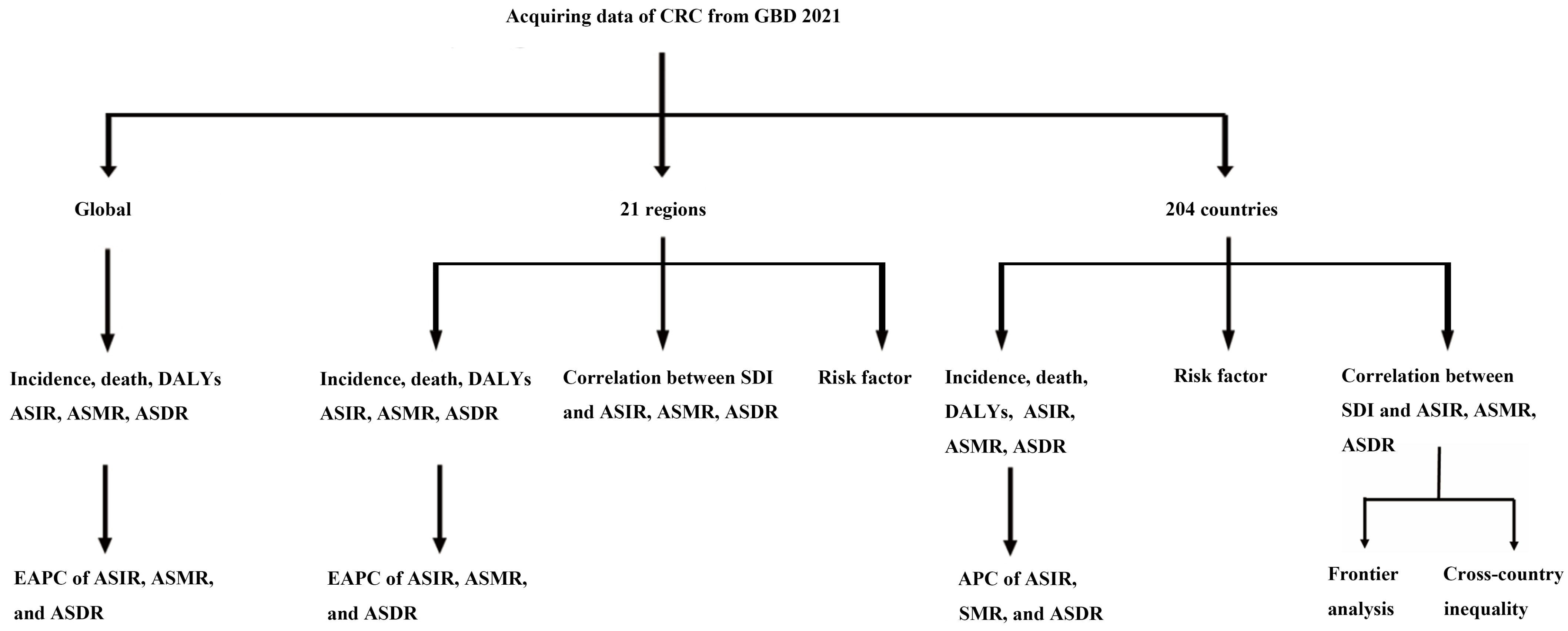

Fig. S1 Flowcharting of the analysis process. GBD Global Burden of Disease Study, CRC colorectal cancer, DALYs disability-adjusted life-years, ASIR age-standardized incidence rate, ASMR age-standardized mortality rate, ASDR age-standardized disability-adjusted life-year rate, EAPC estimated annual percentage change, SDI socio-demographic index.

A

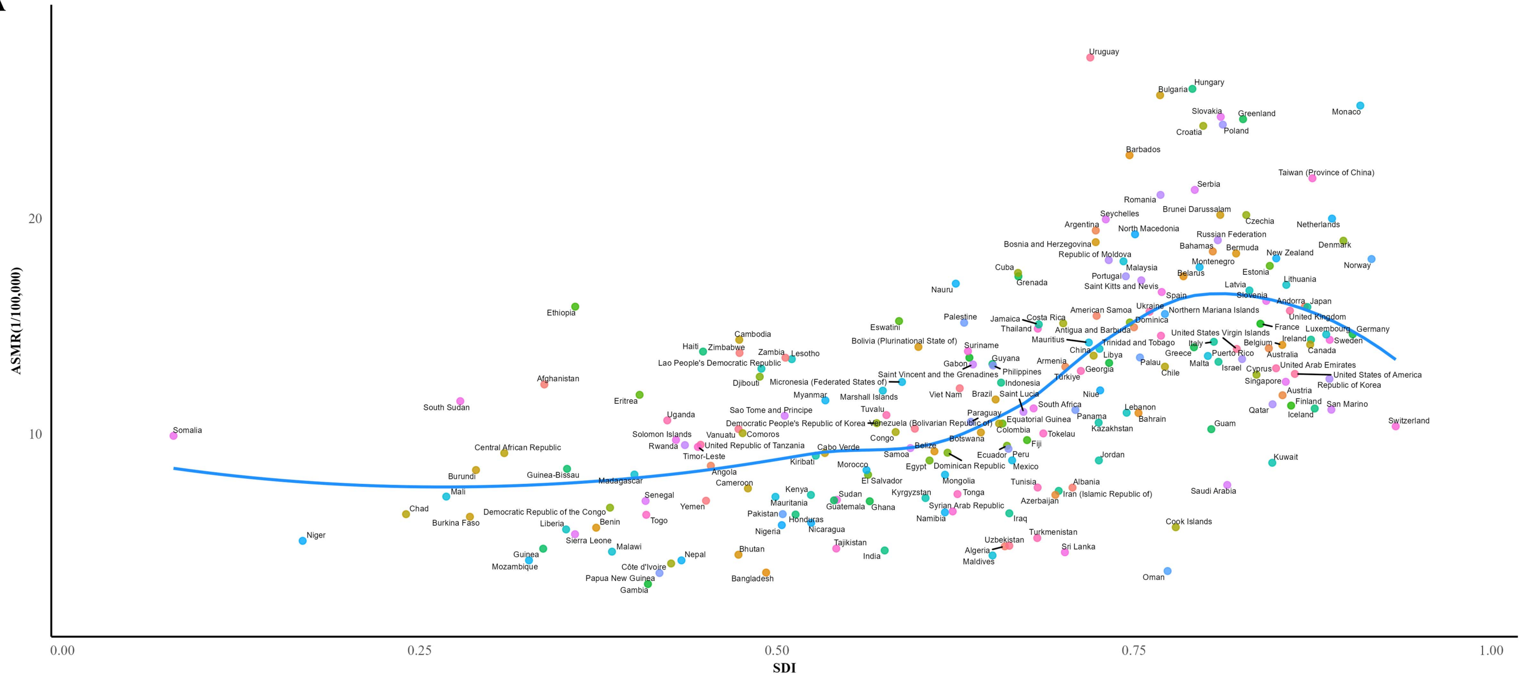

B

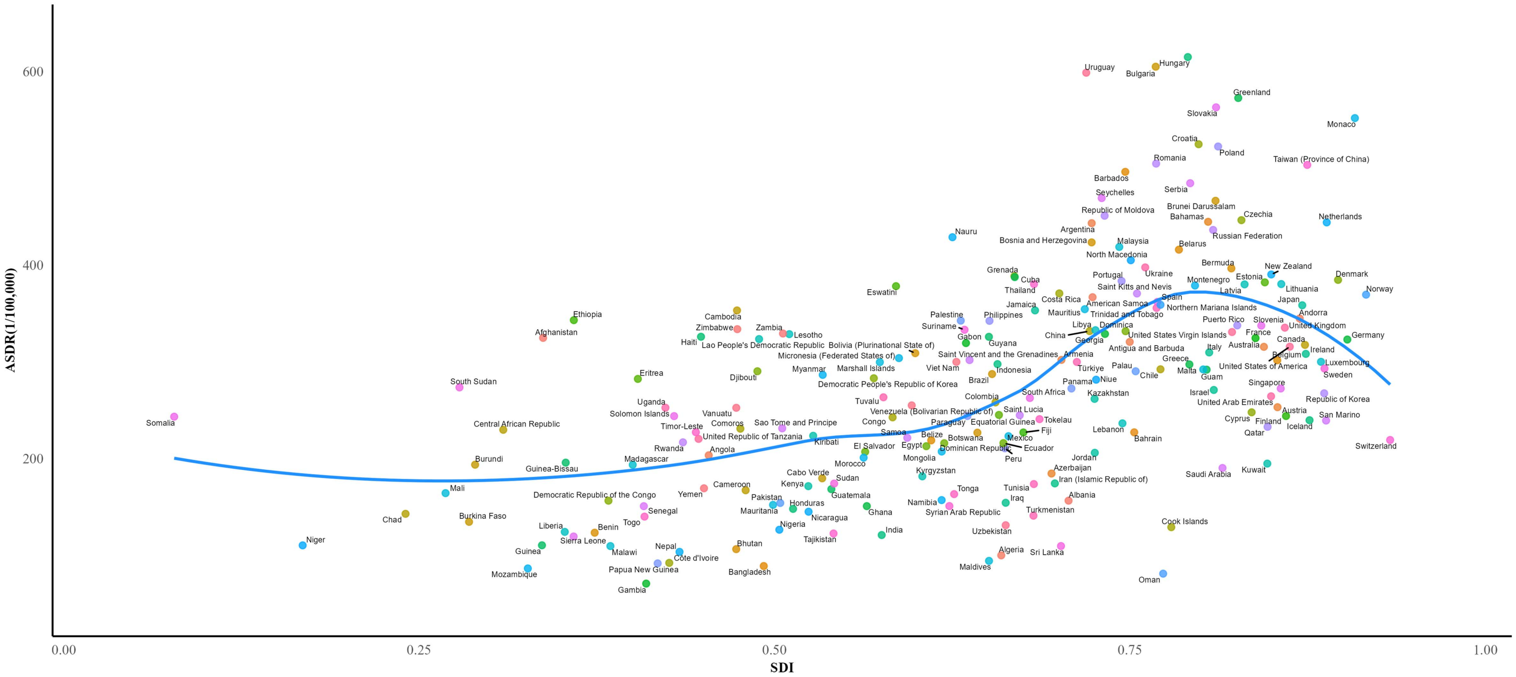

Fig.S2 A: ASMRs for CRC of 204 countries and territories in 2021. B: ASDRs for CRC of 204 countries and territories in 2021. ASMR age-standardized mortality rate, ASDR age-standardized disability-adjusted life-year rate, CRC colorectal cancer, SDI socio-demographic index.

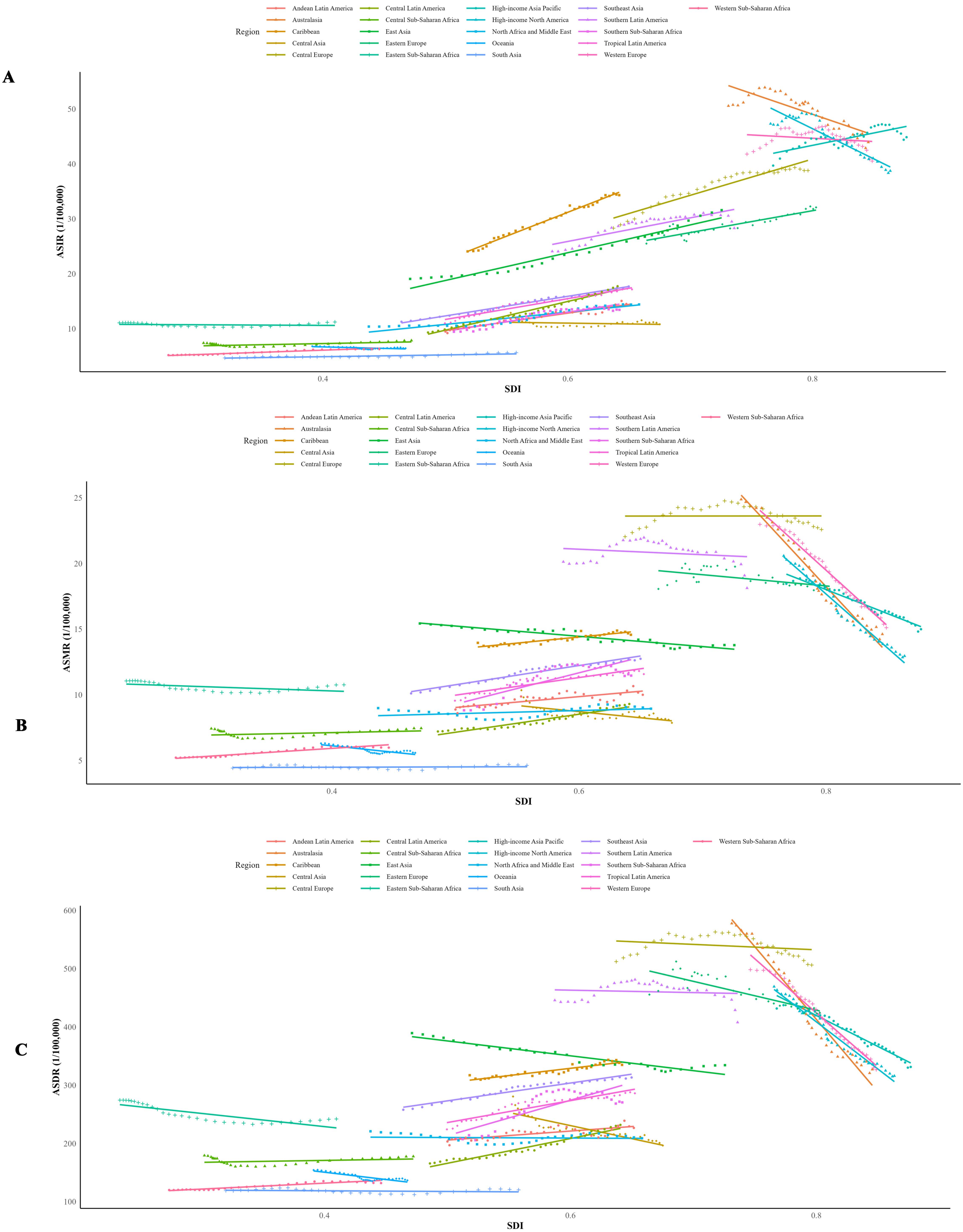

Fig.S3 ASIRs (A), ASMRs (B) and ASDRs (C) for CRC of 21 regions in 2021. ASIR age-standardized incidence rate, ASMR age-standardized mortality rate, ASDR age-standardized disability-adjusted life-year rate, CRC colorectal cancer, SDI socio-demographic index.

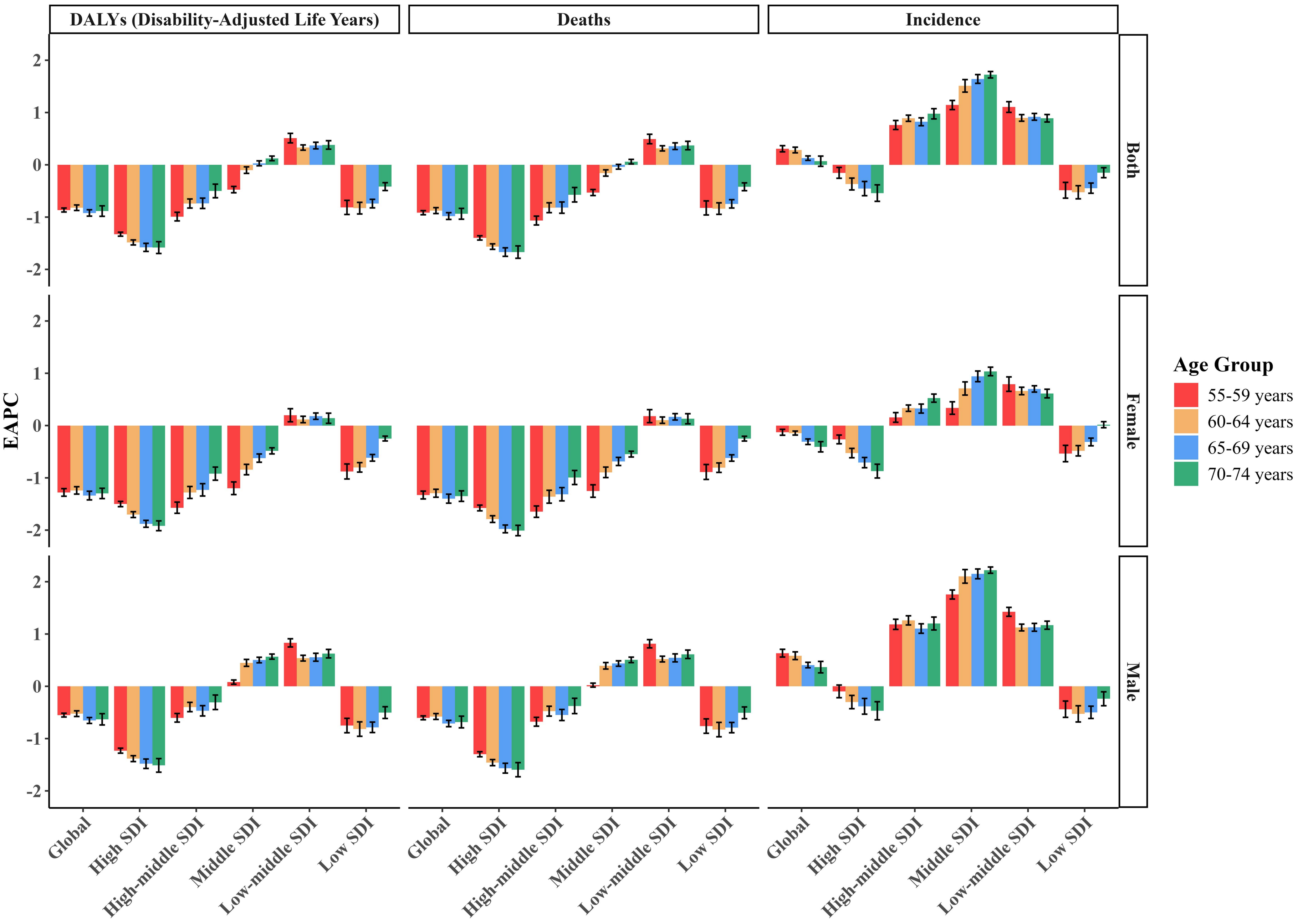

Fig.S4 EAPC of ASIR, ASMR, and ASDR for CRC in countries with five SDI levels from 1990 to 2021. ASIR age-standardized incidence rate, ASMR age-standardized mortality rate, ASDR age-standardized disability-adjusted life-year rate, CRC colorectal cancer, SDI socio-demographic index.

A

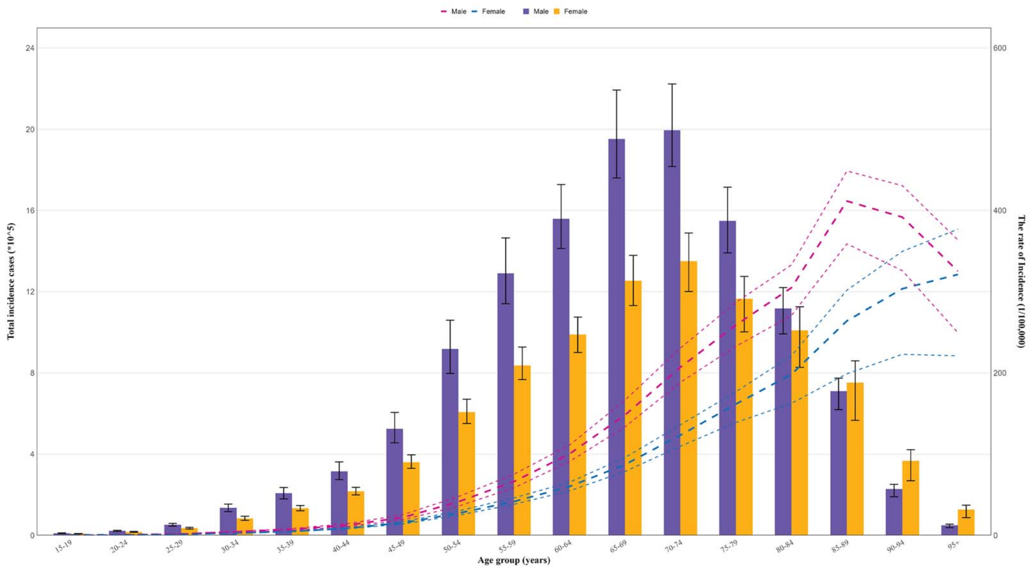

B

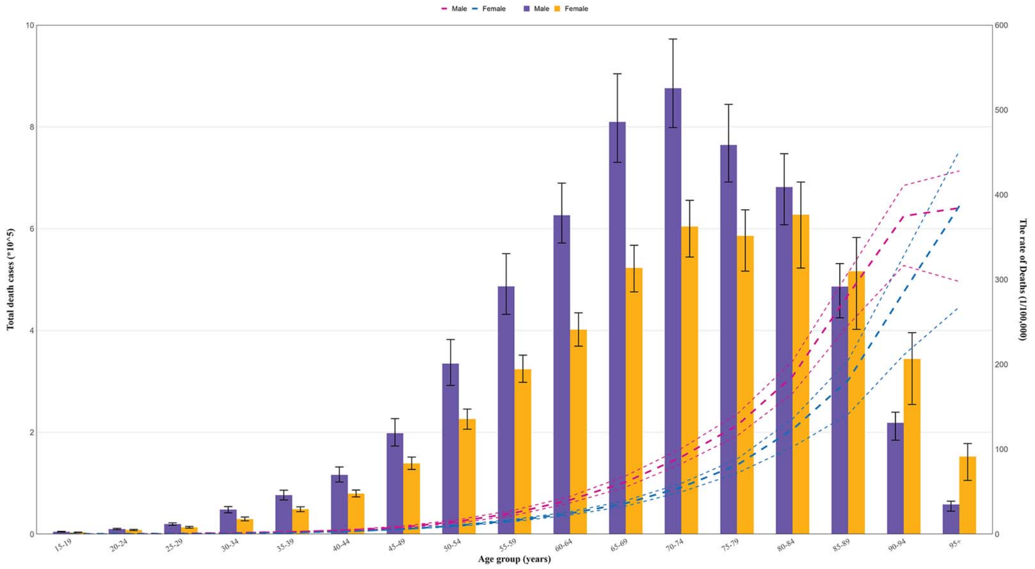

C

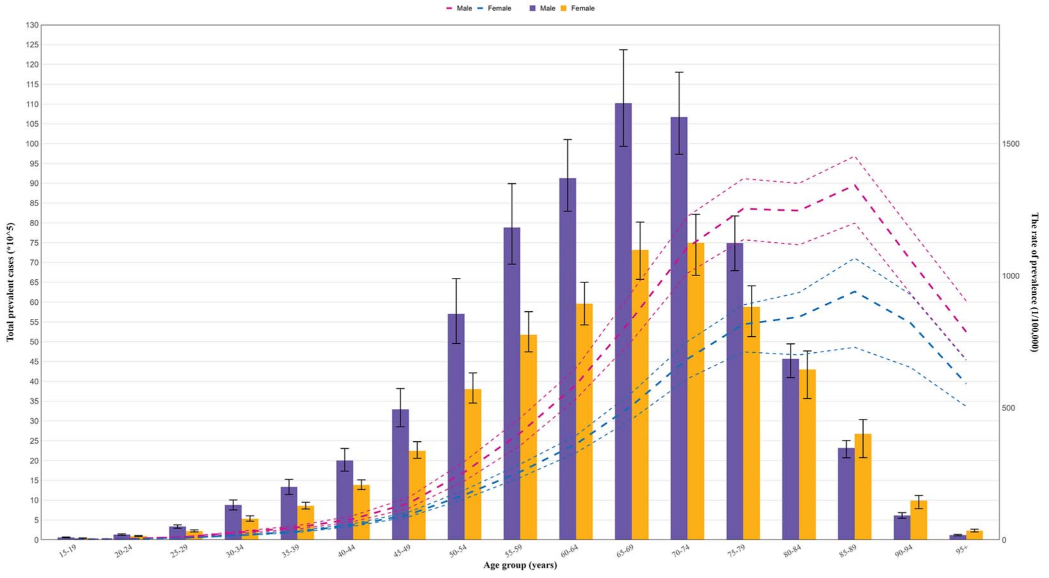

Fig.S5 Global incidence (A), mortality (B), prevalence (C) rates and numbers of colorectal cancer across different age groups in 2021.

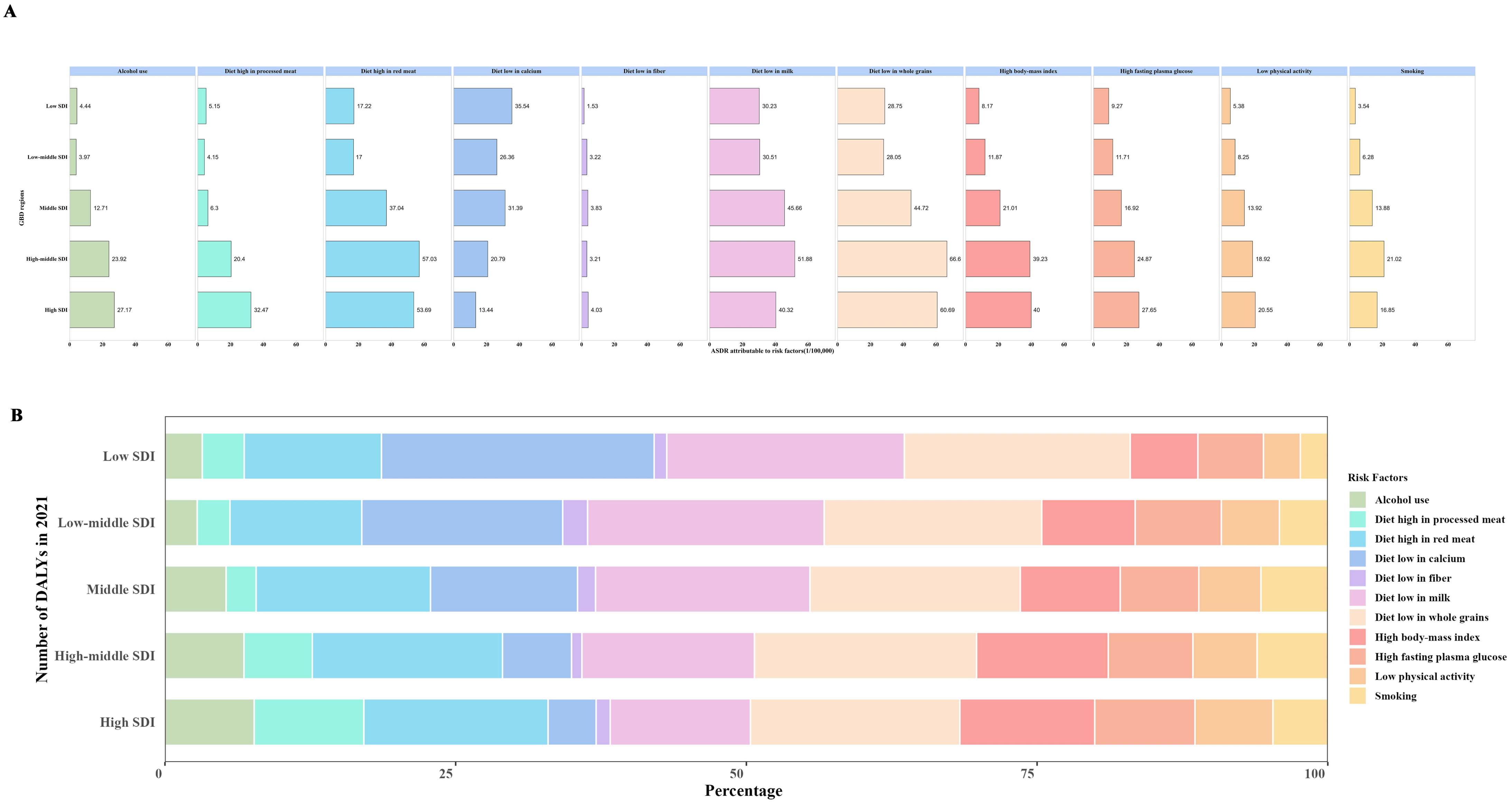

Fig.S6 Attributable DALYs by CRC risk factors in five SDI levels regions in 2021. A: Numbers of ASDRs attributable to CRC risk factors in five SDI levels regions in 2021. B: Percentage of DALYs attributable to CRC risk factors in five SDI levels regions in 2021. ASDR age-standardized disability-adjusted life-year rate, CRC colorectal cancer, SDI socio-demographic index.

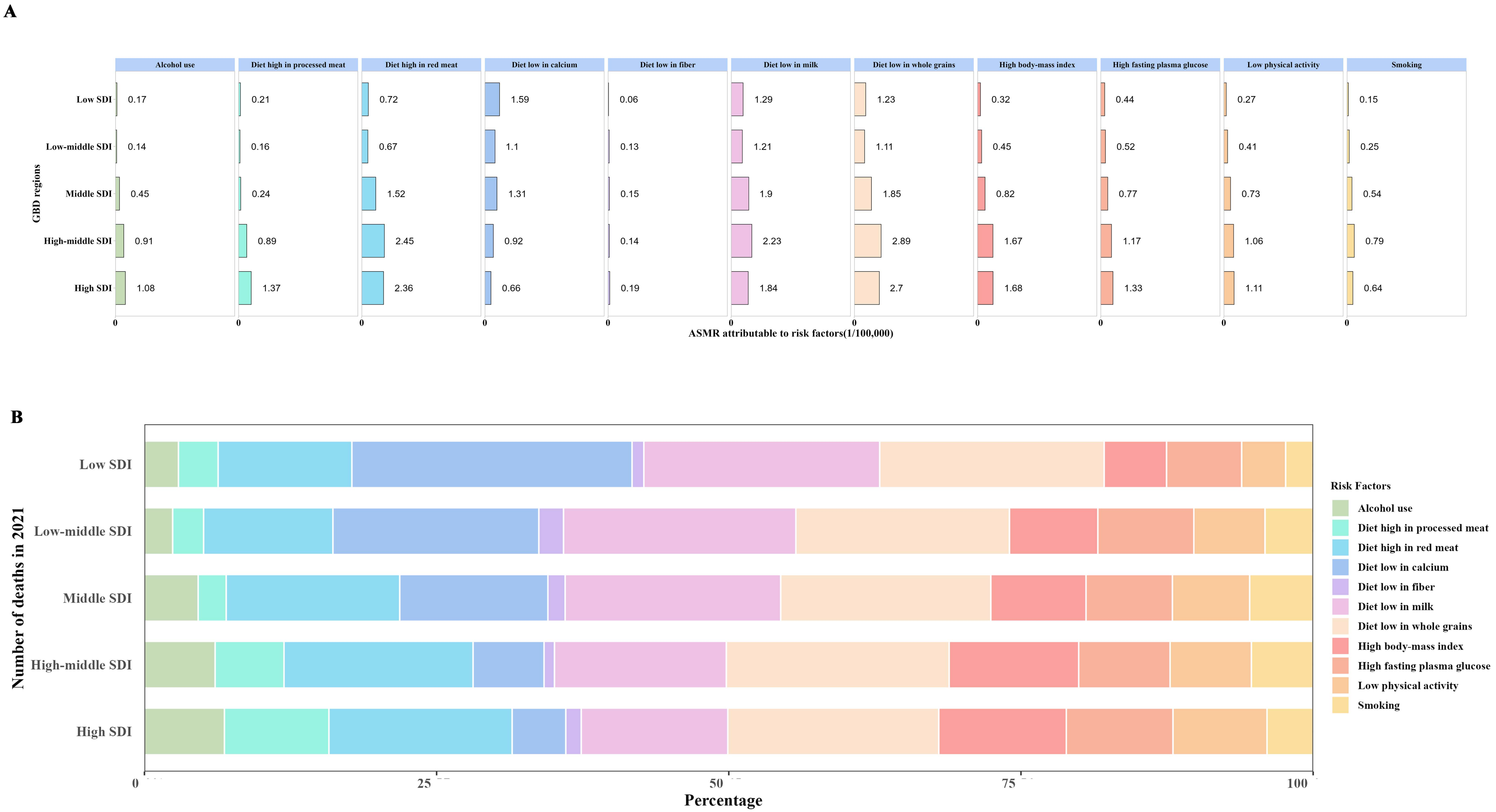

Fig.S7 Attributable deaths by CRC risk factors in five SDI levels regions in 2021. A: Numbers of ASMRs attributable to CRC risk factors in five SDI levels regions in 2021. B: Percentage of deaths attributable to CRC risk factors in five SDI levels regions in 2021. ASMR age-standardized mortality rate, CRC colorectal cancer, SDI socio-demographic index.

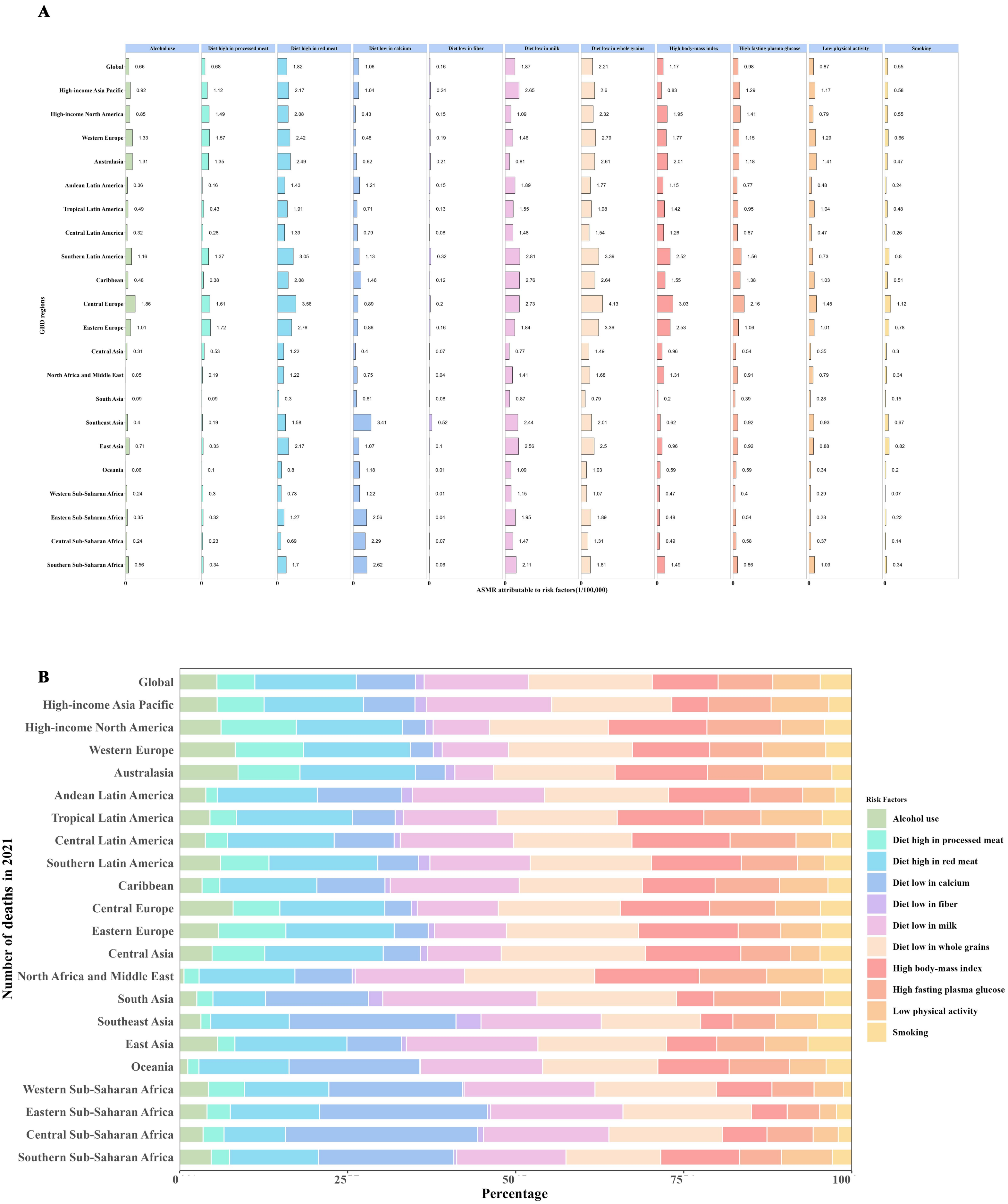

Fig.S8 Attributable deaths by CRC risk factors in 21 regions in 2021. A: Numbers of ASMRs attributable to CRC risk factors in 21 regions in 2021. B: Percentage of deaths attributable to CRC risk factors in 21 regions in 2021. ASMR age-standardized mortality rate, CRC colorectal cancer.

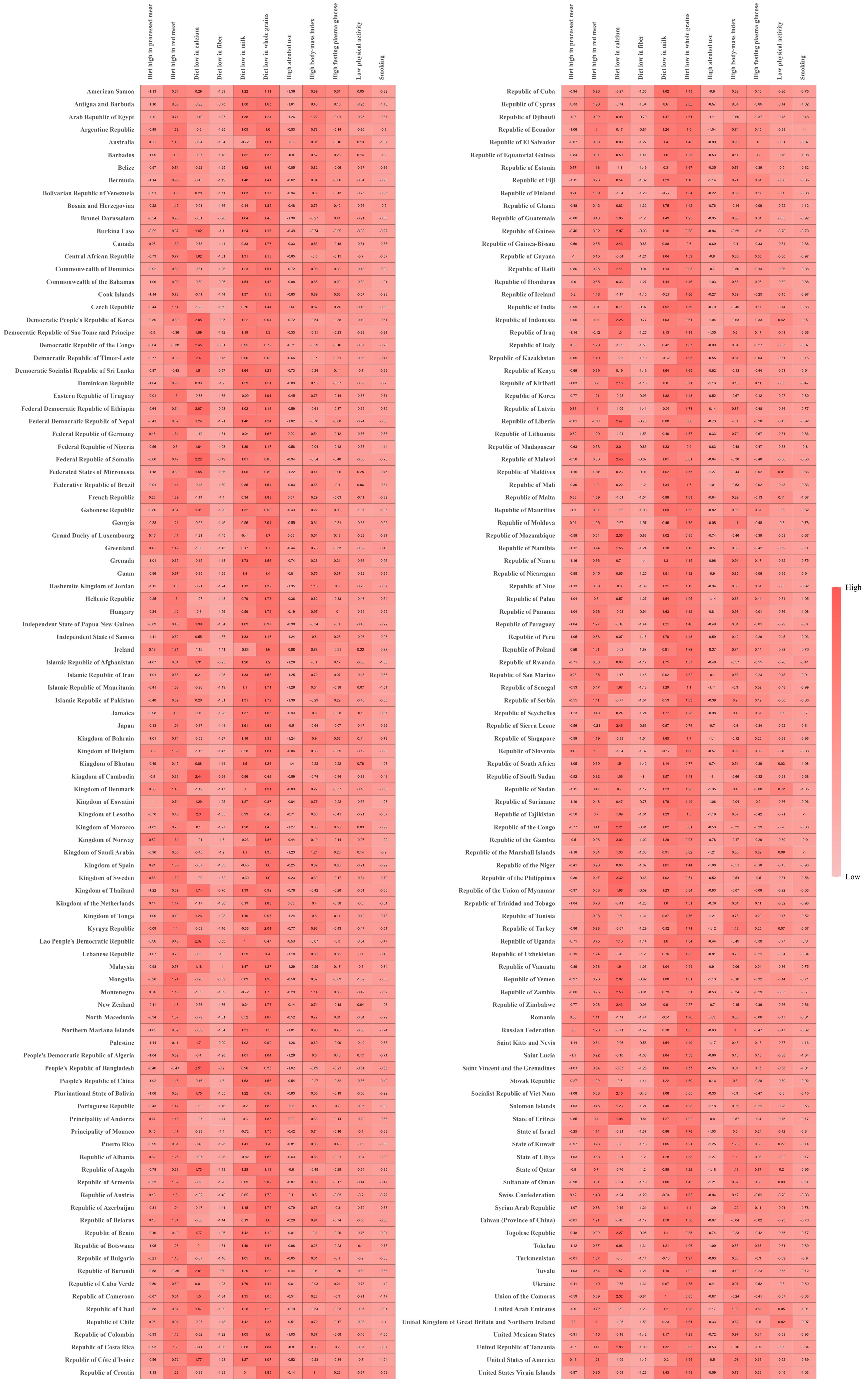

Fig.S9 Attributable age-standardized death rate by CRC risk factors in 204 countries and territories in 2021. The darker the red color, the higher the value. CRC colorectal cancer.

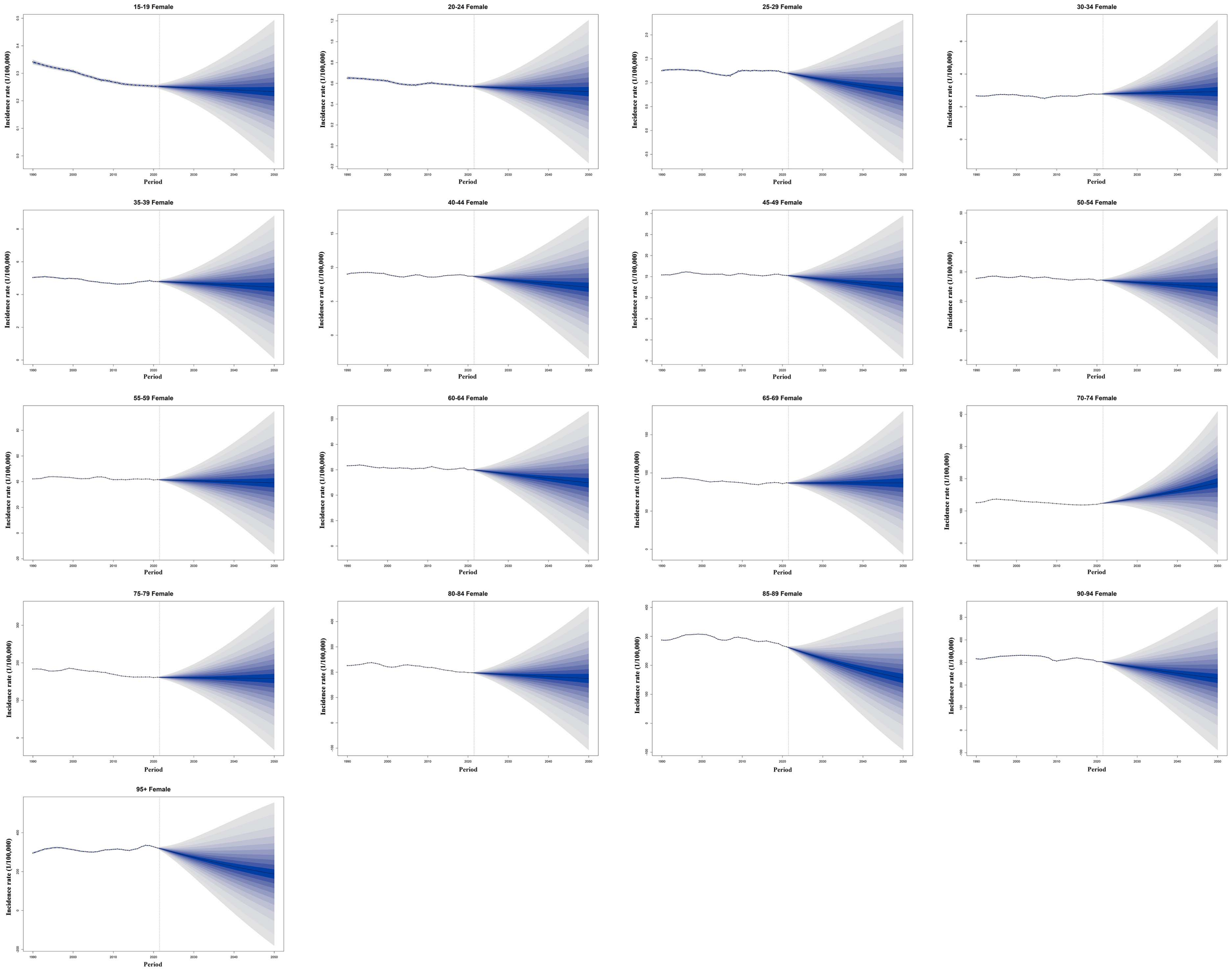

Fig.S10 Historical trends and future predictions of global female CRC incidence rates across different age groups from 1990 to 2050. CRC colorectal cancer.
